# Comparative Proteomics Analysis of Urine Reveals Down-Regulation of Acute Phase Response Signaling and LXR/RXR Activation Pathways in Prostate Cancer

**DOI:** 10.3390/proteomes6010001

**Published:** 2017-12-29

**Authors:** Katarina Davalieva, Sanja Kiprijanovska, Ivana Maleva Kostovska, Sotir Stavridis, Oliver Stankov, Selim Komina, Gordana Petrusevska, Momir Polenakovic

**Affiliations:** 1 Research Centre for Genetic Engineering and Biotechnology “Georgi D Efremov”, Macedonian Academy of Sciences and Arts, Skopje, Krste Misirkov 2, 1000 Skopje, Macedonia; skiprijanovska@manu.edu.mk (S.K.); maleva_i@manu.edu.mk (I.M.K.); momir@manu.edu.mk (M.P.); 2 University Clinic for Urology, University Clinical Centre “Mother Theresa”, 1000 Skopje, Macedonia; sotir.stavridis@medf.ukim.edu.mk (S.S.); oliver.stankov@medf.ukim.edu.mk (O.S.); 3 Institute of Pathology, Medical Faculty, University “St. Cyril and Methodius”, 1000 Skopje, Macedonia; s_komina@yahoo.com (S.K.); gordanap61@yahoo.com (G.P.)

**Keywords:** proteomics, non-invasive biomarker, prostate cancer, urine, LC-MS/MS, 2-D DIGE/MS

## Abstract

Detecting prostate cancer (PCa) using non-invasive diagnostic markers still remains a challenge. The aim of this study was the identification of urine proteins that are sufficiently sensitive and specific to detect PCa in the early stages. Comparative proteomics profiling of urine from patients with PCa, benign prostate hyperplasia, bladder cancer, and renal cancer, coupled with bioinformatics analysis, were performed. Statistically significant difference in abundance showed 20 and 85 proteins in the 2-D DIGE/MS and label-free LC-MS/MS experiments, respectively. In silico analysis indicated activation, binding, and cell movement of subset of immune cells as the top affected cellular functions in PCa, together with the down-regulation of Acute Phase Response Signaling and Liver X Receptor/ Retinoid X Receptor (LXR/RXR) activation pathways. The most promising biomarkers were 35, altered in PCa when compared to more than one group. Half of these have confirmed localization in normal or PCa tissues. Twenty proteins (CD14, AHSG, ENO1, ANXA1, CLU, COL6A1, C3, FGA, FGG, HPX, PTGDS, S100A9, LMAN2, ITIH4, ACTA2, GRN, HBB, PEBP1, CTSB, SPP1) are oncogenes, tumor suppressors, and multifunctional proteins with highly confirmed involvement in PCa, while 9 (AZU1, IGHG1, RNASE2, PZP, REG1A, AMY1A, AMY2A, ACTG2, COL18A1) have been associated with different cancers, but not with PCa so far, and may represent novel findings. LC-MS/MS data are available via ProteomeXchange with identifier PXD008407.

## 1. Introduction

Despite the intense research of prostate cancer (PCa) in the last years, it still remains the second most common cause of malignancy death in men of all ages [1]. Screening and detection of PCa are still mainly based on serum prostate specific antigen (PSA), which is more sensitive than specific for PCa [2] and produces a significant portion of false negative [3] and false positive [4] results. The high necessity for the more reliable screening tool has driven an extensive research which delivered a number of new potential biomarkers for screening and/or diagnosis of PCa [5,6,7,8]. These biomarkers are peptides, proteins, RNA transcripts, DNA methylations, and large-scale mitochondrial DNA deletions [8]. Some of these have already entered into clinical practice, but mainly as a supplement to PSA testing or as an additional supplement to biopsy-based diagnosis and prognosis of PCa.

It has been widely accepted now that understanding the pathophysiology of PCa as complex, heterogenic disease and the discovery of more sensitive/specific tools for disease detection requires a systems approach. Comparative proteomics studies have a significant and important role in this by aiming to detect and quantify proteins with altered abundance without prior biological knowledge, which subsequently may reveal candidate biomarkers. Proteomics studies have identified a large number of putative biomarkers for screening, differentiation between disease stages, and prognosis [9,10,11], and many are tested for their clinical utility. Several possible non-invasive or minimally invasive biomarkers sources, each with advantages and limitations, are under current investigation, including urine, serum, plasma, and prostatic fluids.

The goal of the PCa biomarker field, as well as the cancer biomarker field in general, is to develop simple, non-invasive tests that can allow for early cancer detection, classify the tumor so the patient can receive the most appropriate therapy, and monitor disease progression, regression, and recurrence [12]. The ideal PCa screening biomarker in addition to the high sensitivity and specificity for PCa should be non-invasive, easy accessible, and present at concentrations that are detectable by current technologies. The research so far has proven that one molecular marker based tests does not possess enough power for clinical use and this concept is replaced by the global assessment strategy and the development of multiplex biomarker assays. This makes sense when one considers the heterogeneity of prostate tumors, complex interactions between various molecules within a single pathway and the cross-talk between molecular pathways. Although detecting PCa using diagnostic markers still remains a challenge, the advent of new high-throughput proteomics techniques and systematic searches through rigorous experimental design and in-depth quantitative studies is driving biomarker discovery forward [13].

In order to detect proteins that are sensitive and specific enough to detect early stages of PCa, we performed a complex comparative proteomic study analyzing urine as a source for non-invasive biomarkers. In clinical proteomics, urine has become a preferred choice for biomarker discovery, especially for the detection and diagnosis of urological conditions because it can be sampled non-invasively in large quantities [14], contains soluble and stable proteins/peptides from plasma or urogenital tract, and it has a lower dynamics range of protein concentrations compared with other biofluids [15]. To date, several urinary biomarkers have been identified and considered for use in PCa screening, each with varying levels of evidence [16]. Our preliminary comparative study of urine from PCa and benign prostate hyperplasia (BPH) patients using two-dimensional Difference Gel Electrophoresis coupled with Mass Spectrometry (2-D DIGE/MS) , revealed a panel of acute phase response proteins as a non-invasive biomarkers for PCa [17]. Although the observed accuracy of the individual proteins was similar to the PSA, their combination yielded a greater accuracy when compared to individual tests. In the current study, using both 2-D DIGE/MS and label-free Liquid Chromatography—tandem Mass Spectrometry (LC-MS/MS), we compared urine samples from patients with early PCa stages and Gleason score between 6 and 7 with samples from patients with BPH and other urological cancers, namely bladder and renal. The main goal of this study was to discover a diagnostic biomarker or a set of biomarkers in urine, which were sufficiently sensitive to detect PCa in its early stages and specific enough to separate the disease from BPH or other urological cancers.

## 2. Materials and Methods

### 2.1. Samples

The urine samples were collected from men attending the University Clinic for Urology, University Clinical Centre “Mother Theresa,” Skopje, Republic of Macedonia in the period from January 2014 until June 2015. Informed consent for the use of these samples for research purposes was obtained from the patients in accordance with the Declaration of Helsinki. The study has been approved by the Ethics Committee of the Macedonian Academy of Sciences and Arts (Code: 011/2014). We analyzed 32 urine samples from patients with prostate cancer (PCa), benign prostate hyperplasia (BPH), bladder cancer (BC), and renal cancer (RC). The samples from the three cancer groups (PCa, BC, and RC) were collected from patients prior prostate, bladder, or kidney biopsy or prior surgery. The samples from non-cancer subjects (BPH Group) were collected prior to needle biopsy of the prostate gland or transurethral resection of the prostate (TURP). Patients that were diagnosed with BPH were followed in the course of one year, during which repeat biopsy was performed to exclude the possibility of developing cancer. The diagnosis of all individuals whose samples were included in this study was based on histological evaluation of the tissues obtained by biopsy or surgical procedure (Supplementary Document 1). The preselection of the urine samples from PCa was made according to the patient’s histological records documenting tumor grade, stage as well as pre-procedure PSA level. The preselection of the BPH patients was made solely by pre-procedure PSA level in order to match with the PCa group. The mean pre-operative PSA serum level was 7.4 ± 2.1 and 6.8 ± 1.8 (ng/mL) of PCa and BPH group, respectively. The Gleason score of PCa patients was 6.9 ± 1.1 (mean ± SD). All of the BC and RC patients were at stage I or II at the time of sample collection. All the patients were male and were matched for age (group mean 61.1–69.8 years).

### 2.2. Sample Preparation

The first morning urine (3–10) mL was collected from the patients prior to clinical intervention and was stored on ice for a short period (<1 h). Samples were centrifuged at 1000× *g*, for 10 min to remove cell debris, aliquoted in 1.5 mL tubes and stored at −80 °C until use.

2-D DIGE/MS analysis: Urine proteome was isolated from 100 μL urine in five or more replicates per sample using 2-D Clean-UP Kit (GE Healthcare, Little Chalfont, UK), according to the manufacturer’s instructions. The pellets from each replicate were dissolved in 10 μL of UTC buffer (8 M Urea, 2 M Thiourea, 4% CHAPS) and pooled together. Samples were quantified in duplicate by Bradford [18] against a standard curve of Bovine Serum Albumin (BSA) and stored at −80 °C.

LC-MS/MS analysis: The starting amount of urine per isolation was 1 mL. Total urine proteome was precipitated using a methanol/chloroform extraction in the ratio 1:1.5:0.4 (urine:methanol:chloroform), washed with 1 volume of methanol, and then dissolved in 50 mM ammonium bicarbonate containing 1 mg/mL RapiGest^TM^ detergent (Waters Corp., Milford, MA, USA). Dithiothreitol (DTT) was added to 5 mM final concentration and the solution was sonicated and boiled for 5 min. Protein concentration was adjusted to 0.6 µg/µL in RapiGest buffer using starting amount of 20 µg protein per sample. The samples were reduced with DTT to 5 mM final concentration for 30 min. at 60 °C, alkylated with iodoacetamide (IAA) to 15 mM final concentration for 30 min. in the dark, at room temperature, and digested with 0.05 µg/µL trypsin sequencing grade (Roche, Basel, Switzerland) in trypsin:protein ratio of 1:100. To the prepared sample containing 200 ng/µL protein, yeast alcohol dehydrogenase (ADH, UniProt P00330) digest (MassPREP ADH Digestion Standard, Waters Corp., Milford, MA, USA) was added as an internal control to 25 fmol/µL final concentration. All of the solvents used for protein isolation, buffers, and enzyme solutions were Ultra CHROMASOLV^®^ grade, tested for UHPLC-MS (Fluka, Buchs, Switzerland).

### 2.3. 2-D DIGE and Mass Spectrometry Identification

The first dimension of the 2-D DIGE analysis was performed on 24 cm ImmobilineDrystrip gels (GE Healthcare) with linear pH 4–7 gradient, while the second dimension was carried out onto 12.5% homogeneous polyacrylamide gels using the EttanDALTsix system (GE Healthcare, Little Chalfont, UK). Labeling of the proteins with the CyDye DIGE Fluor minimal dyes (GE Healthcare) and experimental procedures regarding 2-D DIGE, preparative 2-D gel for spot picking and mass spectrometry identification were the same as previously published [17]. Protein identifications were based on MASCOT search (version 2.4.01, MatrixScience, London, UK) of all human proteins and sequence information from Swiss-Prot (version 2016_05, 20202 sequences) and NCBInr (version 20160604, 320591 sequences) through the mMass software version 5.5.0 [19]. Search parameters were set as follows: fixed modification Carbamidomethyl (C), variable modifications Acetyl (Protein N-term), and Oxidation (M), up to one missed tryptic cleavage and a peptide mass tolerance of ±0.4 Da. Positive identification was based on a Mascot score greater than 56, above the significance level (*p* < 0.05) and four or more peptide matches per identification.

### 2.4. Label-Free LC-MS/MS Analysis

A label-free LC-MS/MS protein profiling was performed using a nano liquid chromatograph ACQUITY UPLC M-Class (Waters Corp., Milford, MA, USA) coupled with Synapt G2Si HDMS QTOF mass spectrometer (Waters Corp., Milford, MA, USA) using MS^E^ data-independent scanning. For each sample, one test run and initial data processing with ProteinLynx Global Server (PLGS, version 3.0.3, Waters Corp., Milford, MA, USA) was done for quality assurance testing, verifying instrument performance, and determination of the exact protein concentration. Optimal loading for MSe runs was determined by testing several samples, starting from 200–400 ng per run and processing in PLGS. Subsequently, two 120 min LC-MS runs were carried out for each sample using the optimal loading of 250 ng per sample/run, providing a total of 40 chromatograms (10 per group) in resolution mode. Peptides were trapped on a Symmetry C18 Trap column, 5 µm particles, 180 µm × 20 mm (Waters), for 3 min at 8 µL/min in 0.1% solvent B (0.1% formic acid in acetonitrile)/99.9% solvent A (0.1% formic acid, aqueous). Weak needle wash was with 1% acetonitrile, 0.1% trifluoroacetic acid aqueous, while strong needle wash was with 0.1% trifluoroacetic acid in acetonitrile. Peptides were separated on a 120-min run on a 75 µm ID × 15 cm HSS T3, 1.8 µm particle diameter reverse phase column (Waters Corp., Milford, MA, USA) at 300 nL/min flow rate, with an acetonitrile/formic acid gradient. For the analytical separation, solvent B was increased in a 90 min linear gradient between 3 and 40%, and post-gradient cycled to 95% B for 2 min, followed by post-run equilibration at 3% B. Spectra were recorded in resolution positive ion mode with a Synapt G2 quadrupole-time-of-flight HDMS mass spectrometer (Waters Corp., Milford, MA, USA). Source settings included capillary voltage of 3.2 kV, sampling cone at 40 V, and source temperature of 80 °C. The cone gas N_2_ flow was 30 L/h. Analyzer settings included quadrupole profile set with mass 1 as 400 (dwell time 20% and ramp time 20%), mass 2 as 500 (dwell time 20% and ramp time 40%), and mass 3 as 600. Collision energy was off for low energy scan and ramped from 20 to 40 V for the high energy scan with a collision gas flow (Ar) of 2.0 mL/min. Alternate 0.5 s scans at low and high energy were recorded for the range between 50 and 2000 *m*/*z*. A reference sprayer was operated at 500 nL/min to produce a lockmass spectrum with Glu-1-Fibrinopeptide B (EGVNDNEEGFFSAR) (*m*/*z* 785.8426) and leucine enkephalin (YGGFL) (*m*/*z* 556.2771) every 45 s. The concentrations of Glu-1-Fibrinopeptide B and leucine enkephalin in the reference solution (50% acetonitrile, 0.1% formic acid) were 100 fmol/µL and 200 pmol/µL, respectively.

Spectra were analyzed with PLGS. Peak processing settings were low energy threshold of 300 counts and elevated energy threshold of 40 counts. Data was searched against the UniProtKB database of manually annotated and reviewed human sequences containing 20,139 proteins (August 2016), to which yeast alcohol dehydrogenase (UniProt P00330) sequences were added. Search settings included up to one missed cleavage, carbamidomethyl cysteine as a fixed modification, and the methionine oxidation as variable modification. A minimum of two fragment ion matches was required per peptide identification and five fragment ion matches per protein identification, with at least two peptide matches per protein identification. The false discovery rate was set to 4%. Matching of peptides by accurate mass and retention time across multiple LC/MS/MS chromatograms and statistical analyses were performed with Progenesis QI for Proteomics (version 3.0) (Nonlinear dynamics, Waters Corp., Milford, MA, USA). Search parameter settings and the database were the same as in PLGS. The protein abundances in individual runs were normalized using the run that is least different from all other runs. Normalization to all proteins was selected. Quantification was done by Hi-N, using three most abundant peptides per protein. Positive identification was based on two or more peptide matches per identification. The mass spectrometry proteomics data have been deposited in the ProteomeXchange Consortium (http://proteomecentral.proteomexchange.org) via the PRIDE partner repository [20] with data set identifier PXD008407 and 10.6019/PXD008407.

### 2.5. Analysis of the Proteomics Data

Proteins with differential abundance among the four groups were considered with fold change ≥ 1.5 and Anova ≤ 0.05. *p*-values between groups were calculated using *t*-test (independent, 2-tailed, unequal variance) and subsequently false discovery rate (FDR) correction for multiple testing [21] was applied. For an overview of the cellular localization, molecular function, and biological processes in which proteins with differential abundance are involved, we used STRAP 1.5 software for rapid automatic annotation of proteins that uses information from the Uniprot and European Bioinformatics Institute (EBI) QuickGO databases [22].

Pathway analysis was carried out for proteins with differential abundance between compared groups using Ingenuity Pathway Analysis (IPA) (QIAGEN Bioinformatics). IPA is a web-based software application for the analysis, integration, and interpretation of data derived from ‘omics experiments that helps in analysis of the data and pinpoints new targets or candidate biomarkers within the context of biological systems. The identified proteins were mapped to the most significant networks, diseases, molecular and cellular functions, generated from previous publications and public protein interaction databases using the Ingenuity Knowledge Base as a reference set. A *p* value calculated with the right-tailed Fisher’s exact test was used to yield a network’s score and to rank networks according to their degree of association with our data set.

The tissue specificity of the selected proteins with differential abundance in PCa was evaluated based on protein localization of the corresponding proteins in several organs from the male reproductive system and cancers of the prostate, kidney, and urothelium, using publicly available database The Human Protein Atlas version 15 [23] (http://www.proteinatlas.org/). Protein localization is determined based on antibody staining (immunohistochemistry, Western blot) and protein arrays.

## 3. Results

### 3.1. 2-D DIGE Analysis

Fifty four spots showed statistically significant difference in abundance between all four of the analyzed groups (ANOVA, *p* < 0.05) (Table 1 and Supplementary Document 1). These spots corresponded to 20 unique proteins. Seven out of twenty proteins were observed differentially abundant between PCa and BPH (KNG1, IGHA1, IGHA2, HBB, ITIH4, AMBP, and MASP2), nineteen differed in abundance between PCa and BC (TF, KNG1, IGHA1, IGHA2, ATE1, FGG, AZGP1, HP, FGA, ITIH4, AMBP, PTGDS, IGKC, APCS, VMO1, CD59, RBP4, MASP2, and HSPG2) and only two proteins (IGHA1 and IGHA2) showed differential abundance between PCa and RC groups. Six proteins showed the same trend in PCa when compared to other groups: KNG1, ITIH4, AMBP, and MASP2 were up-regulated and IGHA1 and IGHA2 were down-regulated in PCa when compared to BPH, BC, and RC groups.

According to the Human Protein Atlas database, six proteins (ATE1, AZGP1, ITIH4, PTGDS, VMO1, MASP2) are expressed in normal as well as tumor prostate tissue by immunohistochemical staining with two additional proteins (AMBP, CD59), localized only in normal prostate tissue and five (TF, HP, HBB, APCS, RBP4) found only in tumor tissue. The identified proteins are enrolled in immune processes (FGG, FGA, ITIH4, AZGP1, HP, AMBP, MASP2, IGKC, CD59), protein metabolism (KNG1, ATE1, APCS), molecular transport (TF, HBB, and RBP4), cell growth (HSPG2), and energy metabolism (PTGDS).

### 3.2. LC-MS/MS Analysis

We have identified a total of 226 proteins in the patient’s urine in the four investigated groups (Supplementary Table S2, ALL), based on a total of 1882 peptides (Supplementary Table S3). We focused on 179 proteins, which were identified based on two or more peptides (Supplementary Table S2, Report). Statistically significant difference in the abundance (Anova ≤ 0.05) and fold change of ≤1.5 showed 85 unique proteins, of which 44 up-regulated and 41 down-regulated in PCa (Table 2). Comparison between groups revealed: 1 protein up-regulated in PCa as compared to BPH, BC, and RC; 32 proteins with differential abundance in PCa when compared to both BPH and BC (15 up-regulated and 17 down-regulated in PCa); 2 proteins with differential abundance in PCa as compared to both BC and RC (1 up-regulated and 1 down-regulated in PCa); 18 proteins with differential abundance in PCa compared to BPH (1 up-regulated and 17 down-regulated in PCa); 30 proteins with differential abundance in PCa when compared to BC (25 up-regulated and 5 down-regulated in PCa); 2 proteins with differential abundance in PCa as compared to RC (1 up-regulated and 1 down-regulated in PCa).

**Figure 1:**
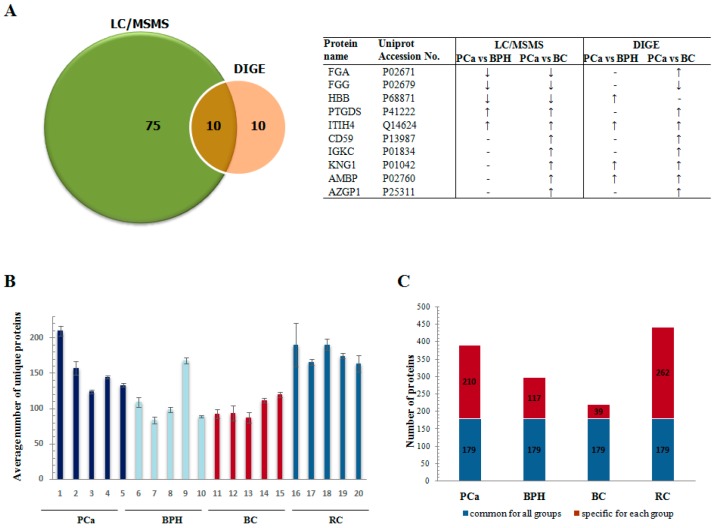
Characterization of the 2-D DIGE and LC-MSMS experiment in terms of number of identified common and unique proteins. (**A**) Number of proteins with altered abundance in PCa in 2-D DIGE and LC-MSMS experiment and identity of the mutually identified proteins (**B**) Average number of unique proteins identified in duplicate LC-MS/MS runs for each sample; (**C**) Number of unique and shared proteins in four groups in the LC-MS/MS experiment. Numbers represent proteins identified based on two or more peptides.

Comparison with the differentially abundant proteins in PCa using2-D DIGE revealed 10 mutually identified proteins: FGA, FGG, HBB, PTGDS, ITIH4, CD59, IGKC, KNG1, AMBP, and AZGP1 (Figure 1A). The average number of unique proteins identified in duplicate LC-MS/MS runs for each sample and the number of unique and shared proteins in four groups in the LC-MS/MS experiment are shown in Figure 1B,C, respectively.

To gain insight into the cell/tissue origin and biological implication of the proteins with altered abundance, we have analyzed the protein localization in several organs from the male reproductive system and the corresponding cancers and the reported molecular functions, cellular localization, and involvement in biological processes. Here, we focused only on the 35 proteins that showed differential abundance in PCa when compared to more than one group. Detailed identification and quantification data of these proteins obtained by Progenesis QI for proteomics version 3.0 (Nonlinear dynamics, Waters Corp., Milford, MA, USA) is given in Supplementary document 2.

**Figure 2:**
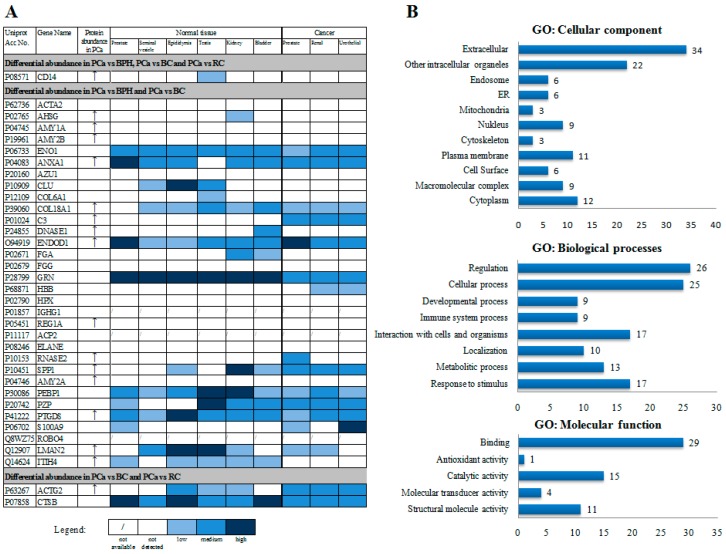
Characterization of the 35 proteins with altered abundance in PCa in more than one group comparison in terms of organ specificity and GO Annotations. (**A**) The specificity was evaluated based on protein localization of the corresponding proteins in several organs from the male reproductive system and cancers of the prostate, kidney and urothelium, using publicly available database The Human Protein Atlas (http://www.proteinatlas.org/). The database contains information about protein localization in prostate, seminal vesicle, epididymus, testis, kidney and bladder as well as detection of these proteins in prostate, renal and urothelial cancer tissues. Protein localization is determined based on antibody staining (immunohistochemistry, Western blot) and protein arrays; (**B**) GO Annotations of proteins obtained by STRAP 1.5 software for rapid automatic annotation of proteins using the Uniprot and EBI QuickGO databases. The *y*-axis represents the GO terms that are associated with the protein set while *x*-axis represents the number of GO annotations per GO term.

The prostate gland specificity of the 35 proteins with altered abundance in PCa was estimated based on the data from publicly available database The Human Protein Atlas (Figure 2A).

Out of 35 proteins, 16 (CD14, ACTA2, AHSG, AMY1A, AMY2B, AZU1, CLU, COL6A1, DNASE1, FGA, FGG, HBB, HPX, REG1A, ELANE, AMY2A) have not been detected at protein level in normal prostate or PCa tissues. These are secreted proteins, some of them with distinct plasma positivity and the remaining with selective expression in various cells/organs (immune, muscle, stromal, erythrocytes, bone marrow, pancreas, extracellular matrix). The data for protein localization of three proteins (IGHG1, ACP2, ROBO4) was not available. The remaining 16 proteins with altered abundance in our study have detected protein localization in either normal prostate or PCa tissues. Among these proteins, for 11 proteins (ENO1, COL18A1, C3, GRN, RNASE2, SPP1, PEBP1, PZP, LMAN2, ACTG2, CTSB), the data for the relative protein abundance between PCa and normal prostate tissues in The Human Protein Atlas corresponds with our comparative proteomics data. These proteins are either ubiquitously expressed (ENO1, GRN, PEBP1, PZP, CTSB, LMAN2, COL18A1, SPP1, ACTG2), or have selective expression in a subset of cells, such as bone marrow, subset of inflammatory cells (RNASE2), and liver (C3). For the remaining five proteins, immunohistochemistry data shows no change between normal and PCa tissue (ENDOD1, S100A9) or opposite to our findings (ANXA1, PTGDS, ITIH4). The majority of the 35 proteins is involved in regulation and cell processes. The major molecular function of these proteins are binding and catalytic activity, and almost all are secreted (Figure 2B).

**Figure 3:**
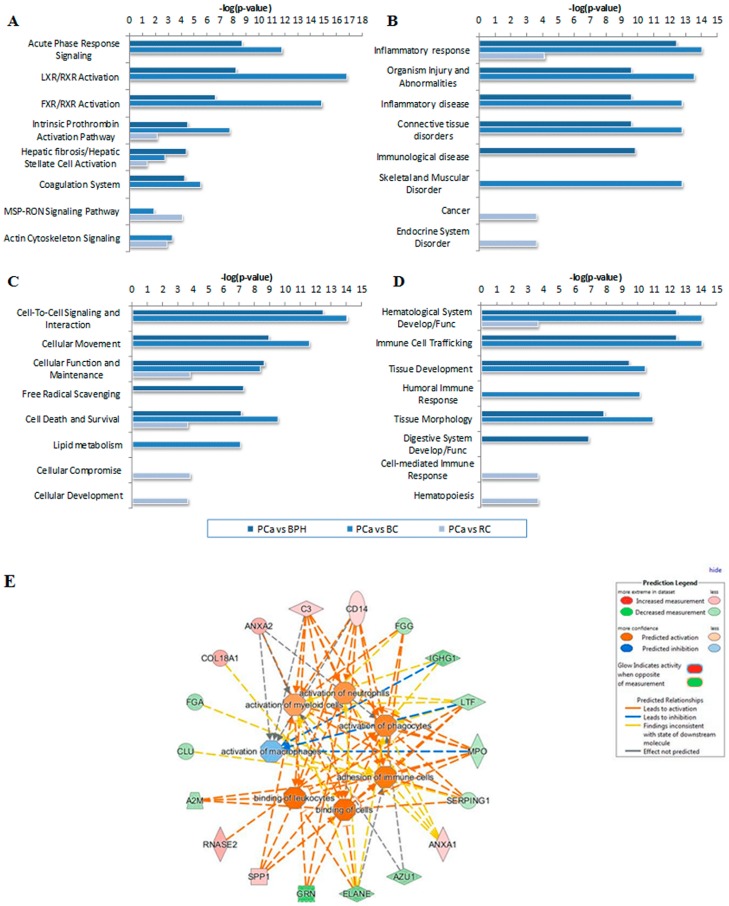
Ingenuity Pathway Analysis (IPA) analysis of proteomics data. The charts represent the top significantly associated (**A**) Canonical pathways (**B**) Diseases and Disorders (**C**) Molecular and Cellular Functions (**D**) Physiological System Development and Function with the proteins with altered abundance in PCa. (**E**) Top affected cellular functions in PCa were activation, binding, and cell movement of subset of immune cells present during acute inflammatory response, and also present in the later stages of the disease progression.

In silico analysis was performed using IPA, by importing Uniprot Accession of 179 proteins and setting the expression *p*-value cutoff of 0.05 and fold change ±1.5. The proteins with differential abundance in each comparison between groups were associated with canonical pathways, diseases, disorders, and biofunctions (Figure 3). Among the canonical pathways, the top significant association of the proteins that were altered in PCa was with the Acute Phase Response Signaling pathway (*p* = 1.97 × 10^−9^ for PCa vs. BPH and *p* = 1.51 × 10^−12^ for PCa vs. BC), followed by LXR/RXR activation and FXR/RXR activation pathways (Figure 3A). Eight proteins (A2M, AHSG, C3, FGA, FGG, HPX, ITIH4, SERPING1) from our dataset are included in Acute Phase Response Signaling pathway from PCa vs. BPH comparison and 11 proteins (AMBP, APOA1, APOH, AHSG, C3, C4A, F2, FGA, FGG, HPX, ITIH4,) from PCa vs. BC comparison. The majority of these proteins are also involved in LXR/RXR activation and FXR/RXR activation pathways. Due to the low number of proteins with altered abundance in PCa vs. RC comparison, the association of this data set with canonical pathways showed only borderline significance. The association of our datasets with the known diseases and disorders showed the highest scores for Inflammatory response and Organism Injury and Abnormalities. In the category Inflammatory response, the highest score was for the subcategories “inflammatory response of cells” and “inflammation of organ”, where 17 proteins from the PCa vs. BPH dataset (A2M, AHSG, ANXA1, ANXA2, AZU1, C3, CD14, COL18A1, ELANE, FGG, GRN, IGHG1, LTF, MPO, RNASE2, SERPING1, SPP1), 24 proteins from the PCa vs. BC dataset (ACTA2, AMBP, AMY2A, AMY2B, ANXA1, APOA1, APOH, C3, C4A, CD14, CD55, CLU, CT58, CTSD, DNASE1, ENO1, F2, HBB, HPX, IGHG1, IGKC, KNG1, PGLYRP1, PIGR), and two proteins from the PCa vs. RC dataset (CD14, CT58) showed association with *p*-values of 1.74 × 10^−11^, 2.60 × 10^−14^ and 7.12 × 10^−5^, respectively (Figure 3B). The association with molecular and cellular functions showed the highest scores for Cell-To-Cell Signaling and Interaction where the same subcategory “activation of phagocytes” had the highest *p*-values (3.44 × 10^−13^ and 8.70 × 10^−15^) in PCa vs. BPH and PCa vs. BC comparisons, respectively (Figure 3C).

Fourteen proteins (ANXA1, ANXA2, AZU1, C3, CD14, ELANE, FGG, GRN, IGHG1, LTF, MPO, RNASE2, SERPING1, SPP1) from PCa vs. BPH dataset and 17 proteins (ANXA1, APOA1, APOH, AZU1, C3, C4A, CD14, ELANE, F2, FGG, GRN, IGHG1, KNG1, RNASE2, S100A9, SPP1, UMOD) from PCa vs. BC dataset are involved in this subcategory. The highest association in the Physiological System Development and Function showed Hematological System Development and Function and Immune Cell Trafficking with *p*-values ranging from 3.44 × 10^−13^ (PCa vs. BPH) to 8.70 × 10^−15^ (PCa vs. BC) (Figure 3D). The proteins involved here are the same as in Cell-To-Cell Signaling and Interaction, subcategory “activation of phagocytes”. In general, the top affected cellular functions in all PCa comparisons were Cell-To-Cell Signaling and Interaction, Hematological System Development and Function, Immune Cell Trafficking, and Inflammatory response with the highest associations for activation and cell movement of phagocytes, activation of leukocytes, cell movement of neutrophils, and inflammation (Figure 3E).

**Figure 4:**
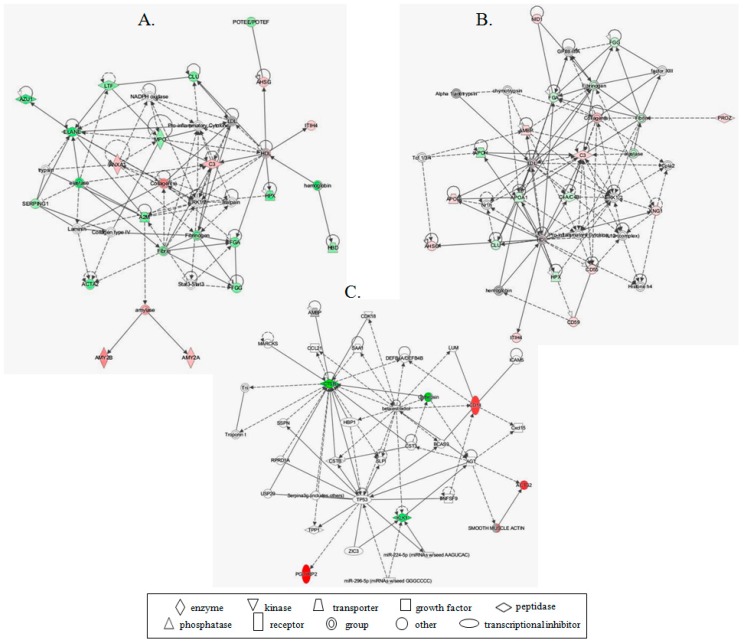
Networks associated with proteins with differential abundance, according to **IPA**. (**A**) Highest ranked protein network of functional associations between 19 proteins with differential abundance in PCa vs. BPH-Cell-To-Cell Signaling and Interaction, Hematological System Development and Function and Immune Cell Trafficking; (**B**) Highest ranked protein network of functional associations between 17 proteins with differential abundance in PCa vs. BC-Hummoral Immune Response, Inflammatory response, Hematological System Development and Function; (**C**) Highest ranked protein network of functional associations between five proteins with differential abundance in PCa vs. RC-Cellular Movement, Hematological System Development and Function and Immune Cell Trafficking. The network is graphically displayed with proteins as nodes and the biological relationships between the nodes as lines. The proteins are distributed according to the subcellular localization. The color of the shapes indicates the degree of over-expression (red) or under-expression (green) of the corresponding protein in PCa. Direct connection between molecules is represented by a solid line while indirect connection with broken line. The length of a line reflects published evidence supporting the node-to-node relationship concerned.

The highest ranked protein networks of functional associations between proteins in group comparisons were “Cell-To-Cell Signaling and Interaction, Hematological System Development and Function, and Immune Cell Trafficking” (score 46) for PCa vs. BPH dataset, “Hummoral Immune Response, Inflammatory response, Hematological System Development and Function” (score 37) for PCa vs. BC dataset and “Cellular Movement, Hematological System Development and Function and Immune Cell Trafficking” (score 15) for PCa vs. RC dataset (Figure 4). Proteins from our dataset were closely connected in the network through several nodes, such as extracellular-signal-regulated kinase 1/2 (ERK1/2), signal transducer and activator of transcription 3 (Stat3), Pro-inflammatory cytokines, and Tumor protein p53 (TP53).

## 4. Discussion

In our previous comparative study of urine from PCa and BPH patients, we identified a set of putative candidates [17] as non-invasive biomarkers for PCa. This prompted us to perform a larger comparative study, including two additional urological cancers, namely invasive low grade papillary urothelial carcinoma and early stage clear cell renal carcinoma, in order to test if the urine contains proteins sensitive to detect early stages of PCa and is specific enough to separate the disease from BPH and other urological cancers.

Urine samples that were used in this study were collected and stored with minimal processing and manipulation, following the recommended standards for clinical proteome analysis [24]. We did not deplete the highly abundant proteins to exclude the possibility of losing low abundant or low molecular weight proteins that exist in complexes with it. This strategy was additionally backed up by the recent systematic study, which showed that no added value of urine depletion strategies can be observed and that depletion did not yield a higher number of protein identifications in samples from either control or diseased patients [25]. Using both gel-based (2-D DIGE/MALDI-TOF) and gel-free (label free LC-MS/MS) proteomics methods, we sought to take advantage of each method in identification and quantification of proteins, and to increase the statistical significance of the results.

2-D DIGE analysis pointed out to 20 distinct proteins with differential abundance among the analyzed groups. Majority of these are plasma proteins with highest expression in liver, involved in immune processes and transport. DIGE analysis produced similar output as our previous gel-based study of urine [17], as well as other studies analyzing body fluids in search of diagnostic markers for PCa [26,27,28]. From a clinical point of view, the highest potential for discriminating PCa from other diseases showed several plasma proteins, such as KNG1, MASP2, ITIH4, AMBP, IGHA1, and IGHA2. In addition to the highly abundant plasma proteins, DIGE analysis also revealed a number of proteins that were expressed in normal and/or tumor prostate tissue, and also one protein, namely zinc alpha 2 glycoprotein (AZGP1), which is predominantly expressed in the prostate. AZGP1 is known candidate biomarker for PCa, which is found over expressed in blood and seminal plasma [26,29,30,31].

LC-MS/MS analysis revealed a much higher number of putative candidates and more prostate-specific proteins compared to DIGE analysis as expected. The list contained a number of already known proteins with altered abundance in PCa, obtained by comparative proteomics studies, such as Alpha-2-HS-glycoprotein (AHSG), Hemopexin (HPX), Protein S100-A9 (S100A9), Inter-alpha-trypsin inhibitor heavy chain H4 (ITIH4), Fibrinogen chains, Alpha-2-macroglobulin (A2M), Apolipoprotein A-IV (APOA4), Afamin (AFM), Kininogen-1 (KNG1), Protein AMBP (AMBP), Uromodulin (UMOD), members of the complement cascade, and zinc alpha 2 glycoprotein (AZGP1) (reviewed extensively in [10,11]).

**Figure 5:**
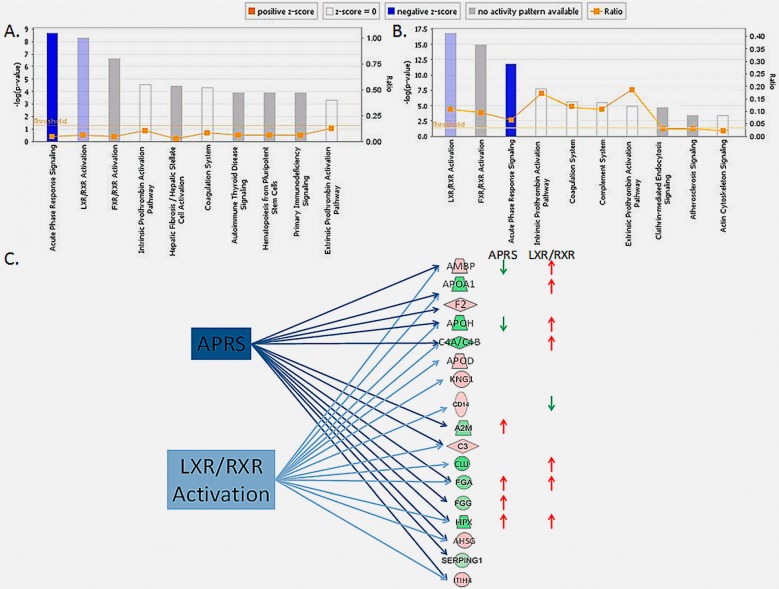
The direction of the significantly affected canonical pathways in PCa according to in silico analysis with IPA. Negative regulations of the acute phase response signaling and LXR/RXR activation pathway were observed in PCa in comparison with BPH (**A**) and BC (**B**); This observation is based on the differential abundance of the proteins involved in these pathways some of which showed an opposite expression level than in the canonical pathways (**C**). The shape of the protein symbol indicates the type of the protein as in the Figure 4 legend. The color of the shapes indicates the degree of over-expression (red) or under-expression (green) of the corresponding protein in PCa. The color of the arrows indicates over-expression (red) or under-expression (green) of the corresponding protein in the canonical pathways.

The IPA analysis shows that multiple pathways are involved in the systemic response to PCa, with the most prominently affected cellular pathways being the acute phase response signaling and LXR/RXR activation pathway. Both pathways were down-regulated evidenced by the negative z-score, which predicts the direction of the process based on the directional change of molecules in the dataset as compared to the canonical pathway (Figure 5).

The activation of the acute phase response in cancer is due to the mechanism of its development, which has many links with inflammation [32]. In addition, a number of epidemiological studies have provided evidence that inflammation establishes an environment that promotes the initiation and growth of a malignancy [33]. In line with this, activated immune cells (lymphocytes, macrophages, myeloid cells, and neutrophils) are found to be significantly increased in the PCa tissues and postulated to cause multiple levels of alterations, which sustain tumor cell survival and proliferation [34]. The top affected cellular functions in PCa in this study, according to IPA, were the activation and binding of leukocytes, activation of myeloid cells, macrophages, neutrophils, phagocytes, and adhesion of immune cells (Figure 3E). In this context, our study is in concordance with previous observations. So, indirectly, the pattern of the differentially expressed proteins in this study pointed to activation of immune cells that are the main players in the acute phase response and also present in the later stages of the disease progression.

The acute phase response is characterized by alteration in the concentrations of a number of plasma proteins, produced by the liver. Numerous reports have correlated altered levels of various acute phase proteins with different types of cancers [35]. Although alterations in the concentration of the acute phase proteins are observed in a wide range of diseases, different patterns are observed for distinct types, subtypes, and even stages of cancer. In this study, out of 13 acute phase proteins that we found with differential abundance in PCa, seven proteins, namely A2M, AHSG, FGG, HPX, SERPING1, AMBP, and C4A have displayed opposite abundance levels than in the canonical pathway. Among them, positive acute phase proteins A2M, FGG, HPX, SERPING1, and C4A displayed decreased levels, while negative acute phase proteins AHSG and AMBP increased levels in PCa, resulting in down-regulation (PCa vs. BPH *z*-score= −1.3; PCa vs. BC *z*-score= −0.8). This is something that we have already observed in urine of PCa patients previously, but to a smaller extent [17]. In view of this association of different patterns of acute phase proteins with PCa, this study confirms that there is a strong potential in using acute phase proteins fingerprinting as a complementary biomarker panel for PCa detection. Whether this pattern is constant throughout the PCa progression or seen only at the early stages of the disease remains to be investigated in future studies as this was not the aim of this study. In addition, the biological mechanism behind this change is another puzzling aspect that deserves further attention.

Liver X Receptors/Retinoid X Receptors (LXR/RXR) activation pathway is involved in cholesterol transport, glucose metabolism, and the modulation of inflammatory responses [36,37]. There is now accumulating evidence to support the involvement of LXRs in a variety of malignancies [38]. The association between disturbances in cholesterol metabolism and early stages of prostate carcinogenesis, has been made almost a century ago [39]. Follow-up studies confirmed the increased epoxycholesterol levels, including products of the LXR target gene SREBF1 during prostate cancer disease progression [40] and direct implication of LXR in PCa based on inhibited proliferation of prostate cancer cell lines by LXR agonists [41]. Later on, LXR activity was shown to be directly down-regulated by the androgen receptor, which reduces LXR target gene expression [42]. In line with this, the observed down-regulation of the LXR/RXR activation pathway in our study (PCa vs. BPH *z*-score = −0.4; PCa vs. BC *z*-score = −0.3) is in concordance with the observed trend in PCa.

The most promising biomarkers to differentiate PCa from other conditions were 35, which showed a differential abundance in PCa when compared to more than one group. We performed an extensive and rigorous literature analysis of each of these 35 putative biomarkers in order to evaluate possible links with PCa. We found that 20 proteins (CD14, AHSG, ENO1, ANXA1, CLU, COL6A1, C3, FGA, FGG, HPX, PTGDS, S100A9, LMAN2, ITIH4, ACTA2, GRN, HBB, PEBP1, CTSB, SPP1) have been associated with PCa, as well as with other cancers previously. Detailed information regarding the molecular/biological function, involvement in diseases, and association with PCa and other cancers of each of these proteins is given in Supplementary Table S4. This group contains several proteins with highly confirmed involvement in PCa progression, such as Osteopontin (SPP1), Complement C3 (C3), Granulin (GRN), Clusterin (CLU), Phosphatidylethanolamine-binding protein 1 (PEBP1), protein S100A9, and Alpha-enolase (ENO1).

Osteopontin (SPP1) is a multifunctional cytokine that is known to be involved in numerous physiologic functions and is associated with progression of various cancers. Its aberrant expression and/or splicing is functionally responsible for undesirable alterations in disease pathologies, specifically cancer, where it is implicated in promoting invasive and metastatic progression [43]. Overexpression of SPP1 in PCa has been confirmed by a number of studies (for detailed references please see Supplementary Table S4) followed by confirmation that SPP1, together with cyclin D1, are the key mediators of prostate cancer growth and metastatic progression within the activated TGFβ/BMP-SMAD4 signaling axis [44].

Complement C3 (C3) is part of the complement system has classically been recognized as a central part of the innate immune response. Complement activation has traditionally been considered as part of the body’s immunosurveillance against cancer, but recent reports also suggest that complement elements can promote tumor growth in the context of chronic inflammation [45,46]. Numerous studies of different cancers have suggested that the complement system is activated in response to the expression of tumor-associated antigens, with the subsequent deposition of complement components on tumor tissue and/or elevated levels in body fluids of cancer patients. Elevated C3 levels have been found in colon, pancreas, esophagus, lung, prostate, bladder, ovary, cervix, breast, and neuroblastoma (for detailed references please see Supplementary Table S4). In line with this, several studies have demonstrated a role for activated components of the complement system among which C3 and C5a being the most prominent, in the various stages of carcinogenesis, such as angiogenesis, activation of mitogenic signaling pathways, sustaining of cellular proliferation and insensitivity to apoptosis, and participation in tumor cell invasion and migration (reviewed extensively in [46]).

Granulin (GRN) contributes to multiple human cancers in a way that potentates neoplastic transformation, stimulates tumor growth, metastases, and inhibits anti-apoptotic mechanisms [47]. GRN-A can serve as a prostate cancer serum and tumor marker with clinical value for both diagnosis and prognosis [48].

Another protein firmly implicated in carcinogenesis and tumour progression is Clusterin (CLU) [49]. CLU expression was consistently found to be significantly reduced in both untreated and hormone-refractory human prostate carcinomas, supporting the idea that prostate cell transformation at early stages requires CLU silencing through chromatin remodeling [50].

Phosphatidylethanolamine-binding protein 1 (PEBP1), an inhibitor of several signaling pathways, has been shown to have metastasis suppressor gene activity and promote apoptosis. While first identified in prostate cancer, the loss of PEBP1 expression is observed in many cancers as they progress [51].

S100A9 is a calcium binding protein with multiple ligands and post-translation modifications that is involved in inflammatory events and the initial development of the cancer [52]. S100A9 has been found mainly up-regulated in different cancers, but, opposite levels were also reported (for detailed references please, see Supplementary Table S4). In PCa, both elevated levels in tissue and serum [53] and decreased level in tissues and urine [54] as we have detected, have been observed.

Alpha-enolase (ENO1) in addition to its glycolytic function, is a multifunctional protein that is involved in several biological and pathophysiological processes, depending on its cellular localization: in the cytoplasm it is considered as an oncogene, while in the nucleus, its shorter isoform has been shown to bind to the c-myc promoter and function as a tumor suppressor [55]. Comparative proteomics, as well as genomics and functional studies of several cancers, including PCa, have shown the overexpression of ENO1, but opposite levels, as we have evidenced, have been reported also (Supplementary Table S4). The contradictory findings regarding the ENO1expression levels may be related to the specific mechanisms underlying each cancer type, so future in-depth investigation is needed to elucidate these observations.

Out of the 35 proteins which showed differential abundance in PCa compared to more than one group, only CD14 (Monocyte differentiation antigen CD14) was increased in PCa in comparison to all three groups. It is preferentially expressed on monocytes/macrophages, but also in other non-myeloid cells, such as endothelial, epithelial, smooth muscle, pancreatic islet cells, fibroblasts, and spermatozoa [56]. It appears that CD14 is a multifunctional protein, which other to being a receptor to LPS and other bacterial structures, may regulate T and B lymphocyte activation and also act as acute phase protein [57]. The soluble CD14 in plasma and urine can be generated either by cleavage from the surface of the cell or released from intracellular pools [58] or can be directly secreted by hepatocytes [59]. CD14 has already been found with significantly increased abundance in urine and expressed prostatic secretions in PCa patients, but also in serum of patients with breast, liver, and head and neck cancers (for detailed references please see Supplementary Table S4).

The second group of proteins with differential abundance in PCa consisted of nine proteins (AZU1, IGHG1, RNASE2, PZP, REG1A, AMY1A, AMY2A, ACTG2, COL18A1), which we found associated with different cancers but not with PCa (Supplementary Table S5). The changes in the abundance level of these proteins may represent novel findings, requiring further validation. Three proteins that we found with increased abundance in PCa, namely Non-secretory ribonuclease (RNASE2), Actin gamma-enteric smooth muscle (ACTG2), and Collagen alpha-1(XVIII) chain (COL18A1), may be of special interest for further investigation as these proteins have been found also increased in PCa tissues but are not detected in normal prostate tissue, according to The Human Protein Atlas.

## 5. Conclusions

Differential urine protein abundance in PCa was analyzed in the context of affected cellular functions and pathways to identify signature proteins that are associated with PCa. Acute phase response signaling and LXR/RXR activation pathways were the most prominently affected cellular pathways. Both pathways have been linked to PCa, but their down-regulation, especially regarding the acute phase response signaling, warrants further investigation. In addition to the proteins that are involved in adaptive and innate immune response this study identified a number of oncogenes, tumor suppressors, and multifunctional proteins with highly confirmed involvement in PCa progression, as well as several proteins with no association with PCa so far, which may represent novel findings. Although this study did not discover alter abundance of protein(s) in urine that are exclusively located in prostate, some of the proteins that are identified here could serve as complementary biomarker panel for PCa detection. The validation of the putative biomarker panel constitutes a future perspective and is beyond the scope of this proof of concept study.

## Figures and Tables

**Table 1:** List of proteins with differential abundance in prostate cancer (PCa) as compared to benign prostate hyperplasia (BPH,) bladder cancer (BC), and renal cancer (RC) obtained by two-dimensional Difference Gel Electrophoresis/Mass Spectrometry (2-D DIGE) and identified by MALDI-TOF MS.

Reff. Spot No.	PCa/BPH		PCa/BC		PCa/RC		Protein Name	Gene Name	Mw (kDa) ^b^	pl	Mascot Protein Score	*p* Value	RMS Error (ppm)	Matched Peptides/Total	% of Sequence Coverage
	*t*-Test	Fold Change	*t*-Test	Fold Change	*t*-Test	Fold Change									
1			4.0 × 10^−2^	−3.8			Serotransferrin	TF	79.3	6.81	205	6.4 × 10^−17^	35	20/23	35
2			8.5 × 10^−3^	−3.6			Serotransferrin	TF	79.3	6.81	203	1.0 × 10^−16^	33	21/27	31
3			2.7 × 10^−2^	−3.6			Serotransferrin	TF	79.3	6.81	190	2.0 × 10^−15^	46	23/37	37
4			3.7 × 10^−2^	−3.0			Serotransferrin	TF	79.3	6.81	287	4.0 × 10^−25^	39	29/36	41
5			4.5 × 10^−2^	−3.6			Serotransferrin	TF	79.3	6.81	148	3.2 × 10^−11^	44	17/34	23
6			3.1 × 10^−3^	−6.5			Serotransferrin	TF	79.3	6.81	70	1.8 × 10^−3^	99	8/11	13
7	2.3 × 10^−2^	3.5					Kininogen-1	KNG1	73.0	6.34	94	7.9 × 10^−6^	88	9/13	18
8	4.3 × 10^−2^	2.6	6.2 × 10^−5^	3.0			Kininogen-1	KNG1	73.0	6.34	156	5.1 × 10^−12^	43	17/24	24
9			2.2 × 10^−2^	2.4			Kininogen-1	KNG1	73.0	6.34	139	2.5 × 10^−10^	56	14/20	24
10			3.4 × 10^−2^	2.6			Kininogen-1	KNG1	73.0	6.34	131	1.6 × 10^−9^	101	11/12	18
11	4.5 × 10^−2^	2.5	6.9 × 10^−3^	4.4			Kininogen-1	KNG1	73.0	6.34	111	1.6 × 10^−7^	35	10/14	19
12			5.5 × 10^−3^	3.1			Kininogen-1	KNG1	73.0	6.34	90	5.6 × 10^−6^	58	9/14	18
13	1.3 × 10^−2^	−2.8	3.7 × 10^−2^	−2.6			mix (Ig alpha-1 chain C region/Ig alpha-2 chain C region)	IGHA1/ IGHA2	38.5/ 37.3	6.08/ 5.71	65/81	2.4 × 10^−3^/ 1.1 × 10^−4^	48/45	6/6/8	14/20
14	3.9 × 10^−2^	−2.4	1.9 × 10^−2^	−2.7			mix (Ig alpha-1 chain C region/Ig alpha-2 chain C region)	IGHA1/ IGHA2	38.5/ 37.3	6.08/ 5.71	59/82	2.4 × 10^−2^/ 1.3 × 10^−4^	48/45	5/6/8	13/20
15	2.2 × 10^−2^	−2.9	1.3 × 10^−2^	−2.5			Ig alpha-1 chain C region	IGHA1	38.5	6.08	84	8.8 × 10^−5^	13	7/13	31
16			8.3 × 10^−3^	−2.1			mix (Ig alpha-1 chain C region/Ig alpha-2 chain C region)	IGHA1/ IGHA2	38.5/ 37.3	6.08/ 5.71	111/84	1.6 × 10^−7^/ 7.7 × 10^−5^	59/63	10/8/19	38/27
17	2.1 × 10^−2^	−2.7	3.5 × 10^−2^	−2.5			mix (Ig alpha-1 chain C region/Ig alpha-2 chain C region)	IGHA1/ IGHA2	38.5/ 37.3	6.08/ 5.71	102/84	1.3 × 10^−6^/ 7.7 × 10^−5^	89/93	9/8/19	33/27
18	3.1 × 10^−2^	−2.2	1.2 × 10^−2^	−2.1	5.1 × 10^−3^	−1.6	mix (Ig alpha-1 chain C region/Ig alpha-2 chain C region)	IGHA1	38.5/ 37.3	6.08/ 5.71	92/56	1.2 × 10^−5^/ 5.0 × 10^−2^	23/28	7/6/20	28/26
19	1.4 × 10^−2^	−2.4			5.6 × 10^−2^	−1.6	mix (Ig alpha-1 chain C region/Ig alpha-2 chain C region)	IGHA1/ IGHA2	38.5/ 37.3	6.08/ 5.71	97/69	3.8 × 10^−6^/ 2.4 × 10^−3^	37/37	10/8/38	38/27
20	2.9 × 10^−2^	−2.0	6.4 × 10^−3^	−2.0	6.7 × 10^−2^	−1.7	mix (Ig alpha-1 chain C region/Ig alpha-2 chain C region)	IGHA1/ IGHA2	38.5/ 37.3	6.08/ 5.71	122/82	1.3 × 10^−8^/ 1.4 × 10^−4^	11/12	9/8/12	26/21
21 ^a^			2.0 × 10^−2^	−3.2			Arginyl-tRNA-protein transferase 1	ATE1	60.0	8.17	98	3.6 × 10^−6^	67	9/16	28
22			1.8 × 10^−2^	−2.0			Fibrinogen gamma chain	FGG	52.1	5.37	121	1.6 × 10^−8^	43	12/23	38
23			2.1 × 10^−2^	−5.3			Fibrinogen gamma chain	FGG	52.1	5.37	174	8.0 × 10^−14^	39	14/18	42
24			3.4 × 10^−2^	−5.7			Fibrinogen gamma chain	FGG	52.1	5.37	117	4.0 × 10^−8^	83	12/19	31
25			3.5 × 10^−2^	−7.1			Fibrinogen gamma chain	FGG	52.1	5.37	107	4.0 × 10^−7^	35	12/26	32
26			4.1 × 10^−2^	−5.8			Fibrinogen gamma chain	FGG	52.1	5.37	139	2.5 × 10^−10^	39	13/25	37
27			2.0 × 10^−3^	2.1			Zinc-alpha-2-glycoprotein	AZGP1	34.5	5.71	102	1.3 × 10^−6^	45	13/27	30
28			2.0 × 10^−2^	−4.7			Haptoglobin	HP	45.9	6.13	87	3.8 × 10^−5^	104	8/17	18
29			1.9 × 10^−2^	−6.7			Haptoglobin	HP	45.9	6.13	108	3.2 × 10^−7^	11	13/25	27
30			2.2 × 10^−2^	−6.3			Haptoglobin	HP	45.9	6.13	107	4.0 × 10^−7^	53	16/37	28
31			2.6 × 10^−2^	−7.5			Haptoglobin	HP	45.9	6.13	98	3.1 × 10^−6^	47	12/27	27
32			2.6 × 10^−2^	−8.1			Haptoglobin	HP	45.9	6.13	86	5.0 × 10^−5^	55	13/30	28
33			2.1 × 10^−2^	−7.7			Haptoglobin	HP	45.9	6.13	129	2.5 × 10^−9^	44	12/16	30
34			1.8 × 10^−3^	3.4			Fibrinogen alpha chain (fragment)	FGA	95.7 (45)	5.7	125	6.4 × 10^−9^	72	12/16	15
35			2.6 × 10^−3^	5.3			Inter-alpha-trypsin inhibitor heavy chain H4 (fragment)	ITIH4	103.5 (45)	6.57	69	2.5 × 10^−3^	47	9/26	12
36	2.2 × 10^−2^	2.5					Hemoglobin subunit beta	HBB	16.1	6.75	141	1.6 × 10^−10^	88	11/22	69
37	8.4 × 10^−3^	2.5	5.0 × 10^−4^	4.3			Inter-alpha-trypsin inhibitor heavy chain H4 (fragment)	ITIH4	103.5 (45)	6.57	90	2.1 × 10^−5^	59	13/23	15
38			1.6 × 10^−2^	4.8			Protein AMBP	AMBP	39.9	5.95	94	8.0 × 10^−6^	66	11/30	24
39			1.5 × 10^−2^	5.0			Protein AMBP	AMBP	39.9	5.95	135	6.4 × 10^−10^	37	13/18	32
40			1.4 × 10^−2^	5.2			Protein AMBP	AMBP	39.9	5.95	108	3.2 × 10^−7^	91	10/19	18
41	5.3 × 10^−3^	2.0					Protein AMBP	AMBP	39.9	5.95	74	7.3 × 10^−4^	12	9/17	26
42			3.3 × 10^−3^	4.0			Prostaglandin-H2 D-isomerase	PTGDS	21.2	7.66	64	7.3 × 10^−3^	63	7/15	33
43			6.2 × 10^−3^	4.0			Prostaglandin-H2 D-isomerase	PTGDS	21.2	7.66	70	2.3 × 10^−3^	72	8/25	27
44			8.9 × 10^−3^	3.3			Prostaglandin-H2 D-isomerase	PTGDS	21.2	7.66	60	1.9 × 10^−2^	93	7/26	26
45 ^a^			4.6 × 10^−2^	1.9			Ig kappa chain C region	IGKC	11.8	5.58	83	1.1 × 10^−4^	51	5/10	56
46			4.1 × 10^−2^	−1.9			Serum amyloid P-component	APCS	25.5	6.1	106	5.1 × 10^−7^	31	10/27	34
47 ^a^			7.3 × 10^−4^	6.9			Vitelline membrane outer layer protein 1 homolog	VMO1	22.0	4.9	82	1.3 × 10^−4^	101	7/24	57
48			5.4 × 10^−4^	8.2			CD59 glycoprotein	CD59	14.8	6.02	67	4.1 × 10^−3^	17	4/5	21
49			4.8 × 10^−2^	3.4			Retinol-binding protein 4	RBP4	23.3	5.76	111	1.6 × 10^−7^	76	9/11	41
50			3.4 × 10^−2^	−7.8			Haptoglobin (fragment)	HP	45.9 (20)	6.13	71	2.4 × 10^−3^	50	6/11	12
51			3.8 × 10^−2^	−8.9			Haptoglobin (fragment)	HP	45.9 (20)	6.13	73	1.1 × 10^−3^	16	6/9	12
52			3.2 × 10^−2^	−10.4			Haptoglobin (fragment)	HP	45.9 (20)	6.13	72	1.3 × 10^−3^	49	6/9	14
53	2.7 × 10^−2^	4.5	3.7 × 10^−3^	11.0			Mannan-binding lectin serine protease 2 (fragment)	MASP2	77.2 (19.5)	5.39	125	6.4 × 10^−9^	104	12/16	18
54			1.7 × 10^−2^	4.5			Chain A, Laminin G Like Domain 3 From Human Perlecan	HSPG2 (gi|361131590)	20.7	5.47	181	2.5 × 10^−13^	55	11/15	73

^a^ Significant number of peptide peaks in peptide mass fingerprinting of the protein spot not matching to the identified protein, indicating possible existence of more than one protein; ^b^ The value in the brackets is the estimated Mw of the protein fragment from the gel.

**Table 2:** List of identified proteins with differential abundance in PCa when compared to the three groups (BPH, BC, and RC) by LC-MS/MS.

Uniprot Accession No.	Protein Name	Gene Name	Peptide Count	Unique Peptides	Confidence Score	ANOVA	Max Fold Change	Highest Mean Condition	Lowest Mean Condition	Group Comparison	*p*-Value ^a^	Ratio
P08571	Monocyte differentiation antigen	CD14	8	7	75.64	8.87 × 10^−10^	2.70	PCa	Bc	PCa vs. BC	2.71 × 10^−4^	2.7
	CD14									PCa vs. BPH	4.67 × 10^−3^	1.8
										PCa vs. RC	2.61 × 10^−2^	1.6
P62736	Actin_ aortic smooth muscle	ACTA2	3	3	24.21	1.75 × 10^−9^	5.89	Bc	PCa	PCa vs. BC	3.40 × 10^−4^	0.2
										PCa vs. BPH	1.75 × 10^−2^	0.4
P02765	Alpha-2-HS-glycoprotein	AHSG	9	9	94.63	5.73 × 10^−4^	2.70	PCa	Bc	PCa vs. BC	9.03 × 10^−3^	2.7
										PCa vs. BPH	4.14 × 10^−2^	2.1
P04745	Alpha-amylase 1	AMY1A	9	5	101.49	1.42 × 10^−7^	3.46	PCa	Bc	PCa vs. BC	3.40 × 10^−4^	3.5
										PCa vs. BPH	4.14 × 10^−2^	1.7
P19961	Alpha-amylase 2B	AMY2B	3	1	21.24	3.52 × 10^−6^	12.86	Rc	Bc	PCa vs. BC	1.25 × 10^−2^	11.9
										PCa vs. BPH	4.32 × 10^−2^	4.1
P06733	Alpha-enolase	ENO1	2	2	14.73	3.64 × 10^−5^	1.78	BPH	PCa	PCa vs. BC	1.54 × 10^−2^	0.7
										PCa vs. BPH	7.97 × 10^−3^	0.6
P04083	Annexin A1	ANXA1	5	5	41.02	5.95 × 10^−12^	5.45	Rc	Bc	PCa vs. BC	1.08 × 10^−3^	3.8
										PCa vs. BPH	1.20 × 10^−2^	2.2
P20160	Azurocidin	AZU1	2	2	13.76	7.34 × 10^−5^	2.80	BPH	PCa	PCa vs. BC	3.35 × 10^−2^	0.6
										PCa vs. BPH	8.89 × 10^−3^	0.4
P10909	Clusterin	CLU	10	10	99.24	6.33 × 10^−6^	2.26	BPH	PCa	PCa vs. BC	5.24 × 10^−3^	0.6
										PCa vs. BPH	2.55 × 10^−2^	0.4
P12109	Collagen alpha-1(VI)	COL6A1	6	4	46.91	2.05 × 10^−7^	3.44	BPH	PCa	PCa vs. BC	7.15 × 10^−4^	0.4
	chain									PCa vs. BPH	8.66 × 10^−3^	0.3
P39060	Collagen alpha-1(XVIII)	COL18A1	2	2	14.39	2.92 × 10^−9^	14.26	Rc	Bc	PCa vs. BC	6.21 × 10^−3^	7.1
	chain									PCa vs. BPH	2.30 × 10^−2^	4.2
P01024	Complement C3	C3	57	57	545.51	2.30 × 10^−7^	3.43	Rc	Bc	PCa vs. BC	6.30 × 10^−3^	2.9
										PCa vs. BPH	2.77 × 10^−2^	2.2
P24855	Deoxyribonuclease-1	DNASE1	3	3	23.62	1.84 × 10^−10^	4.85	Rc	Bc	PCa vs. BC	6.30 × 10^−3^	3.6
										PCa vs. BPH	3.55 × 10^−2^	2.3
O94919	Endonuclease domain-containing 1	ENDOD1	3	3	24.51	1.64 × 10^−7^	2.97	PCa	Bc	PCa vs. BC	1.01 × 10^−3^	3.0
	protein									PCa vs. BPH	4.67 × 10^−3^	2.8
P02671	Fibrinogen alpha chain	FGA	24	24	293.54	7.67 × 10^−6^	2.72	BPH	Rc	PCa vs. BC	2.94 × 10^−3^	0.6
										PCa vs. BPH	3.15 × 10^−2^	0.4
P02679	Fibrinogen gamma chain	FGG	20	20	251.65	1.07 × 10^−4^	2.32	BPH	Rc	PCa vs. BC	2.48 × 10^−2^	0.5
										PCa vs. BPH	4.25 × 10^−2^	0.5
P28799	Granulins	GRN	2	2	18.13	2.87 × 10^−8^	3.92	BPH	PCa	PCa vs. BC	2.32 × 10^−3^	0.5
										PCa vs. BPH	8.89 × 10^−3^	0.3
P68871	Hemoglobin subunit beta	HBB	10	10	138.23	1.54 × 10^−5^	4.62	Bc	PCa	PCa vs. BC	2.96 × 10^−2^	0.2
										PCa vs. BPH	1.75 × 10^−2^	0.3
P02790	Hemopexin	HPX	16	16	164.63	4.66 × 10^−6^	2.44	BPH	PCa	PCa vs. BC	6.60 × 10^−3^	0.6
										PCa vs. BPH	1.30 × 10^−2^	0.4
P01857	Ig gamma-1 chain C region	IGHG1	6	6	76.08	6.40 × 10^−4^	3.29	BPH	PCa	PCa vs. BC	6.71 × 10^−3^	0.5
										PCa vs. BPH	4.14 × 10^−2^	0.3
P05451	Lithostathine-1-alpha	REG1A	5	4	35.95	3.83 × 10^−4^	3.96	PCa	BPH	PCa vs. BC	6.30 × 10^−3^	3.8
										PCa vs. BPH	1.50 × 10^−2^	4.0
P11117	Lysosomal acid	ACP2	5	4	45.84	5.59 × 10^−6^	2.11	BPH	PCa	PCa vs. BC	7.15 × 10^−4^	0.6
	phosphatase									PCa vs. BPH	6.07 × 10^−3^	0.5
P08246	Neutrophil elastase	ELANE	3	3	39.04	6.45 × 10^−3^	3.25	BPH	PCa	PCa vs. BC	2.74 × 10^−2^	0.5
										PCa vs. BPH	4.99 × 10^−2^	0.3
P10153	Non-secretory	RNASE2	4	3	51.80	3.38 × 10^−8^	5.21	PCa	Bc	PCa vs. BC	8.08 × 10^−3^	5.2
	ribonuclease									PCa vs. BPH	2.55 × 10^−2^	3.9
P10451	Osteopontin	SPP1	12	11	164.02	1.10 × 10^−11^	15.72	Rc	Bc	PCa vs. BC	2.32 × 10^−3^	8.4
										PCa vs. BPH	2.55 × 10^−2^	2.6
P04746	Pancreatic alpha-amylase	AMY2A	3	1	27.44	3.66 × 10^−6^	5.06	PCa	Bc	PCa vs. BC	4.27 × 10^−3^	5.1
										PCa vs. BPH	4.32 × 10^−2^	2.3
P30086	Phosphatidylethanolamine-	PEBP1	3	3	27.16	2.93 × 10^−5^	3.38	BPH	PCa	PCa vs. BC	7.15 × 10^−4^	0.5
	binding protein 1									PCa vs. BPH	1.75 × 10^−2^	0.3
P20742	Pregnancy zone protein	PZP	7	7	82.62	4.09 × 10^−8^	2.27	Bc	Rc	PCa vs. BC	2.32 × 10^−3^	0.5
										PCa vs. BPH	4.76 × 10^−2^	0.7
P41222	Prostaglandin-H2	PTGDS	9	7	140.59	4.51 × 10^−11^	5.56	PCa	Bc	PCa vs. BC	3.40 × 10^−4^	5.6
	D-isomerase									PCa vs. BPH	1.75 × 10^−2^	1.8
P06702	Protein S100-A9	S100A9	3	3	26.61	1.49 × 10^−6^	6.46	BPH	PCa	PCa vs. BC	1.22 × 10^−3^	0.5
										PCa vs. BPH	5.03 × 10^−2^	0.2
Q8WZ75	Roundabout homolog 4	ROBO4	7	7	55.71	3.32 × 10^−3^	2.27	BPH	PCa	PCa vs. BC	1.47 × 10^−2^	0.6
										PCa vs. BPH	2.96 × 10^−2^	0.4
Q12907	Vesicular integral-membrane	LMAN2	9	8	94.66	1.64 × 10^−6^	2.48	Rc	Bc	PCa vs. BC	2.32 × 10^−3^	2.4
	protein VIP36									PCa vs. BPH	2.55 × 10^−2^	1.7
Q14624	Inter-alpha-trypsin inhibitor heavy	ITIH4	9	9	94.01	3.21 × 10^−13^	5.55	Rc	Bc	PCa vs. BC	5.07 × 10^−5^	3.5
	chain H4									PCa vs. BPH	4.67 × 10^−3^	1.6
P63267	Actin_ gamma-enteric	ACTG2	3	3	19.63	4.29 × 10^−2^	1.79	PCa	Rc	PCa vs. BC	1.08 × 10^−3^	1.7
	smooth muscle									PCa vs. RC	2.56 × 10^−3^	1.8
P07858	Cathepsin B	CTSB	3	2	19.04	5.87 × 10^−3^	3.16	Bc	PCa	PCa vs. BC	3.10 × 10^−2^	0.3
										PCa vs. RC	3.14 × 10^−2^	0.4
P01023	Alpha-2-macroglobulin	A2M	38	38	491.82	1.42 × 10^−4^	2.29	BPH	PCa	PCa vs. BPH	6.07 × 10^−3^	0.4
P15144	Aminopeptidase N	ANPEP	2	2	14.55	3.31 × 10^−4^	1.70	BPH	PCa	PCa vs. BPH	4.20 × 10^−2^	0.6
P07355	Annexin A2	ANXA2	2	2	16.06	9.99 × 10^−5^	4.27	Rc	BPH	PCa vs. BPH	4.76 × 10^−2^	4.1
P06727	Apolipoprotein A-IV	APOA4	3	3	31.32	3.77 × 10^−3^	2.28	BPH	PCa	PCa vs. BPH	5.03 × 10^−2^	0.4
Q8NFZ8	Cell adhesion molecule 4	CADM4	8	7	60.30	1.09 × 10^−3^	2.26	BPH	PCa	PCa vs. BPH	3.15 × 10^−2^	0.4
P17900	Ganglioside GM2 activator	GM2A	4	3	40.41	4.80 × 10^−2^	1.81	BPH	PCa	PCa vs. BPH	4.97 × 10^−2^	0.6
P69905	Hemoglobin subunit alpha	HBA1	5	5	56.91	9.41 × 10^−3^	4.36	Bc	PCa	PCa vs. BPH	4.67 × 10^−3^	0.6
P02042	Hemoglobin subunit delta	HBD	3	3	29.39	1.38 × 10^−5^	13.11	Bc	Rc	PCa vs. BPH	4.20 × 10^−2^	0.5
P01859	Ig gamma-2 chain C region	IGHG2	7	7	67.03	6.75 × 10^−5^	2.18	BPH	Bc	PCa vs. BPH	6.07 × 10^−3^	0.5
P01861	Ig gamma-4 chain C region	IGHG4	6	6	72.40	9.74 × 10^−4^	2.05	BPH	Rc	PCa vs. BPH	4.32 × 10^−2^	0.6
P01772	Ig heavy chain V-III region KOL	IGHV3-33	2	1	12.43	4.33 × 10^−3^	1.57	BPH	PCa	PCa vs. BPH	4.89 × 10^−2^	0.6
P0CF74	Ig lambda-6 chain C region	IGLC6	3	3	28.38	7.00 × 10^−4^	1.97	BPH	Bc	PCa vs. BPH	2.30 × 10^−2^	0.5
P02788	Lactotransferrin	LTF	43	41	601.64	8.73 × 10^−5^	2.61	BPH	Rc	PCa vs. BPH	4.20 × 10^−2^	0.5
P10253	Lysosomal alpha-glucosidase	GAA	8	8	80.33	9.63 × 10^−5^	1.77	BPH	PCa	PCa vs. BPH	4.14 × 10^−2^	0.6
P05164	Myeloperoxidase	MPO	16	15	159.24	4.69 × 10^−5^	1.76	BPH	PCa	PCa vs. BPH	4.67 × 10^−3^	0.6
P32119	Peroxiredoxin-2	PRDX2	3	1	23.32	2.58 × 10^−2^	13.40	Bc	PCa	PCa vs. BPH	5.03 × 10^−2^	0.2
P05155	Plasma protease C1 inhibitor	SERPING1	4	4	35.56	4.75 × 10^−3^	1.71	BPH	PCa	PCa vs. BPH	4.99 × 10^−2^	0.6
Q6S8J3	POTE ankyrin domain family member E	POTEE	11	8	93.51	3.30 × 10^−4^	2.04	BPH	Rc	PCa vs. BPH	4.76 × 10^−2^	0.5
P43652	Afamin	AFM	6	5	48.31	8.56 × 10^−4^	1.65	PCa	Bc	PCa vs. BC	5.76 × 10^−3^	1.6
P02647	Apolipoprotein A-I	APOA1	12	12	145.00	4.72 × 10^−4^	1.86	BPH	Rc	PCa vs. BC	3.02 × 10^−2^	0.7
P05090	Apolipoprotein D	APOD	6	6	69.92	4.00 × 10^−7^	2.12	PCa	Bc	PCa vs. BC	1.19 × 10^−3^	2.1
P02749	Beta-2-glycoprotein 1	APOH	11	10	112.68	1.13 × 10^−4^	3.66	Bc	Rc	PCa vs. BC	7.15 × 10^−4^	0.3
P19835	Bile salt-activated lipase	CEL	3	3	20.03	1.76 × 10^−6^	2.04	Rc	Bc	PCa vs. BC	7.58 × 10^−3^	1.6
P07339	Cathepsin D	CTSD	6	6	54.69	1.61 × 10^−3^	1.85	PCa	Bc	PCa vs. BC	1.38 × 10^−2^	1.9
P13987	CD59 glycoprotein	CD59	5	5	85.01	1.42 × 10^−6^	4.38	Rc	Bc	PCa vs. BC	2.94 × 10^−3^	4.3
P0C0L4	Complement C4-A	C4A	3	3	21.19	1.16 × 10^−2^	1.85	Bc	PCa	PCa vs. BC	6.21 × 10^−3^	0.5
P08174	Complement decay-accelerating factor	CD55	5	5	40.67	2.66 × 10^−5^	2.37	Rc	Bc	PCa vs. BC	1.02 × 10^−2^	2.2
Q02487	Desmocollin-2	DSC2	4	3	24.44	4.00 × 10^−6^	2.62	Bc	BPH	PCa vs. BC	1.78 × 10^−3^	0.6
Q9UHL4	Dipeptidyl peptidase 2	DPP7	2	2	12.10	4.08 × 10^−2^	4.13	BPH	PCa	PCa vs. BC	2.84 × 10^−2^	0.6
Q9HCU0	Endosialin	CD248	6	6	43.09	2.31 × 10^−6^	2.20	PCa	Bc	PCa vs. BC	1.14 × 10^−2^	2.2
P01834	Ig kappa chain C region	IGKC	9	9	154.85	3.59 × 10^−3^	2.04	PCa	Bc	PCa vs. BC	1.11 × 10^−3^	2.0
P01617	Ig kappa chain V-II region TEW	IGKV2D-28	5	3	60.57	1.37 × 10^−2^	2.22	Rc	Bc	PCa vs. BC	7.15 × 10^−4^	1.6
P01625	Ig kappa chain V-IV region Len	IGKV4-1	2	1	17.70	1.49 × 10^−3^	1.62	Rc	Bc	PCa vs. BC	7.58 × 10^−3^	1.6
P80748	Ig lambda chain V-III region LOI	IGLV3-21	3	3	29.50	1.01 × 10^−3^	2.13	PCa	Bc	PCa vs. BC	1.00 × 10^−4^	2.1
P0CG04	Ig lambda-1 chain C regions	IGLC1	6	6	112.58	6.98 × 10^−3^	2.14	BPH	Bc	PCa vs. BC	2.40 × 10^−3^	1.8
P0CG05	Ig lambda-2 chain C regions	IGLC2	3	3	57.93	1.94 × 10^−2^	1.73	BPH	Bc	PCa vs. BC	3.90 × 10^−3^	1.7
P01042	Kininogen-1	KNG1	27	27	362.50	8.88 × 10^−7^	2.71	Rc	Bc	PCa vs. BC	8.22 × 10^−3^	1.7
P14543	Nidogen-1	NID1	9	8	84.01	5.13 × 10^−5^	3.95	BPH	Bc	PCa vs. BC	2.23 × 10^−3^	2.0
O75594	Peptidoglycan recognition protein 1	PGLYRP1	5	5	50.82	6.11 × 10^−5^	2.30	BPH	Bc	PCa vs. BC	3.28 × 10^−3^	1.9
P01833	Polymeric immunoglobulin receptor	PIGR	25	24	263.55	5.20 × 10^−5^	2.50	Rc	Bc	PCa vs. BC	6.21 × 10^−3^	1.6
P02760	Protein AMBP	AMBP	25	24	366.65	4.27 × 10^−3^	2.49	Rc	Bc	PCa vs. BC	1.02 × 10^−2^	1.9
P00734	Prothrombin	F2	16	14	176.60	3.60 × 10^−5^	5.38	Rc	Bc	PCa vs. BC	7.15 × 10^−4^	2.7
P07998	Ribonuclease pancreatic	RNASE1	4	4	39.91	7.99 × 10^−15^	4.42	Rc	Bc	PCa vs. BC	7.63 × 10^−4^	3.5
Q9UBC9	Small proline-rich protein 3	SPRR3	2	2	22.69	7.14 × 10^−3^	2.10	PCa	Bc	PCa vs. BC	1.25 × 10^−2^	2.1
Q9UGT4	Sushi domain-containing protein 2	SUSD2	2	2	13.45	5.40 × 10^−5^	32.32	PCa	Bc	PCa vs. BC	4.99 × 10^−2^	32.3
P07911	Uromodulin	UMOD	27	27	399.14	6.23 × 10^−6^	2.98	Rc	Bc	PCa vs. BC	4.00 × 10^−2^	1.7
P22891	Vitamin K-dependent protein Z	PROZ	4	3	42.25	1.29 × 10^−2^	2.21	PCa	Bc	PCa vs. BC	2.92 × 10^−2^	2.2
P25311	Zinc-alpha-2-glycoprotein	AZGP1	17	15	230.16	1.61 × 10^−2^	2.47	PCa	Bc	PCa vs. BC	1.25 × 10^−2^	2.5
P06870	Kallikrein-1	KLK1	6	6	64.59	4.90 × 10^−4^	2.36	BPH	Bc	PCa vs. RC	2.61 × 10^−2^	0.6
Q96PD5	*N*-acetylmuramoyl-L-alanine amidase	PGLYRP2	5	5	56.28	7.41 × 10^−3^	2.14	PCa	Rc	PCa vs. RC	2.56 × 10^−3^	2.1

^a^ Corrected *p*-value for multiple testing using Benjamini and Hochberg False Discovery Rate.

## Supplementary Materials

The following are available online www.mdpi.com/2227-7382/6/1/1/s1: Supplementary document 1: Representative 2-D map of the urine proteome obtained by 2-D gel electrophoresis and the annotated MS spectra of spots representing the identified proteins with differential abundance, Supplementary document 2: Identification and quantification data of the 35 putative biomarkers for PCa in this study, Supplementary Table S1: Age, gender and clinical information of patients included in the study together with their PSA levels, histology grading and tumor stage, Supplementary Table S2: List of identified proteins with LC-MS/MS with quantification values, Supplementary Table S3: List of all identified peptides with LC-MS/MS in this study, Supplementary Table S4: Functional characterization and association with prostate and other types of malignancy of 20 proteins with differential abundance in PCa identified in this study in more than one group comparison, Supplementary Table S5: Functional characterization and association with malignancy of 9 proteins with differential abundance in PCa identified in this study in more than one group comparison.
