# Supplementary material for: Comparative Proteomics Analysis of Urine Reveals Down-Regulation of Acute Phase Response Signaling and LXR/RXR Activation Pathways in Prostate Cancer
Source: Proteomes. 2017 Dec 29;6(1):1. doi: 10.3390/proteomes6010001 (PMC5874760; doi:10.3390/proteomes6010001)
Supplement: Supplementary file 1 [file proteomes-06-00001-s001.zip › proteomes-247986 supplementary 12.27/Supplementary document 1.pdf]

Supplementary document 1

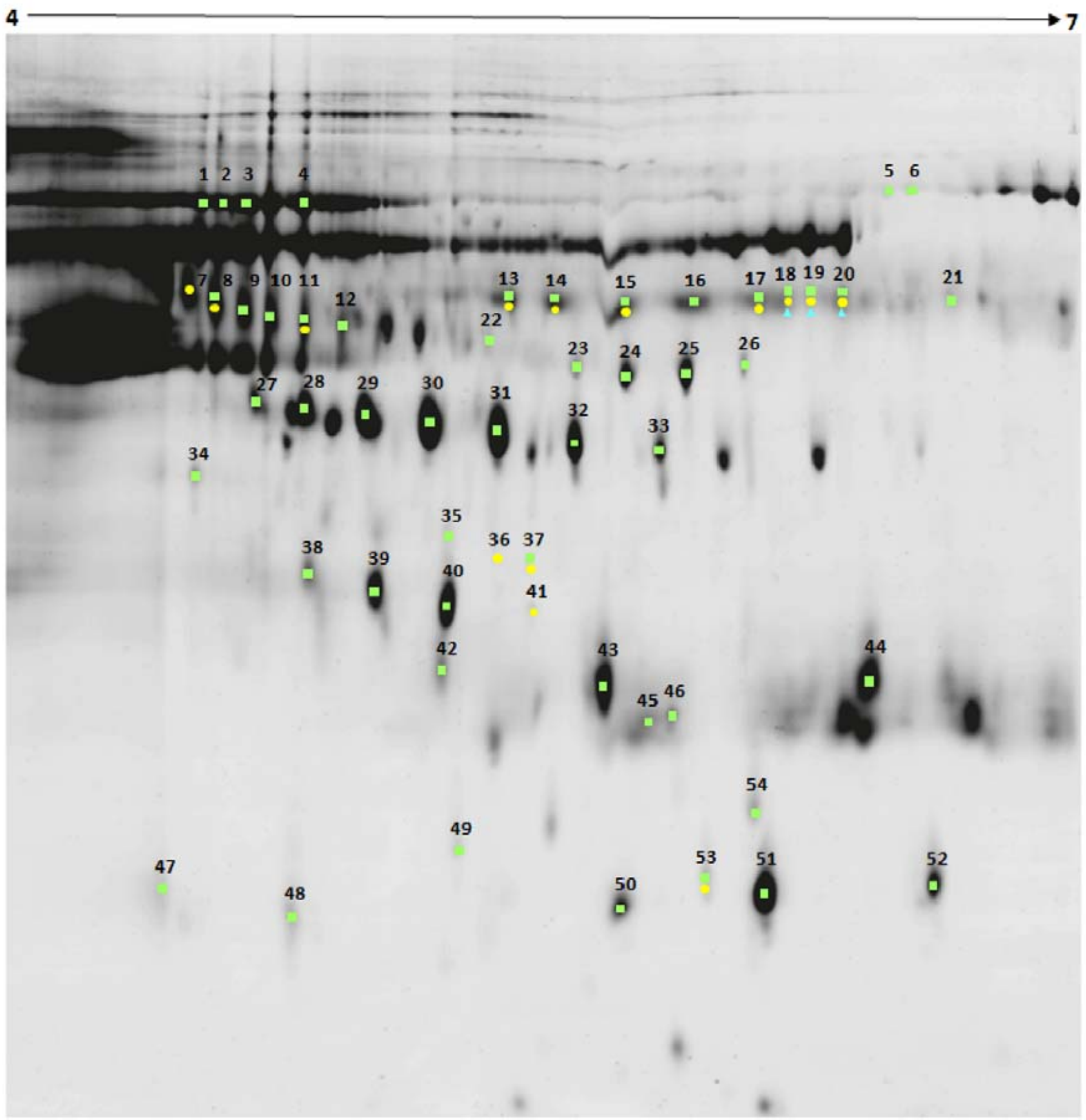

Representative 2-D map of the urine proteome obtained by using IEF on pH 4–7 IPG strip and 2-D gel electrophoresis on 12.5% SDS-PAGE. Protein with differential abundance between studied groups are marked as follows: (●) PCa/BPH, (■) PCa/BC and (▲) PCa/RC. Details of spots identification by peptide mass fingerprinting using MALDI-TOF MS are given in Table 1.

The annotated MS spectra of selected spots representing each of the identified proteins with differential abundance in this study. The peaks submitted for Mascot search are annotated with orange lines and m/z values. The peaks specific for the identified protein are marked with green dots. Spectra annotated with orange star have significant number of peptide peaks in peptide mass fingerprinting not matching to the identified protein, indicating possible existence of more than one protein. The remaining (not annotated) peaks belong to matrix, trypsin autolysis products and other contaminants.

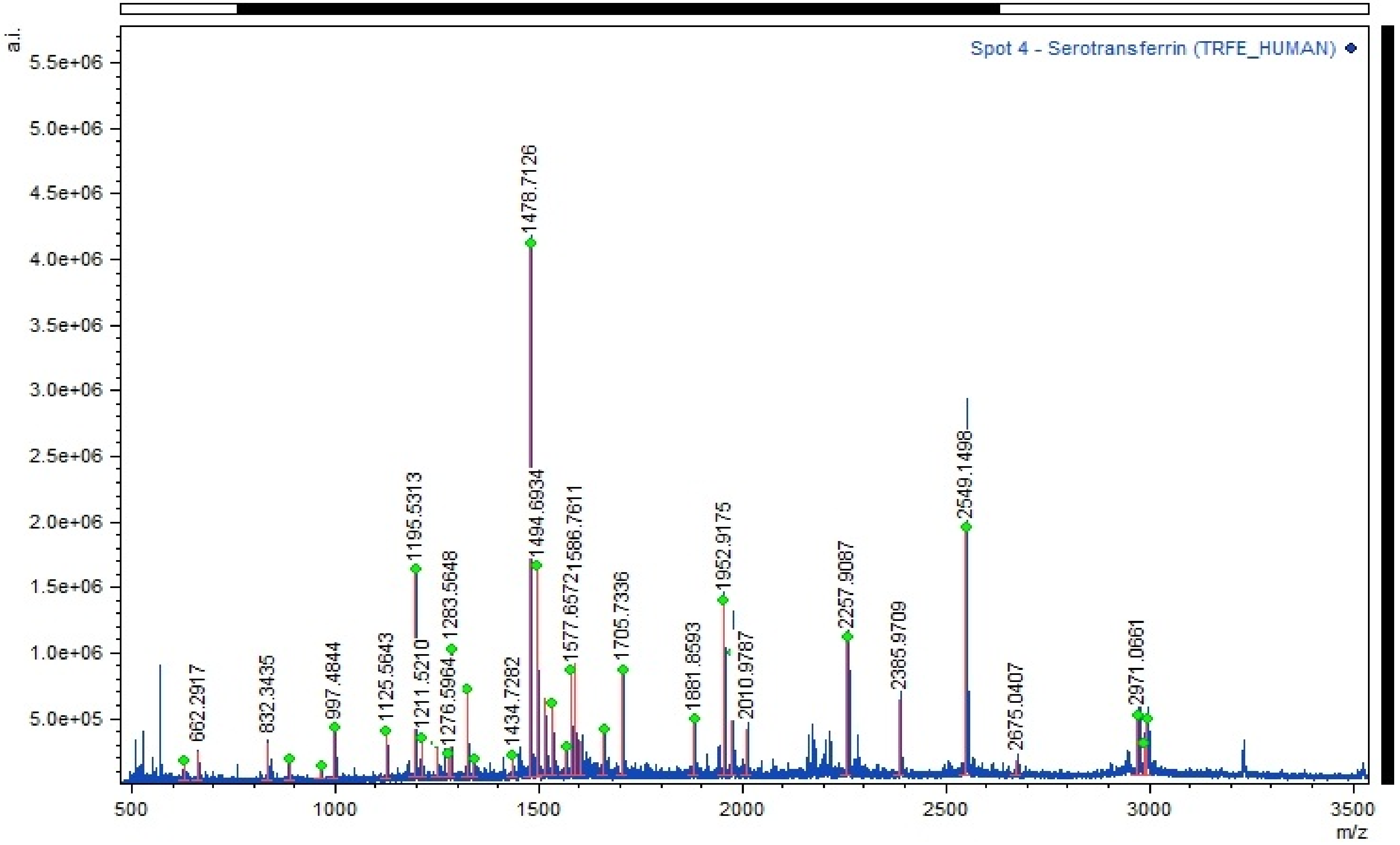

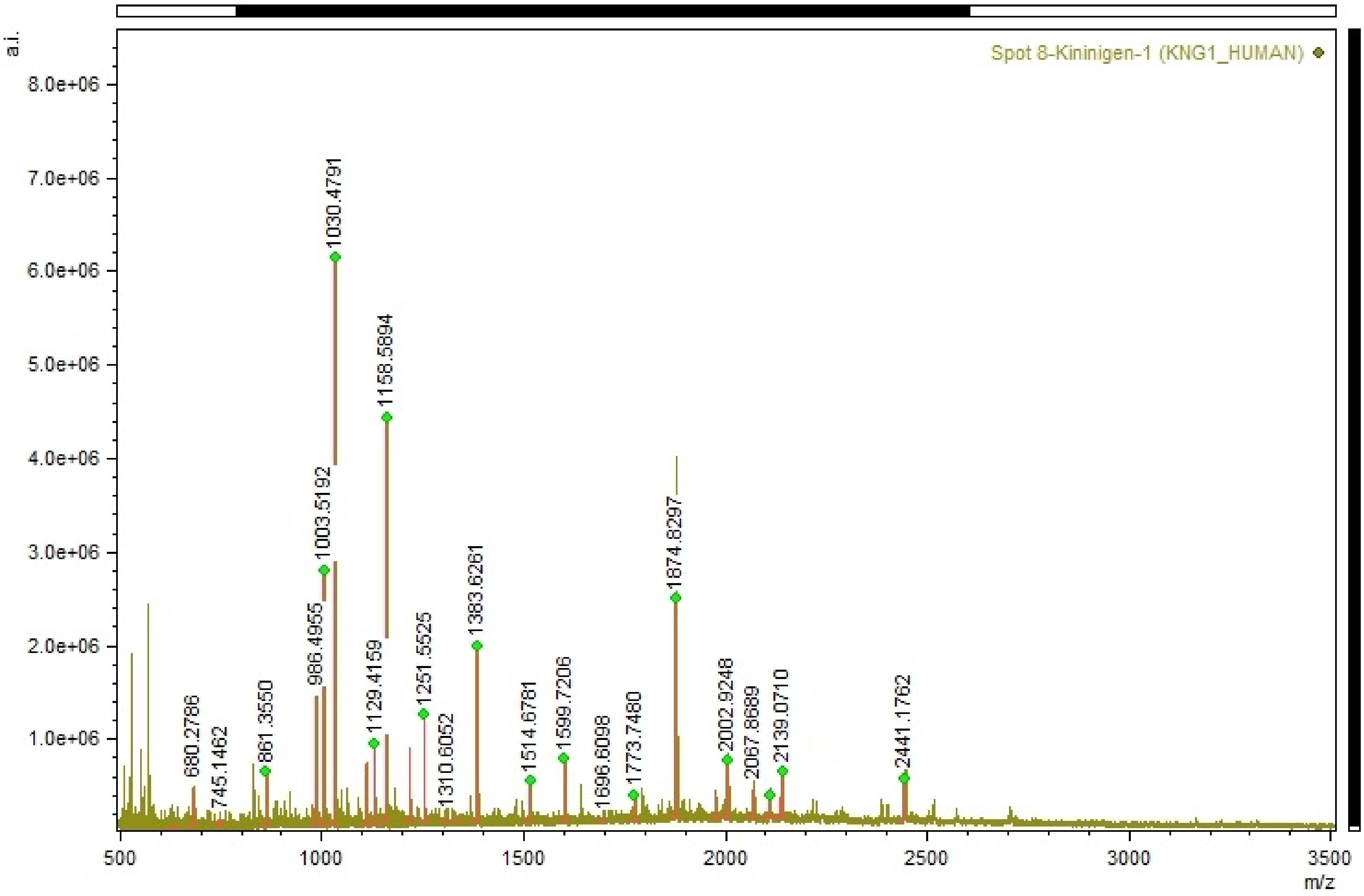

Spot 8-Kininigen-1 (KNG1_HUMAN)
a.i.
8.0e+06
7.0e+06
6.0e+06
5.0e+06
4.0e+06
3.0e+06
2.0e+06
1.0e+06
1030.4791
1158.5894
1003.5192
986.4955
1874.8297
1383.6261
1129.4159
1251.5525
1310.6052
680.2786
745.1462
861.3550
1514.6781
1599.7206
1696.6098
1773.7480
2002.9248
2067.8689
2139.0710
2441.1762
500
1000
1500
2000
2500
3000
3500
m/z

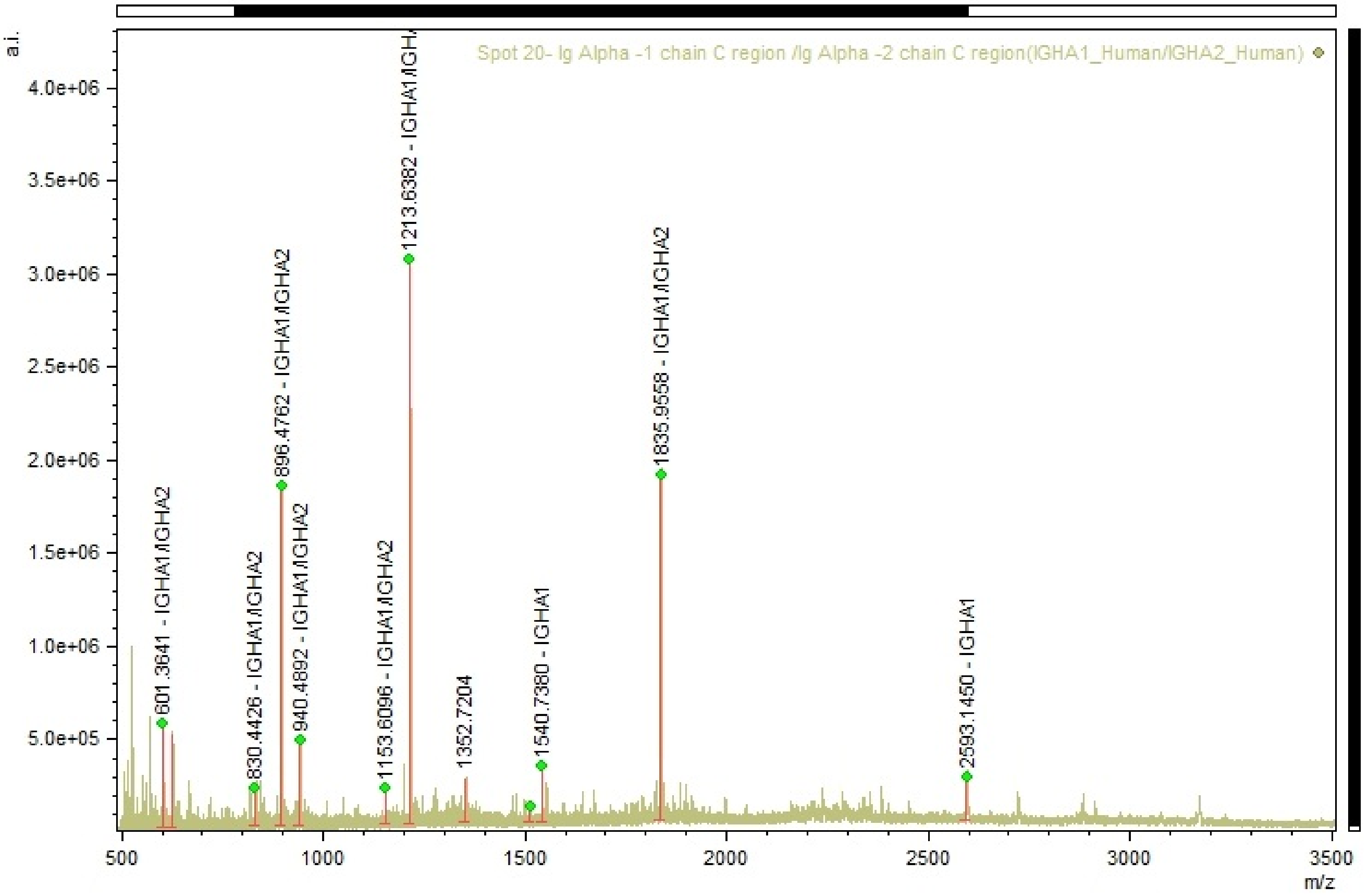

Spot 20- Ig Alpha -1 chain C region /Ig Alpha -2 chain C region(IGHA1_Human/IGHA2_Human)
a.i.
4.0e+06
3.5e+06
3.0e+06
2.5e+06
2.0e+06
1.5e+06
1.0e+06
5.0e+05
601.3641 - IGHA1/IGHA2
830.4426 - IGHA1/IGHA2
896.4762 - IGHA1/IGHA2
940.4892 - IGHA1/IGHA2
1153.6096 - IGHA1/IGHA2
1213.6382 - IGHA1/IGH
1352.7204
1540.7380 - IGHA1
1835.9558 - IGHA1/IGHA2
2593.1450 - IGHA1
500
1000
1500
2000
2500
3000
3500
m/z

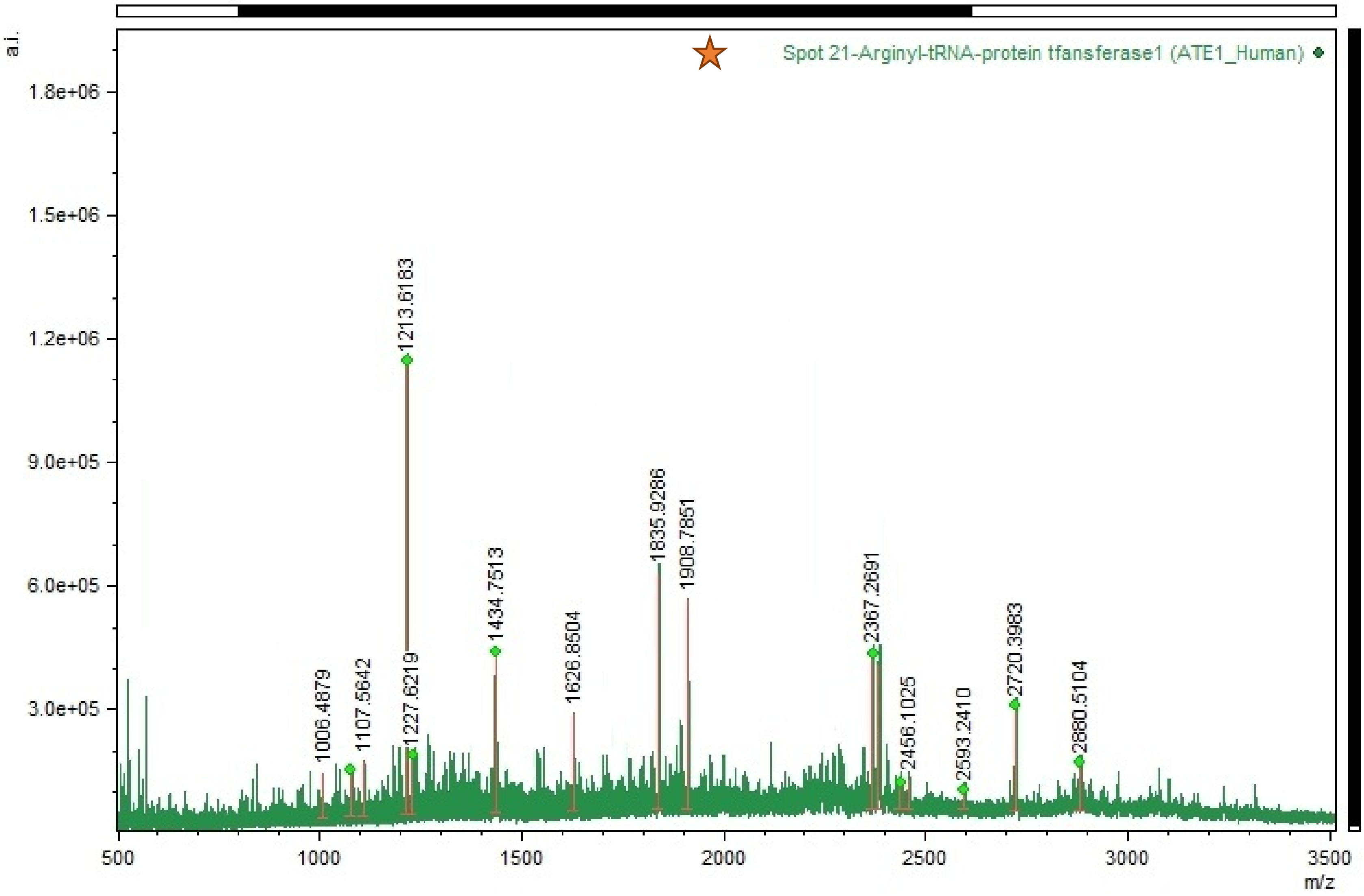

Spot 21-Arginyl-tRNA-protein tfansferase1 (ATE1_Human)
a.i.
1.8e+06
1.5e+06
1.2e+06
9.0e+05
6.0e+05
3.0e+05
1213.6183
1835.9286
1908.7851
1434.7513
1626.8504
2367.2691
2720.3983
1006.4879
1107.5642
1227.6219
2456.1025
2593.2410
2880.5104
500
1000
1500
2000
2500
3000
3500
m/z

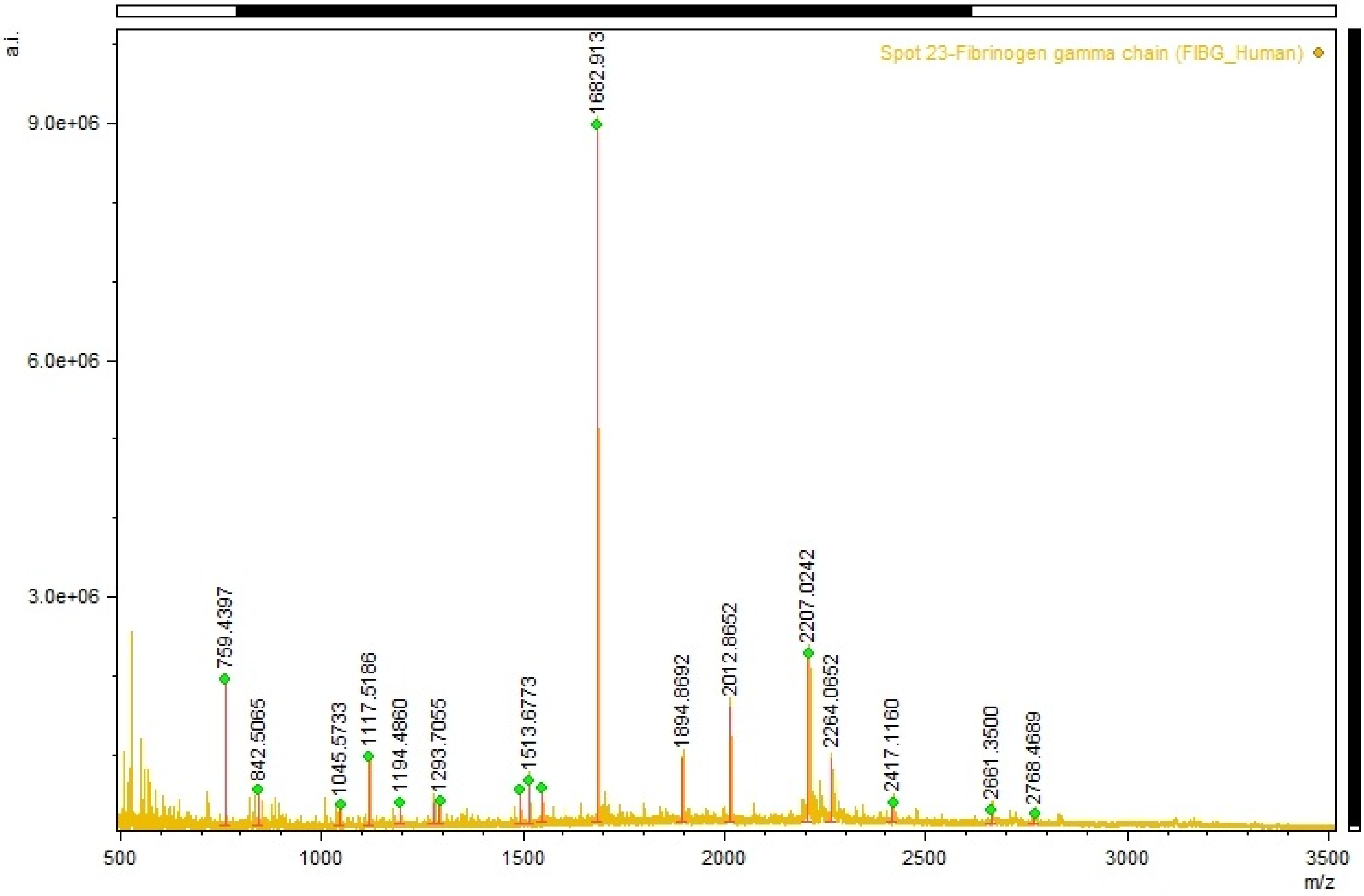
Spot 23-Fibrinogen gamma chain (FIBG_Human)
a.i.
9.0e+06
6.0e+06
3.0e+06
1682.913
759.4397
842.5065
1045.5733
1117.5186
1194.4860
1293.7055
1513.6773
1894.8692
2012.8652
2207.0242
2264.0652
2417.1160
2661.3500
2768.4689
500
1000
1500
2000
2500
3000
3500
m/z

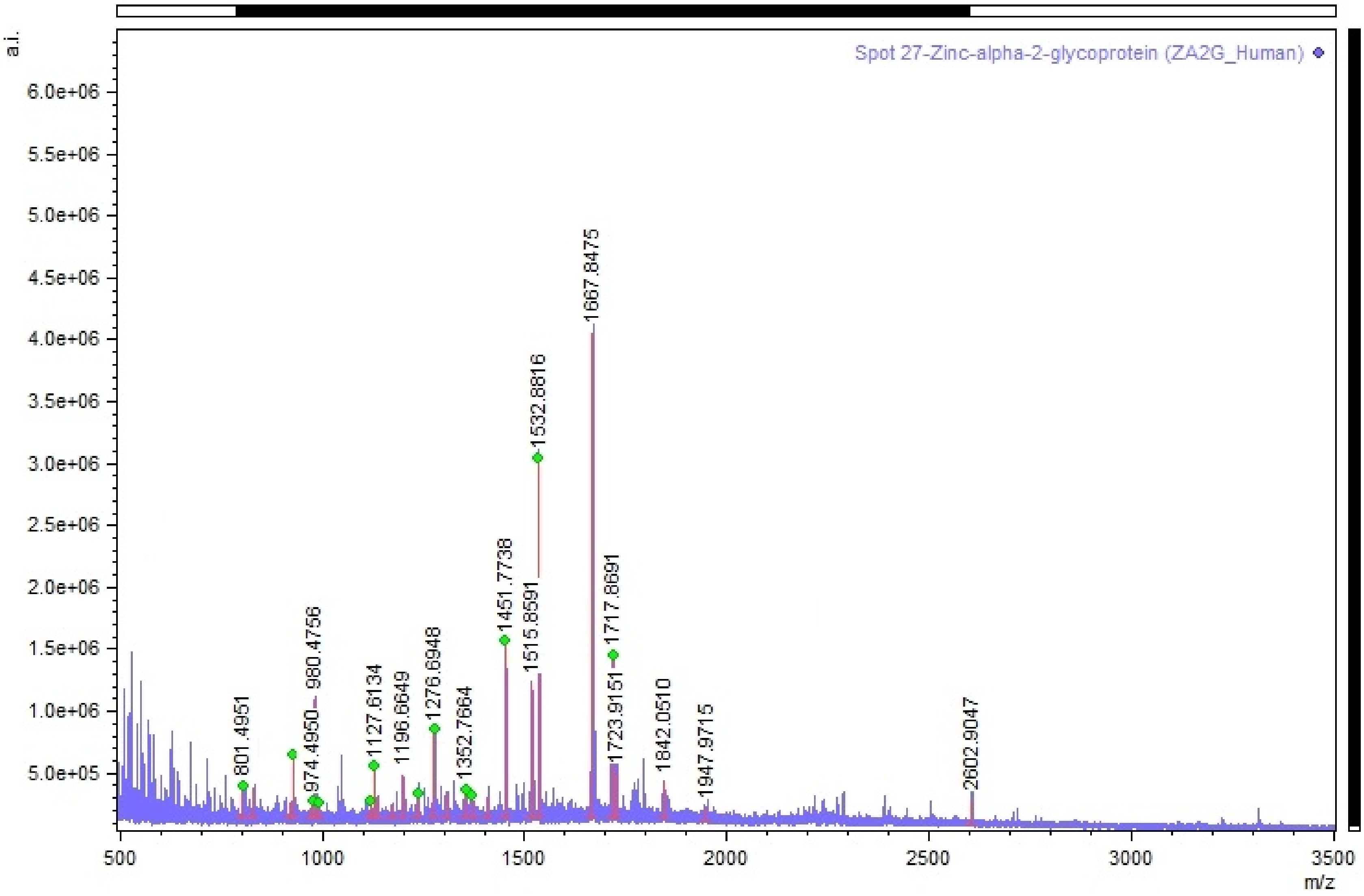

Spot 27-Zinc-alpha-2-glycoprotein (ZA2G_Human)
a.i.
6.0e+06
5.5e+06
5.0e+06
4.5e+06
4.0e+06
3.5e+06
3.0e+06
2.5e+06
2.0e+06
1.5e+06
1.0e+06
5.0e+05
801.4951
974.4950
980.4756
1127.6134
1196.6649
1276.6948
1352.7664
1451.7738
1515.8591
1532.8816
1667.8475
1717.8691
1723.9151
1842.0510
1947.9715
2602.9047
500
1000
1500
2000
2500
3000
3500
m/z

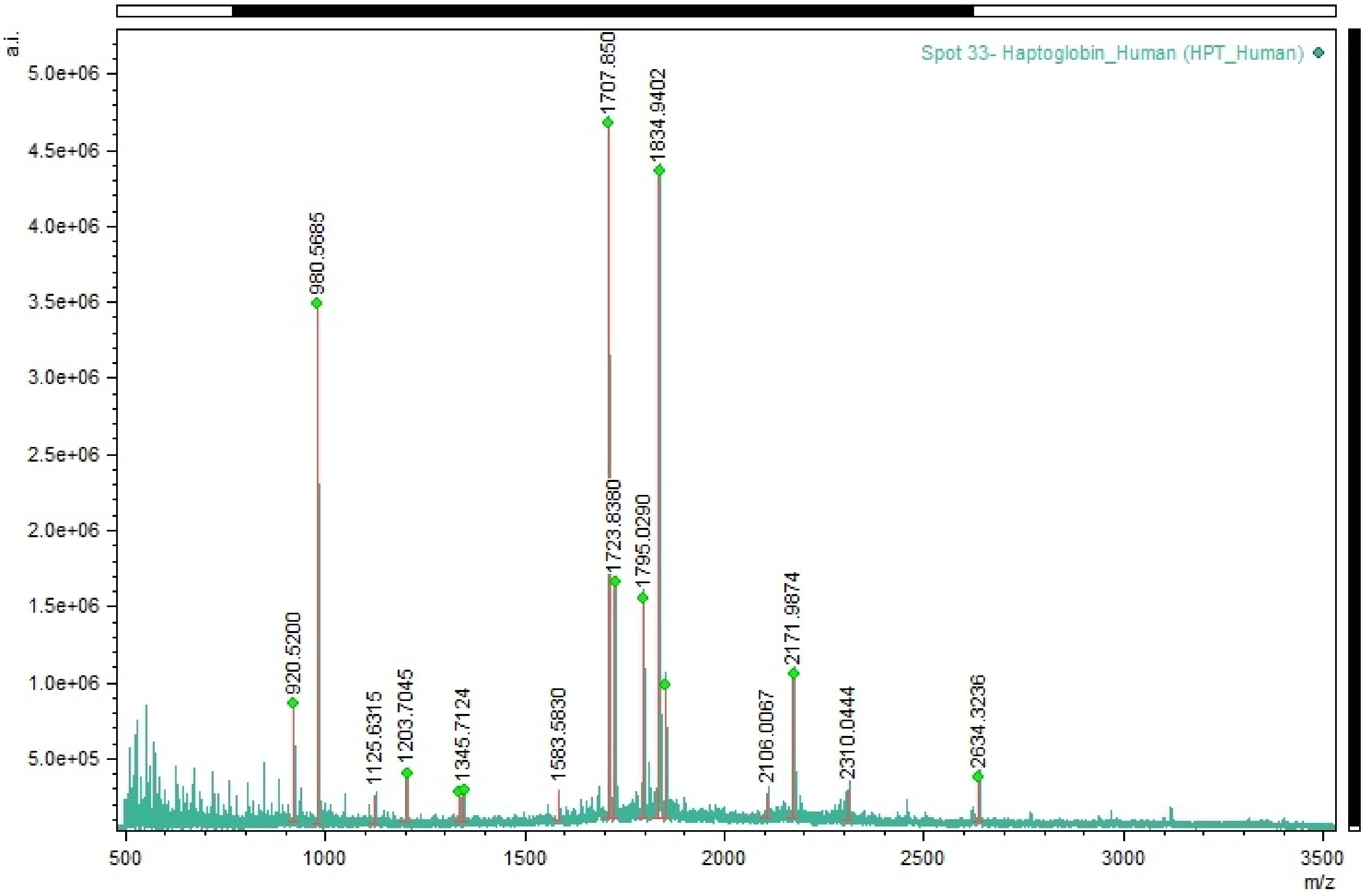

Spot 33- Haptoglobin_Human (HPT_Human)
a.i.
5.0e+06
4.5e+06
4.0e+06
3.5e+06
3.0e+06
2.5e+06
2.0e+06
1.5e+06
1.0e+06
5.0e+05
920.5200
980.5685
1125.6315
1203.7045
1345.7124
1583.5830
1707.850
1723.8380
1795.0290
1834.9402
2106.0067
2171.9874
2310.0444
2634.3236
500
1000
1500
2000
2500
3000
3500
m/z

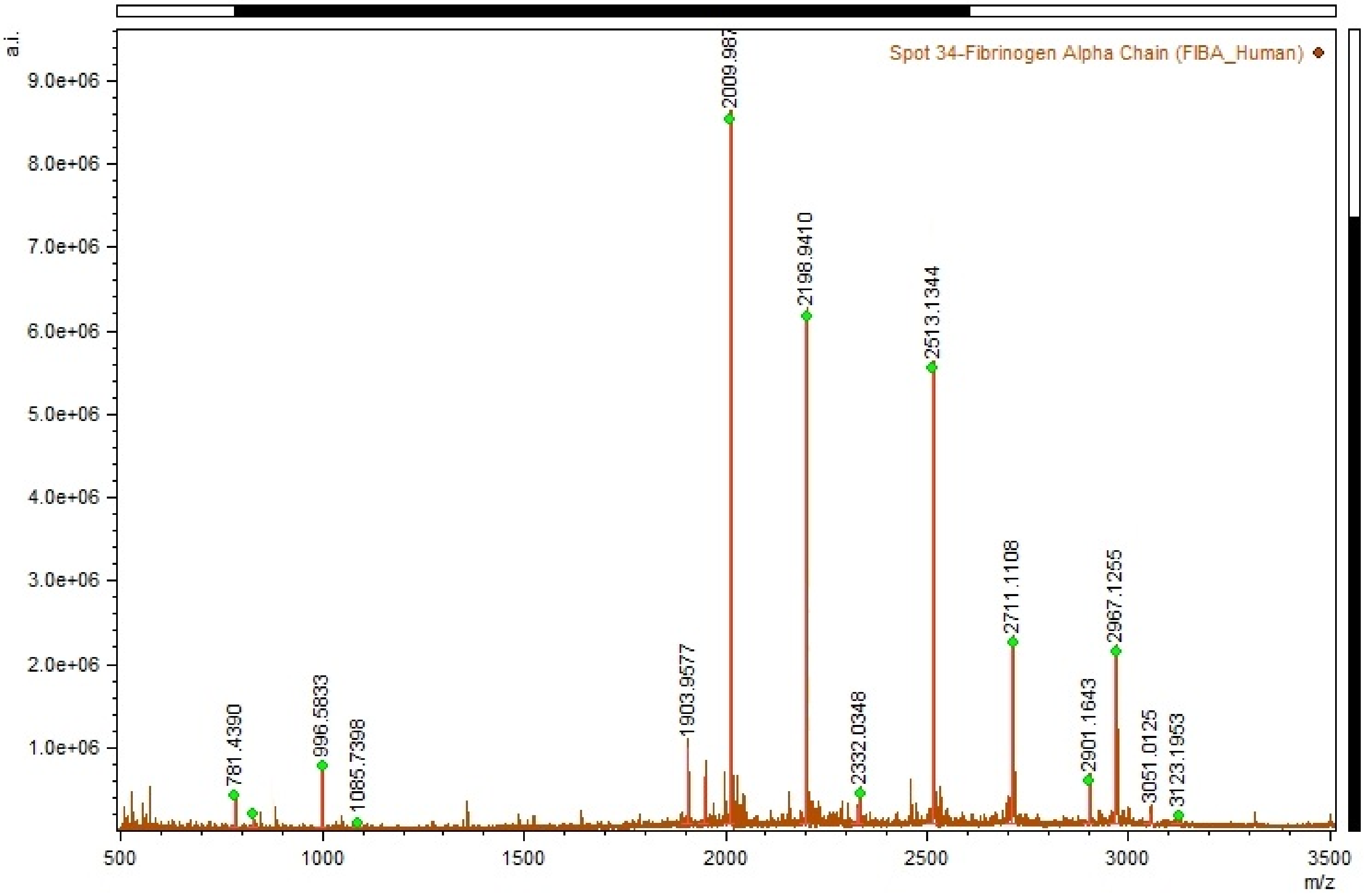
a.i.
9.0e+06
8.0e+06
7.0e+06
6.0e+06
5.0e+06
4.0e+06
3.0e+06
2.0e+06
1.0e+06
Spot 34-Fibrinogen Alpha Chain (FIBA_Human)
2009.987
2198.9410
2513.1344
2711.1108
2967.1255
1903.9577
2332.0348
2901.1643
3051.0125
3123.1953
781.4390
996.5833
1085.7398
500
1000
1500
2000
2500
3000
3500
m/z

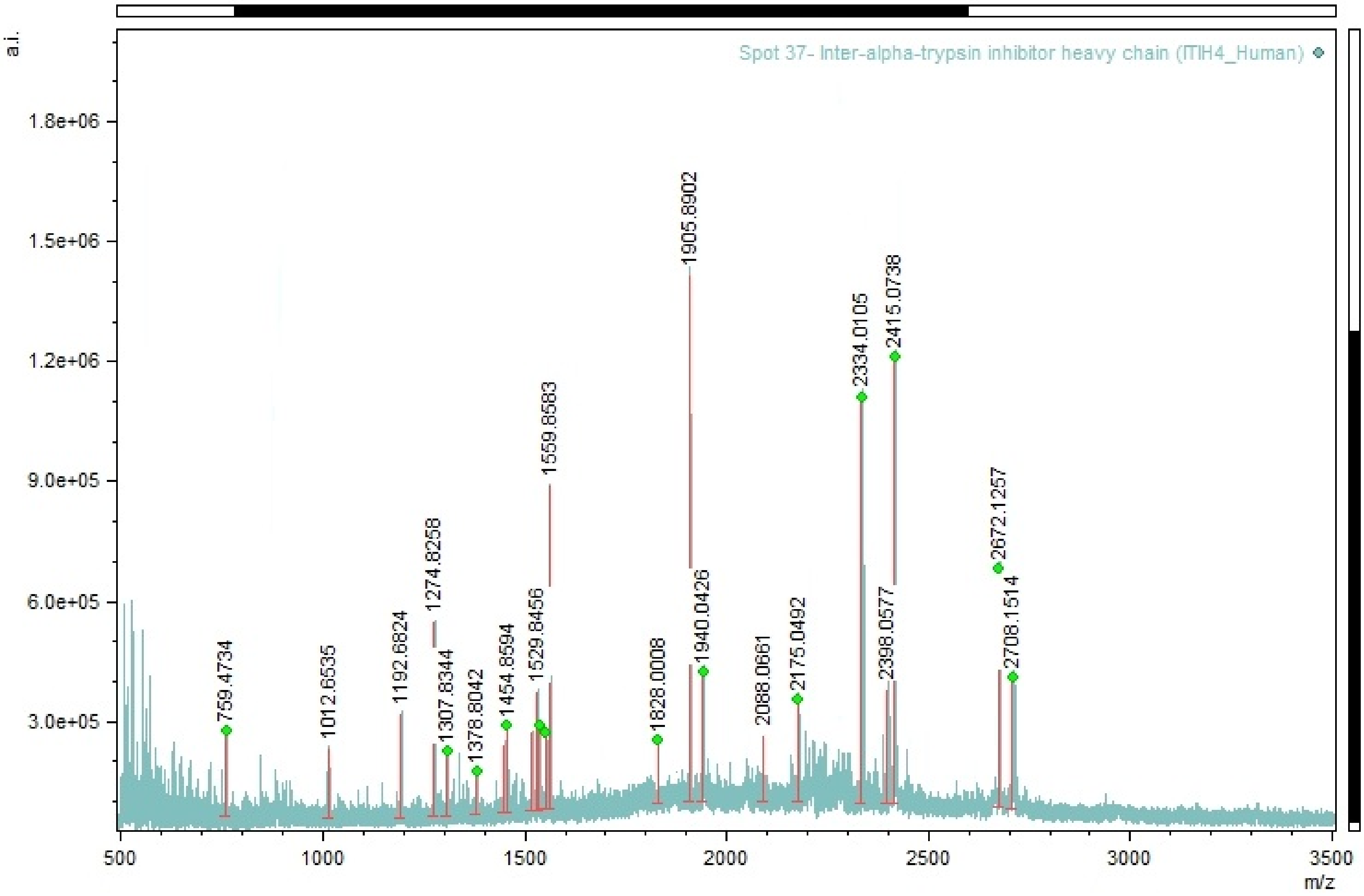

a.i.
Spot 37- Inter-alpha-trypsin inhibitor heavy chain (ITIH4_Human)
1.8e+06
1.5e+06
1.2e+06
9.0e+05
6.0e+05
3.0e+05
759.4734
1012.6535
1192.6824
1274.8258
1307.8344
1378.8042
1454.8594
1529.8456
1559.8583
1828.0008
1905.8902
1940.0426
2088.0661
2175.0492
2334.0105
2398.0577
2415.0738
2672.1257
2708.1514
500
1000
1500
2000
2500
3000
3500
m/z

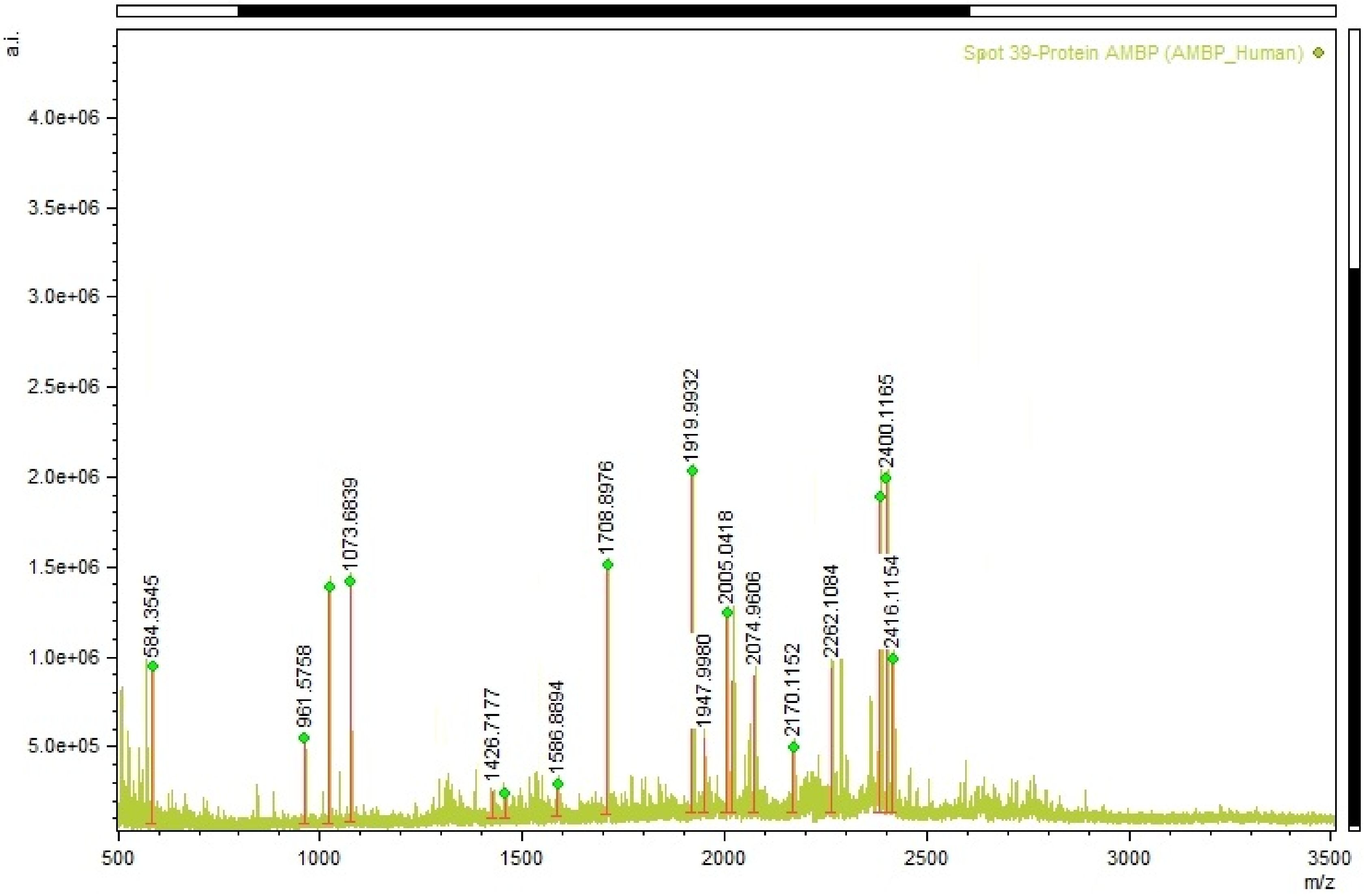

Spot 39-Protein AMBP (AMBP_Human)
a.i.
4.0e+06
3.5e+06
3.0e+06
2.5e+06
2.0e+06
1.5e+06
1.0e+06
5.0e+05
584.3545
961.5758
1073.6839
1426.7177
1586.8894
1708.8976
1919.9932
1947.9980
2005.0418
2074.9606
2170.1152
2262.1084
2400.1165
2416.1154
500
1000
1500
2000
2500
3000
3500
m/z

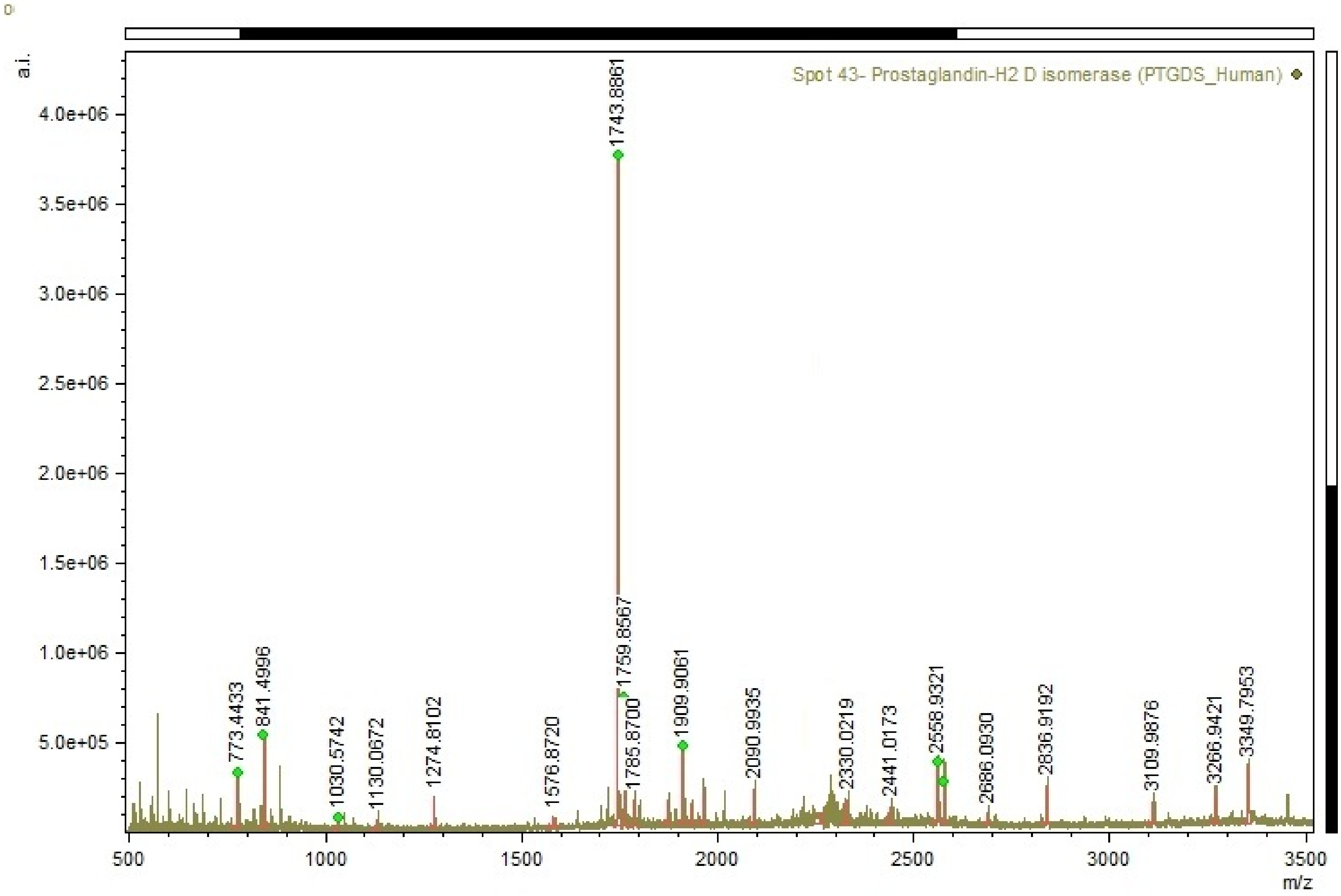

a.i.
Spot 43- Prostaglandin-H2 D isomerase (PTGDS_Human)
4.0e+06
3.5e+06
3.0e+06
2.5e+06
2.0e+06
1.5e+06
1.0e+06
5.0e+05
1743.8861
1759.8567
1785.8700
773.4433
841.4996
1030.5742
1130.0672
1274.8102
1576.8720
1909.9061
2090.9935
2330.0219
2441.0173
2558.9321
2686.0930
2836.9192
3109.9876
3266.9421
3349.7953
500
1000
1500
2000
2500
3000
3500
m/z

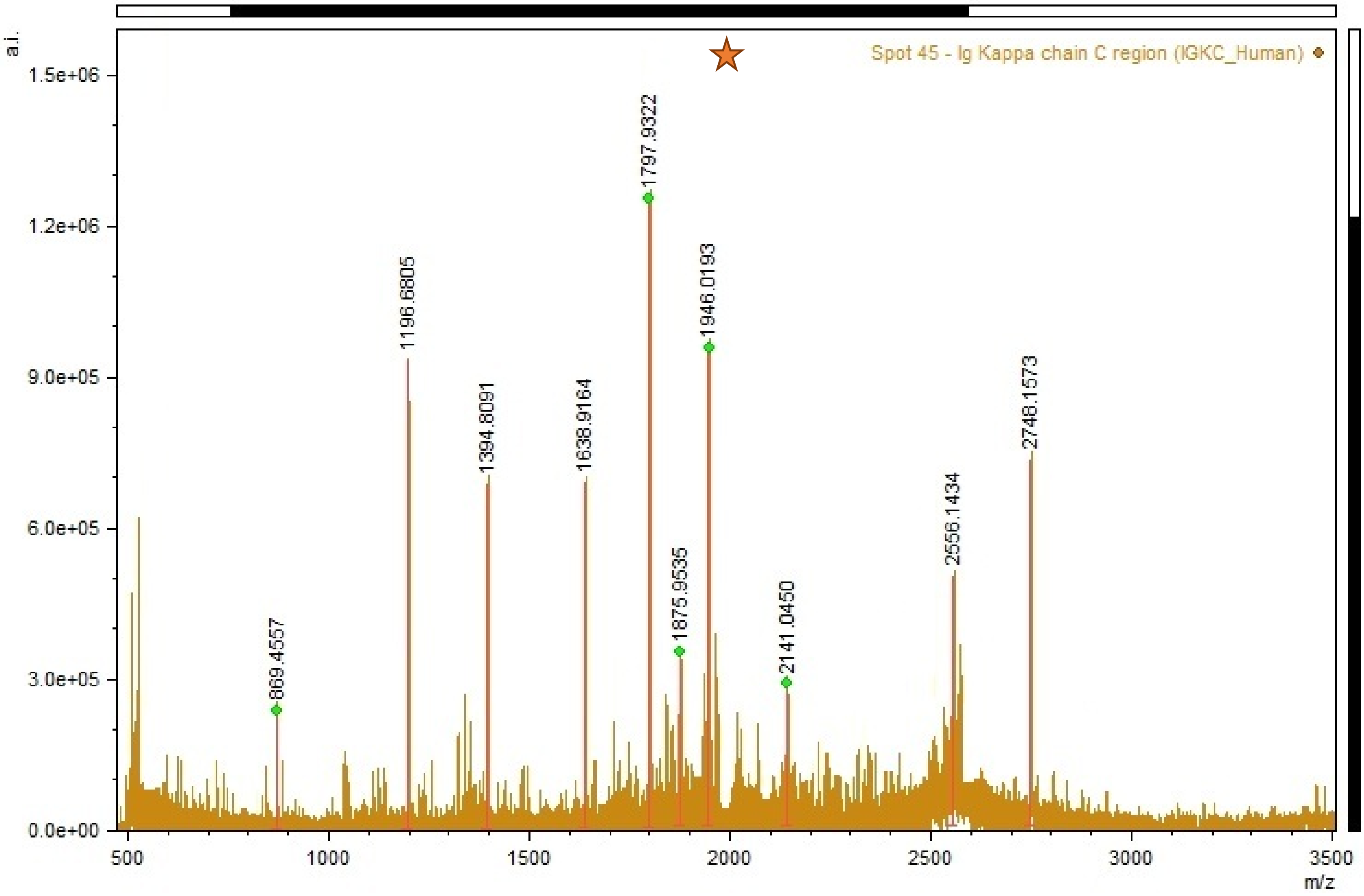

a.i.
Spot 45 - Ig Kappa chain C region (IGKC_Human)
1.5e+06
1.2e+06
9.0e+05
6.0e+05
3.0e+05
0.0e+00
869.4557
1196.6805
1394.8091
1638.9164
1797.9322
1875.9535
1946.0193
2141.0450
2556.1434
2748.1573
500
1000
1500
2000
2500
3000
3500
m/z

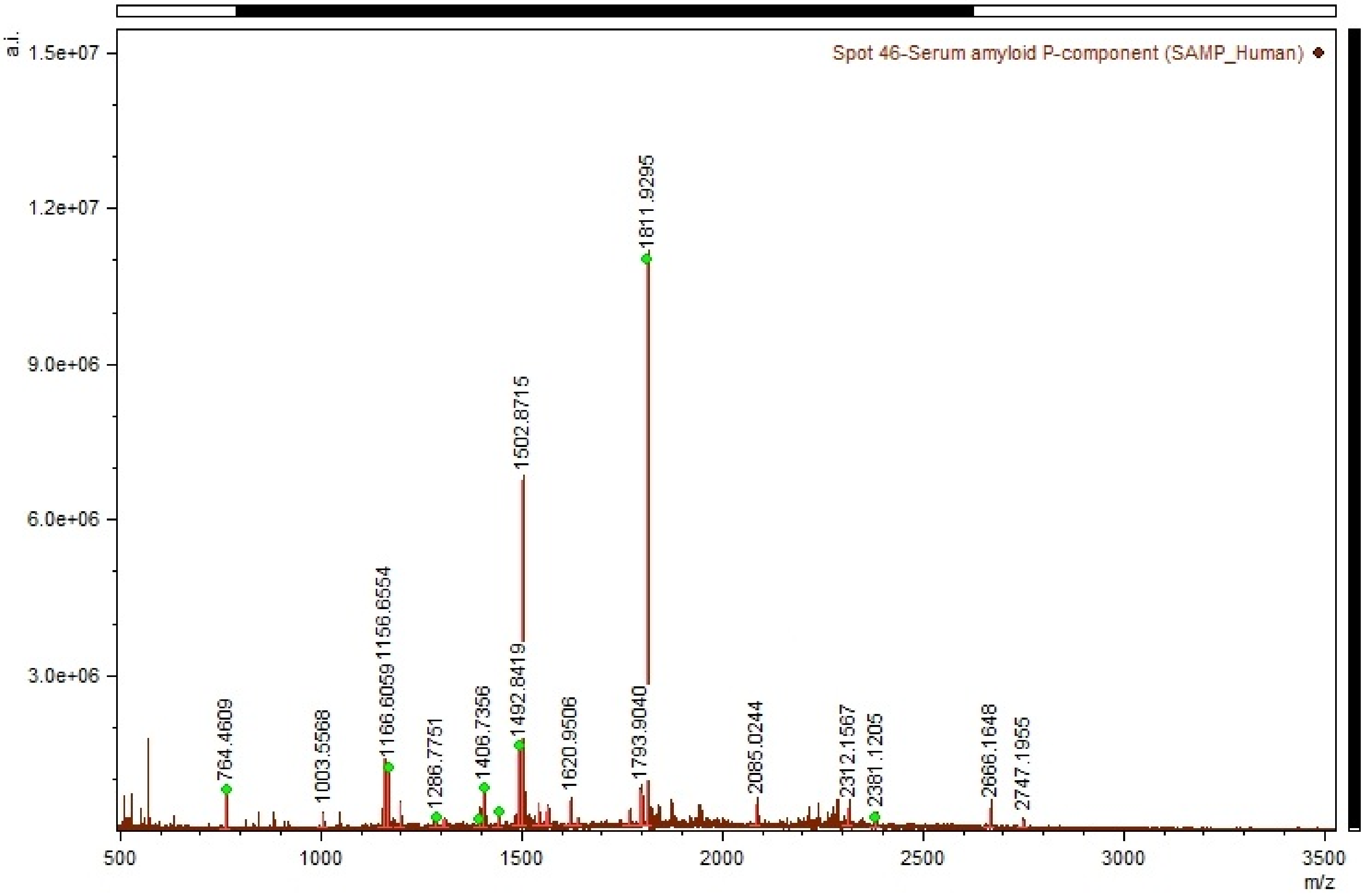
a.i.
Spot 46-Serum amyloid P-component (SAMP_Human)
1.5e+07
1.2e+07
9.0e+06
6.0e+06
3.0e+06
1811.9295
1502.8715
1156.6554
1166.6059
764.4609
1003.5568
1286.7751
1406.7356
1492.8419
1620.9506
1793.9040
2085.0244
2312.1567
2381.1205
2666.1648
2747.1955
500
1000
1500
2000
2500
3000
3500
m/z

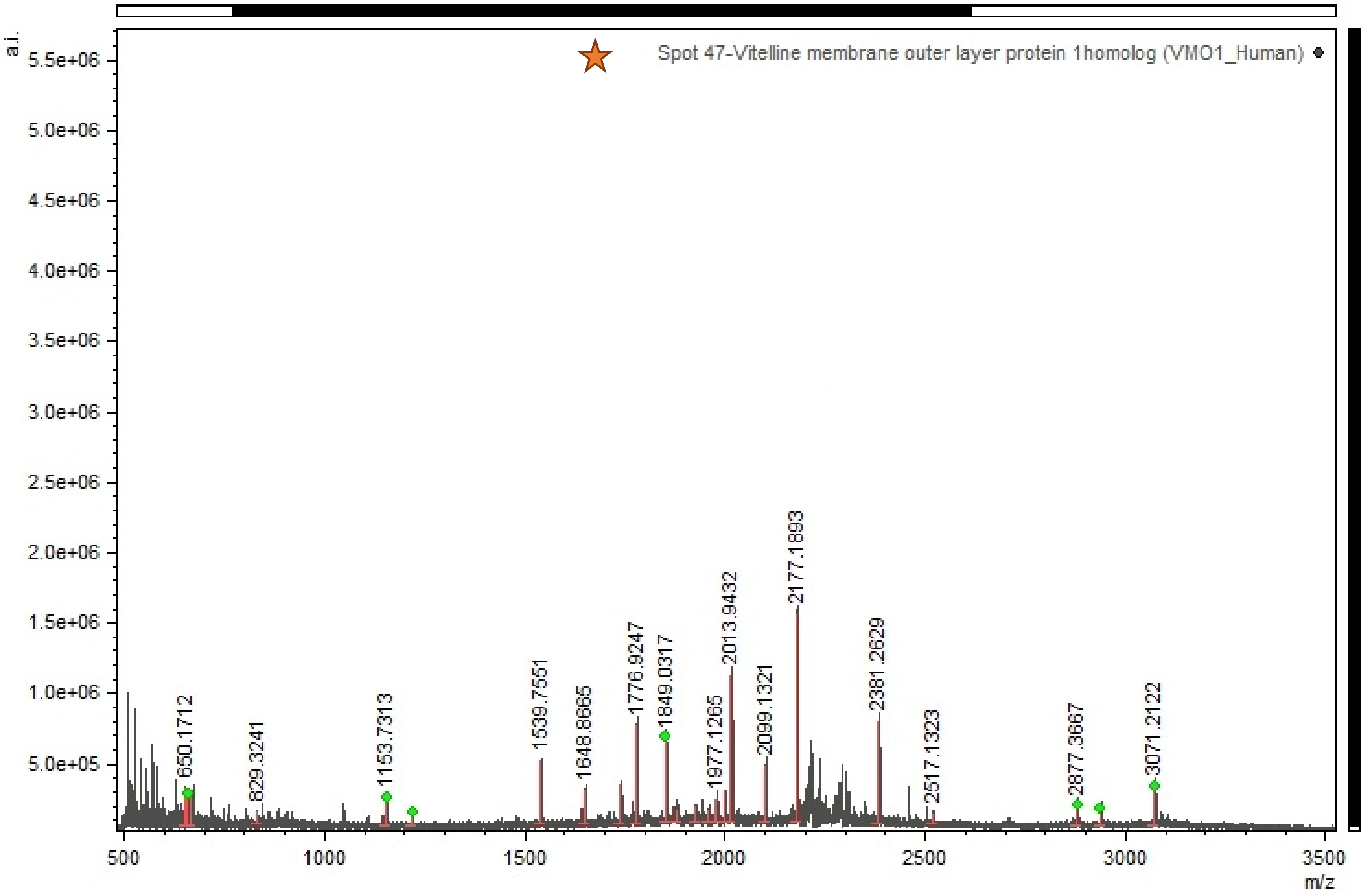
Spot 47-Vitelline membrane outer layer protein 1homolog (VMO1_Human)
a.i.
5.5e+06
5.0e+06
4.5e+06
4.0e+06
3.5e+06
3.0e+06
2.5e+06
2.0e+06
1.5e+06
1.0e+06
5.0e+05
650.1712
829.3241
1153.7313
1539.7551
1648.8665
1776.9247
1849.0317
1977.1265
2013.9432
2099.1321
2177.1893
2381.2629
2517.1323
2877.3667
3071.2122
500
1000
1500
2000
2500
3000
3500
m/z

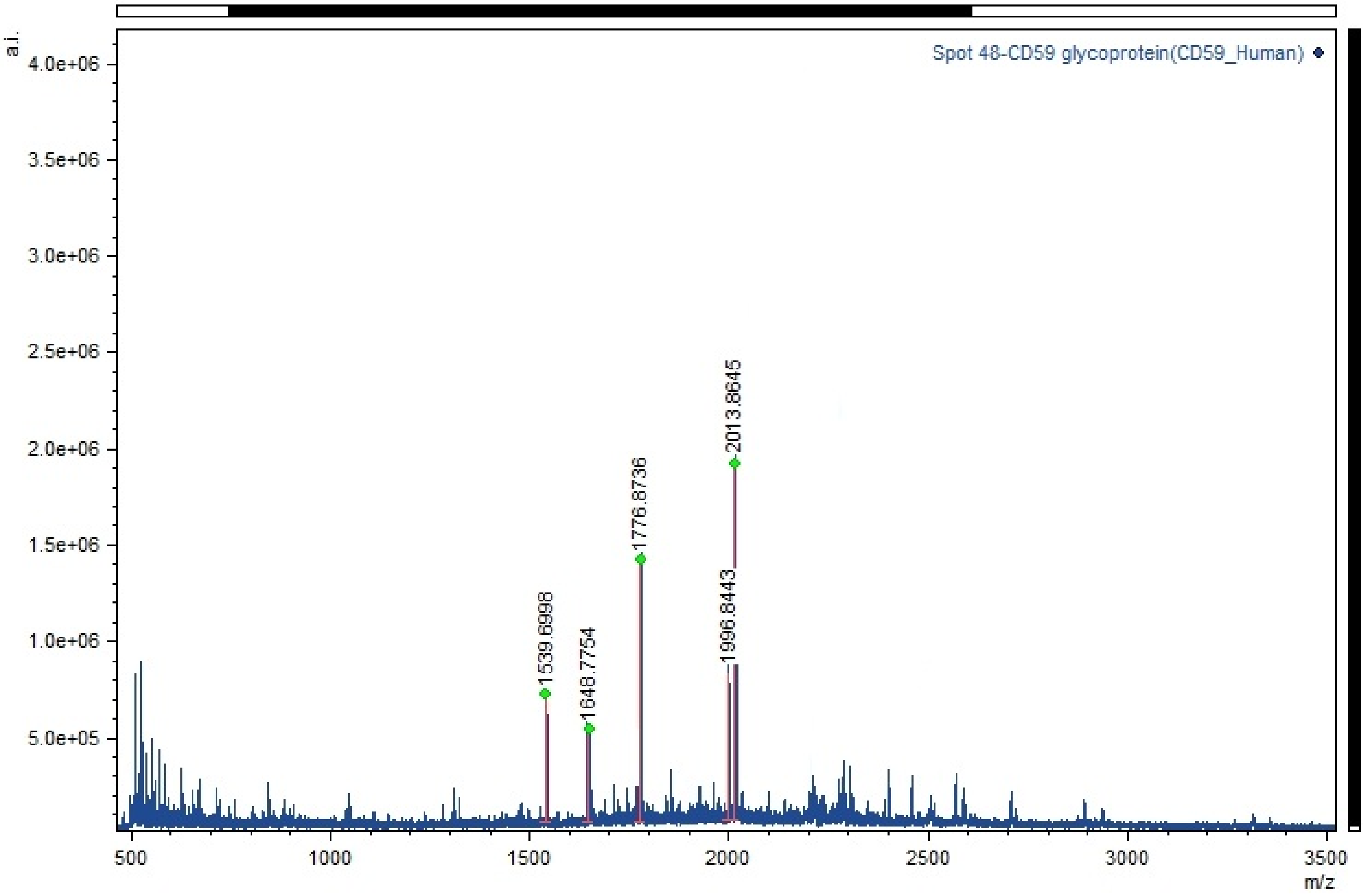
a.i.
4.0e+06
3.5e+06
3.0e+06
2.5e+06
2.0e+06
1.5e+06
1.0e+06
5.0e+05
Spot 48-CD59 glycoprotein(CD59_Human)
1539.6998
1648.7754
1776.8736
1996.8443
2013.8645
500
1000
1500
2000
2500
3000
3500
m/z

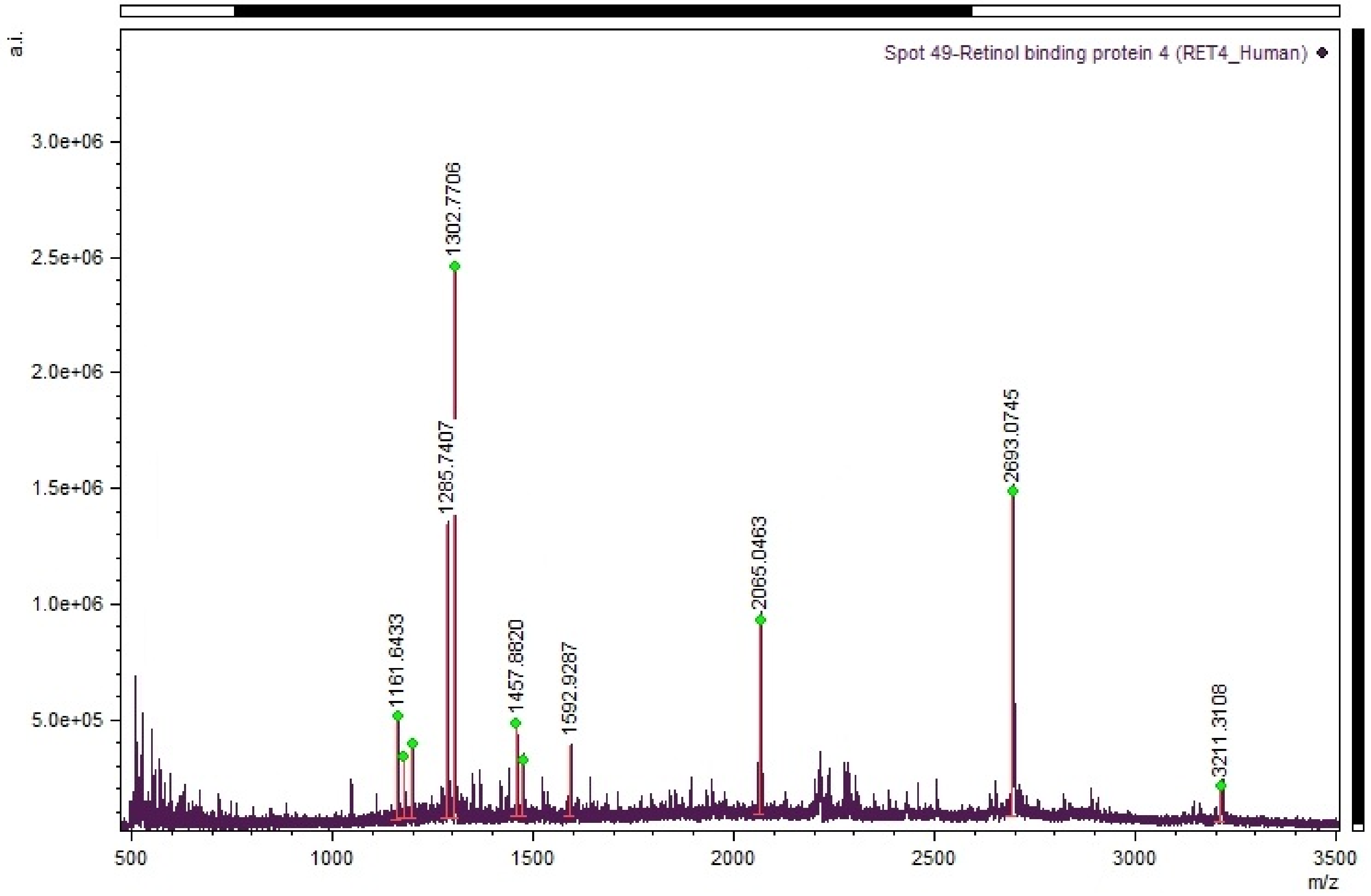

Spot 49-Retinol binding protein 4 (RET4_Human)
a.i.
3.0e+06
2.5e+06
2.0e+06
1.5e+06
1.0e+06
5.0e+05
1302.7706
1285.7407
2693.0745
2065.0463
1161.6433
1457.8820
1592.9287
3211.3108
500
1000
1500
2000
2500
3000
3500
m/z

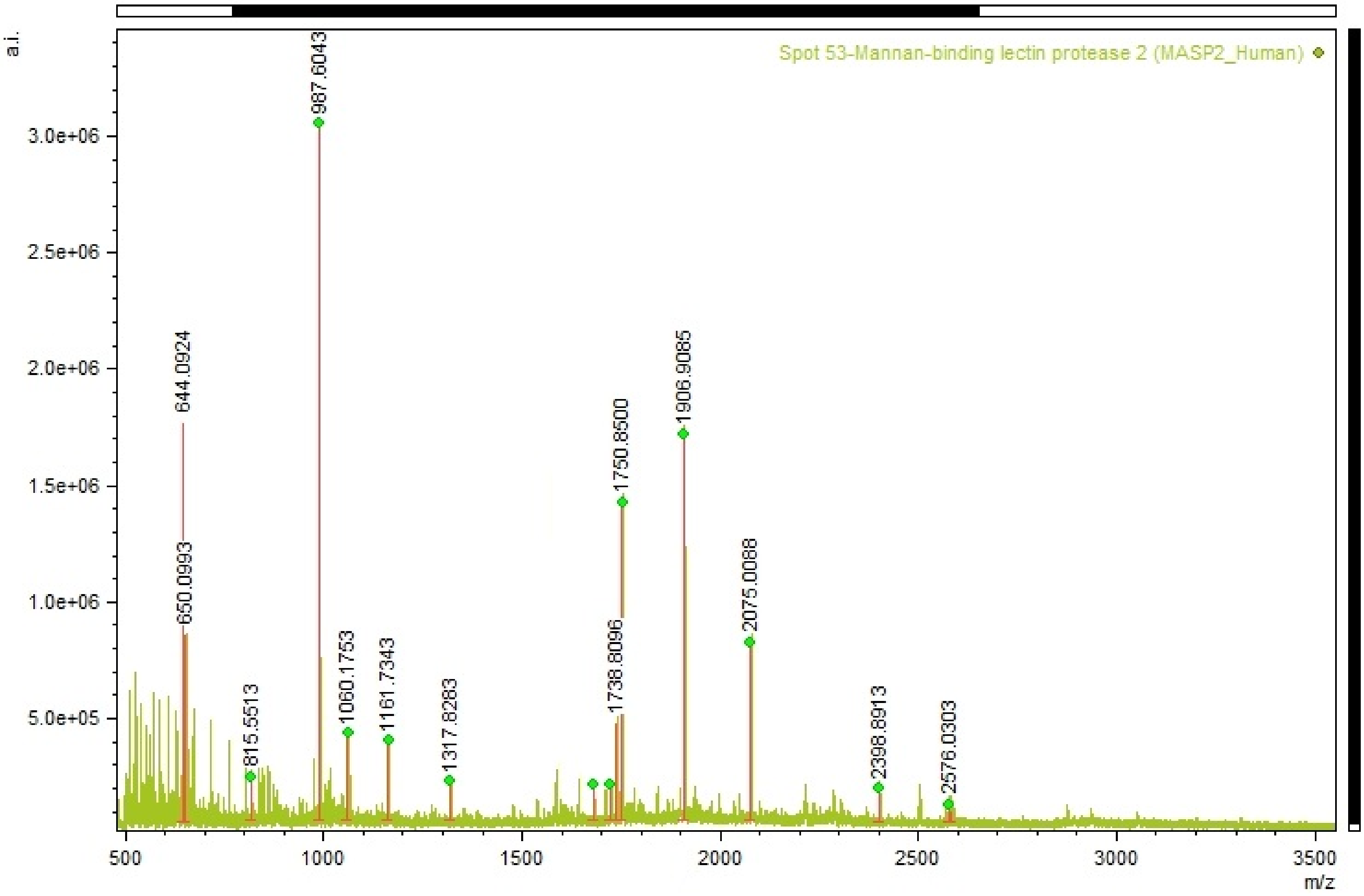
Spot 53-Mannan-binding lectin protease 2 (MASP2_Human)
a.i.
3.0e+06
2.5e+06
2.0e+06
1.5e+06
1.0e+06
5.0e+05
987.6043
644.0924
650.0993
815.5513
1060.1753
1161.7343
1317.8283
1738.8096
1750.8500
1906.9085
2075.0088
2398.8913
2576.0303
500
1000
1500
2000
2500
3000
3500
m/z

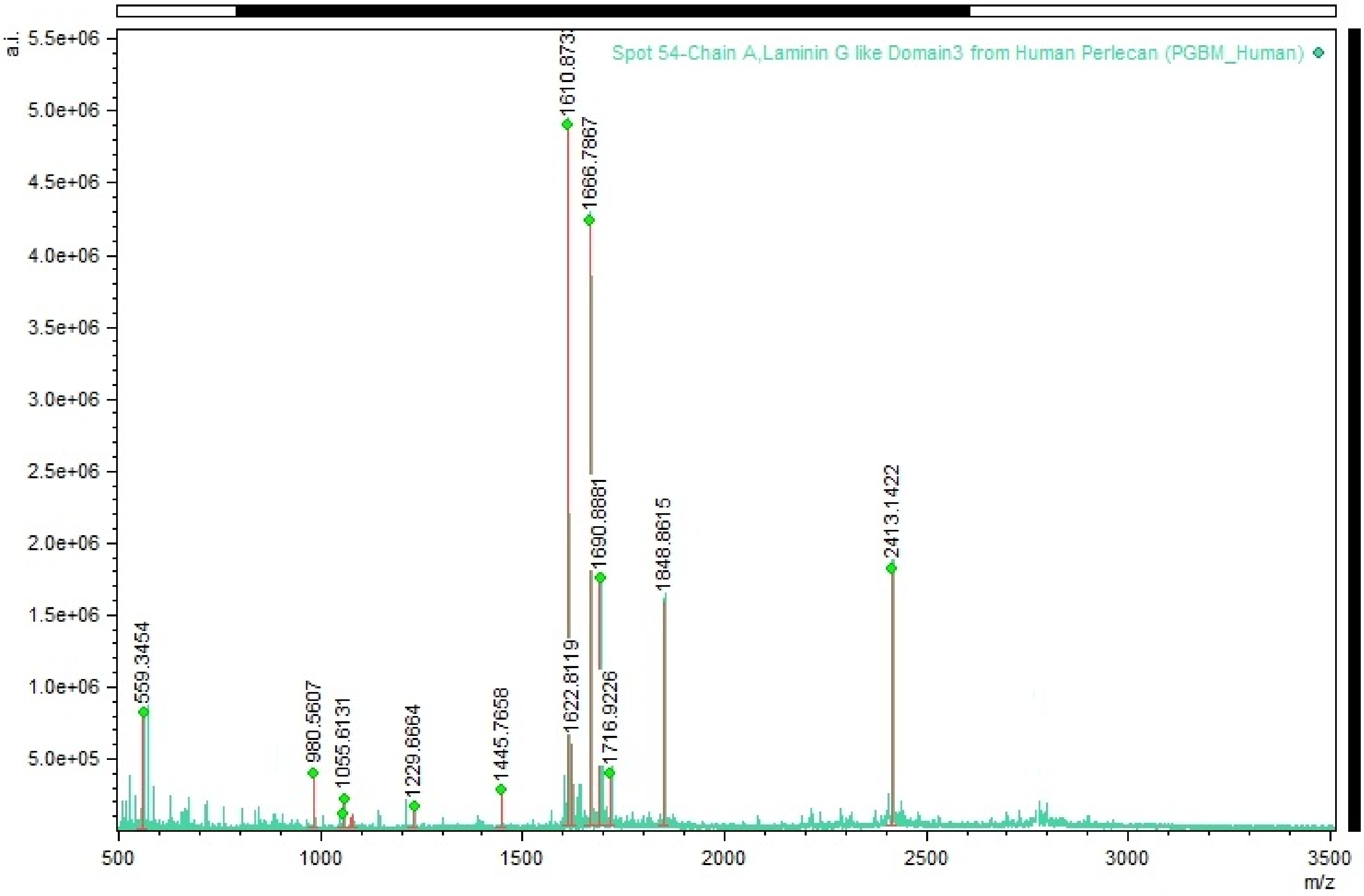

Spot 54-Chain A,Laminin G like Domain3 from Human Perlecan (PGBM_Human)
a.i.
5.5e+06
5.0e+06
4.5e+06
4.0e+06
3.5e+06
3.0e+06
2.5e+06
2.0e+06
1.5e+06
1.0e+06
5.0e+05
559.3454
980.5607
1055.6131
1229.6664
1445.7658
1610.873
1622.8119
1666.7867
1690.8881
1716.9226
1848.8615
2413.1422
500
1000
1500
2000
2500
3000
3500
m/z
