# Supplementary material for: Comparative Proteomics Analysis of Urine Reveals Down-Regulation of Acute Phase Response Signaling and LXR/RXR Activation Pathways in Prostate Cancer
Source: Proteomes. 2017 Dec 29;6(1):1. doi: 10.3390/proteomes6010001 (PMC5874760; doi:10.3390/proteomes6010001)

Supplementary document 2. Identification and quantification data of the 35 putative biomarkers for PCa in this study (proteins with statistically significant differential abundance in PCa in more than one group comparison (given in Table 1 and Figure 2) produced by Progenesis QI for proteomics V 3.0 (Nonlinear dynamics, Waters Corporation). The protein abundances in individual runs were normalized using the run that is least different from all other runs. Search settings included up to one missed cleavage, carbamidomethyl cysteine as a fixed modification and the methionine oxidation as variable modification. A minimum of 2 fragment ion matches were required per peptide identification and 5 fragment ion matches per protein identification with at least 2 peptide matches per protein identification. Quantification was done by Hi-N, using 3 most abundant peptides per protein. For each of the proteins, a table with the identified peptides as well as representative MS/MS spectra of peptides used to quantify protein expression levels is given. Peptide fragment annotation indicates the peaks that correspond to fragment ions from the peptide, and marks the positions of these ions within the peptide sequence. Peak colors legend: Red = y-series ion; Blue = b-series ion; Green = neutral loss ion.

P08571

Monocyte differentiation antigen CD14 OS=Homo sapiens GN=CD14 PE=1 SV=2
8 peptides

Sequence	Peptide Ion	Score	Hits	Mass	Charge	Tags	Conflicts	Modifications	In quantitation	Average Normalised Abundances			
										PCa	BPH	Bc	Rc
AFPALTSLDLSDNPGLGER	1607	9.60	22	1971.9901	2		0		yes	1.11e+006	4.58e+005	3.58e+005	6.19e+005
ATVNPSAPR	1193	8.35	17	911.4822	2		0		yes	3.90e+005	2.69e+005	1.55e+005	2.62e+005
CMWSSALNSLNLSFAGLEQVPK	2586	6.97	9	2451.1047	3		1	[1] Carbamidomethyl C	no	5.59e+005	7.24e+005	2.66e+005	6.00e+005
CMWSSALNSLNLSFAGLEQVPK	4104	---	---	2451.1066	2		1	[1] Carbamidomethyl C	no	1.74e+005	2.90e+004	1.37e+004	2.70e+005
LTVGAAQVPAQLLVGALR	13206	8.14	3	1776.0680	2		0		no	9041.84	1737.47	1314.00	1629.09
QYADTVK	3322	8.42	5	823.3974	2		0		no	2.60e+005	2.17e+005	2.58e+005	2.17e+005
QYADTVK	8861	8.42	5	823.3461	1		0		no	1.86e+005	1.06e+005	1.08e+005	1.67e+005
RVDADADPR	1529	8.23	19	1013.4823	2		0		no	8.47e+004	6.18e+004	3.12e+004	6.92e+004
RVDADADPR	1995	7.52	3	1013.4811	2		0		no	7.46e+004	2.67e+004	1.45e+004	2.69e+004
VLDLSCNR	1266	8.82	15	975.4798	2		0	[6] Carbamidomethyl C	no	1.98e+005	1.01e+005	9.70e+004	1.22e+005
VLSIAQAHSPAFSCEQVR	2033	8.50	17	1998.9711	3		0	[14] Carbamidomethyl C	yes	6.49e+005	4.78e+005	2.82e+005	4.54e+005
VLSIAQAHSPAFSCEQVR	2973	8.70	2	1999.0043	3		0	[14] Carbamidomethyl C	no	2.46e+005	7.93e+004	4.71e+004	6.29e+004

Fragment ions for: AFPALTSLDLSDNPGLGER

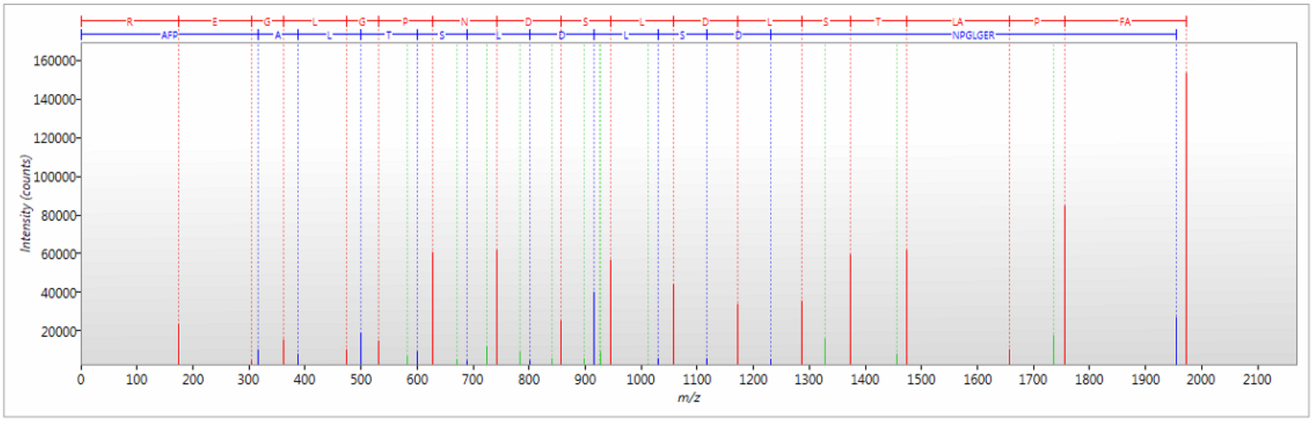

Fragment ions for: ATVNPSAPR

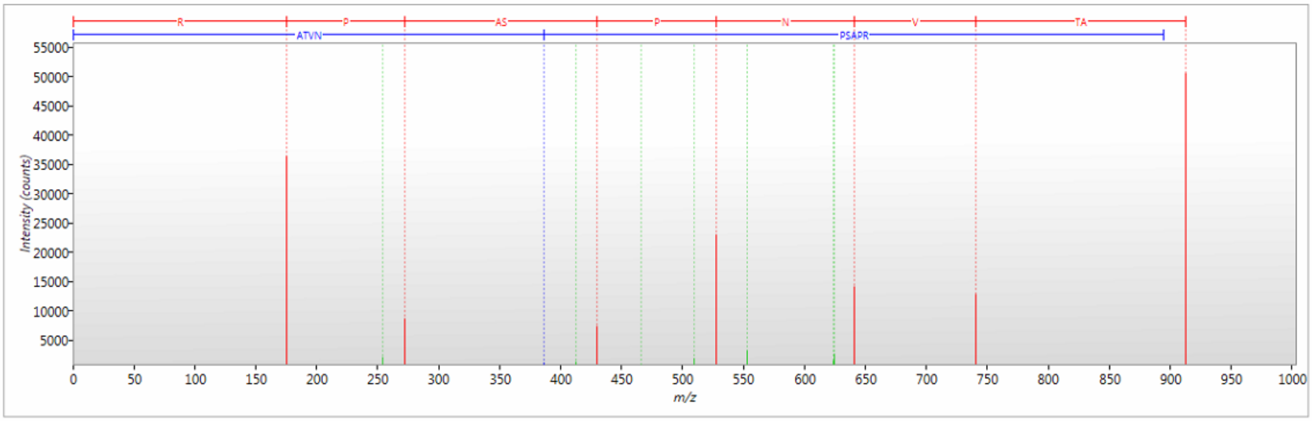

Fragment ions for: VLSIAQAHSPAFSCEQVR

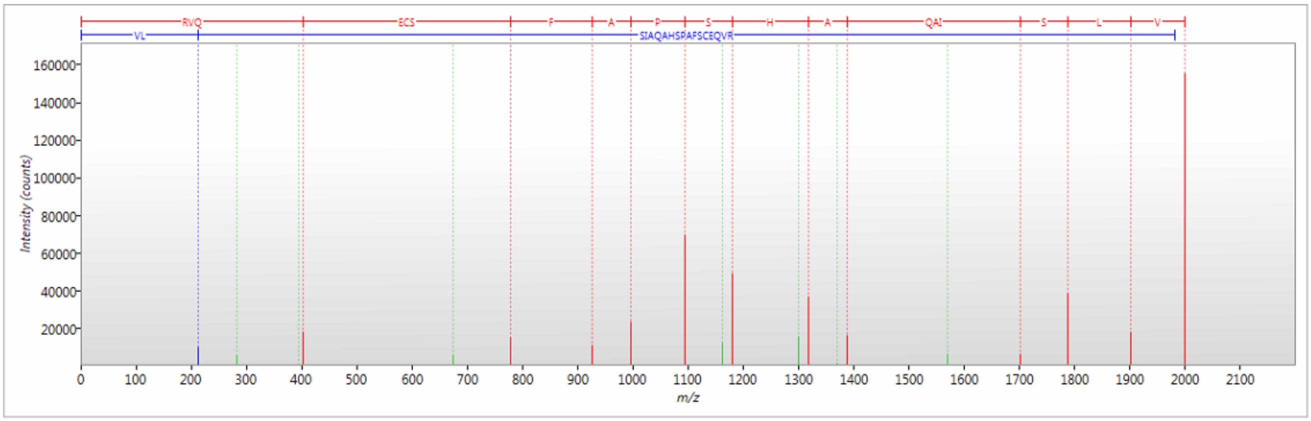

P62736

Actin_ aortic smooth muscle OS=Homo sapiens GN=ACTA2 PE=1 SV=1
3 peptides

Sequence	Peptide Ion	Score	Hits	Mass	Charge	Tags	Conflicts	Modifications	In quantitation	Average Normalised Abundances			
										PCa	BPH	Bc	Rc
CEEEDSTALVCDNGSGLCK	1150	5.58	2	2142.8759	3		0	[1] Carbamidomethyl C | [11] Carbamidomethyl C | [18] Carbamidomethyl C	yes	1.40e+005	8.33e+004	9.61e+005	7.53e+004
CEEEDSTALVCDNGSGLCK	3717	6.34	2	2142.8534	2		0	[1] Carbamidomethyl C | [11] Carbamidomethyl C | [18] Carbamidomethyl C	yes	1.50e+005	6.25e+005	5.52e+005	3.26e+005
YPIEHGIITNWDDMEKIWHHSFYNELR	11856	6.28	2	3442.7453	5		0		yes	1.68e+005	4.49e+005	1.18e+006	6.37e+005
YSVWIGGSILASLSTFQQMWISKQEYDEAGPSIVHR	35065	6.01	3	4082.8749	4		0		no	2.75e+005	3.62e+004	1548.87	5.06e+004

Fragment ions for: CEEEDSTALVCDNGSGLCK

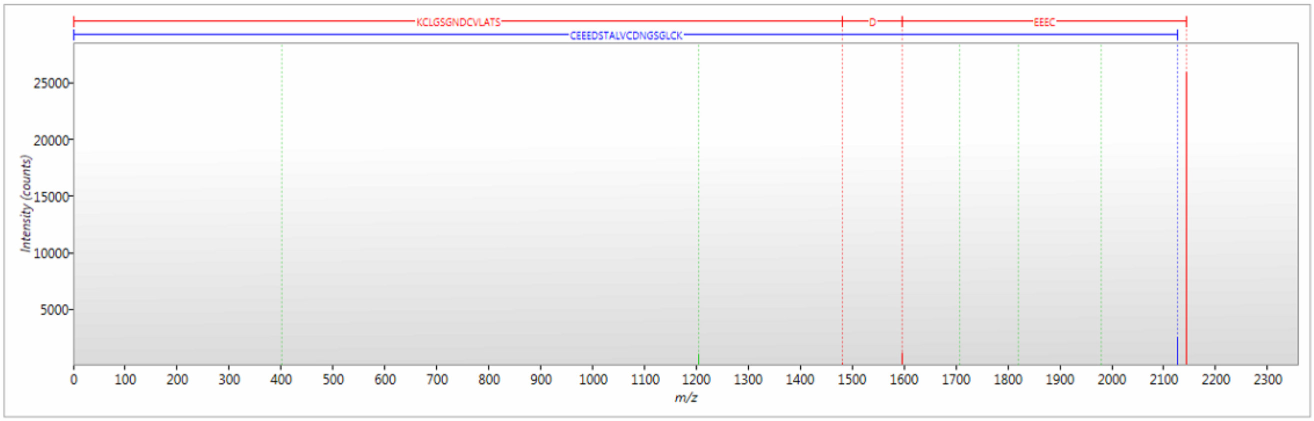

Fragment ions for: CEEEDSTALVCDNGSGLCK

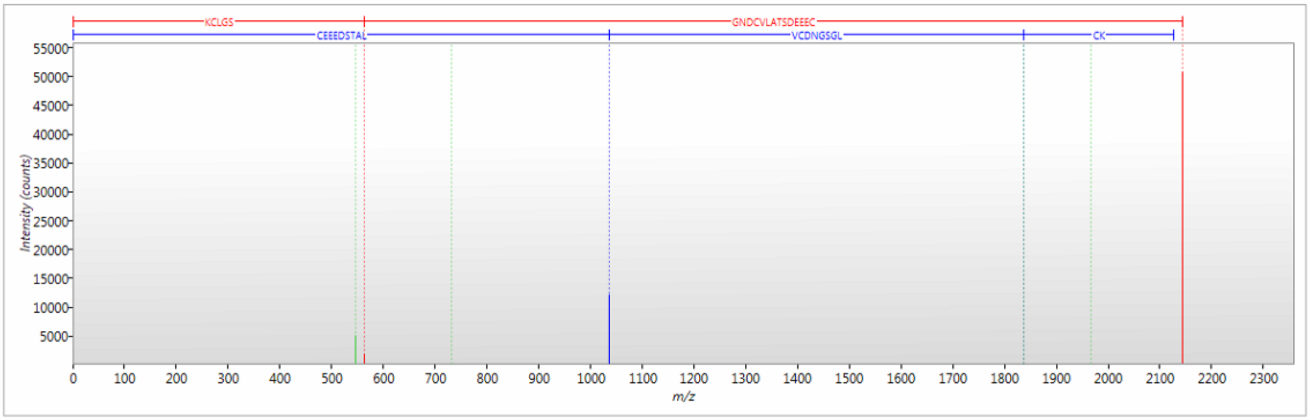

Fragment ions for: YPIEHGIVTNWDDMEKIWHHTFYNELR

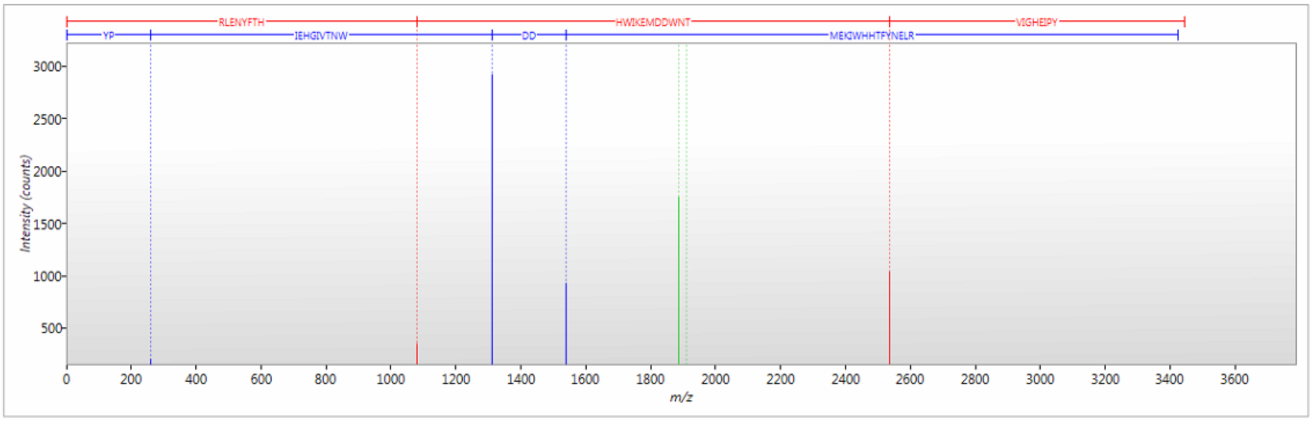

P02765

Alpha-2-HS-glycoprotein OS=Homo sapiens GN=AHSG PE=1 SV=1
9 peptides

Sequence	Peptide Ion	Score	Hits	Mass	Charge	Tags	Conflicts	Modifications	In quantitation	Average Normalised Abundances			
										PCa	BPH	Bc	Rc
AQLVPLPPSTYVEFTVSGTDCVAK	7440	8.35	20	2578.2956	2		0	[21] Carbamidomethyl C	yes	1.03e+006	7.79e+004	9.75e+004	4.48e+005
AQLVPLPPSTYVEFTVSGTDCVAKEATEAAK	11451	7.74	6	3278.6310	3		0	[21] Carbamidomethyl C	no	1.44e+005	1.13e+004	7236.49	6.05e+004
CDSSPDSAEDVR	26963	7.96	2	1336.5567	3		0	[1] Carbamidomethyl C	no	1042.38	1289.09	396.14	369.53
EHAVEGDCDFQLLK	2392	6.48	2	1659.8029	3		0	[8] Carbamidomethyl C	no	1.62e+005	3.75e+005	1.74e+005	1.73e+005
EHAVEGDCDFQLLK	3226	9.08	9	1659.8030	3		0	[8] Carbamidomethyl C	no	6.87e+004	1.24e+005	1.28e+005	1.09e+005
EHAVEGDCDFQLLK	48117	7.57	2	1659.7538	1		0	[8] Carbamidomethyl C	no	13.40	0.00	1207.73	30.73
FSVVYAK	743	8.96	34	812.4408	2		0		no	3.65e+005	5.34e+005	2.97e+005	3.51e+005
FSVVYAK	1409	8.91	2	812.4034	2		0		no	3.61e+005	3.66e+005	3.38e+005	2.75e+005
HTLNQIDEVK	1367	9.49	6	1195.6177	2		0		no	1.46e+005	3.08e+004	6.92e+004	1.55e+004
QPNCDDPETEEAALVAIDYINQNLPWGYK	25585	10.01	16	3362.5197	2		0	[4] Carbamidomethyl C	no	1.48e+004	241.60	754.79	3927.96
QPNCDDPETEEAALVAIDYINQNLPWGYK	30935	9.86	2	3362.5106	2		0	[4] Carbamidomethyl C	no	1.33e+004	0.00	0.00	0.00
TVVQPSVGAAAGPVVPPCPGR	2193	9.48	35	2015.0521	3		0	[18] Carbamidomethyl C	yes	4.21e+005	2.60e+005	1.53e+005	2.91e+005
TVVQPSVGAAAGPVVPPCPGR	2286	9.48	34	2015.0407	2		0	[18] Carbamidomethyl C	yes	5.58e+005	2.10e+005	1.79e+005	4.63e+005
VCQDCPLLAPLNDTR	2683	6.51	2	1770.8115	2		0	[2] Carbamidomethyl C | [5] Carbamidomethyl C	yes	4.45e+005	6.38e+005	4.79e+005	3.92e+005

Fragment ions for: AQLVPLPPSTYVEFTVSGTDCVAK

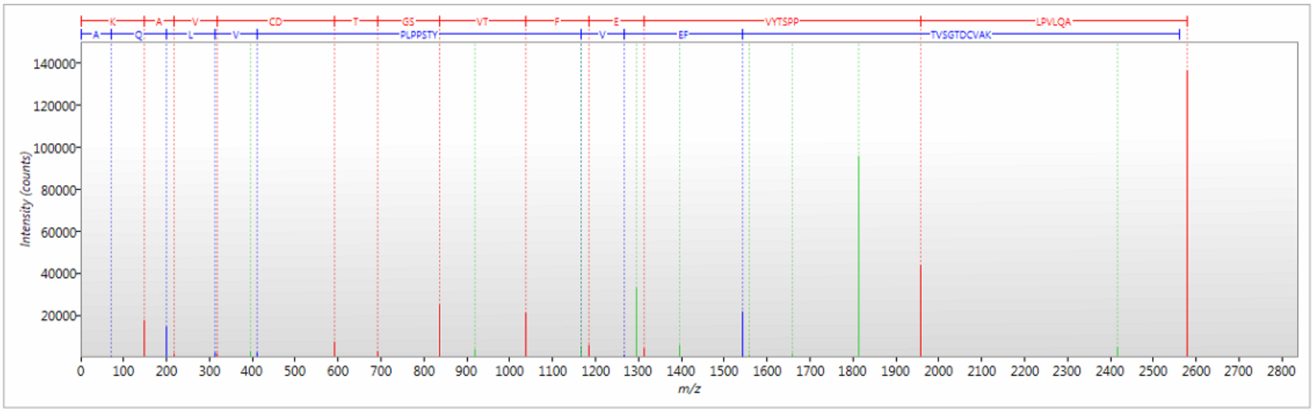

Fragment ions for: TVVQPSVGAAAGPVVPPCPGR

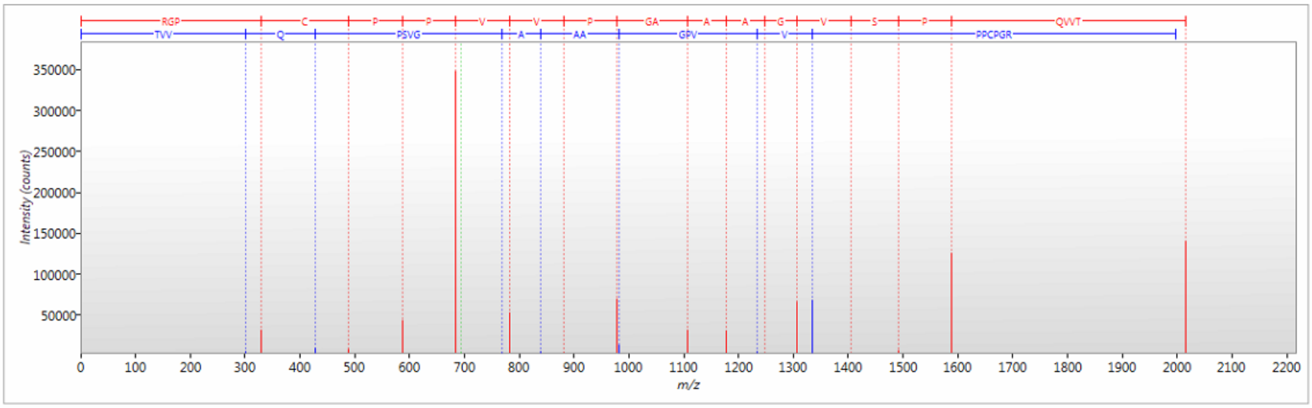

Fragment ions for: VCQDCPLLAPLNDTR

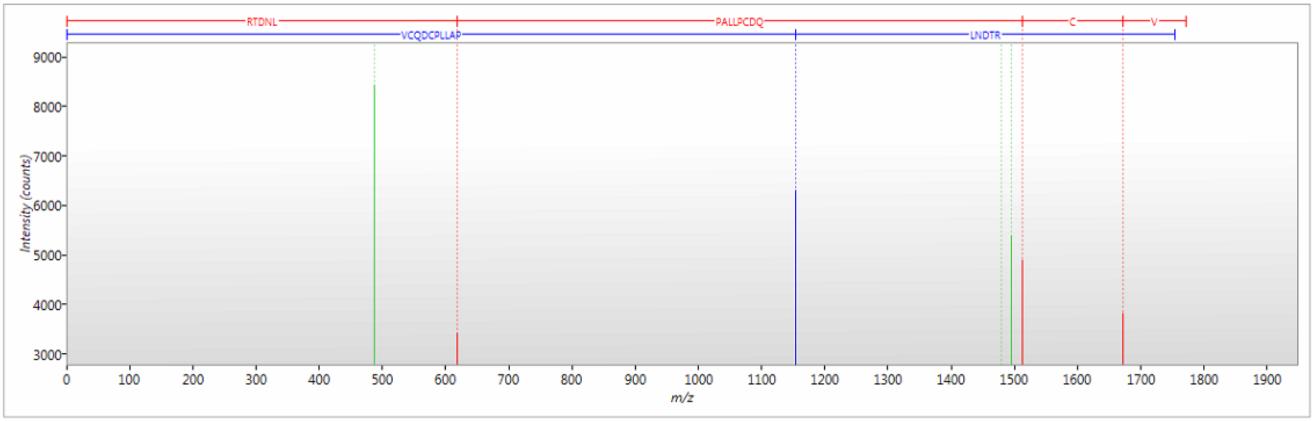

P04745

Alpha-amylase 1 OS=Homo sapiens GN=AMY1A PE=1 SV=2
9 peptides

Sequence	Peptide Ion	Score	Hits	Mass	Charge	Tags	Conflicts	Modifications	In quantitation	Average Normalised Abundances			
										PCa	BPH	Bc	Rc
ALVFVDNHDNQR	3349	8.66	23	1426.6995	3		0		no	3.88e+004	3.08e+004	5147.43	6.14e+004
DFPAVPYSGWDFNDGK	3244	9.05	24	1813.8456	2		1		yes	6.36e+005	3.06e+005	1.28e+005	4.56e+005
EVTINPDTTCGNDWVCEHR	4172	5.97	4	2302.0265	3		0	[10] Carbamidomethyl C | [16] Carbamidomethyl C	no	1.52e+005	5.15e+004	2.66e+004	1.64e+005
EVTINPDTTCGNDWVCEHR	8041	8.53	5	2301.9755	2		0	[10] Carbamidomethyl C | [16] Carbamidomethyl C	no	4.16e+004	1.41e+004	6300.43	6.08e+004
IYVSDDGK	1405	9.15	24	895.4270	2		0		no	2.18e+005	1.99e+005	1.29e+005	2.29e+005
IYVSDDGK	8308	9.15	23	895.4154	1		0		no	2.58e+004	1.20e+004	1.12e+004	3.31e+004
NVVDGQPFTNWYDNGSNQVAFGR	4959	8.67	20	2584.1530	2		1		no	1.52e+005	1.06e+005	4.87e+004	2.84e+005
SGNEDEFR	1796	8.12	5	952.3979	2		0		no	3.83e+004	4.28e+004	2.72e+004	8.30e+004
TGSGDIENYNDATQVR	828	9.47	27	1738.7766	2		1		yes	5.69e+005	3.87e+005	1.83e+005	6.98e+005
TSIVHLFEWR	12668	6.36	2	1286.6785	3		1		no	1082.51	2.10e+004	7.72	4.21e+004
WVDIALECER	1288	9.17	17	1289.6045	2		0	[8] Carbamidomethyl C	yes	4.74e+005	2.75e+005	1.74e+005	3.72e+005
WVDIALECER	12939	9.17	9	1289.5980	1		0	[8] Carbamidomethyl C	no	1.15e+004	368.54	298.47	7586.60

Fragment ions for: DFPAVPYSGWDFNDGK

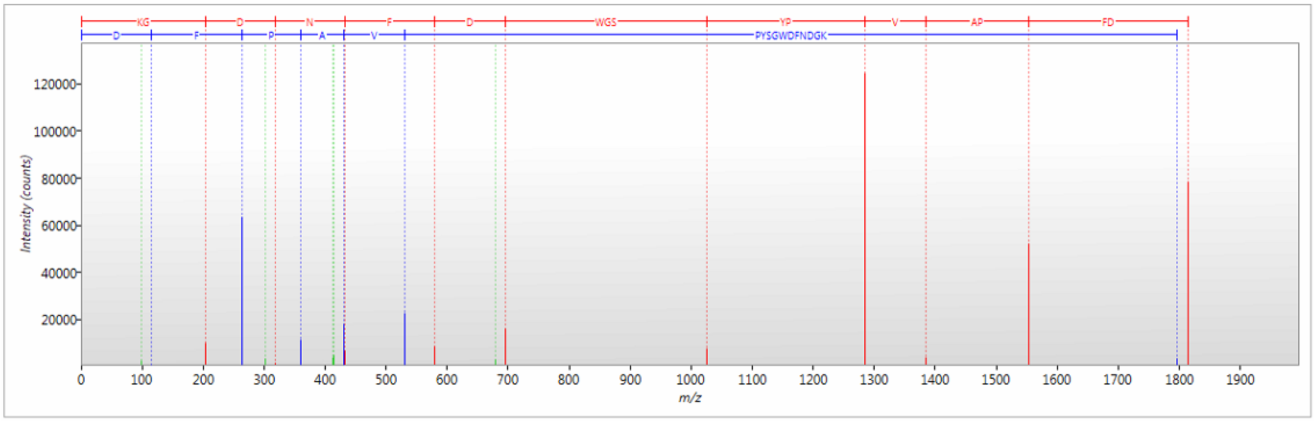

Fragment ions for: TGSGDIENYNDATQVR

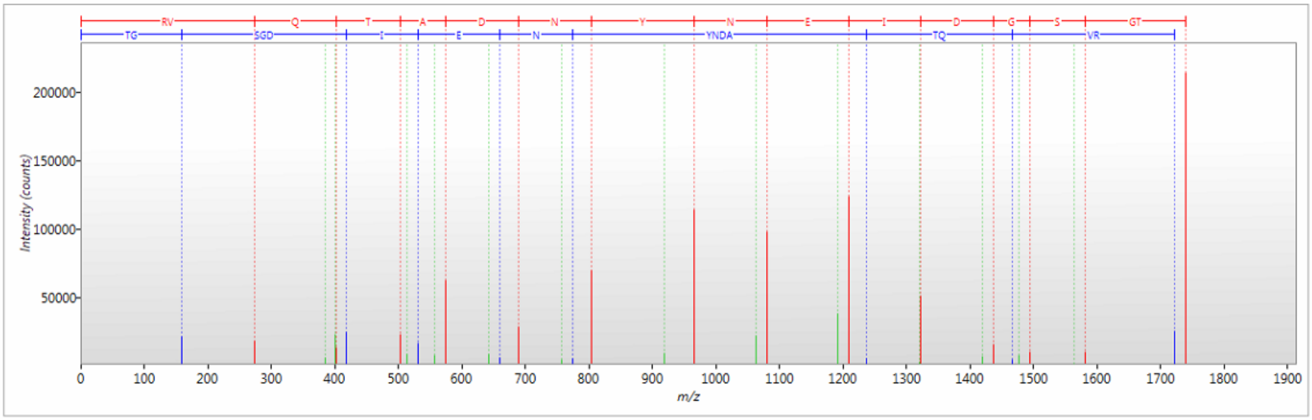

Fragment ions for: WVDIALECER

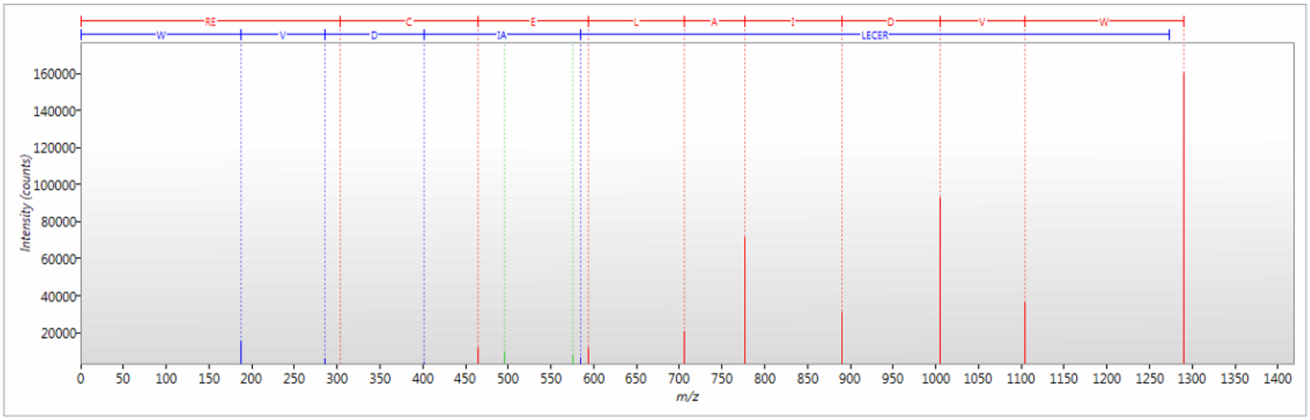

P19961

Alpha-amylase 2B OS=Homo sapiens GN=AMY2B PE=1 SV=1
3 peptides

Sequence	Peptide Ion	Score	Hits	Mass	Charge	Tags	Conflicts	Modifications	In quantitation	Average Normalised Abundances			
										PCa	BPH	Bc	Rc
CNNVGVRIYVDAVINHMSGNAVSAGTSSTCGSYFNPGSR	25324	6.20	2	4117.8162	3		0	[1] Carbamidomethyl C | [30] Carbamidomethyl C	yes	1.33e+004	754.84	126.46	9901.89
NVVDGQPFTNWYDNGSNQVAFGR	4959	8.67	20	2584.1530	2		1		yes	1.52e+005	1.06e+005	4.87e+004	2.84e+005
TSIVHLFEWR	12668	6.36	2	1286.6785	3		1		yes	1082.51	2.10e+004	7.72	4.21e+004

Fragment ions for: CNNVGVRIYVDAVINHMSGNAVSAGTSSTCGSYFNPGSR

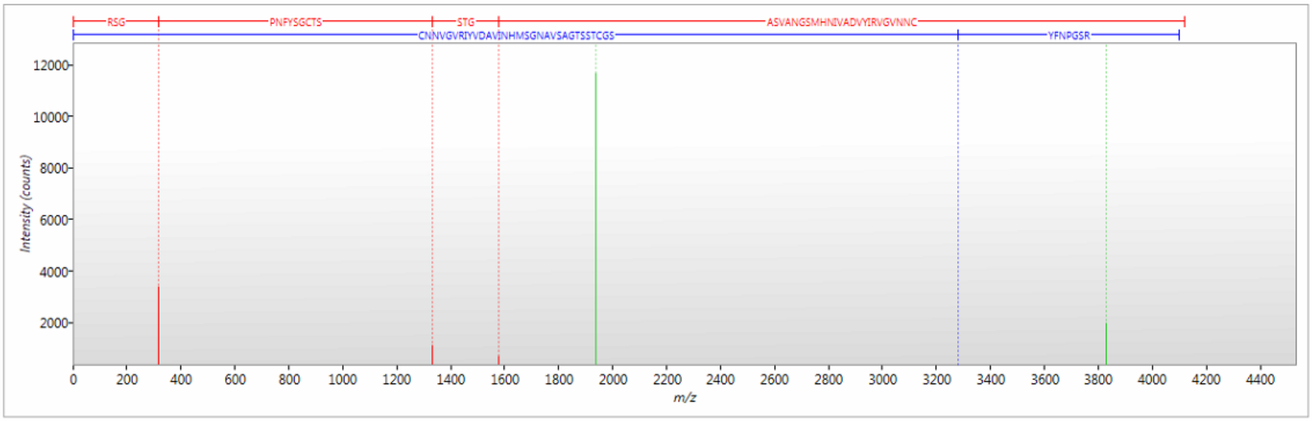

Fragment ions for: NVVDGQPFTNWYDNGSNQVAFGR

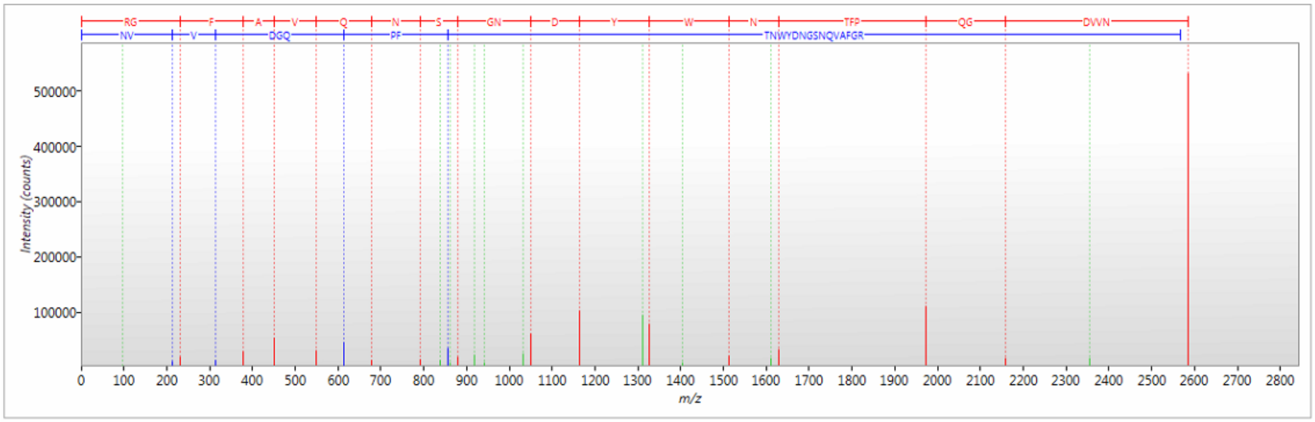

Fragment ions for: TSIVHLFEWR

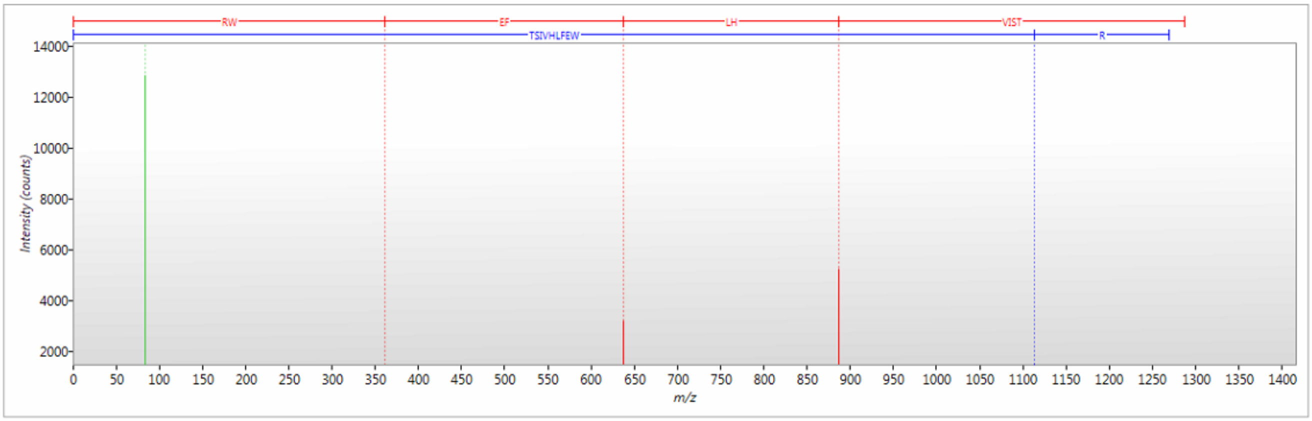

P06733

Alpha-enolase OS=Homo sapiens GN=ENO1 PE=1 SV=2
2 peptides

Sequence	Peptide Ion	Score	Hits	Mass	Charge	Tags	Conflicts	Modifications	In quantitation	Average Normalised Abundances			
										PCa	BPH	Bc	Rc
DYPVVSIEDPFDQDDWGAWQK	28693	7.37	5	2509.1077	2		0		yes	2356.92	3097.84	1849.30	4420.39
TIAPALVSK	3471	7.36	6	898.4641	2		0		yes	2.08e+005	3.72e+005	3.15e+005	2.24e+005

Fragment ions for: DYPVVSIEDPFDQDDWGAWQK

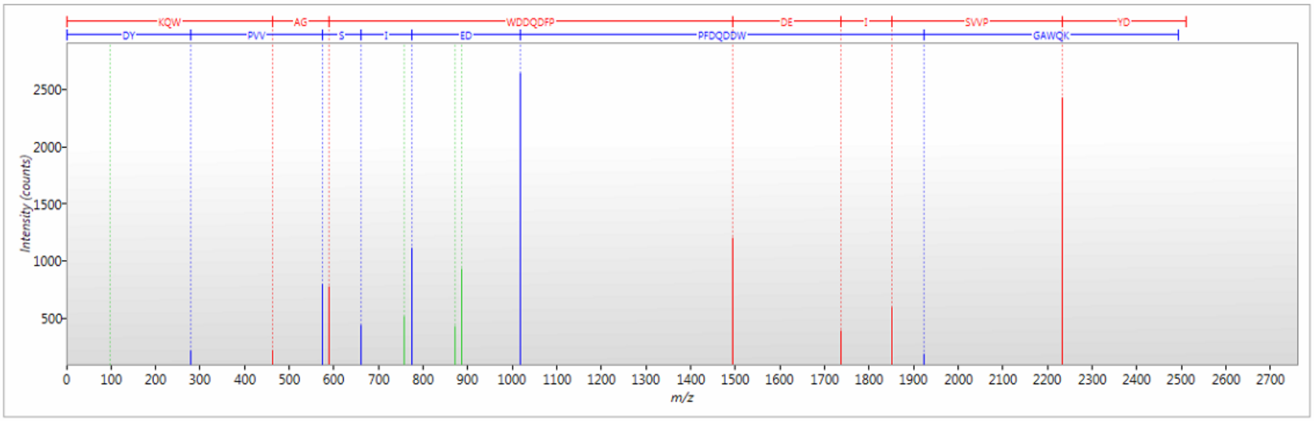

Fragment ions for: TIAPALVSK

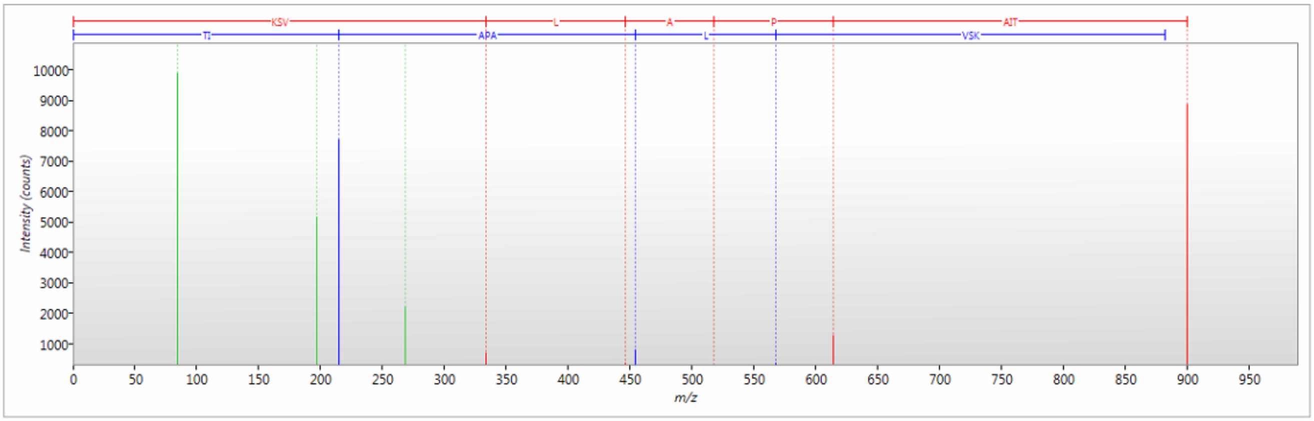

P04083

Annexin A1 OS=Homo sapiens GN=ANXA1 PE=1 SV=2
5 peptides

Sequence	Peptide Ion	Score	Hits	Mass	Charge	Tags	Conflicts	Modifications	In quantitation	Average Normalised Abundances			
										PCa	BPH	Bc	Rc
AAYLQETGKPLDETLK	4547	7.25	2	1775.8335	2		0		yes	5.74e+005	4.34e+005	3.06e+005	5.17e+005
DITSDTSGDFR	29302	8.71	2	1212.5313	1		0		yes	299.89	26.93	0.00	5007.30
GDRSEDFGVNEDLADSDAR	203	7.96	3	2066.8788	2		0		yes	1.99e+006	7.27e+005	3.72e+005	3.18e+006
GTDVNVFNTILTTR	53352	8.53	2	1549.6953	1		0		no	289.55	0.00	810.81	0.00
TPAQFDADELR	48550	8.57	2	1261.5912	1		0		no	599.28	24.91	628.43	1788.98

Fragment ions for: AAYLQETGKPLDETLK

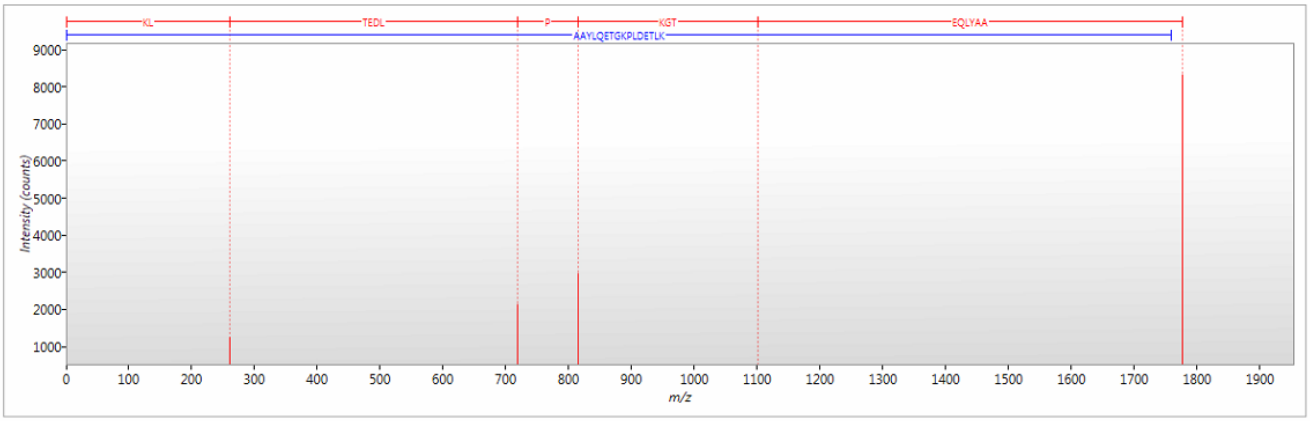

Fragment ions for: DITSDTSGDFR

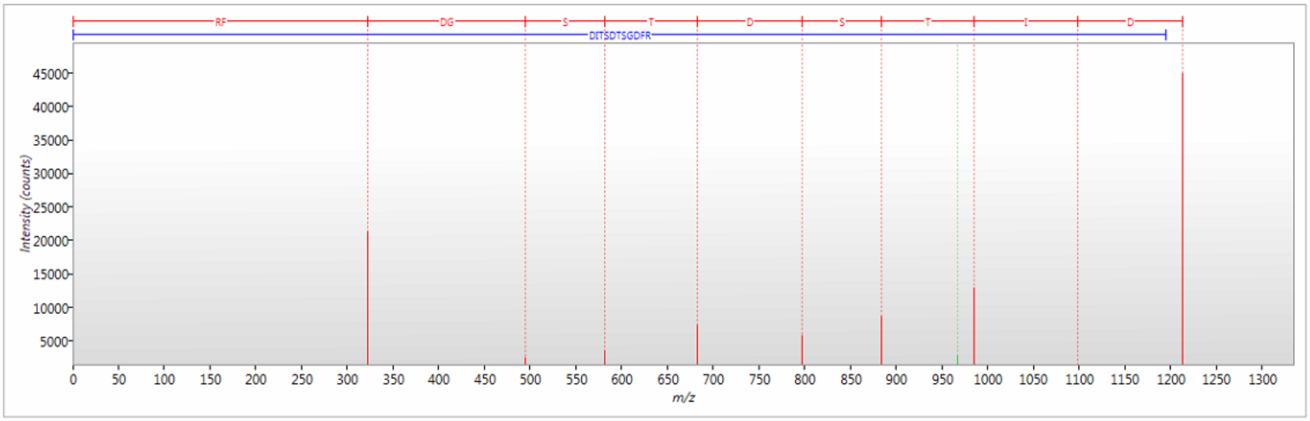

Fragment ions for: GDRSEDFGVNEDLADSDAR

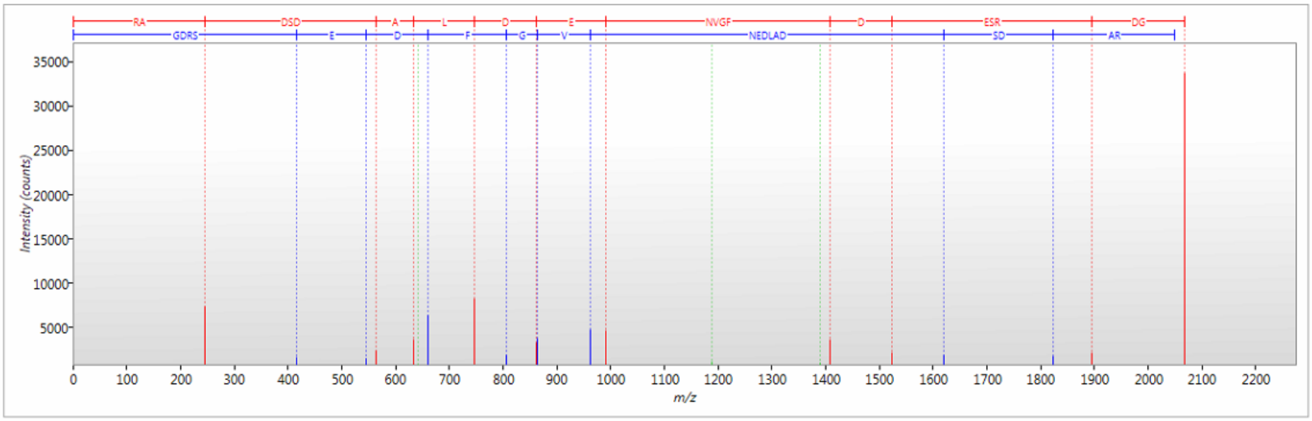

P20160

Azurocidin OS=Homo sapiens GN=AZU1 PE=1 SV=3
2 peptides

Sequence	Peptide Ion	Score	Hits	Mass	Charge	Tags	Conflicts	Modifications	In quantitation	Average Normalised Abundances			
										PCa	BPH	Bc	Rc
GPDFFTR	2261	7.02	5	838.4108	2		0		yes	6.48e+004	1.83e+005	1.15e+005	7.90e+004
QFPFLASIQNQGR	32351	6.74	2	1504.7693	1		0		yes	539.89	88.25	3340.67	502.05

Fragment ions for: GPDFFTR

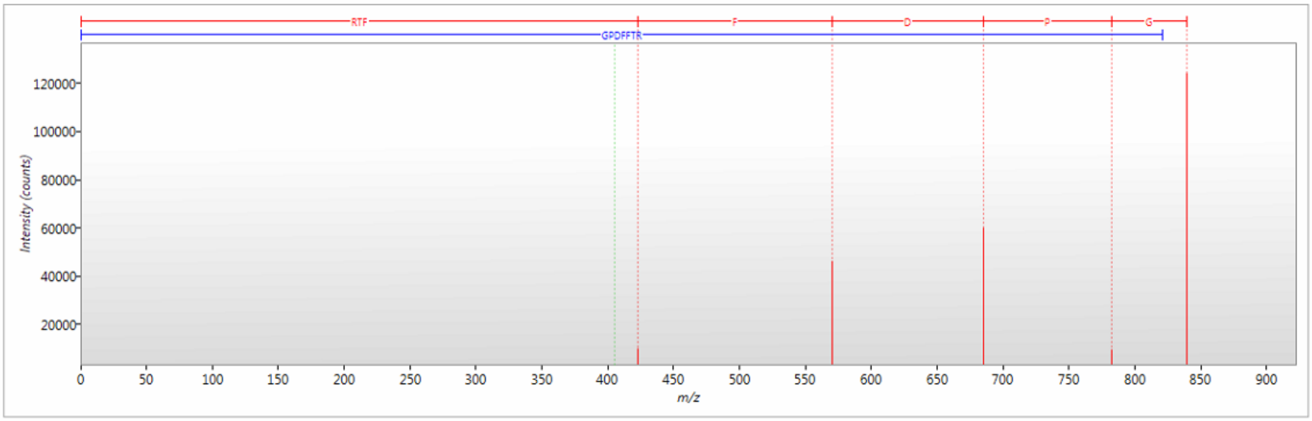

Fragment ions for: QFPFLASIQNQGR

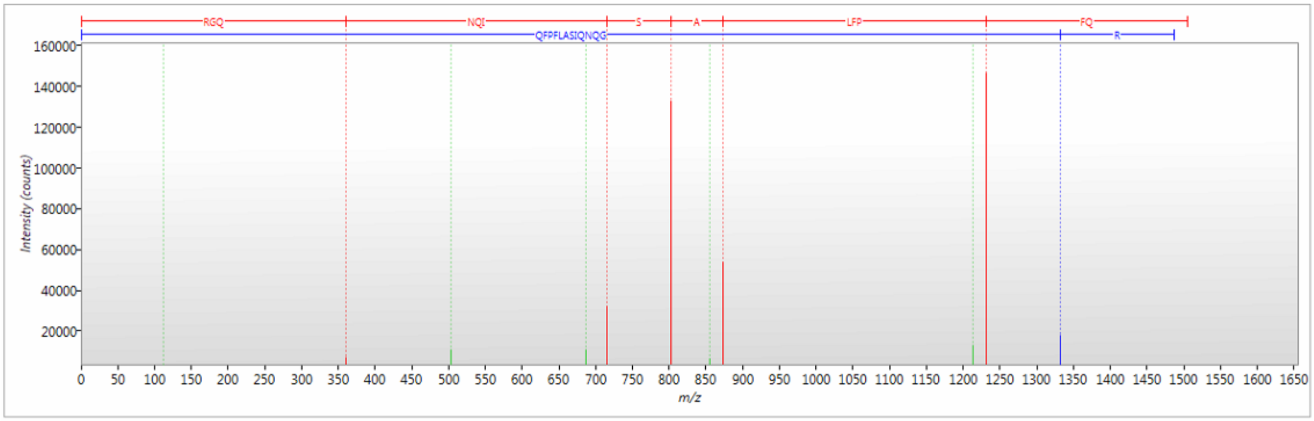

P10909

Clusterin OS=Homo sapiens GN=CLU PE=1 SV=1
10 peptides

Sequence	Peptide Ion	Score	Hits	Mass	Charge	Tags	Conflicts	Modifications	In quantitation	Average Normalised Abundances			
										PCa	BPH	Bc	Rc
ALQEYR	8245	8.70	5	778.3929	1		0		no	7765.59	4141.64	4177.99	1.38e+004
ASSIIDELFQDR	2351	8.61	30	1392.7006	2		0		no	3.93e+005	2.89e+005	2.59e+005	2.96e+005
ASSIIDELFQDR	39635	8.40	7	1392.6822	1		0		no	18.26	57.08	0.00	1022.51
EILSVDCSTNNPSQAK	829	9.40	34	1761.8174	2		0	[7] Carbamidomethyl C	yes	4.89e+005	2.65e+005	2.29e+005	7.42e+005
EILSVDCSTNNPSQAK	46692	9.40	8	1761.8062	1		0	[7] Carbamidomethyl C	no	123.76	0.00	196.31	2321.54
EIQNAVNGVK	17391	7.93	9	1070.5611	1		0		no	6050.02	1917.85	4897.45	9389.06
HNSTGCLR	4833	6.66	4	943.4447	2		0	[6] Carbamidomethyl C	no	7.51e+004	1.21e+005	5.87e+004	8.67e+004
IDSLLENDR	142	8.49	2	1073.5498	2		0		yes	1.50e+006	4.48e+006	3.08e+006	1.91e+006
IDSLLENDR	2043	---	---	1073.5510	3		0		yes	2.20e+004	6.97e+004	7.77e+004	2.19e+004
IDSLLENDR	3602	---	---	1073.5449	1		0		yes	2.50e+004	7.76e+004	1.08e+005	5.04e+004
LKELPGVCNETMMALWEECKPCLK	7176	7.07	4	2967.3754	3		0	[8] Carbamidomethyl C|[12] Oxidation M|[13] Oxidation M|[19] Carbamidomethyl C|[22] Carbamidomethyl C	no	1.25e+005	4.09e+005	4.96e+004	5.47e+005
TLIEKTNEER	21629	7.77	2	1231.6410	3		0		no	891.81	3.50	101.28	15.12
TLLSNLEEAK	1375	8.07	10	1116.5636	2		0		yes	3.43e+005	4.91e+005	3.43e+005	4.01e+005
VTTVASHTSDSDVPSGVTEVVVK	6455	8.75	35	2313.1640	2		0		no	7.56e+004	3.14e+004	3.55e+004	1.40e+005

Fragment ions for: EILSVDCSTNNPSQAK

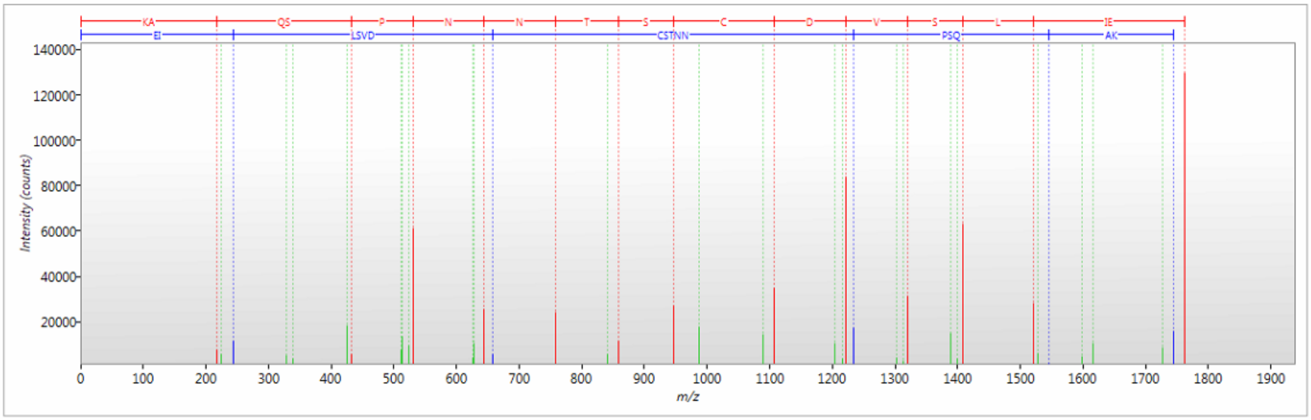

Fragment ions for: IDSLLENDR

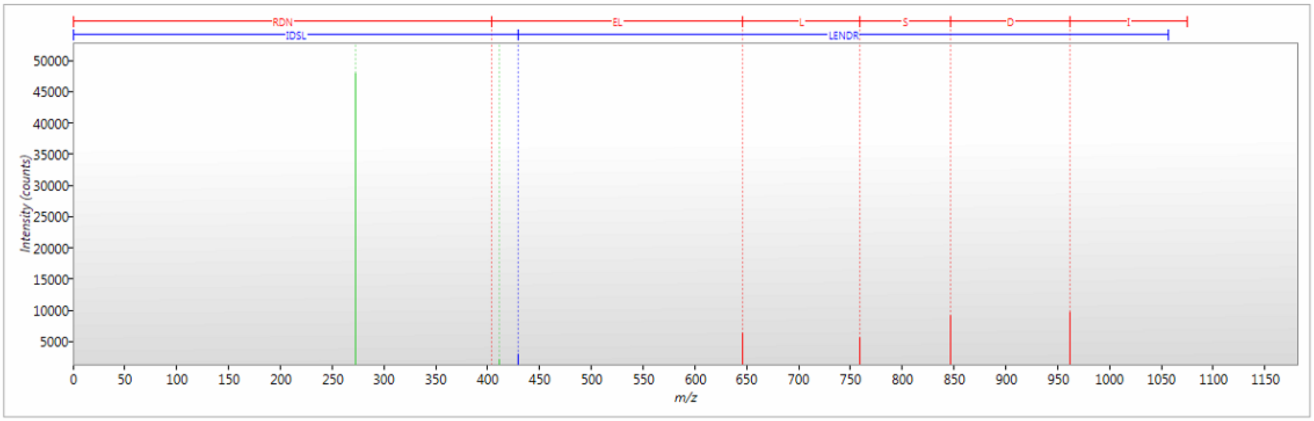

Fragment ions for: TLLSNLEEAK

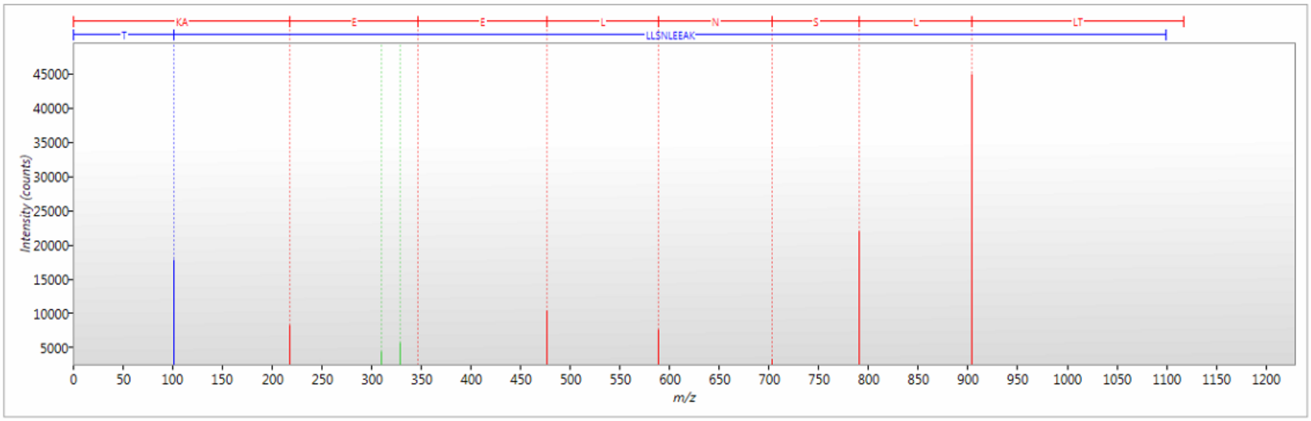

P12109

Collagen alpha-1(VI) chain OS=Homo sapiens GN=COL6A1 PE=1 SV=3
6 peptides

Sequence	Peptide Ion	Score	Hits	Mass	Charge	Tags	Conflicts	Modifications	In quantitation	Average Normalised Abundances			
										PCa	BPH	Bc	Rc
GDEGEAGDPGDDNNDIAPR	27054	5.80	2	1912.7679	2		0		yes	3699.88	1426.31	514.70	2311.34
GPPGLR	67	6.39	3	595.3453	1		0		yes	7.33e+005	2.87e+006	2.10e+006	1.30e+006
GPPGLR	777	6.39	2	595.3451	2		0		yes	8.69e+004	2.17e+005	1.73e+005	1.24e+005
LLLFSDGNSQGATPAAIEK	3617	7.87	12	1930.9723	2		1		no	3.25e+005	1.92e+005	3.01e+005	4.02e+005
MCSCCECK	11508	6.88	3	1133.3556	2		0	[2] Carbamidomethyl C | [4] Carbamidomethyl C | [5] Carbamidomethyl C | [7] Carbamidomethyl C	no	927.10	306.61	40.65	3517.94
NFTAADWGQSRDAEEAISQTIDTIVDMIK	3111	5.62	4	3224.5192	3		1		no	1.08e+006	1.38e+006	8.32e+005	9.63e+005
VFSVAITPDHLEPR	2155	7.97	13	1579.8392	3		0		yes	9.86e+004	7.71e+004	7.23e+004	1.83e+005

Fragment ions for: GDEGEAGDPGDDNNDIAPR

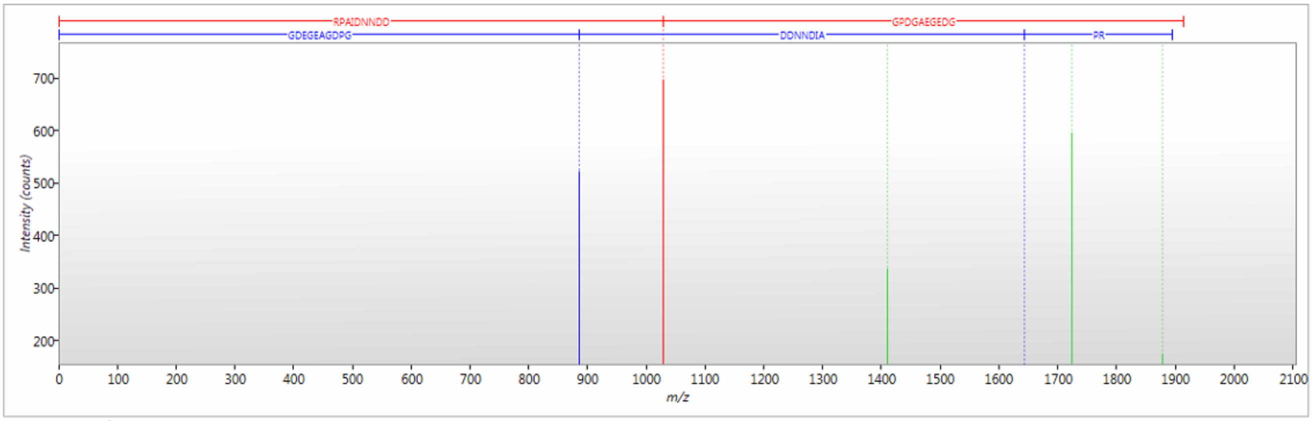

Fragment ions for: GPPGLR

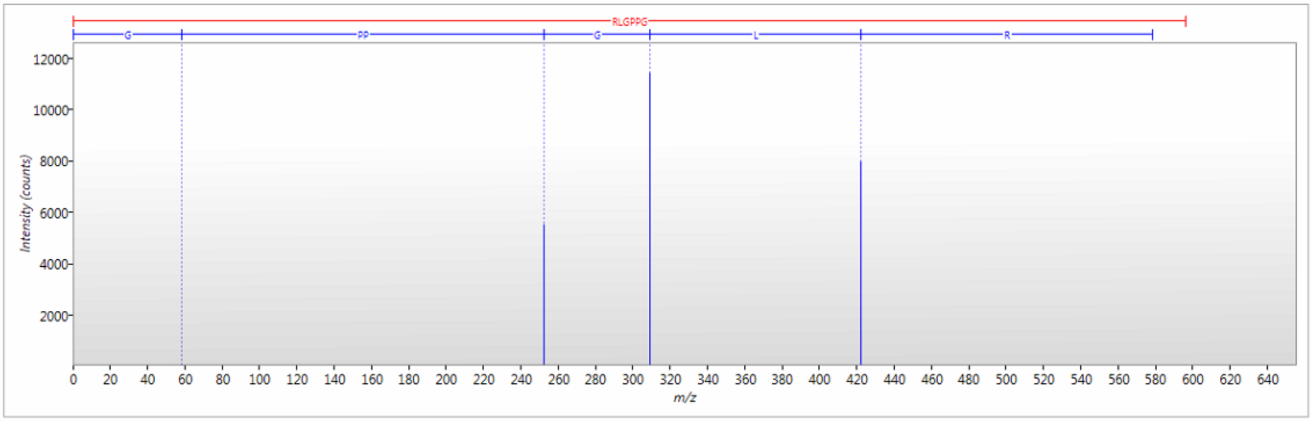

Fragment ions for: VFSVAITPDHLEPR

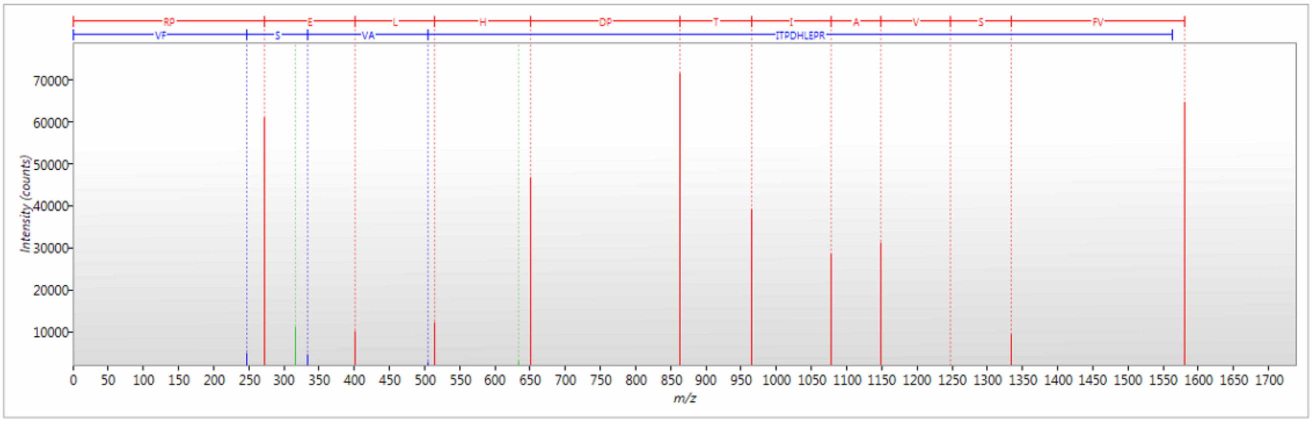

P39060

Collagen alpha-1(XVIII) chain OS=Homo sapiens GN=COL18A1 PE=1 SV=5
2 peptides

Sequence	Peptide Ion	Score	Hits	Mass	Charge	Tags	Conflicts	Modifications	In quantitation	Average Normalised Abundances			
										PCa	BPH	Bc	Rc
GLELEPGAGLFVAQAGGADPDKFQGVIAELK	5440	7.18	3	3096.6057	3		0		yes	2.26e+005	6.18e+004	3.72e+004	4.69e+005
ISEEVGLLQLLGDPPPQQVTQTDDPDVGLAYVFGPDANSGQVAR	8701	7.21	2	4605.2769	4		0		yes	4.87e+004	4454.71	1673.72	8.58e+004

Fragment ions for: GLELEPGAGLFVAQAGGADPDKFQGVIAELK

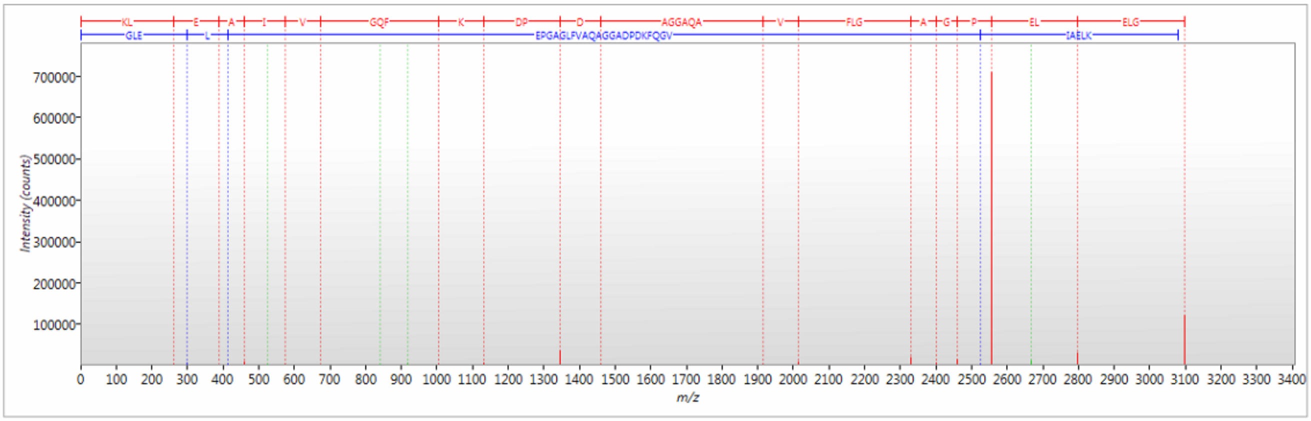

Fragment ions for: ISEEVGLLQLLGDPPPQQVTQTDDPDVGLAYVFGPDANSGQVAR

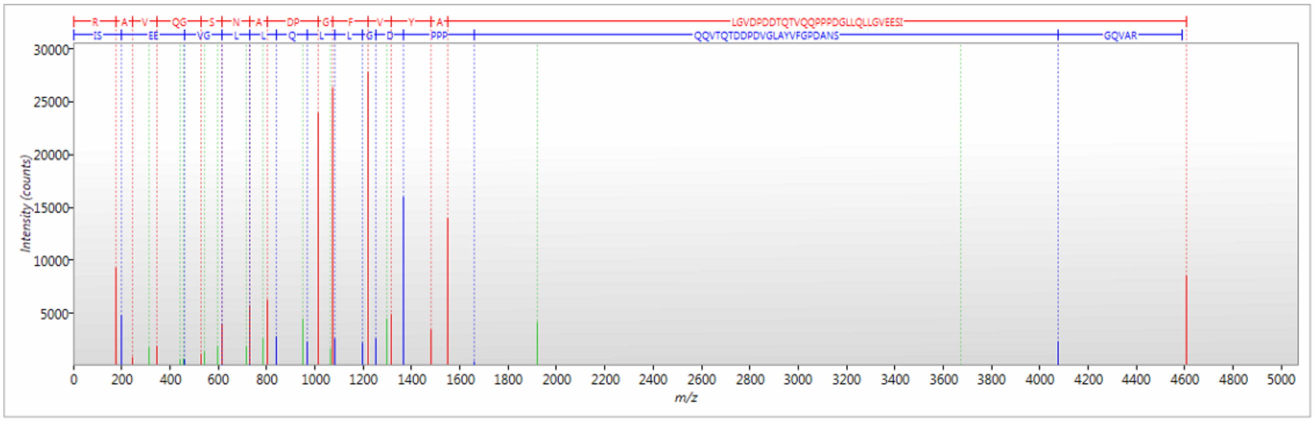

P01024

Complement C3 OS=Homo sapiens GN=C3 PE=1 SV=2
57 peptides

Sequence	Peptide Ion	Score	Hits	Mass	Charge	Tags	Conflicts	Modifications	In quantitation	Average Normalised Abundances			
										PCa	BPH	Bc	Rc
AAVYHHFISDGVR	1054	7.39	2	1470.6718	2		0		no	1.00e+005	4.63e+005	1.58e+005	2.01e+005
ACEPGVDYVYK	23229	8.39	8	1299.5776	1		0	[2] Carbamidomethyl C	no	2263.11	702.30	1.34e+004	403.57
ACEPGVDYVYK	2075	8.39	12	1299.5845	2		0	[2] Carbamidomethyl C	no	2.08e+005	3.60e+005	3.24e+005	1.36e+005
ADIGCTPGSGK	12236	8.51	15	1061.4852	1		0	[5] Carbamidomethyl C	no	5382.78	6817.20	1.47e+004	5624.23
ADIGCTPGSGK	26271	8.92	3	1061.4981	1		0	[5] Carbamidomethyl C	no	3729.79	2061.72	8840.33	1056.67
ADIGCTPGSGK	1819	8.92	20	1061.5094	2		0	[5] Carbamidomethyl C	no	2.42e+005	3.36e+005	3.02e+005	1.84e+005
AEDLVGK	2794	7.86	5	730.3692	2		0		no	3.46e+004	4.39e+004	3.32e+004	2.63e+004
ASHLGLAR	7339	7.08	8	823.3427	1		0		no	8.55e+004	3.47e+004	4.55e+004	6.13e+004
ASHLGLAR	22492	8.50	5	823.4525	1		0		no	145.83	167.71	4266.05	222.30
AYYENSPQQVFSTEFEVK	3486	8.83	16	2164.9962	2		0		no	5.19e+004	2.59e+005	3.03e+005	7.70e+004
CAEENCFIQK	1496	8.07	18	1297.5481	2		0	[1] Carbamidomethyl C|[6] Carbamidomethyl C	no	7.46e+004	1.52e+005	1.05e+005	4.06e+004
CAEENCFIQK	21137	8.07	17	1297.5408	1		0	[1] Carbamidomethyl C|[6] Carbamidomethyl C	no	245.10	65.53	5461.23	221.13
DFDFVPPVVR	14369	7.84	7	1189.6088	1		0		no	2.04e+004	1.05e+004	2.26e+004	1.10e+004
DICEEQVNSLPGSITK	2695	9.16	21	1788.8524	2		0	[3] Carbamidomethyl C	no	2.66e+005	4.13e+005	4.23e+005	2.40e+005
DTWVEHWPEEDECQDEENQK	14299	8.75	14	2602.0218	2		0	[13] Carbamidomethyl C	no	369.72	2.98e+004	2.85e+004	3289.87
DTWVEHWPEEDECQDEENQK	3195	8.75	18	2602.1485	3		0	[13] Carbamidomethyl C	no	2.63e+005	5.05e+005	3.32e+005	2.65e+005
DYAGVFSDAGLTFTSSSGQQTAQR	23139	7.21	4	2493.1010	2		0		no	9353.25	1184.98	9452.53	2939.02
EALKLEEK	2848	7.68	10	958.5011	2		0		no	9.57e+004	1.40e+005	1.35e+005	9.60e+004
EALKLEEK	22260	7.68	8	958.5123	1		0		no	872.20	1711.90	5876.29	821.42
ENEGFTVTAEGK	4229	7.34	2	1280.5991	2		0		no	2.09e+005	2.51e+005	2.22e+005	2.37e+005
ENEGFTVTAEGKGQGTLSVVTMYHAK	132	6.31	2	2769.3238	3		0	[22] Oxidation M	yes	5.42e+006	1.35e+006	1.85e+005	7.51e+006
EPGQDLVVLPLSITTDFIPSFR	7403	9.32	16	2443.2950	2		0		no	1.45e+004	6.71e+004	8.36e+004	7650.72
EPGQDLVVLPLSITTDFIPSFR	10044	9.22	8	2443.2961	2		0		no	4294.40	1.08e+004	9.48e+004	2464.64
EVVADSVWVDVK	13998	---	---	1344.7099	1		0		yes	436.24	116.53	2.39e+004	30.84
EVVADSVWVDVK	376	5.59	4	1344.6822	2		0		yes	4.85e+005	1.58e+006	2.06e+006	4.50e+005
EYVLPSFEVIVEPTEK	3732	9.27	5	1877.9616	2		0		no	6.32e+004	5.21e+004	2.33e+005	4.14e+004
FISLGEACK	990	8.33	19	1023.5139	2		0	[8] Carbamidomethyl C	no	1.95e+005	4.03e+005	2.22e+005	1.64e+005
FLTTAK	3715	---	---	679.3210	2		0		no	3.50e+004	4.01e+004	2.07e+004	3.16e+004
FLTTAK	1617	7.43	4	679.3094	1		0		no	3.94e+005	3.23e+005	3.35e+005	3.67e+005
FYHPEKEDGK	4678	7.62	2	1248.5856	3		0		no	6571.43	7655.15	1.46e+004	6498.34
FYYIYNEK	16820	8.24	10	1138.5271	1		0		no	9619.41	3972.55	1.25e+004	5521.69
GQGTLSVVTMYHAK	4736	5.17	2	1490.7028	2		0		no	2.00e+005	1.92e+005	1.14e+005	1.67e+005
ILLQGTPVAQMTEDAVDAER	9525	9.03	2	2156.0803	3		0		no	1510.54	1386.59	9.23e+004	2399.86
ILLQGTPVAQMTEDAVDAERLK	7046	7.59	3	2397.2055	3		0		no	2.52e+005	2.12e+005	2.90e+005	3.52e+005
ISLPESLK	3282	7.92	8	885.5116	2		0		no	5.75e+004	1.17e+005	1.10e+005	3.79e+004
IWDVVEK	1093	7.88	13	887.4759	2		0		no	8.31e+004	2.13e+005	2.10e+005	7.06e+004
KQELSEAEQATR	3117	8.51	22	1388.6509	2		0		no	1.80e+005	2.01e+005	2.11e+005	1.66e+005
KVLLDGVQNPR	5976	7.18	2	1237.6135	3		0		no	1.14e+004	1.56e+004	4.80e+004	1.16e+004
LCRDELCR	11136	6.95	4	1120.5147	3		0	[2] Carbamidomethyl C|[7] Carbamidomethyl C	no	4547.84	6158.24	1797.00	1347.70
LMNIFLK	5510	8.03	3	877.5089	2		0		no	1586.40	7259.74	1.81e+004	2815.48
LPYSVVR	435	8.08	3	832.3271	1		0		no	5.81e+005	5.68e+005	5.53e+005	5.90e+005
LVAYYTLIGASGQR	12615	8.24	11	1510.9001	3		0		no	2.33e+004	4.09e+004	1.09e+004	1.20e+004
NTMILEICTR	32337	7.80	4	1249.5955	1		0	[8] Carbamidomethyl C	no	3250.82	275.66	6679.33	2882.33
QDSLSSQNQLGVLPLSWDIPELVNMGQWK	16508	7.34	5	3297.6236	3		0	[25] Oxidation M	no	2093.24	2.57e+004	1.27e+004	4245.89

QGALELIK	8478	7.97	6	870.5048	1		0		no	6822.77	1.06e+004	1.81e+004	5642.67
QGALELIK	2381	7.97	7	870.4782	2		0		no	8.81e+004	1.27e+005	7.87e+004	8.81e+004
QKPDGVFQEDAPVIHQEMIGGLR	3425	6.21	2	2579.2966	3		0	[18] Oxidation M	yes	1.57e+006	5.23e+005	2.85e+005	8.02e+005
QVREPGQDLVVLPLSITTDFIPSFR	25694	8.11	6	2826.5242	3		0		no	5360.94	3219.16	1.93e+004	1617.50
SDDKVTLEER	4966	7.56	2	1190.4161	2		0		no	3.64e+004	3.11e+004	4.79e+004	3.80e+004
SDDKVTLEER	4627	8.26	18	1190.5777	3		0		no	5.56e+004	1.03e+005	5.83e+004	4.54e+004
SGIPIVTSPYQIHFTK	2097	8.95	22	1786.9619	3		0		no	2.41e+005	4.14e+005	2.04e+005	1.28e+005
SGQSEDRQPVPGQQMTLK	15449	7.97	13	1984.9255	2		0		no	7766.89	1.48e+004	2.98e+004	8789.05
SGSDEVQVGQQR	29356	8.57	22	1288.5991	1		0		no	1689.48	2566.39	7107.69	368.79
SNLDEDIIAEENIVSR	2669	9.21	20	1815.8837	2		0		no	4.73e+005	5.84e+005	4.19e+005	4.94e+005
SNLDEDIIAEENIVSRSEFPESWLWNVEDLK	11194	6.28	4	3675.5742	3		0		no	4.35e+005	1.07e+005	1.77e+005	1.27e+005
SSLSVPYVIVPLK	42222	8.93	6	1400.8237	1		0		no	19.77	0.00	1805.09	1.64
TELRPGETLNVNFLLR	2171	8.57	6	1871.0219	3		0		no	1.45e+005	1.82e+005	1.92e+005	1.38e+005
TFISPIK	2521	8.26	23	804.4666	2		0		no	5.14e+004	1.02e+005	7.25e+004	5.93e+004
TGLQEVEVK	1428	8.24	15	1001.5355	2		0		no	2.49e+005	4.81e+005	3.67e+005	2.86e+005
TGLQEVEVK	20706	7.70	9	1001.5253	1		0		no	1372.96	3497.63	2018.21	1146.75
TIYTPGSTVLYR	28024	8.91	12	1369.7248	1		0		no	54.58	211.94	5721.02	112.55
TLDPER	3026	7.44	2	729.3369	1		0		no	1.05e+005	8.90e+004	1.26e+005	8.11e+004
TMQALPYSTVGNSNNYLHLSVLR	8253	7.28	5	2577.2864	3		0		no	5.39e+004	1.14e+005	3.08e+004	6.36e+004
TMQALPYSTVGNSNNYLHLSVLR	14122	8.22	6	2577.1258	2		0		no	6875.11	8763.10	2.81e+004	1.39e+004
VFLDCCNYITELR	1596	9.10	18	1701.7858	2		0	[5] Carbamidomethyl C|[6] Carbamidomethyl C	no	5.89e+005	5.85e+005	5.82e+005	4.12e+005
VHQYFNVELIQPGAVK	8988	9.14	9	1840.8846	2		0		no	6.72e+004	9.18e+004	1.22e+005	4.45e+004
VHQYFNVELIQPGAVK	7824	7.77	3	1840.9197	3		0		no	3.64e+004	1.62e+005	5.52e+004	4.66e+004
VLLDGVQNPR	2238	8.23	13	1109.5916	2		0		no	1.38e+005	1.83e+005	1.97e+005	1.08e+005
VLLDGVQNPR	32669	8.23	10	1109.5973	1		0		no	648.26	1315.82	3549.55	459.40
VQLSNDFDEYIMAIEQTIK	11629	9.12	15	2256.0959	2		0		no	2.70e+004	4.44e+004	7.00e+004	1.93e+004
VTIKPAPETEKRPQDAK	20668	6.82	5	1907.0749	3		0		no	2444.10	1.67e+004	2173.76	985.12
VVLVAVDK	10461	8.59	15	841.5103	1		0		no	1049.06	3533.92	2.28e+004	1706.13
VVPEGIR	4638	8.34	3	768.4349	2		0		no	2.81e+004	2.49e+004	2.74e+004	1.95e+004

Fragment ions for: ENEGFTVTAEGKGQGTLSVVTMYHAK

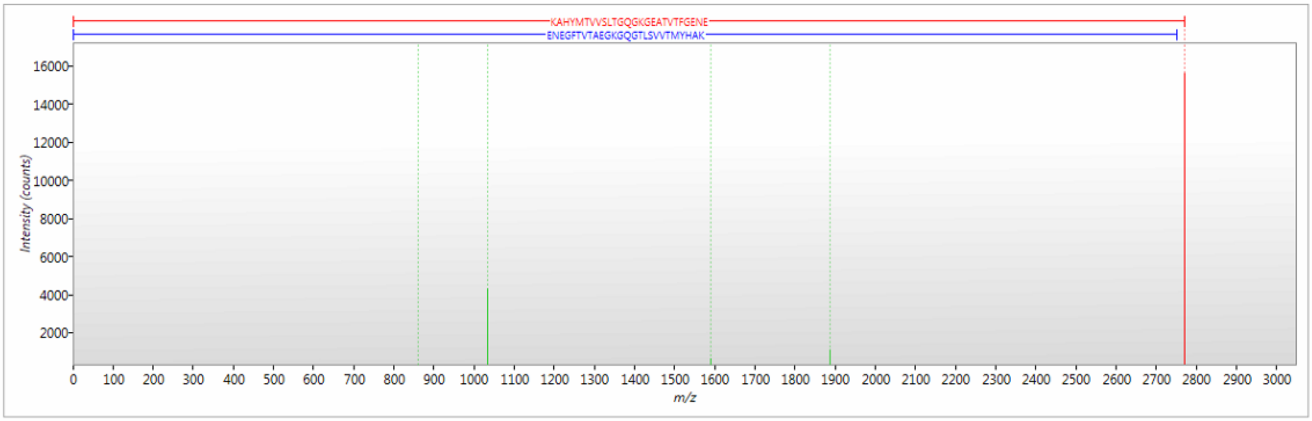

Fragment ions for: DICEEQVNSLPGSITK

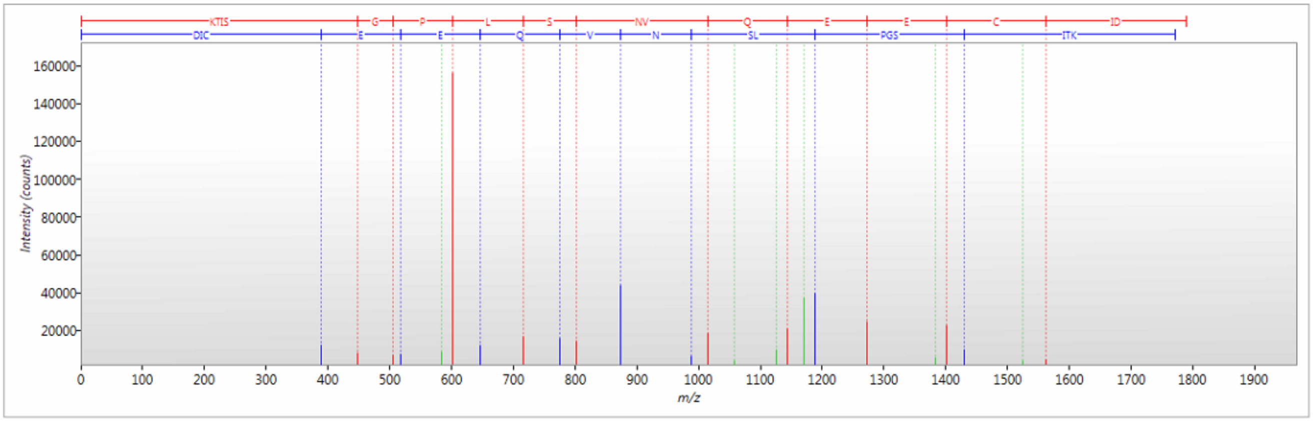

Fragment ions for: EPGQDLVVLPLSITTDFIPSFR

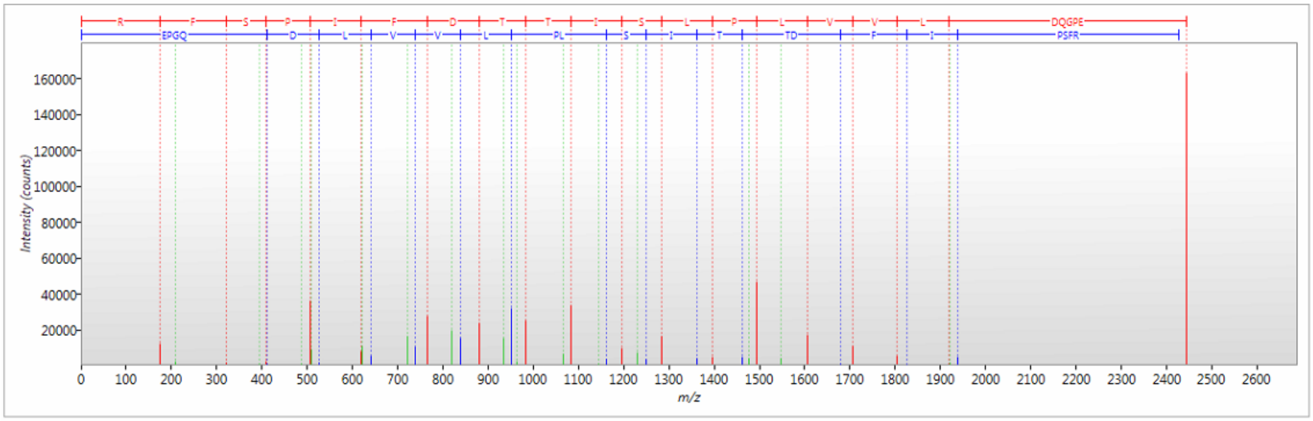

Fragment ions for: EYVLPSFEVIVEPTEK

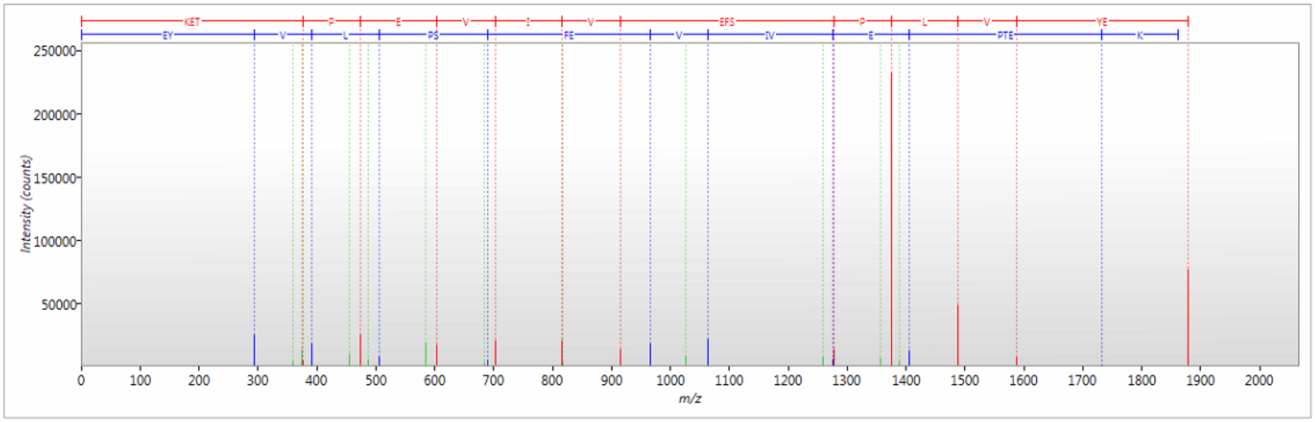

Fragment ions for: QKPDGVFQEDAPVIHQEMIGGLR

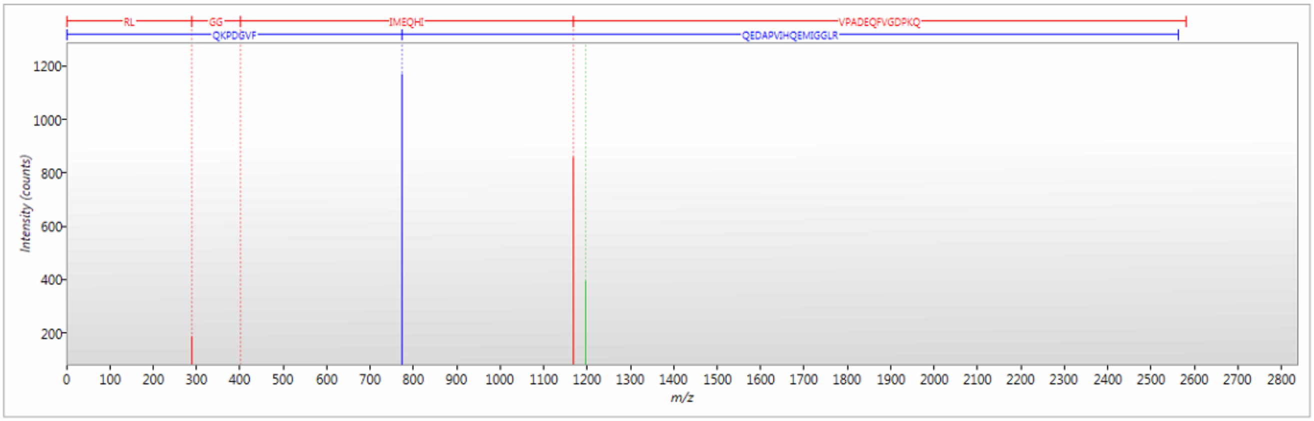

P24855

Deoxyribonuclease-1 OS=Homo sapiens GN=DNASE1 PE=1 SV=1
3 peptides

Sequence	Peptide Ion	Score	Hits	Mass	Charge	Tags	Conflicts	Modifications	In quantitation	Average Normalised Abundances			
										PCa	BPH	Bc	Rc
IAAFNIQTFGETK	10667	7.17	3	1438.7837	3		0		yes	1379.25	7420.64	3645.24	2.84e+004
LLDNLNQDAPDTYHYVVSEPLGR	1234	8.17	18	2628.2673	3		0		yes	7.03e+005	2.72e+005	1.97e+005	7.71e+005
LWTSPTFQWLIPDSADTTATPTHCAYDR	7771	8.27	10	3250.5177	3		0	[24] Carbamidomethyl C	yes	1.49e+005	8.81e+004	3.51e+004	3.45e+005

Fragment ions for: IAAFNIQTFGETK

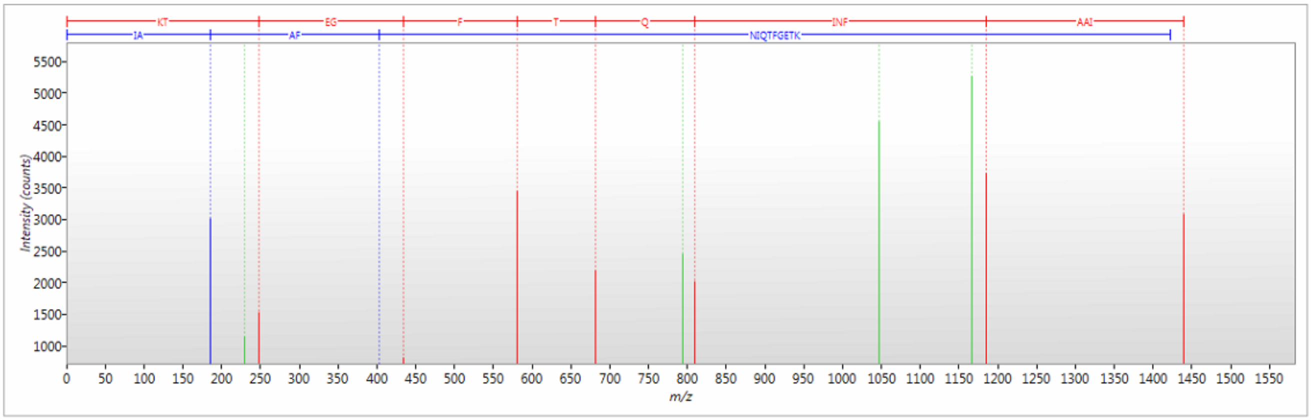

Fragment ions for: LLDNLNQDAPDTYHYVVSEPLGR

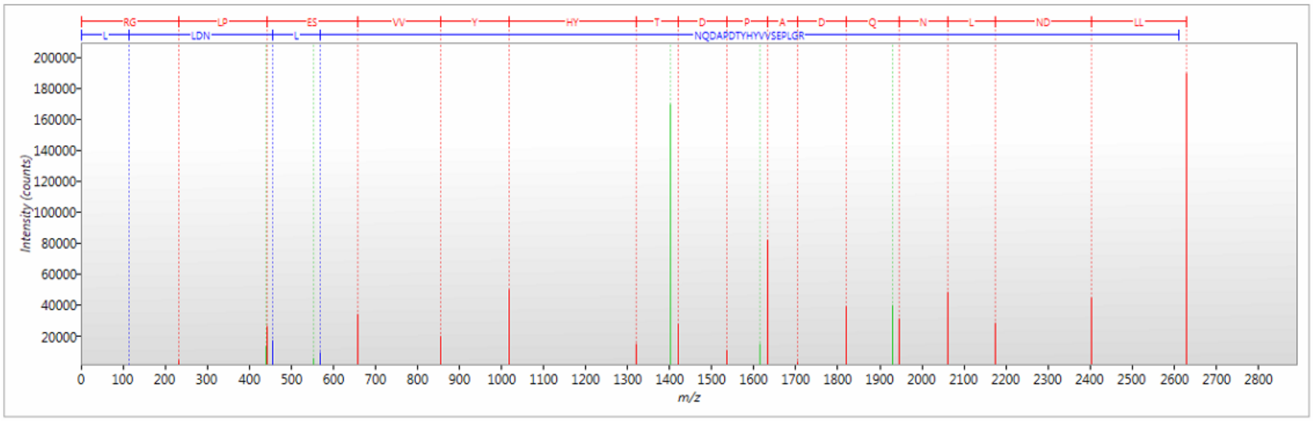

Fragment ions for: LWTSPTFQWLIPDSADTTATPTHCAYDR

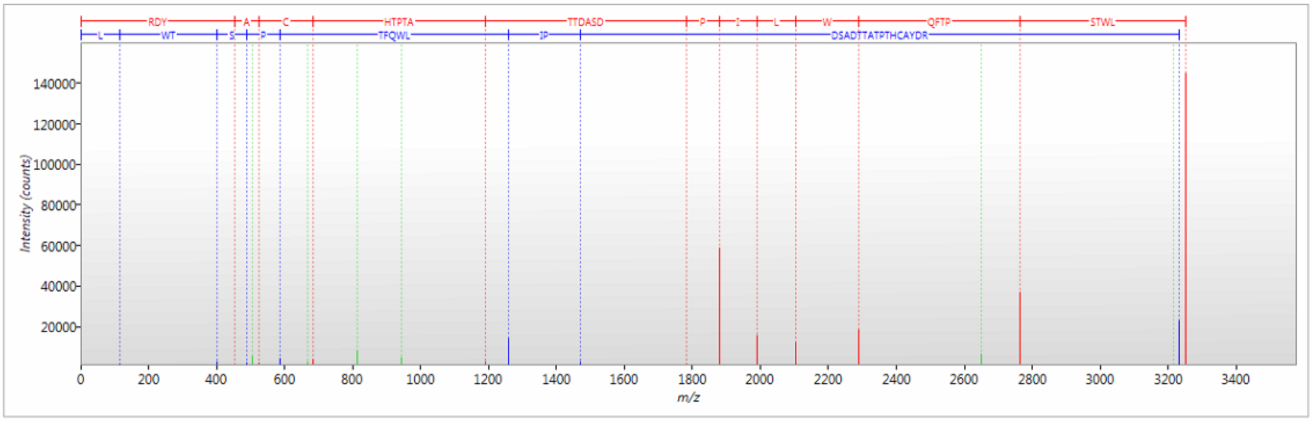

O94919

Endonuclease domain-containing 1 protein OS=Homo sapiens GN=ENDOD1 PE=1 SV=2
3 peptides

Sequence	Peptide Ion	Score	Hits	Mass	Charge	Tags	Conflicts	Modifications	In quantitation	Average Normalised Abundances			
										PCa	BPH	Bc	Rc
ILEVVNQIQDEER	4000	8.04	12	1583.8087	2		0		yes	1.01e+005	5.47e+004	8.11e+004	9.50e+004
QALNTDYLDSDYQR	6468	8.26	17	1700.7903	2		0		yes	9.96e+004	5.80e+004	4.14e+004	8.25e+004
WLVEPQIDDPNSNLEEAINEAEAITSVNSLGSK	7639	8.21	7	3581.7292	3		0		yes	1.71e+005	2.12e+004	2606.47	7.35e+004

Fragment ions for: ILEVVNQIQDEER

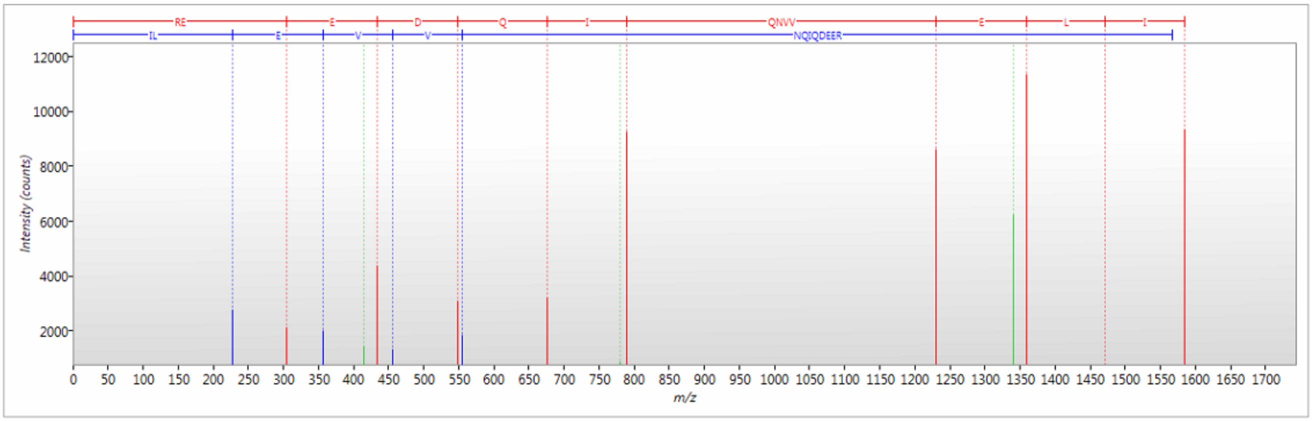

Fragment ions for: QALNTDYLDSDYQR

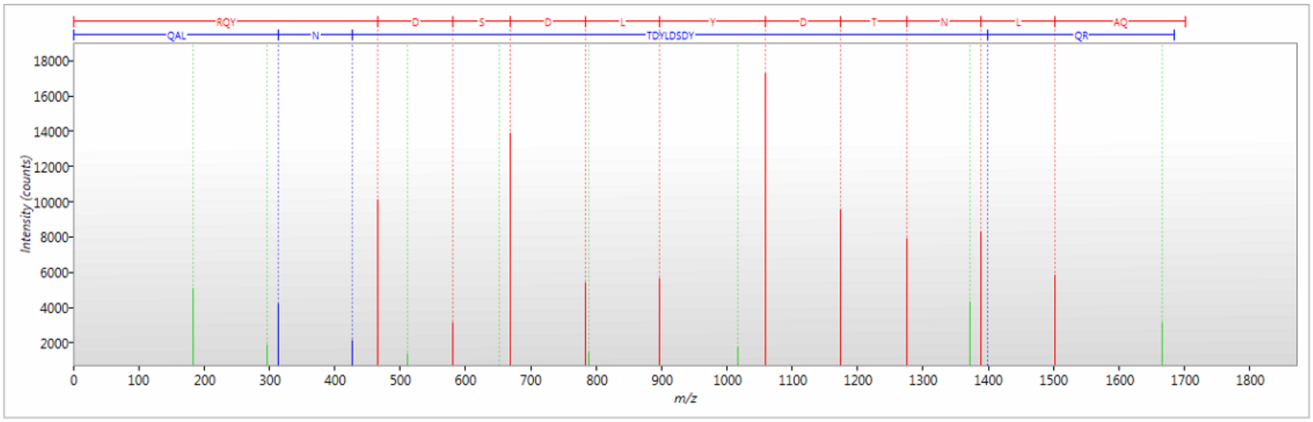

Fragment ions for: WLVEPQIDDPNSNLEEAINEAEAITSVNSLGSK

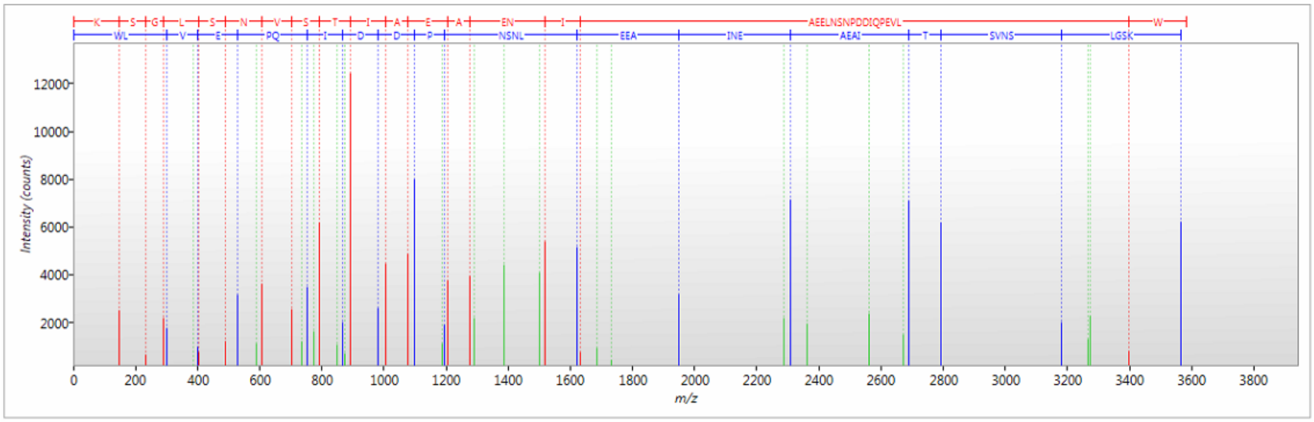

P02675

Fibrinogen beta chain OS=Homo sapiens GN=FGB PE=1 SV=2
24 peptides

Sequence	Peptide Ion	Score	Hits	Mass	Charge	Tags	Conflicts	Modifications	In quantitation	Average Normalised Abundances			
										PCa	BPH	Bc	Rc
AHYGGFTVQNEANK	26759	---	---	1534.7131	1		0		no	0.00	0.00	6736.81	5.67
AHYGGFTVQNEANK	6345	9.11	9	1534.7004	2		0		no	1.60e+005	3.12e+005	1.91e+005	1.82e+005
AHYGGFTVQNEANK	5298	8.45	6	1534.7356	3		0		no	8.09e+004	1.72e+005	4.90e+004	3.71e+004
AHYGGFTVQNEANK	2963	9.39	9	1534.7014	2		0		no	5.57e+004	6.02e+004	3.91e+005	6.27e+004
AHYGGFTVQNEANK	2609	9.39	9	1534.7267	3		0		no	3.55e+004	7.45e+004	1.68e+005	3.76e+004
DNDGWLTSDPR	23995	8.56	9	1274.5567	1		0		no	846.34	229.50	7127.18	3503.90
DNENVVNEYSSELEK	40786	9.65	8	1767.7655	1		0		no	0.00	0.00	5031.72	0.00
EDGGGWWYNR	25203	8.98	3	1238.5151	1		0		no	3089.72	409.98	7879.93	677.43
EDGGGWWYNR	19106	9.24	17	1238.5177	1		0		no	5616.09	7282.33	1.91e+004	3633.92
EDGGGWWYNR	1331	9.24	13	1238.5206	2		0		no	1.04e+005	1.55e+005	4.08e+005	6.40e+004
EDGGGWWYNR	1203	9.16	10	1238.5543	2		0		no	2.03e+005	5.41e+005	4.24e+005	1.95e+005
EDGGGWWYNRCHAANPNGR	6398	6.48	2	2215.9327	2		0	[11] Carbamidomethyl C	no	2.29e+004	6.69e+004	1.42e+005	2.02e+004
GHRPLDK	52290	8.53	3	821.4456	1		0		no	282.75	59.63	1343.78	335.40
GHRPLDKK	12564	7.26	2	949.5454	3		0		no	793.45	2995.09	1.06e+004	2156.56
GSWYSMRK	1650	6.34	2	1029.4818	3		0	[6] Oxidation M	no	6.13e+004	1.22e+005	7.45e+004	5.89e+004
GSWYSMRK	3738	6.78	2	1029.4780	2		0	[6] Oxidation M	no	1.56e+005	1.68e+005	1.45e+005	1.43e+005
HGTDDGVVWMNWK	7733	8.81	9	1543.7877	3		0		no	1.03e+004	5.67e+004	4.59e+004	2.70e+004
IRPFFPQQ	928	8.91	18	1031.5651	2		0		no	2.02e+005	9.36e+005	6.08e+005	2.84e+005
KAPDAGGCLHADPDLGVLCPTGCQLQEALLQQERPIR	8536	8.22	3	4083.0109	5		0	[8] Carbamidomethyl C | [19] Carbamidomethyl C | [23] Carbamidomethyl C	no	1.35e+005	1.22e+005	5.83e+004	1.64e+004
KAPDAGGCLHADPDLGVLCPTGCQLQEALLQQERPIR	7897	8.75	5	4083.0729	4		0	[8] Carbamidomethyl C | [19] Carbamidomethyl C | [23] Carbamidomethyl C	no	5.10e+004	1.18e+005	7.21e+005	7.75e+004
KAPDAGGCLHADPDLGVLCPTGCQLQEALLQQERPIR	5255	8.71	4	4083.0132	5		0	[8] Carbamidomethyl C | [19] Carbamidomethyl C | [23] Carbamidomethyl C	no	1.39e+005	1.02e+005	9.74e+005	9.98e+004
KWDPYK	2637	7.80	2	835.4123	2		0		no	2.83e+005	4.43e+005	3.36e+005	2.45e+005
LESDVSAQMEYCR	531	---	---	1602.8212	4		0	[9] Oxidation M | [12] Carbamidomethyl C	yes	1.80e+006	2.81e+006	4.22e+005	8.34e+005
LESDVSAQMEYCR	1387	8.06	2	1602.8075	2		0	[9] Oxidation M | [12] Carbamidomethyl C	yes	1.34e+006	1.39e+006	3.43e+005	9.67e+005
LESDVSAQMEYCR	185	---	---	1602.8120	3		0	[9] Oxidation M | [12] Carbamidomethyl C	yes	6.02e+006	9.79e+006	1.44e+006	3.70e+006
NYCGLPGEYWLGNDK	605	9.74	25	1784.8154	2		0	[3] Carbamidomethyl C	yes	8.15e+005	1.48e+006	1.41e+006	6.87e+005
NYCGLPGEYWLGNDK	35896	9.74	7	1784.7651	1		0	[3] Carbamidomethyl C	no	0.00	0.00	3837.26	0.00
NYCGLPGEYWLGNDKISQLTR	36967	7.64	3	2483.1826	2		0	[3] Carbamidomethyl C	no	1.03e+004	120.58	235.66	873.22
NYCGLPGEYWLGNDKISQLTR	42747	7.78	3	2483.1518	2		0	[3] Carbamidomethyl C	no	3292.94	511.01	3560.50	721.47
QCSKEDGGGWWYNR	4075	6.44	2	1741.8186	3		0	[2] Carbamidomethyl C	no	6.65e+004	2.51e+005	1.03e+005	1.35e+005
QDGSVDFGR	764	8.92	19	979.4432	2		0		no	2.08e+005	5.42e+005	4.23e+005	1.16e+005
QGFGNVATNTDGK	401	9.14	22	1307.6124	2		0		no	3.76e+005	8.56e+005	6.99e+005	2.79e+005
QGFGNVATNTDGK	12333	9.14	17	1307.6038	1		0		no	1411.14	2772.24	2.00e+004	3442.53
QGFGNVATNTDGKNYCGLPGEYWLGNDK	8604	5.67	2	3074.4099	4		0	[16] Carbamidomethyl C	no	4131.41	4.18e+005	3.92e+004	4.09e+004
QVKDNENVVNEYSSELEK	925	---	---	2123.0597	4		0		yes	1.04e+005	1.57e+005	1.17e+006	1.09e+005
QVKDNENVVNEYSSELEK	1278	8.41	6	2123.0448	3		0		yes	2.77e+005	6.30e+005	1.47e+006	1.82e+005
SILENLR	5026	8.68	9	843.4667	1		0		no	1.42e+004	1.50e+004	4.68e+004	5.71e+004
TPCTVSCNIPVVSGK	595	9.67	24	1617.7759	2		0	[3] Carbamidomethyl C | [7] Carbamidomethyl C	no	5.83e+005	1.03e+006	1.29e+006	4.64e+005
TPCTVSCNIPVVSGK	31868	9.67	9	1617.7821	1		0	[3] Carbamidomethyl C | [7] Carbamidomethyl C	no	68.51	0.00	5080.40	235.96
TPCTVSCNIPVVSGKECEEIIR	27982	9.49	12	2547.2042	2		0	[3] Carbamidomethyl C | [7] Carbamidomethyl C | [17] Carbamidomethyl C	no	7513.94	373.97	6324.94	601.46
TPCTVSCNIPVVSGKECEEIIR	2291	9.49	15	2547.2173	3		0	[3] Carbamidomethyl C | [7] Carbamidomethyl C | [17] Carbamidomethyl C	no	4.91e+005	3.66e+005	2.42e+005	1.71e+005
YQISVNK	631	8.60	15	850.4506	2		0		no	5.22e+004	3.12e+005	1.84e+005	4.10e+004

Fragment ions for: LESDVSAQMEYCR

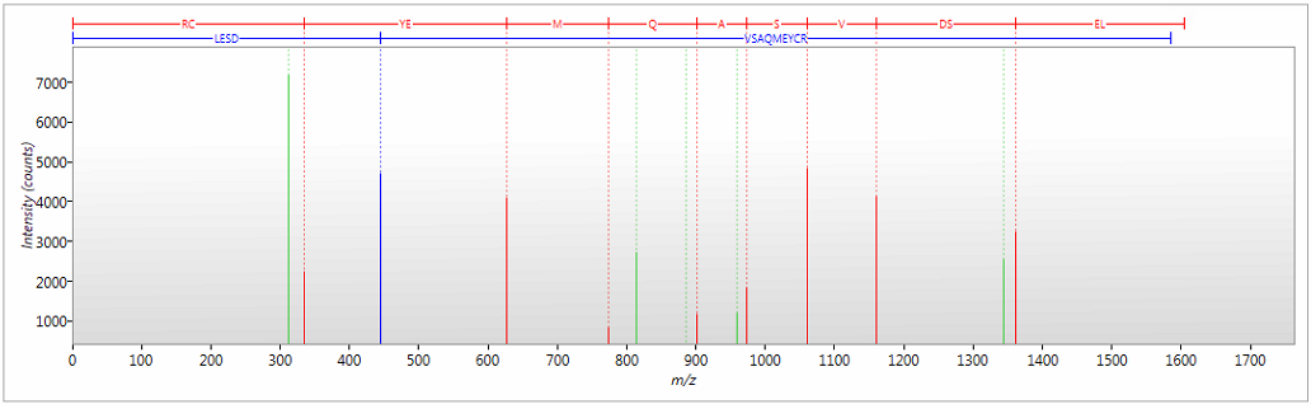

Fragment ions for: NYCGLPGEYWLGNDK

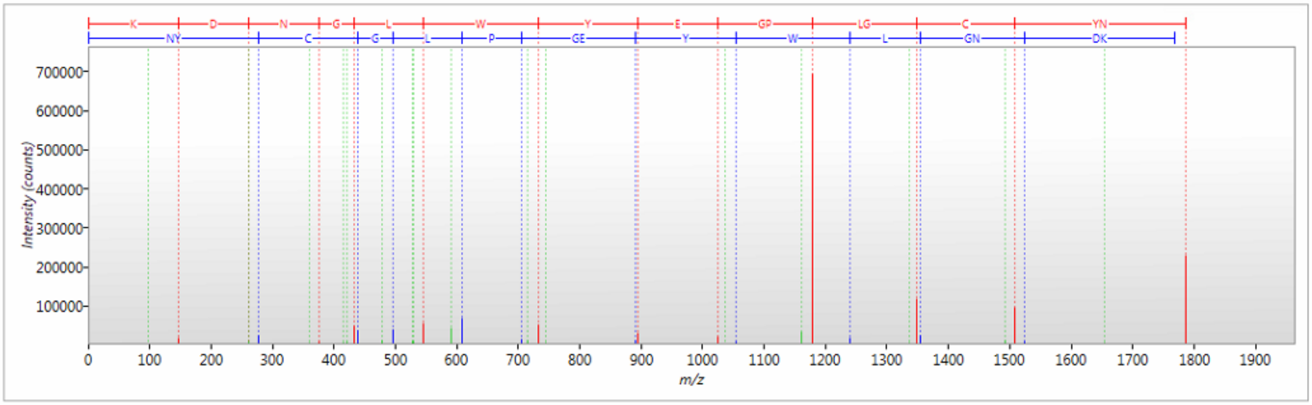

Fragment ions for: QVKDNENVVNEYSSELEK

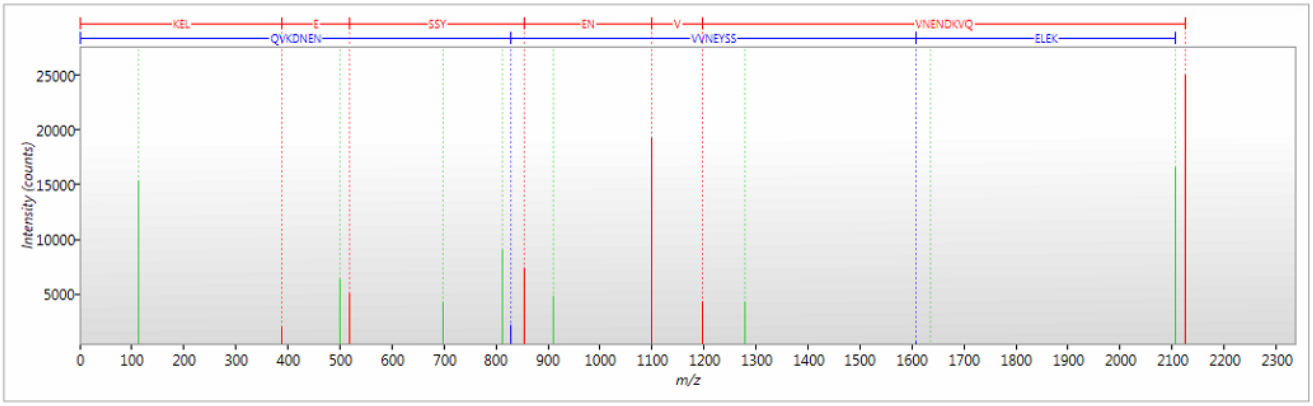

[P02679](P02679)

Fibrinogen gamma chain OS=Homo sapiens GN=FGG PE=1 SV=3
20 peptides

Sequence	Peptide Ion	Score	Hits	Mass	Charge	Tags	Conflicts	Modifications	In quantitation	Average Normalised Abundances			
										PCa	BPH	Bc	Rc
AIQLTYNPDESSKPNMIDAATLK	27009	8.97	10	2519.2055	2		0		no	3757.99	8057.03	2.56e+004	1093.73
AIQLTYNPDESSKPNMIDAATLK	14222	8.74	11	2535.2477	2		0	[16] Oxidation M	no	1460.36	7973.94	3.81e+004	3319.80
ASTPNGYDNGIIWATWK	2447	8.79	13	1892.9153	2		0		no	3.27e+005	2.49e+005	3.40e+005	3.72e+005
DCQDIANK	303	9.44	18	962.4164	2		0	[2] Carbamidomethyl C	no	9.54e+004	3.58e+005	5.86e+005	8.10e+004
DCQDIANK	4698	9.44	18	962.4138	1		0	[2] Carbamidomethyl C	no	2.05e+004	2.18e+004	5.69e+004	1.93e+004
DNCCILDER	457	9.40	23	1193.4988	2		0	[3] Carbamidomethyl C | [4] Carbamidomethyl C	yes	7.19e+005	1.05e+006	1.21e+006	5.77e+005
EGFGHLSPTGTTEFWLGNEK	587	9.50	11	2206.0559	3		0		no	5.91e+005	3.55e+005	1.94e+006	5.32e+005
EGFGHLSPTGTTEFWLGNEK	1708	9.19	11	2206.0540	3		0		no	3.42e+005	1.64e+006	4.47e+005	3.50e+005
FGSYCPTTCGIADFLSTYQTK	2135	9.45	16	2416.0723	2		0	[5] Carbamidomethyl C | [9] Carbamidomethyl C	no	5.30e+004	4.90e+005	2.51e+005	1.98e+004
FGSYCPTTCGIADFLSTYQTK	2689	9.91	5	2416.0720	2		0	[5] Carbamidomethyl C | [9] Carbamidomethyl C	no	2.24e+004	6491.52	4.93e+005	1.78e+004
FGSYCPTTCGIADFLSTYQTK	1325	9.64	17	2416.1137	3		0	[5] Carbamidomethyl C | [9] Carbamidomethyl C	no	1.63e+005	7.71e+005	5.05e+005	9.22e+004
FGSYCPTTCGIADFLSTYQTK	1942	9.91	5	2416.1202	3		0	[5] Carbamidomethyl C | [9] Carbamidomethyl C	no	4.25e+004	7.42e+004	5.82e+005	5.68e+004
IHLISTQSAIPYALR	6095	9.42	9	1681.9505	3		0		no	2774.25	1.21e+004	5.71e+004	3741.69
LTYAYFAGGDAGDAFDGFDFGDDPSDK	18948	8.99	8	2833.1673	2		0		no	946.28	5949.98	1.81e+004	80.38
LTYAYFAGGDAGDAFDGFDFGDDPSDK	20348	9.04	6	2833.1678	2		0		no	1185.79	45.13	2.61e+004	0.00
NWIQYK	2895	8.19	5	850.4384	2		0		no	4.18e+004	1.12e+005	5.82e+004	4.38e+004
QSGLYFIKPLK	3910	8.39	8	1292.7426	3		0		no	7.99e+004	2.15e+005	6.14e+004	7.75e+004
RLDGSVDFK	1145	8.42	13	1035.5264	2		0		no	2.43e+005	3.77e+005	4.84e+005	2.18e+005
RLDGSVDFK	18355	8.42	10	1035.4927	1		0		no	1.97e+004	7872.94	2.50e+004	1.73e+004
TSTADYAMFK	826	8.97	9	1133.5248	2		0		yes	1.46e+006	2.14e+006	1.20e+006	1.30e+006
TSTADYAMFK	27323	7.95	4	1149.5020	1		0	[8] Oxidation M	no	1409.49	1738.35	4734.46	349.00
VAQLEAQCQEPCK	5012	8.72	4	1559.6897	2		0	[8] Carbamidomethyl C | [12] Carbamidomethyl C	no	2.91e+004	6.25e+004	6.35e+004	3.25e+004
VAQLEAQCQEPCK	55189	8.72	2	1559.6946	1		0	[8] Carbamidomethyl C | [12] Carbamidomethyl C	no	0.00	12.43	422.33	0.00
VAQLEAQCQEPCKDTVQIHDITGK	21287	---	---	2767.3241	2		0	[8] Carbamidomethyl C | [12] Carbamidomethyl C	no	2028.62	1051.60	1.79e+004	9.35
VAQLEAQCQEPCKDTVQIHDITGK	16917	---	---	2767.3236	2		0	[8] Carbamidomethyl C | [12] Carbamidomethyl C	no	47.40	188.18	3.69e+004	6.84
VAQLEAQCQEPCKDTVQIHDITGK	3479	9.45	5	2767.3063	3		0	[8] Carbamidomethyl C | [12] Carbamidomethyl C	no	3.16e+004	1.25e+004	3.15e+005	1.65e+004
VAQLEAQCQEPCKDTVQIHDITGK	3420	9.53	16	2767.3048	3		0	[8] Carbamidomethyl C | [12] Carbamidomethyl C	no	1.39e+005	7.36e+005	4.93e+005	1.12e+005
VAQLEAQCQEPCKDTVQIHDITGK	863	9.53	22	2767.3296	4		0	[8] Carbamidomethyl C | [12] Carbamidomethyl C	yes	3.31e+005	1.89e+006	2.18e+006	3.12e+005
VAQLEAQCQEPCKDTVQIHDITGKDCQDIANK	5155	7.86	7	3711.7830	5		0	[8] Carbamidomethyl C | [12] Carbamidomethyl C | [26] Carbamidomethyl C	no	2.51e+005	2.43e+005	5.54e+004	8.91e+004
VGPEADKYR	4682	8.26	9	1033.5211	3		0		no	1.41e+004	3.57e+004	3.61e+004	9128.42
VGPEADKYR	758	8.65	13	1033.5178	2		0		no	8.27e+004	2.46e+005	2.91e+005	4.17e+004
YEASILTHDSSIR	1298	9.10	5	1490.7410	3		0		no	3.81e+004	4.46e+004	3.64e+005	1.99e+004
YEASILTHDSSIR	1083	8.93	5	1490.7375	3		0		no	4.09e+004	5.62e+004	2.17e+005	2.99e+004
YEASILTHDSSIR	3519	8.93	5	1490.7156	2		0		no	3.30e+004	3.61e+004	1.28e+005	3.65e+004
YEASILTHDSSIR	3591	9.10	5	1490.7150	2		0		no	7.10e+004	5.18e+004	1.83e+005	4.04e+004
YLQEIYNSNNQK	14027	9.65	12	1512.7190	1		0		no	1420.01	718.99	2.87e+004	572.71
YLQEIYNSNNQK	610	9.65	24	1512.7213	2		0		no	3.81e+005	1.07e+006	1.28e+006	2.56e+005

Fragment ions for: DNCCILDER

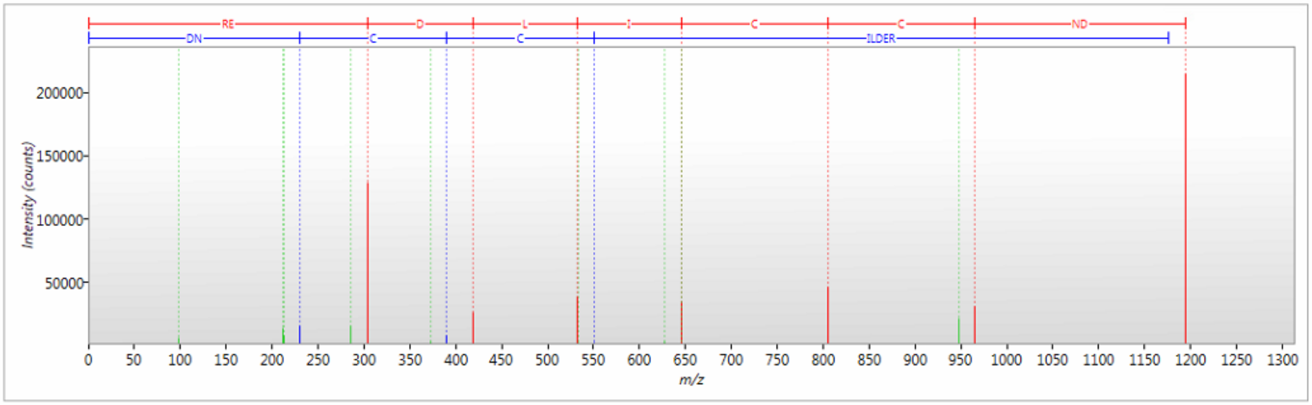

Fragment ions for: TSTADYAMFK

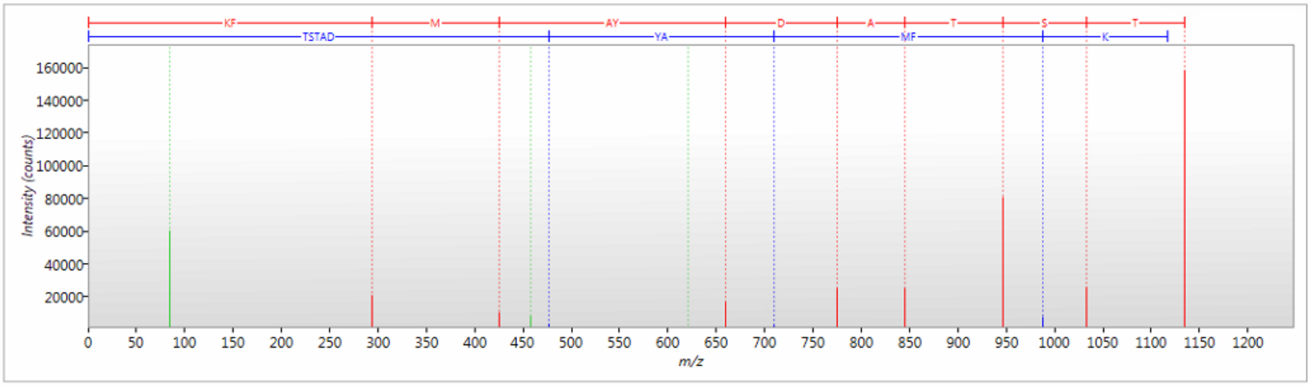

Fragment ions for: VAQLEAQCQEPCKDTVQIHDITGK

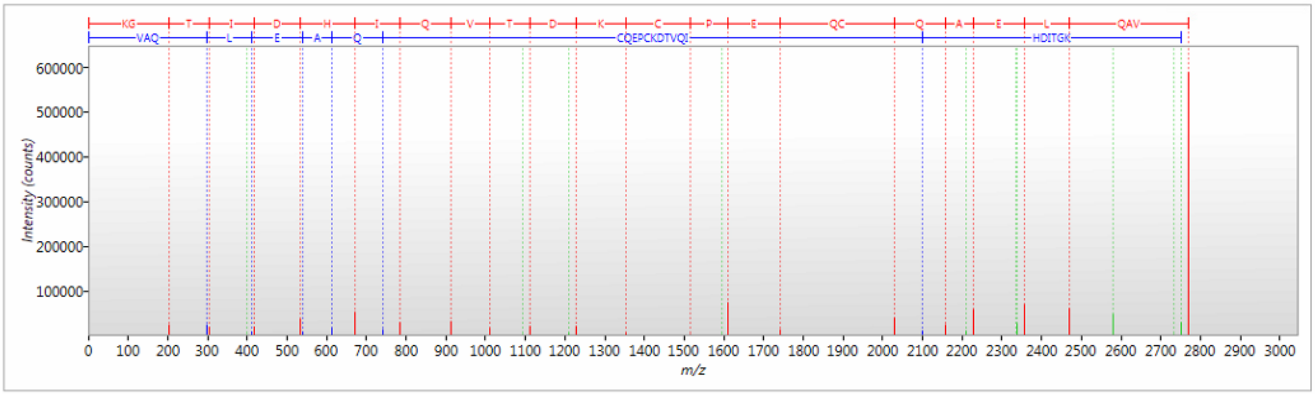

P28799

Granulins OS=Homo sapiens GN=GRN PE=1 SV=2
2 peptides

Sequence	Peptide Ion	Score	Hits	Mass	Charge	Tags	Conflicts	Modifications	In quantitation	Average Normalised Abundances			
										PCa	BPH	Bc	Rc
HCCPAGFR	632	6.29	4	1003.5125	2		0	[2] Carbamidomethyl C | [3] Carbamidomethyl C	yes	5.23e+005	6.00e+005	4.37e+005	5.97e+005
HCCPAGFR	7225	6.29	4	1003.5157	1		0	[2] Carbamidomethyl C | [3] Carbamidomethyl C	yes	2.88e+004	3.28e+004	2.83e+004	3.03e+004
QGWACCPYRQGVCCADR	618	5.55	2	2142.8618	3		0	[5] Carbamidomethyl C | [6] Carbamidomethyl C | [13] Carbamidomethyl C | [14] Carbamidomethyl C	yes	8.91e+005	5.02e+006	2.40e+006	1.55e+006

Fragment ions for: HCCPAGFR

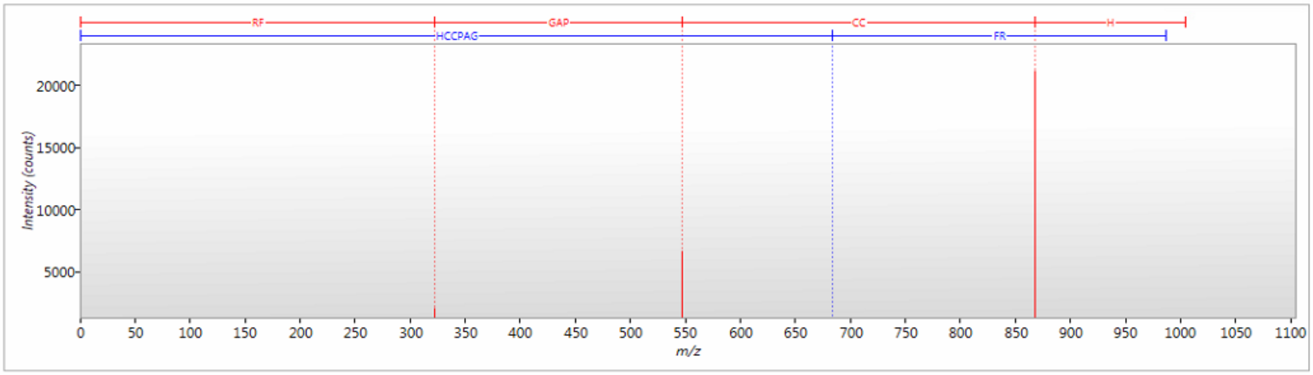

Fragment ions for: CEEEDSTALVCDNGSGLCK

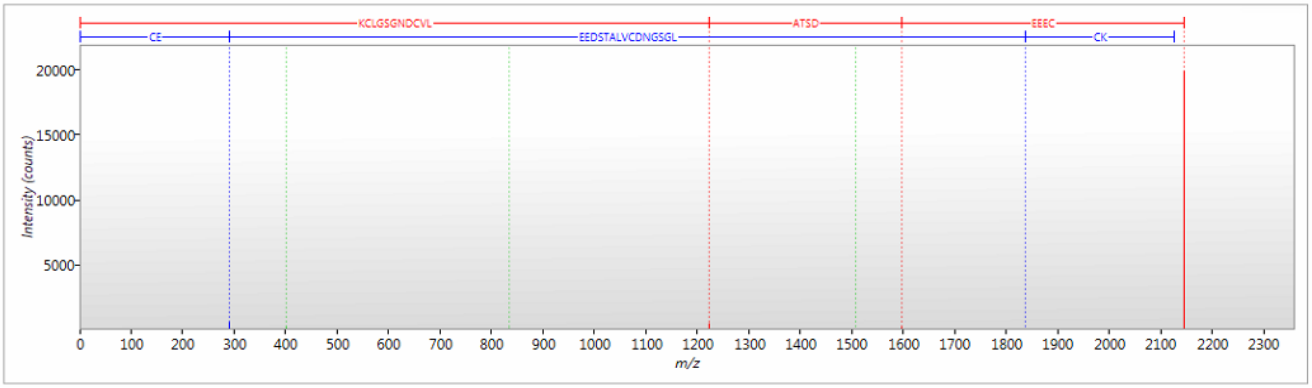

P68871

Hemoglobin subunit beta OS=Homo sapiens GN=HBB PE=1 SV=2
10 peptides

Sequence	Peptide Ion	Score	Hits	Mass	Charge	Tags	Conflicts	Modifications	In quantitation	Average Normalised Abundances			
										PCa	BPH	Bc	Rc
EFTPPVQAAYQK	994	6.84	4	1377.6885	2		0		no	1.29e+005	2.95e+005	1.99e+005	1.08e+005
EFTPPVQAAYQK	13938	6.63	3	1377.6704	1		0		no	2775.17	3851.68	1.10e+004	1410.74
FFESFGDLSTPDAVMGNPK	158	10.32	15	2058.1039	3		0		yes	1.56e+006	5.92e+006	4.88e+006	3.67e+006
FFESFGDLSTPDAVMGNPK	316	10.32	16	2057.9433	2		0		yes	3.49e+005	9.33e+005	4.79e+006	2.49e+005
FFESFGDLSTPDAVMGNPK	1251	---	---	2058.1276	2		0		yes	6.45e+005	2.01e+006	1.19e+006	7.58e+005
FFESFGDLSTPDAVMGNPK	3168	9.82	5	2074.0015	3		0	[15] Oxidation M	no	3.20e+005	4.97e+005	2.09e+005	4.56e+005
FFESFGDLSTPDAVMGNPK	1824	9.82	12	2073.9398	2		0	[15] Oxidation M	no	1.19e+005	2.48e+005	7.77e+005	1.40e+005
FFESFGDLSTPDAVMGNPK	1400	9.98	9	2073.9406	2		0	[15] Oxidation M	no	1.40e+005	1.10e+005	8.78e+005	1.22e+005
GTFATLSELHCDK	15686	9.35	4	1477.7172	3		0	[11] Carbamidomethyl C	no	3826.31	1.68e+004	2.35e+004	5117.50
GTFATLSELHCDK	1302	9.35	5	1477.7077	2		0	[11] Carbamidomethyl C	no	6.21e+005	8.82e+005	6.79e+005	6.44e+005
MVHLTPEEKSAVTALWGK	6367	6.82	2	1996.0066	3		0		no	8.20e+004	2.45e+005	1.68e+005	6.77e+004
SAVTALWGK	1700	9.87	18	931.4917	1		0		no	2.19e+005	2.36e+005	1.16e+006	2.28e+005
SAVTALWGK	4318	9.80	4	931.4826	1		0		no	9.51e+004	7.01e+004	1.24e+005	9.37e+004
VHLTPEEK	2846	9.43	10	951.4995	1		0		no	5870.63	1.11e+004	8.32e+004	4551.35
VHLTPEEK	256	9.71	5	951.5011	2		0		no	1.29e+005	4.39e+005	2.10e+006	1.19e+005
VHLTPEEK	157	9.43	10	951.5030	2		0		no	5.35e+004	1.96e+005	6.55e+005	5.29e+004
VLGAFSDGLAHLDNLK	673	9.94	13	1668.8576	3		0		yes	2.33e+005	6.89e+005	2.02e+006	2.82e+005
VNVDEVGGEALGR	3072	10.08	24	1313.6576	1		0		no	4611.70	3.43e+004	6.77e+005	6027.29
VVAGVANALAHK	6849	9.76	3	1148.6674	3		0		no	6779.42	5238.83	6.02e+004	4646.51

Fragment ions for: FFESFGDLSTPDAVMGNPK

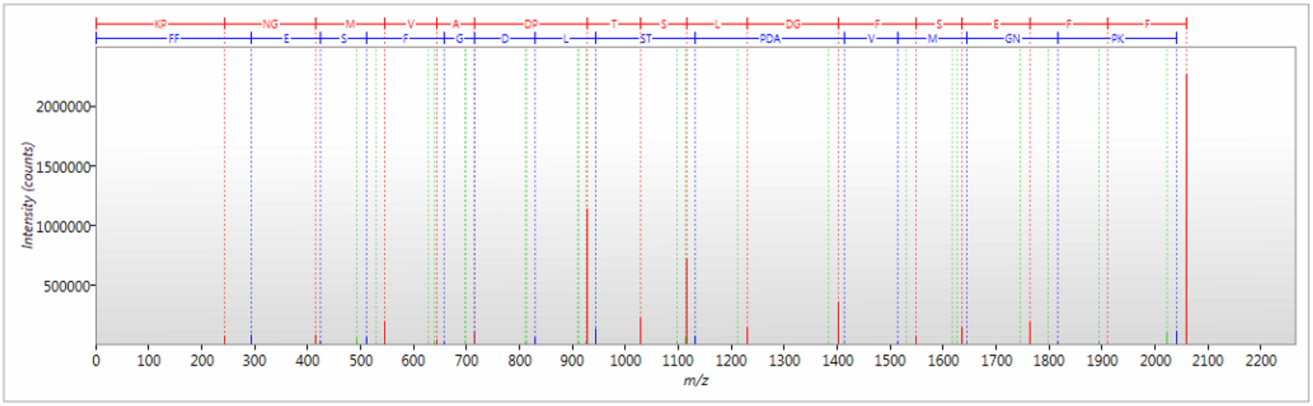

Fragment ions for: VLGAFSDGLAHLDNLK

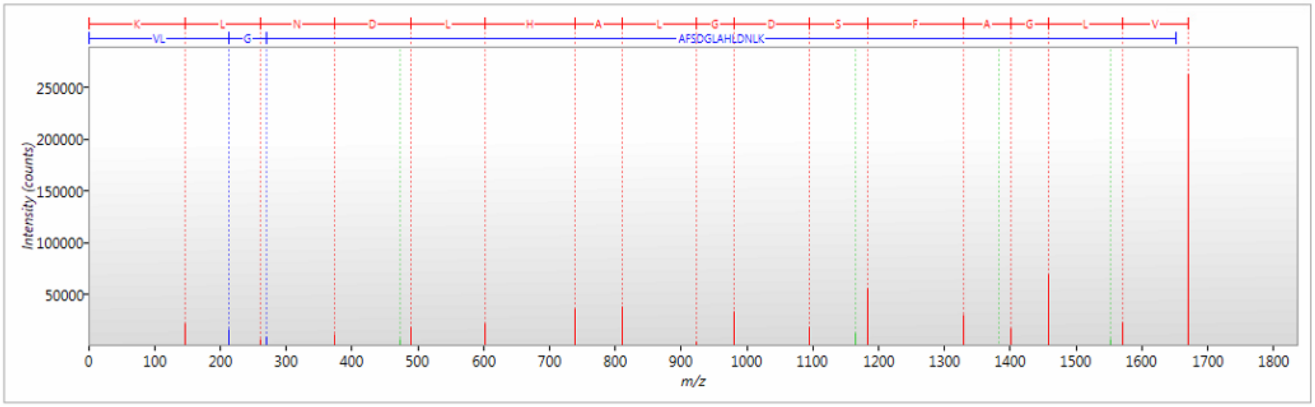

P02790

Hemopexin OS=Homo sapiens GN=HPX PE=1 SV=2
16 peptides

Sequence	Peptide Ion	Score	Hits	Mass	Charge	Tags	Conflicts	Modifications	In quantitation	Average Normalised Abundances			
										PCa	BPH	Bc	Rc
DYFMPCPGR	3126	8.50	14	1141.4899	2		0	[6] Carbamidomethyl C	no	1.30e+005	1.83e+005	9.31e+004	1.74e+005
ELISER	2354	7.83	5	745.3325	1		0		no	8.67e+004	1.27e+005	1.09e+005	8.58e+004
EVGTPHGIILDSVDAAFICPGSSR	1106	9.39	32	2497.2348	3		0	[19] Carbamidomethyl C	no	3.89e+005	9.97e+005	7.23e+005	4.25e+005
EVGTPHGIILDSVDAAFICPGSSR	2400	9.55	6	2497.2278	3		0	[19] Carbamidomethyl C	no	9.62e+004	3.84e+004	1.51e+005	4.28e+004
EVGTPHGIILDSVDAAFICPGSSR	9904	9.39	29	2497.1884	2		0	[19] Carbamidomethyl C	no	1.30e+004	5.88e+004	1.08e+005	3.04e+004
EVGTPHGIILDSVDAAFICPGSSR	16967	9.55	4	2497.2032	2		0	[19] Carbamidomethyl C	no	3192.02	6974.88	2.26e+004	4392.08
GEFVWK	6956	8.25	13	764.4027	2		0		no	1.16e+004	1.76e+004	1.19e+004	1.32e+004
GGYTLVSGYPK	11643	7.83	14	1140.5579	1		0		no	3465.98	3620.22	1.52e+004	7280.19
LLQDEFPGIPSPLDAAVECHR	739	9.39	35	2363.1595	3		0	[19] Carbamidomethyl C	yes	3.76e+005	1.52e+006	1.42e+006	5.06e+005
LLQDEFPGIPSPLDAAVECHR	8642	9.39	30	2363.1061	2		0	[19] Carbamidomethyl C	yes	1.76e+004	5.85e+004	7.84e+004	4.84e+004
LWWLDLK	4319	8.82	6	972.5371	2		0		no	1.16e+004	4.00e+004	3.20e+004	1.07e+004
NFPSPVDAAFR	359	8.52	36	1219.6052	2		0		yes	6.37e+005	1.52e+006	7.33e+005	5.55e+005
NFPSPVDAAFR	24032	8.78	2	1219.5919	1		0		no	1287.17	85.07	5659.54	1898.72
NFPSPVDAAFR	9513	8.52	33	1219.5866	1		0		yes	4.68e+004	2.63e+004	4.85e+004	4.06e+004
NFPSPVDAAFR	1793	8.78	2	1219.6012	2		0		no	1.20e+005	1.85e+005	2.64e+005	1.19e+005
QGHNSVFLIK	16115	6.73	4	1141.6258	3		0		no	1679.84	34.27	7580.48	53.81
QGHNSVFLIK	11917	7.33	7	1141.6286	3		0		no	821.83	2.47e+004	1.50e+004	4034.55
QGHNSVFLIK	12341	6.77	7	1141.6270	3		0		no	1.09e+004	1972.04	523.33	4470.92
QGHNSVFLIK	15415	7.32	3	1141.6235	3		0		no	0.00	0.00	6808.96	301.17
SGAQATWTELPWPHEK	1734	8.70	11	1836.9075	3		0		no	1.91e+005	3.12e+005	7.79e+005	1.85e+005
SLGPNSCSANGPGLYLIHGPNLYCYSDVEK	1504	7.74	19	3281.5111	3		0	[7] Carbamidomethyl C | [24] Carbamidomethyl C	yes	8.74e+005	1.64e+006	1.04e+006	8.12e+005
SLGPNSCSANGPGLYLIHGPNLYCYSDVEKLNAAK	3975	7.07	4	3778.8132	4		0	[7] Carbamidomethyl C | [24] Carbamidomethyl C	no	9.43e+005	1.72e+005	8.50e+004	5.84e+005
SLGPNSCSANGPGLYLIHGPNLYCYSDVEKLNAAK	15749	7.07	2	3778.7897	3		0	[7] Carbamidomethyl C | [24] Carbamidomethyl C	no	7.41e+004	2602.00	3356.97	3.18e+004
VDGALCMEK	7902	7.46	10	1021.4457	1		0	[6] Carbamidomethyl C	no	7.07e+004	2.68e+004	5.24e+004	7.34e+004
VDGALCMEKSLGPNSCSANGPGLYLIHGPNLYCYSDVEK	9894	6.13	3	4300.8817	3		0	[6] Carbamidomethyl C | [7] Oxidation M | [16] Carbamidomethyl C | [33] Carbamidomethyl C	no	6.23e+004	1.53e+004	1992.79	6.79e+004
VWVYPPEKK	11511	6.87	3	1144.6236	3		0		no	1.03e+004	4.44e+004	1.43e+004	5445.52
YYCFQGNQFLR	24802	9.58	20	1494.6668	1		0	[3] Carbamidomethyl C	no	286.26	3638.62	5841.89	300.43

Fragment ions for: LLQDEFPGIPSPLDAAVECHR

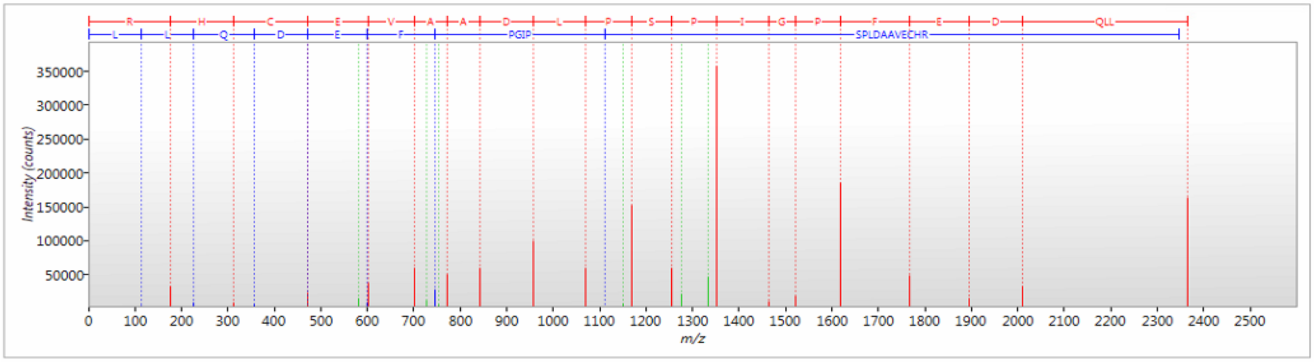

Fragment ions for: NFPSPVDAAFR

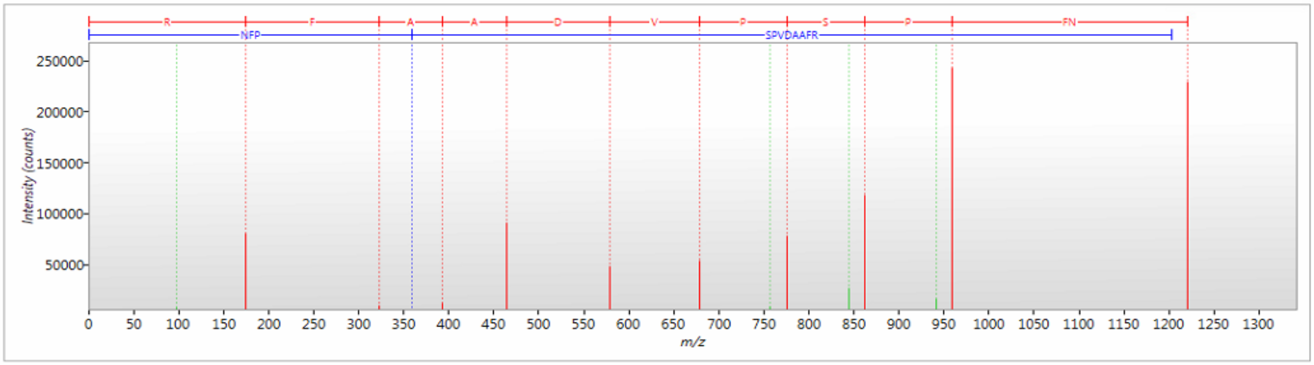

Fragment ions for: SLGPNSCSANGPGLYLIHGPNLYCYSDVEK

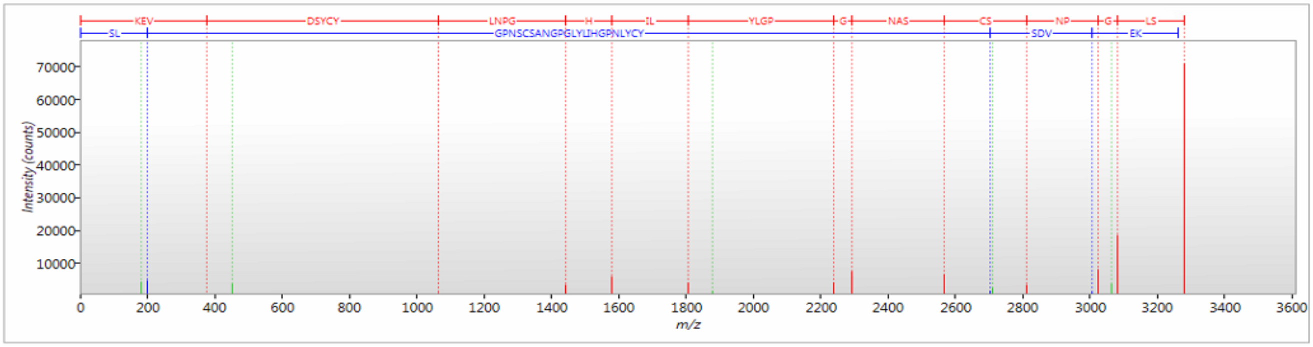

P01857

Ig gamma-1 chain C region OS=Homo sapiens GN=IGHG1 PE=1 SV=1
6 peptides

Sequence	Peptide Ion	Score	Hits	Mass	Charge	Tags	Conflicts	Modifications	In quantitation	Average Normalised Abundances			
										PCa	BPH	Bc	Rc
FNWYVDGVEVHNAK	134	9.94	38	1676.7989	3		0		yes	1.37e+006	4.91e+006	2.79e+006	1.32e+006
FNWYVDGVEVHNAKTKPR	3105	7.42	9	2159.0944	3		0		no	4.41e+005	2.21e+005	1.36e+005	3.60e+005
GPSVFPLAPSSK	120	9.74	29	1185.6400	2		0		yes	1.57e+006	4.57e+006	2.73e+006	1.93e+006
GPSVFPLAPSSK	6495	9.74	22	1185.6390	1		0		yes	3.49e+004	7.79e+004	1.01e+005	5.23e+004
THTCPPCPAPELLGGPSVFLFPPKPK	6391	9.42	18	2843.4363	3		0	[4] Carbamidomethyl C | [7] Carbamidomethyl C	no	5.99e+005	1.45e+006	2.95e+005	4.45e+005
TPEVTCVVVDVSHEDPEVK	37	10.19	40	2138.0245	3		0	[6] Carbamidomethyl C	yes	3.86e+006	1.35e+007	8.12e+006	4.32e+006
TPEVTCVVVDVSHEDPEVK	975	10.19	40	2138.0098	2		0	[6] Carbamidomethyl C	yes	5.18e+005	1.10e+006	9.47e+005	5.63e+005
TTPPVLDSDGSFFLYSK	12563	9.44	6	1872.8941	1		0		no	200.07	4984.66	5.92e+004	2035.40

Fragment ions for: FNWYVDGVEVHNAK

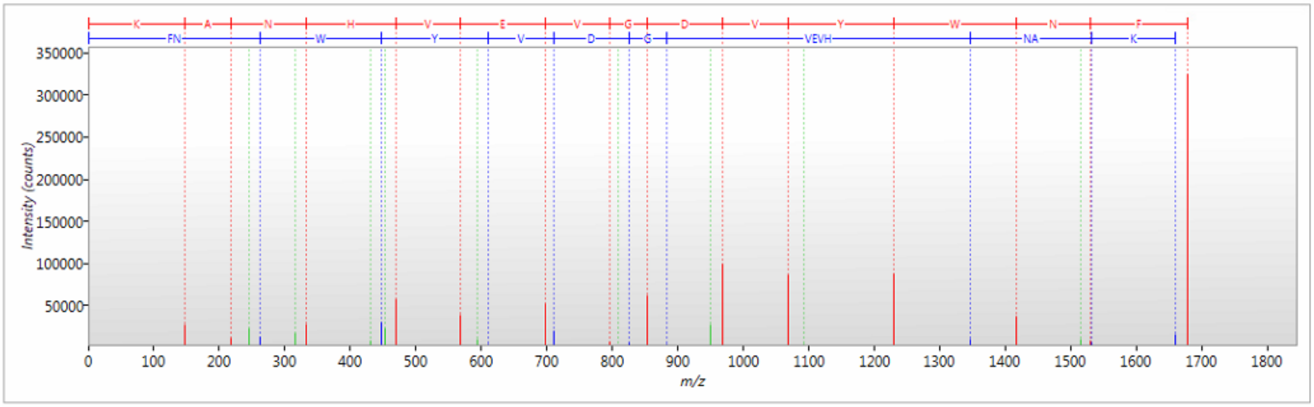

Fragment ions for: GPSVFPLAPSSK

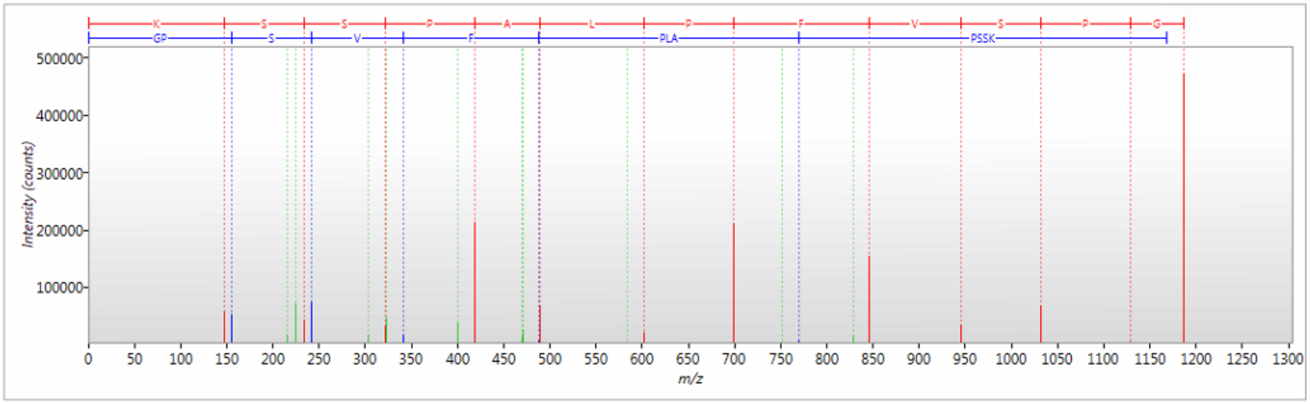

Fragment ions for: TPEVTCVVVDVSHEDPEVK

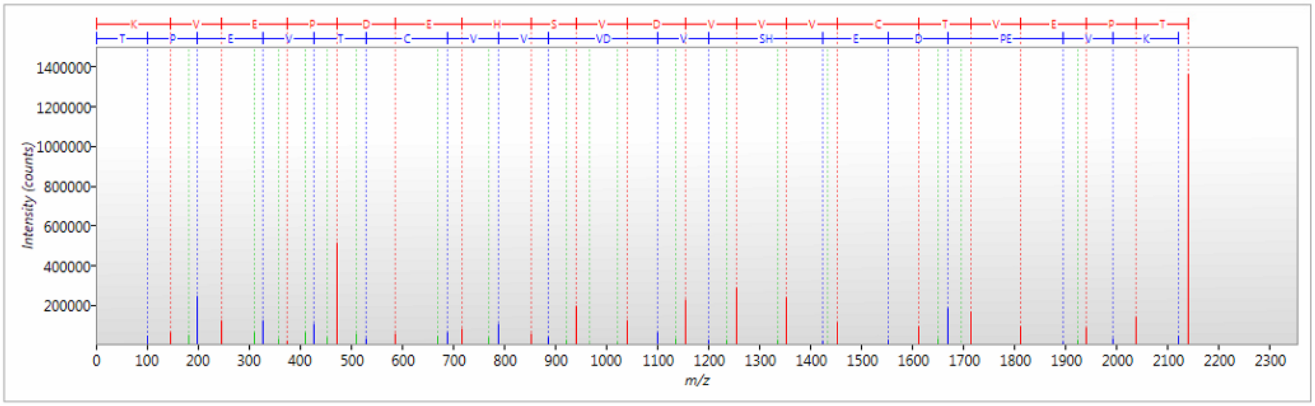

P05451

Lithostathine-1-alpha OS=Homo sapiens GN=REG1A PE=1 SV=3
5 peptides

Sequence	Peptide Ion	Score	Hits	Mass	Charge	Tags	Conflicts	Modifications	In quantitation	Average Normalised Abundances			
										PCa	BPH	Bc	Rc
DVPCEDK	8919	6.35	2	861.3525	1		0	[4] Carbamidomethyl C	yes	6.63e+004	4.49e+004	5.02e+004	5.85e+004
ISCPEGTNAYR	1730	8.51	10	1266.5851	2		1	[3] Carbamidomethyl C	no	3.43e+005	2.48e+005	1.56e+005	3.13e+005
MAQTSSYFMLISCLMFLSQSQGQEAQTELPQAR	10493	5.55	2	3780.8949	4		0	[13] Carbamidomethyl C	yes	4.39e+005	1.20e+005	1.24e+005	3.24e+005
MAQTSSYFMLISCLMFLSQSQGQEAQTELPQAR	14206	---	---	3780.9118	3		0	[13] Carbamidomethyl C	yes	2.12e+005	1.16e+004	2.95e+004	2.17e+005
SWGIGAPSSVNPGYCVSLTSSTGFQK	12052	7.62	5	2686.2267	2		0	[15] Carbamidomethyl C	yes	1.06e+005	3.11e+004	1.35e+004	6.86e+004
SYCYYFNEDR	36560	7.93	2	1415.6434	1		0	[3] Carbamidomethyl C	no	2589.29	626.70	2911.25	375.00

Fragment ions for: DVPCEDK

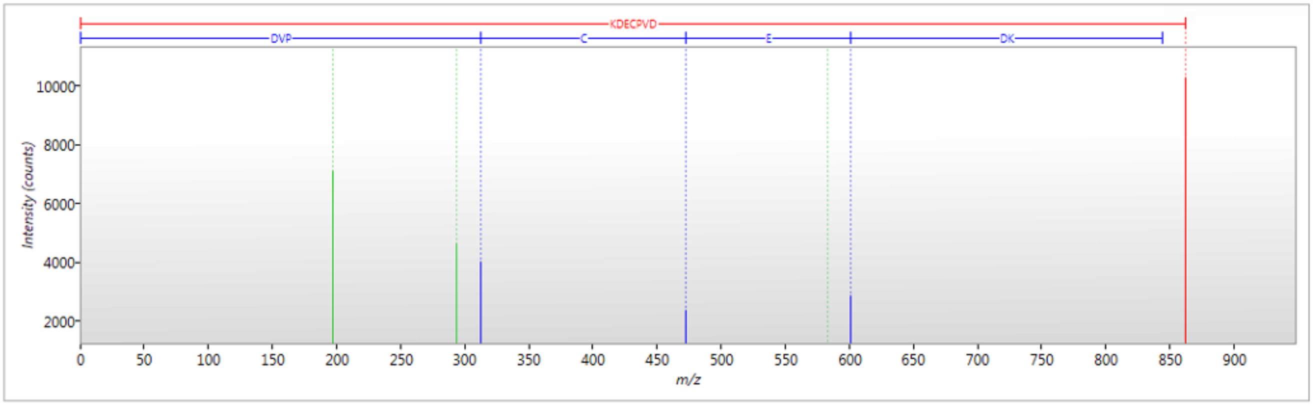

Fragment ions for: MAQTSSYFMLISCLMFLSQSQGQEAQTELPQAR

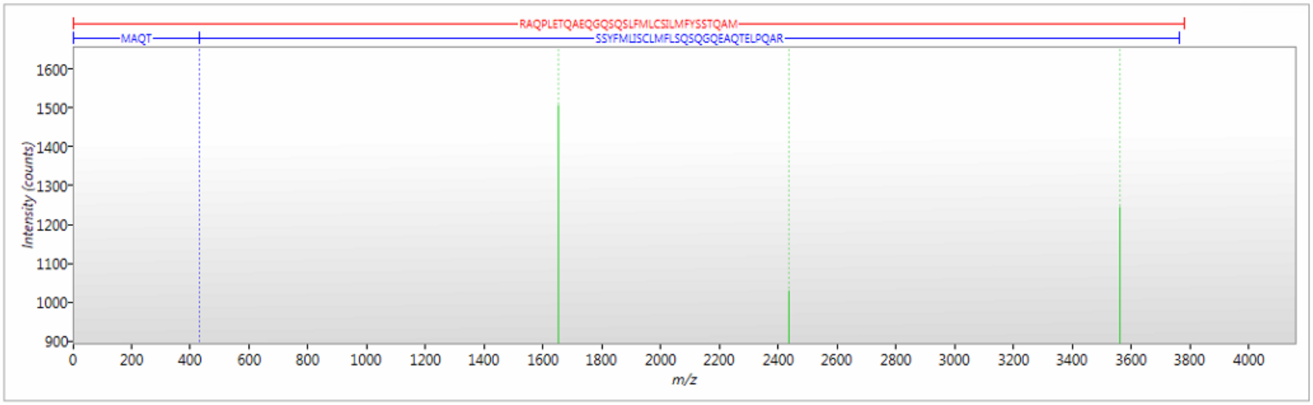

Fragment ions for: SWGIGAPSSVNPGYCVSLTSSTGFQK

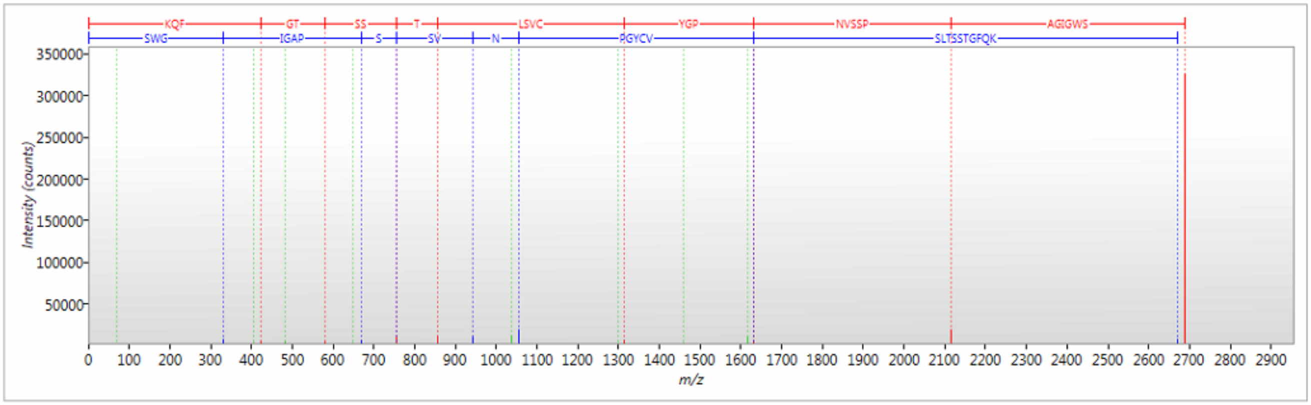

P11117

Lysosomal acid phosphatase OS=Homo sapiens GN=ACP2 PE=1 SV=3
5 peptides

Sequence	Peptide Ion	Score	Hits	Mass	Charge	Tags	Conflicts	Modifications	In quantitation	Average Normalised Abundances			
										PCa	BPH	Bc	Rc
CPLQDFLR	3417	7.36	4	1047.5278	2		0	[1] Carbamidomethyl C	yes	7.69e+004	7.35e+004	4.03e+004	1.39e+005
DPYQEEEWPQGFGQLTK	6536	5.92	2	2051.0637	3		0		no	6.51e+004	4.78e+004	2.02e+004	2.86e+004
FPLGPCPR	226	6.55	3	942.5178	2		0	[6] Carbamidomethyl C	yes	6.51e+005	1.74e+006	1.31e+006	9.69e+005
QTPEYQNESSR	4272	6.17	2	1337.5738	2		1		no	1.72e+005	9.94e+004	7.31e+004	1.71e+005
QTPEYQNESSR	4291	6.17	2	1337.5693	3		1		no	1.31e+004	5440.79	2860.27	1.28e+004
TYPKDPYQEEEWPQGFGQLTK	4977	6.84	4	2540.1879	3		0		yes	1.96e+005	1.44e+005	1.27e+005	2.89e+005
TYPKDPYQEEEWPQGFGQLTK	28353	6.84	3	2540.1784	2		0		yes	879.82	113.84	181.07	1.30e+004

Fragment ions for: CPLQDFLR

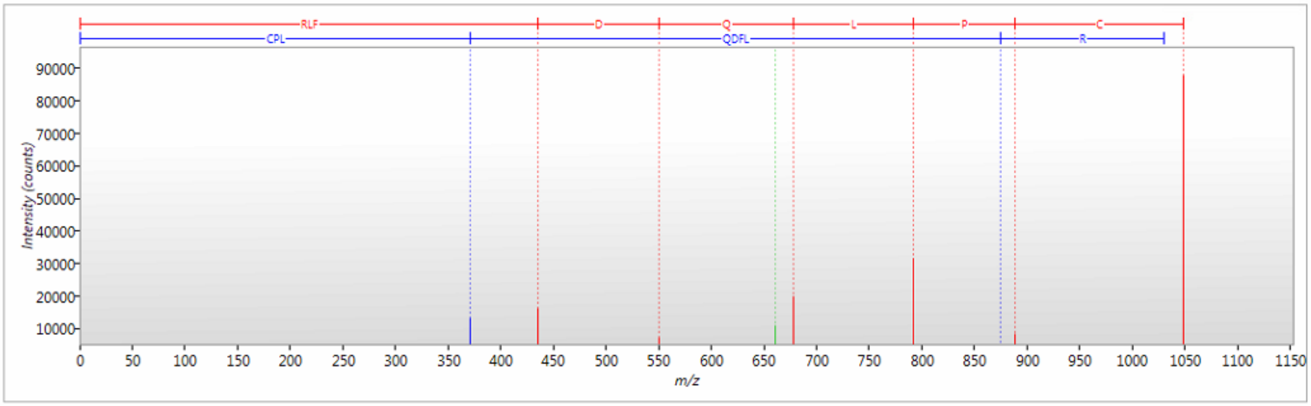

Fragment ions for: FPLGPCPR

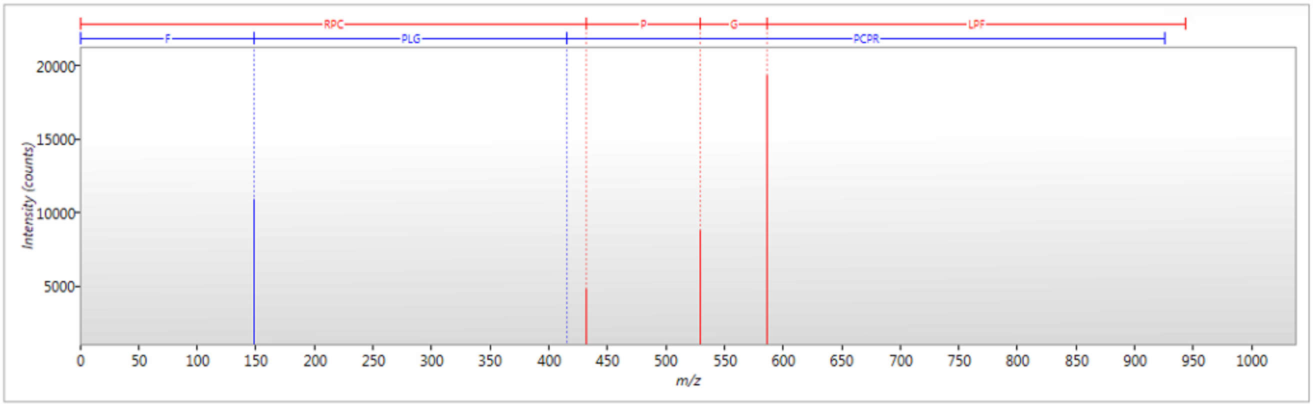

Fragment ions for: TYPKDPYQEEEWPQGFGQLTK

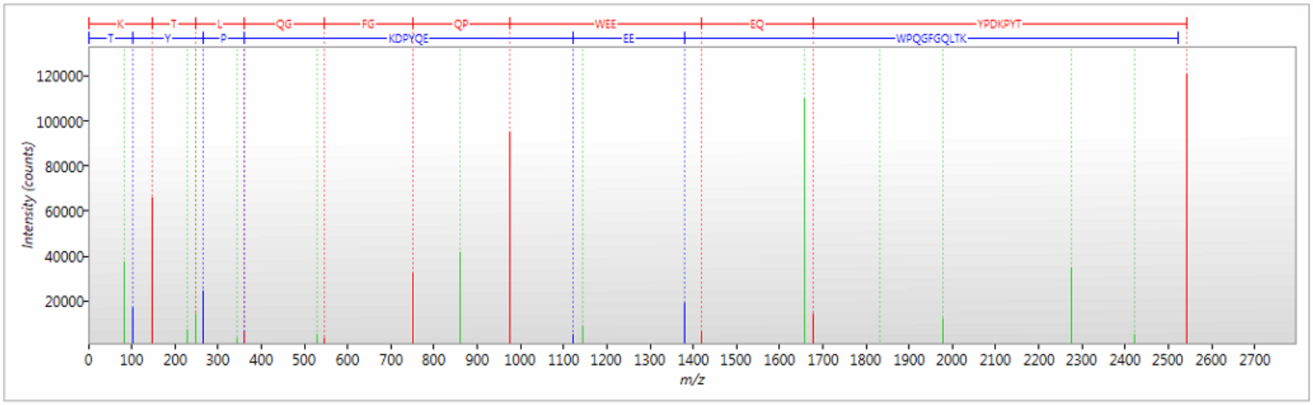

P08246

Neutrophil elastase OS=Homo sapiens GN=ELANE PE=1 SV=1
3 peptides

Sequence	Peptide Ion	Score	Hits	Mass	Charge	Tags	Conflicts	Modifications	In quantitation	Average Normalised Abundances			
										PCa	BPH	Bc	Rc
GIASVLQELNVTVVTSLCR	108	6.36	2	2058.1066	3		0	[18] Carbamidomethyl C	yes	3.06e+006	9.87e+006	5.81e+006	7.10e+006
SNVCTLVR	774	8.50	7	947.4753	2		0	[4] Carbamidomethyl C	yes	7.52e+004	4.72e+005	2.14e+005	9.47e+004
SNVCTLVR	8331	8.50	7	947.4689	1		0	[4] Carbamidomethyl C	no	1.00e+004	2.69e+004	2.93e+004	1.16e+004
VVLGAHNLSR	1378	7.85	2	1064.5671	2		0		yes	8.65e+004	1.44e+005	1.91e+005	8.67e+004
VVLGAHNLSR	21155	7.85	2	1064.5979	1		0		yes	948.68	399.85	4308.81	883.22

Fragment ions for: GIASVLQELNVTVVTSLCR

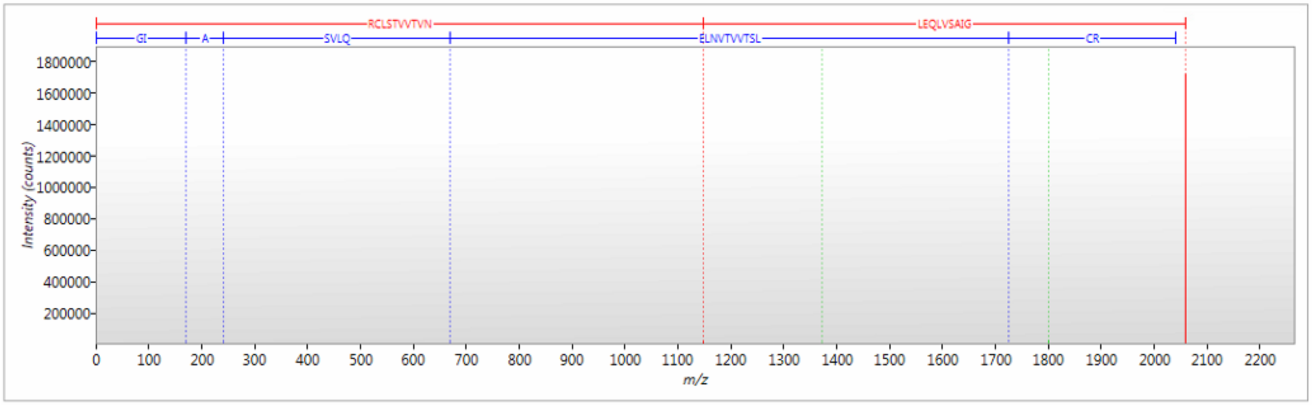

Fragment ions for: SNVCTLVR

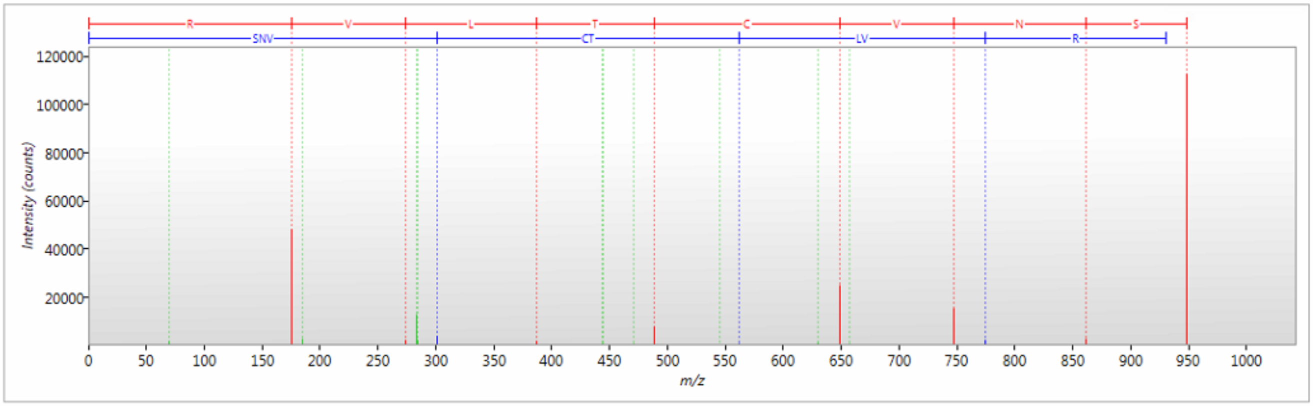

Fragment ions for: VVLGAHNLSR

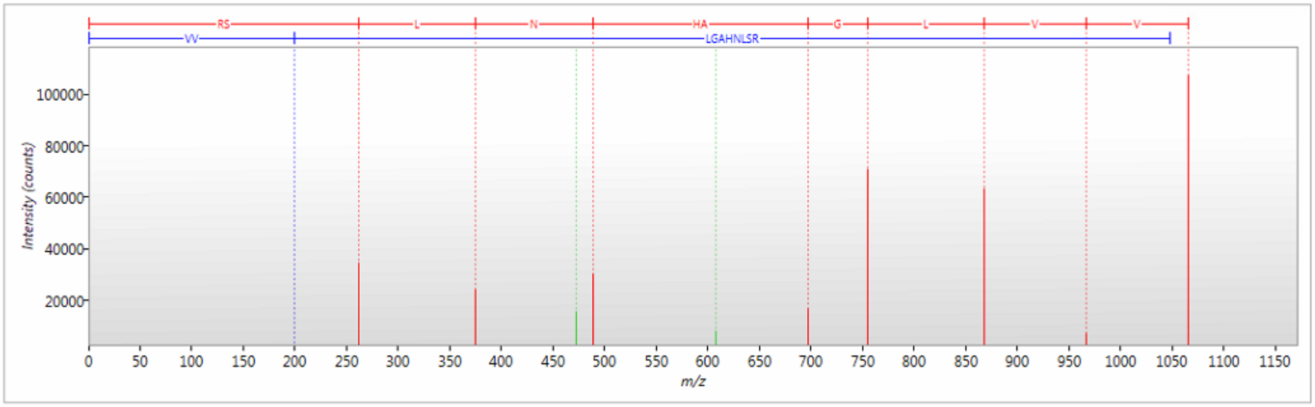

P10153

Non-secretory ribonuclease OS=Homo sapiens GN=RNASE2 PE=1 SV=2
4 peptides

Sequence	Peptide Ion	Score	Hits	Mass	Charge	Tags	Conflicts	Modifications	In quantitation	Average Normalised Abundances			
										PCa	BPH	Bc	Rc
NCHHSGSQVPLIHCNLTTPSPQNISNCR	37114	6.28	2	3227.5388	4		0	[2] Carbamidomethyl C | [14] Carbamidomethyl C | [27] Carbamidomethyl C	no	1.52e+004	1520.83	552.59	4546.25
RDPPQYPVVPVHLDR	1826	9.21	13	1786.9502	3		4		no	8.00e+005	7.19e+005	5.05e+005	1.12e+006
RDPPQYPVVPVHLDR	1834	9.21	11	1786.8730	2		4		no	7.58e+005	4.67e+005	3.11e+005	7.08e+005
YAQTPANMFYIVACDNR	3357	9.22	18	2032.9330	2		0	[14] Carbamidomethyl C	yes	3.96e+005	9.64e+004	9.22e+004	3.96e+005
YAQTPANMFYIVACDNR	7697	9.22	3	2032.9203	3		0	[14] Carbamidomethyl C	yes	7.79e+004	2.44e+004	2.00e+004	4.83e+004
YAQTPANMFYIVACDNR	4203	8.65	9	2048.9156	2		0	[8] Oxidation M | [14] Carbamidomethyl C	yes	2.63e+005	6.99e+004	2.92e+004	2.20e+005

Fragment ions for: YAQTPANMFYIVACDNR

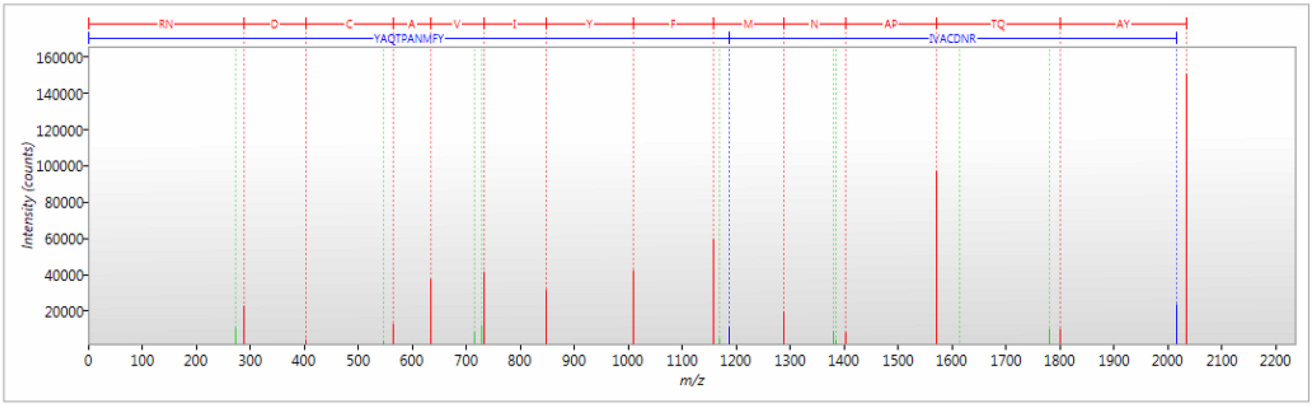

Fragment ions for: YAQTPANMFYIVACDNR

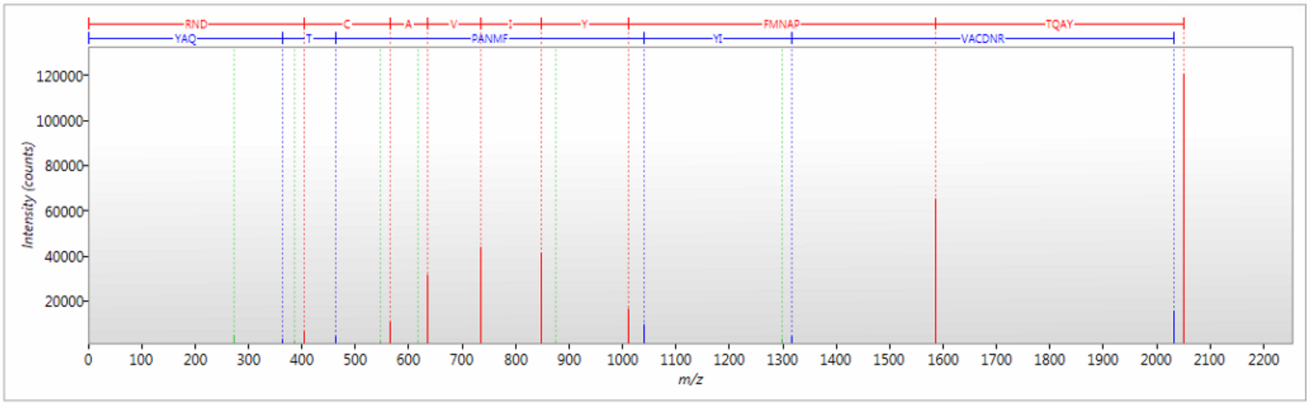

P10451

Osteopontin OS=Homo sapiens GN=SPP1 PE=1 SV=1
12 peptides

Sequence	Peptide Ion	Score	Hits	Mass	Charge	Tags	Conflicts	Modifications	In quantitation	Average Normalised Abundances			
										PCa	BPH	Bc	Rc
AIPVAQDLNAPSDWDSR	49	10.19	29	1853.8932	2		0		yes	4.91e+006	1.41e+006	2.94e+005	8.90e+006
AIPVAQDLNAPSDWDSR	12065	8.56	2	1853.8636	1		0		yes	2.10e+004	2349.36	38.11	5.26e+004
ANDESNEHSDVIDSQELSK	8148	9.02	19	2115.8263	2		1		no	6.43e+004	2.81e+004	8.19e+004	9.37e+004
DSYETSQLDDQSAETHSHK	15603	7.64	2	2176.9175	3		0		no	7767.28	20.15	0.00	12.49
GDSVVYGLR	883	9.36	27	964.4756	1		0		no	3.96e+005	3.11e+005	2.92e+005	7.03e+005
GKDSYETSQLDDQSAETHSHK	2773	9.70	12	2362.0429	4		0		no	1.21e+005	2.30e+004	1.41e+004	1.34e+005
GKDSYETSQLDDQSAETHSHK	18667	---	---	2362.0330	5		0		no	5575.23	1809.25	66.66	4217.73
GKDSYETSQLDDQSAETHSHK	13960	---	---	2362.0279	2		0		no	2.95e+004	6476.19	8218.69	5.87e+004
GKDSYETSQLDDQSAETHSHK	12532	---	---	2362.0287	2		1		no	3280.36	732.37	2933.33	3.50e+004
GKDSYETSQLDDQSAETHSHK	4598	9.56	5	2362.0252	3		0		no	9.44e+004	1.66e+004	2.21e+004	1.60e+004
GKDSYETSQLDDQSAETHSHK	3955	9.70	19	2362.0292	3		0		no	2.10e+005	7.52e+004	4.88e+004	2.80e+005
GKDSYETSQLDDQSAETHSHK	3829	8.50	2	2362.0276	3		0		no	1.27e+005	6.21e+004	6.42e+004	2.21e+005
GKDSYETSQLDDQSAETHSHK	22782	---	---	2362.0290	2		0		no	9947.65	20.17	299.55	726.90
GKDSYETSQLDDQSAETHSHK	2706	9.56	6	2362.0348	4		0		no	1.46e+005	2.86e+004	3.58e+004	4.06e+004
ISHELDSASSEVN	8146	8.98	16	1386.6102	1		0		no	2.39e+004	8453.70	1.26e+004	3.29e+004
ISHELDSASSEVN	223	8.98	18	1386.6220	2		0		no	7.87e+005	7.03e+005	2.46e+005	9.83e+005
KANDESNEHSDVIDSQELSK	12254	8.30	7	2244.0060	2		0		no	3.96e+004	8692.03	3355.67	6.88e+004
QETLPSK	3207	8.97	15	801.4198	1		0		no	1.94e+004	7762.62	3168.97	5.23e+004
QETLPSK	1769	9.24	4	801.4157	2		0		no	6.80e+004	4.41e+004	2.50e+004	9.14e+004
QETLPSK	750	8.97	16	801.4209	2		0		no	1.16e+005	1.17e+005	5.00e+004	1.85e+005
QLYNKYPDAVATWLNPDPSQK	4022	9.67	26	2447.2022	2		0		yes	4.58e+005	1.07e+005	7.29e+004	6.74e+005
QLYNKYPDAVATWLNPDPSQK	395	9.67	28	2447.2125	3		0		yes	3.46e+006	1.29e+006	3.70e+005	5.62e+006
QNLLAPQNAVSSEETNDFKQETLPSK	15602	9.06	21	2887.4166	2		0		no	1.53e+004	1040.55	97.36	1.50e+004
QNLLAPQNAVSSEETNDFKQETLPSK	726	9.06	29	2887.4055	3		0		no	1.09e+006	2.42e+005	2.49e+005	1.07e+006
RPDIQYPDATDEDITSHMESEELNGAYK	16392	7.96	2	3239.4217	3		0	[18] Oxidation M	no	1.01e+004	3175.95	3927.86	1.81e+004
YPDAVATWLNPDPSQK	282	9.94	20	1800.8694	2		0		yes	1.57e+006	1.16e+006	5.08e+005	4.30e+006
YPDAVATWLNPDPSQK	23471	---	---	1800.8552	1		0		yes	1917.48	50.20	306.93	2.78e+004

Fragment ions for: AIPVAQDLNAPSDWDSR

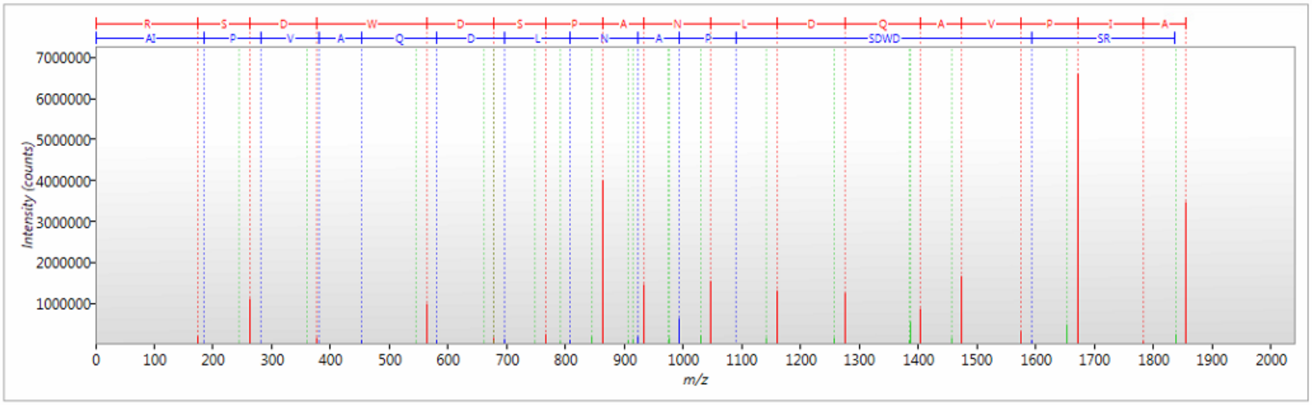

Fragment ions for: QLYNKYPDAVATWLNPDPSQK

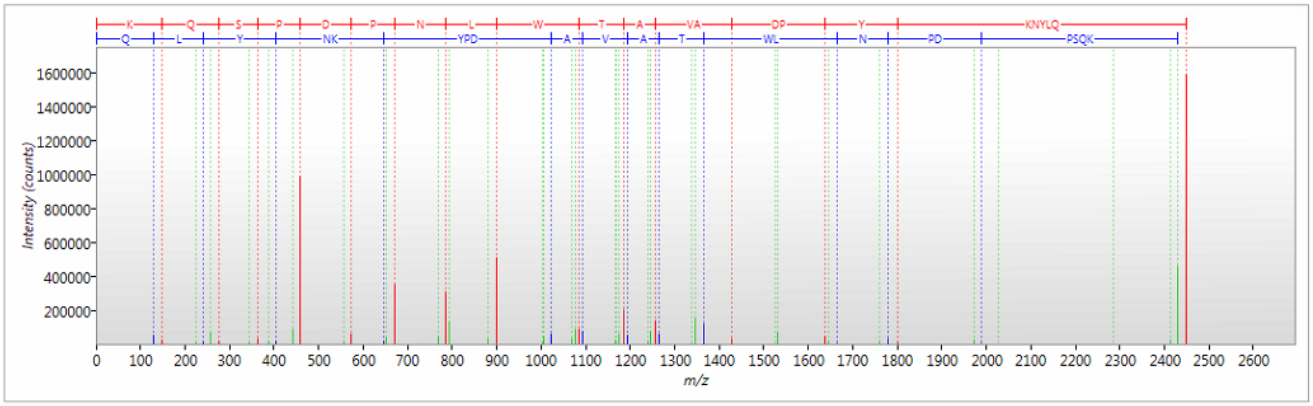

Fragment ions for: YPDAVATWLNPDPSQK

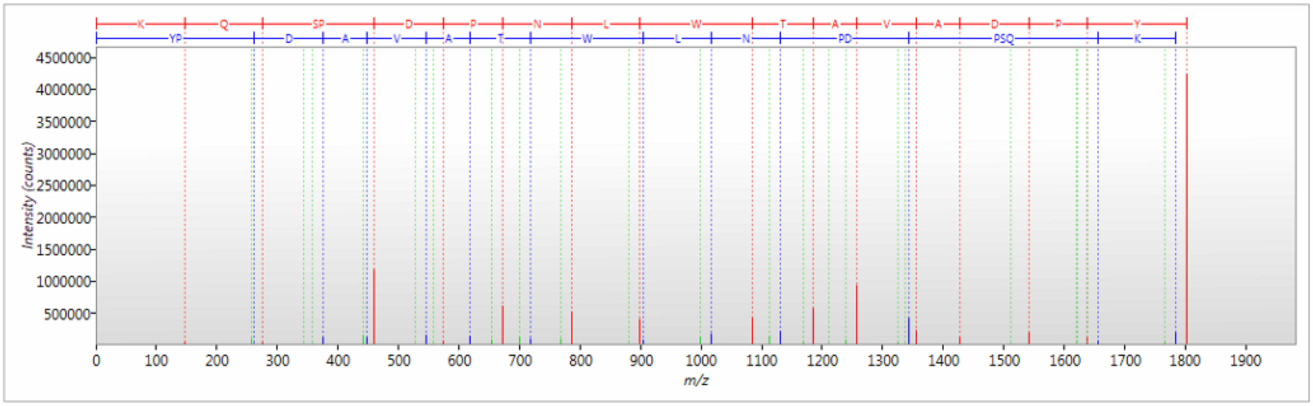

P04746

Pancreatic alpha-amylase OS=Homo sapiens GN=AMY2A PE=1 SV=2
3 peptides

Sequence	Peptide Ion	Score	Hits	Mass	Charge	Tags	Conflicts	Modifications	In quantitation	Average Normalised Abundances			
										PCa	BPH	Bc	Rc
DFPAVPYSGWDFNDGK	3244	9.05	24	1813.8456	2		1		yes	6.36e+005	3.06e+005	1.28e+005	4.56e+005
NWGEGWGFVPSDR	40574	8.92	4	1505.7445	1		0		yes	3908.80	198.28	149.71	3108.02
TGSGDIENYNDATQVR	828	9.47	27	1738.7766	2		1		yes	5.69e+005	3.87e+005	1.83e+005	6.98e+005

Fragment ions for: DFPAVPYSGWDFNDGK

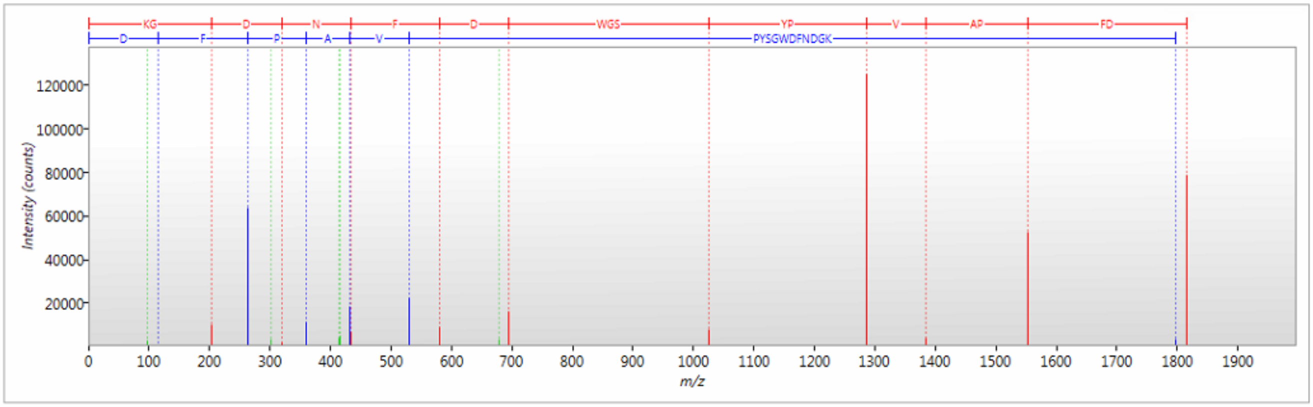

Fragment ions for: NWGEGWGFVPSDR

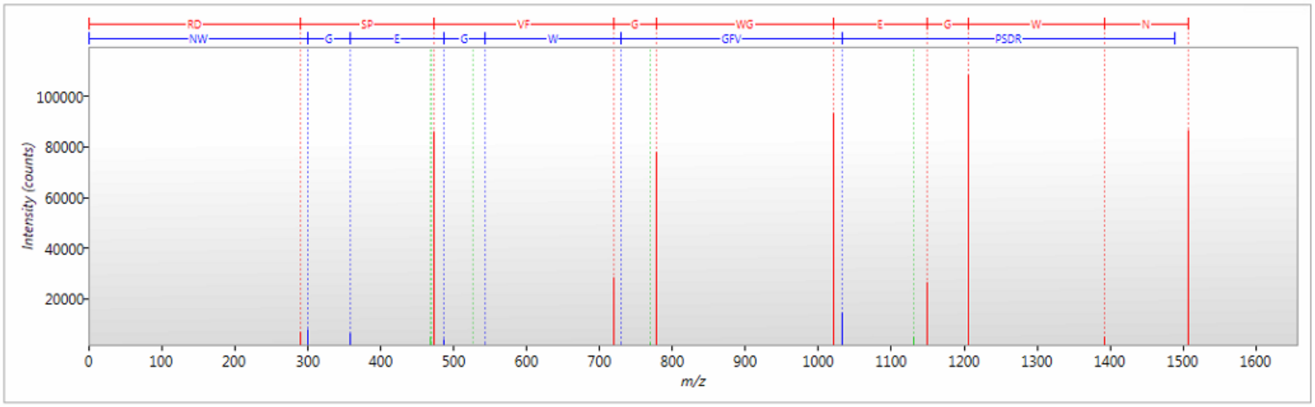

Fragment ions for: TGSGDIENYNDATQVR

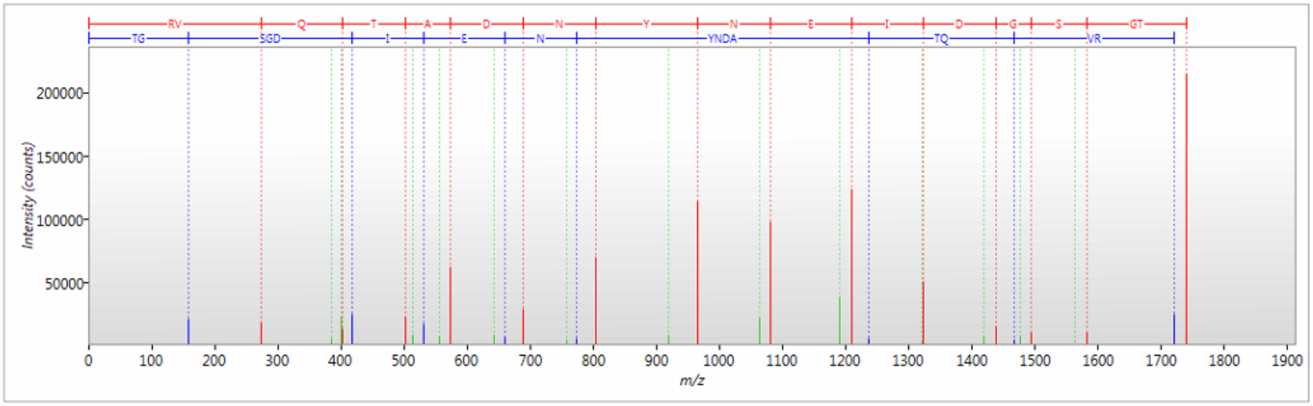

P30086

Phosphatidylethanolamine-binding protein 1 OS=Homo sapiens GN=PEBP1 PE=1 SV=3
3 peptides

Sequence	Peptide Ion	Score	Hits	Mass	Charge	Tags	Conflicts	Modifications	In quantitation	Average Normalised Abundances			
										PCa	BPH	Bc	Rc
CDEPILSNRSGDHR	1506	6.48	2	1654.7567	3		0	[1] Carbamidomethyl C	yes	1.70e+005	8.96e+005	2.67e+005	3.94e+005
CDEPILSNRSGDHR	4567	6.48	2	1654.7438	2		0	[1] Carbamidomethyl C	yes	6.62e+004	2.32e+005	9.78e+004	1.17e+005
LYEQLSGK	504	7.06	3	936.4529	1		0		yes	3.00e+005	6.88e+005	7.13e+005	3.37e+005
VLTPTQVK	27675	7.13	3	884.5237	1		0		yes	210.67	0.96	45.33	945.23

Fragment ions for: CDEPILSNRSGDHR

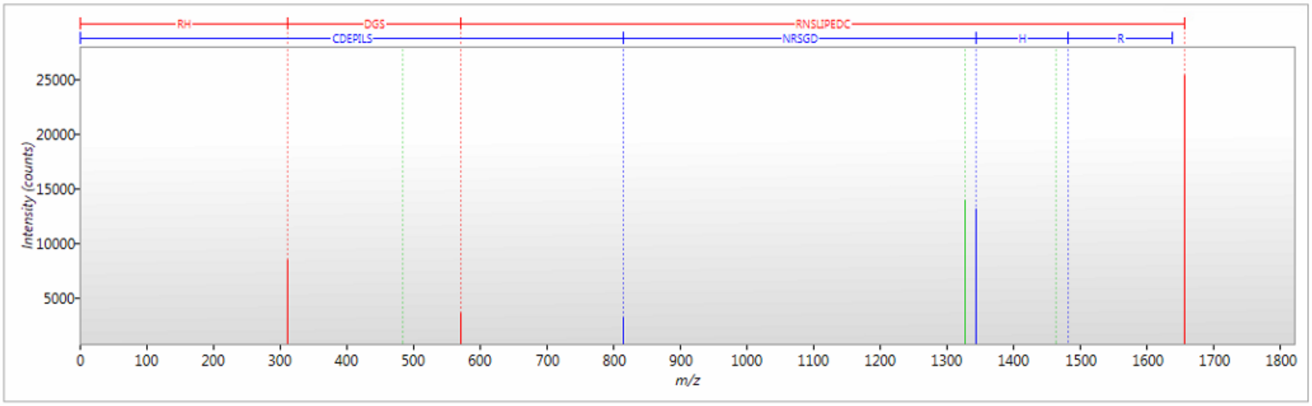

Fragment ions for: LYEQLSGK

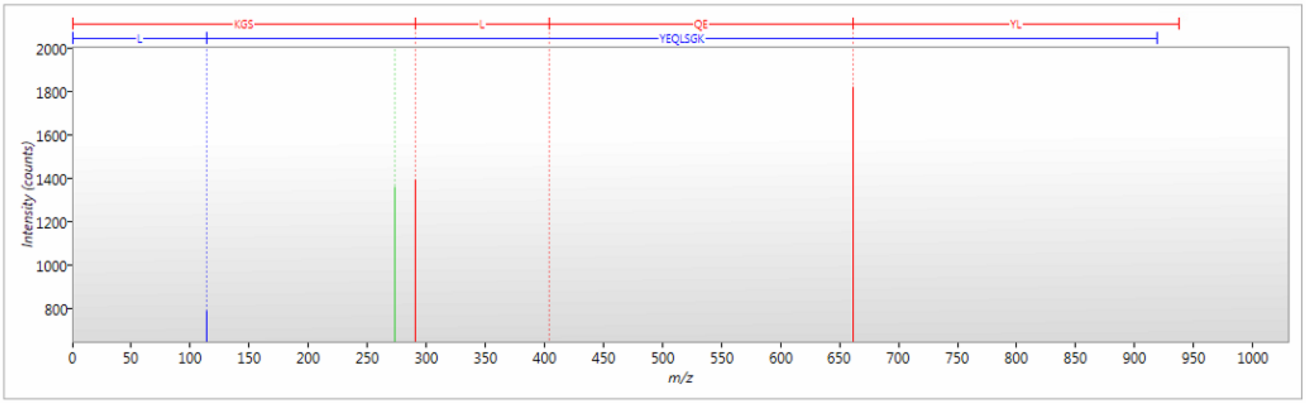

Fragment ions for: VLTPTQVK

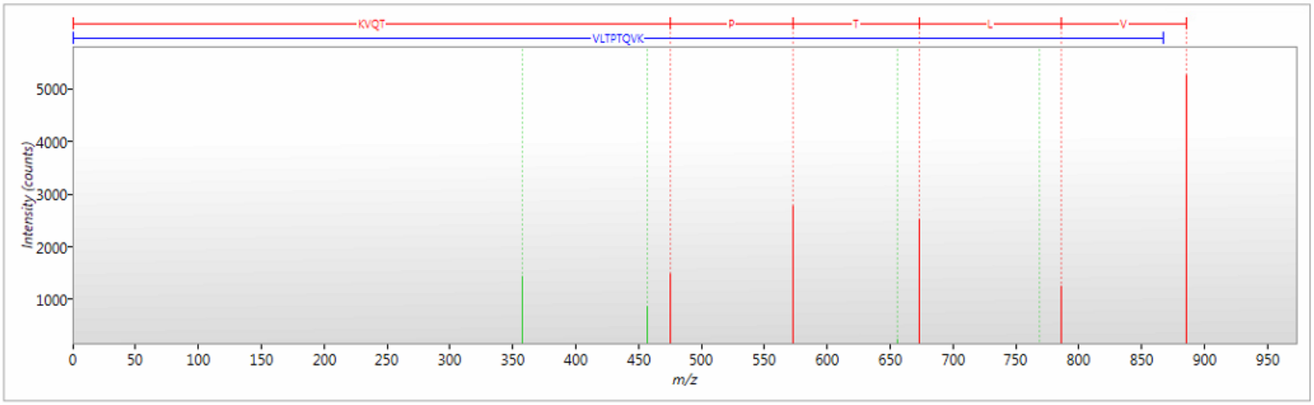

P20742

Pregnancy zone protein OS=Homo sapiens GN=PZP PE=1 SV=4
7 peptides

Sequence	Peptide Ion	Score	Hits	Mass	Charge	Tags	Conflicts	Modifications	In quantitation	Average Normalised Abundances			
										PCa	BPH	Bc	Rc
ATVLNYLPK	3454	8.16	2	1017.4788	2		0		yes	7.01e+004	1.59e+005	1.78e+005	7.47e+004
ATVLNYLPK	6752	8.16	3	1017.4771	1		0		yes	1.81e+005	1.66e+005	2.07e+005	2.05e+005
ATVLNYLPK	11989	7.65	2	1017.5664	1		0		no	1.43e+004	1.33e+004	2.54e+004	1.99e+004
GEESYCICGNER	3626	5.16	2	1472.5341	3		0	[6] Carbamidomethyl C | [8] Carbamidomethyl C	no	3.23e+004	4.41e+004	3.42e+004	1.97e+004
NQGNTWLTAFVLK	59867	8.88	2	1490.7403	2		0		no	2.56e+004	6.78e+004	5.62e+004	5.52e+004
NQGNTWLTAFVLKTFAQAR	33453	7.99	5	2165.0244	2		0		no	3.85e+004	2.15e+004	1.17e+004	1.38e+004
SSGSLLNNAIK	1003	8.83	3	1102.6033	2		0		yes	2.86e+005	3.97e+005	6.70e+005	2.32e+005
SSGSLLNNAIK	12251	8.83	2	1102.5911	1		0		yes	1.01e+004	4769.72	2.28e+004	5619.48
VDSHFR	3010	8.71	5	759.3659	1		0		no	4.04e+004	5.40e+004	8.85e+004	3.70e+004
VDSHFR	3391	8.71	5	759.3723	2		0		no	1.17e+004	4.55e+004	5.23e+004	1.97e+004
YGAATFTR	1217	9.19	7	885.4315	2		0		yes	4.34e+004	1.73e+005	1.72e+005	3.39e+004

Fragment ions for: ATVLNYLPK

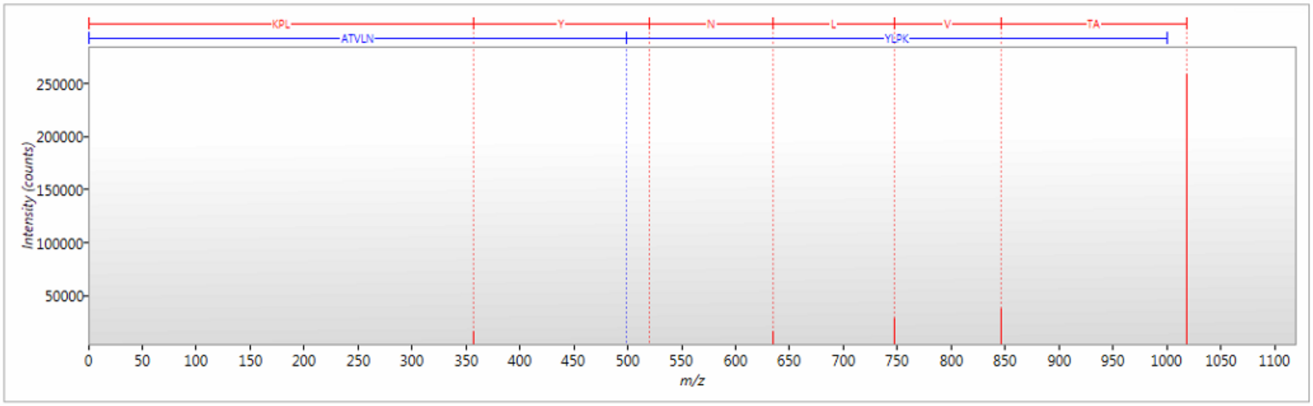

Fragment ions for: SSGSLLNNAIK

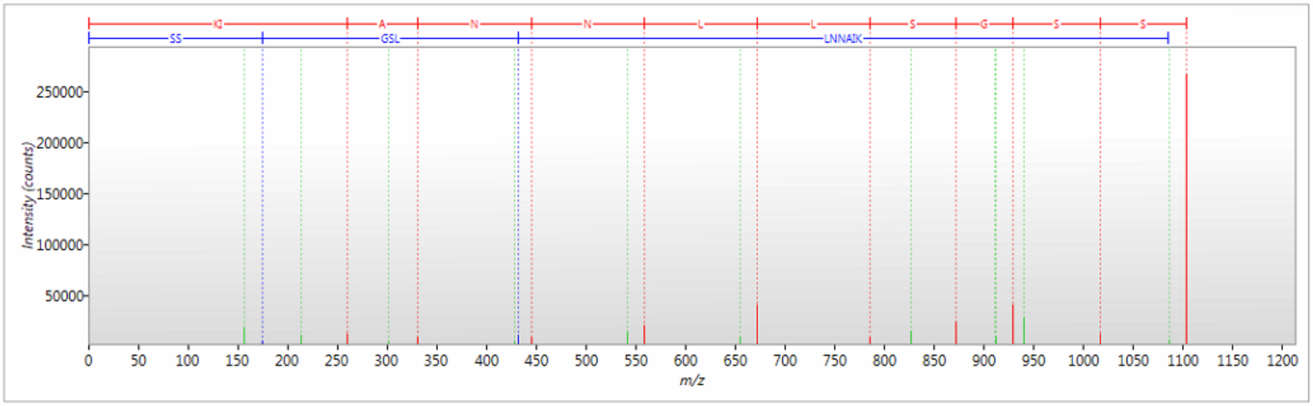

Fragment ions for: YGAATFTR

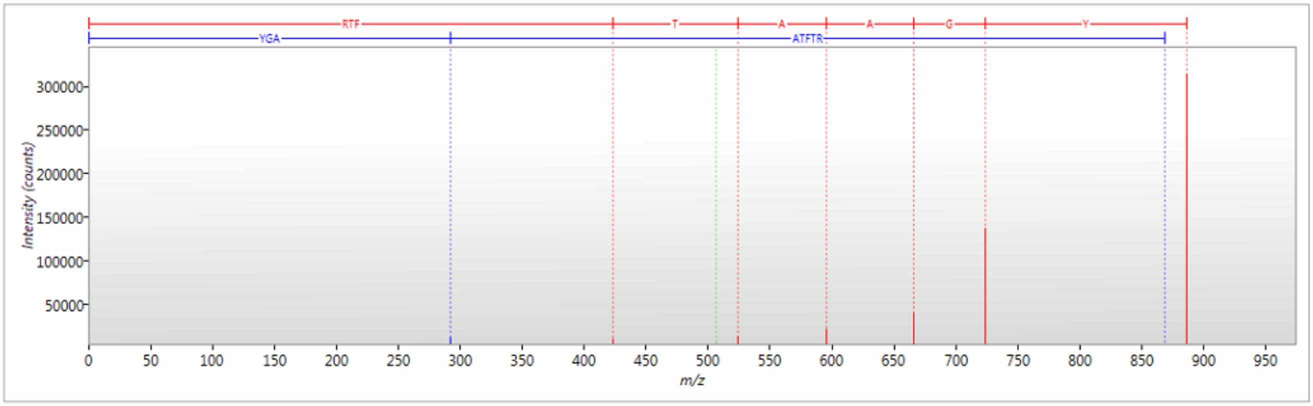

P41222

Prostaglandin-H2 D-isomerase OS=Homo sapiens GN=PTGDS PE=1 SV=1
9 peptides

Sequence	Peptide Ion	Score	Hits	Mass	Charge	Tags	Conflicts	Modifications	In quantitation	Average Normalised Abundances			
										PCa	BPH	Bc	Rc
AQGFTEDTIVFLPQTDK	93	10.15	38	1908.9483	2		0		yes	6.98e+006	3.46e+006	8.58e+005	3.96e+006
AQGFTEDTIVFLPQTDK	625	10.15	38	1908.9242	3		0		yes	1.24e+006	8.88e+005	5.40e+005	6.90e+005
AQGFTEDTIVFLPQTDK	11612	---	---	1908.9079	1		0		yes	5.51e+004	9478.26	5261.12	2.48e+004
AQGFTEDTIVFLPQTDKCMTEQ	2544	9.64	11	2558.1465	2		1	[18] Carbamidomethyl C	no	3.08e+005	2.88e+005	2.34e+005	5.02e+005
AQGFTEDTIVFLPQTDKCMTEQ	913	9.64	11	2558.1419	3		1	[18] Carbamidomethyl C	no	1.06e+006	3.43e+005	2.11e+005	9.77e+005
AQGFTEDTIVFLPQTDKCMTEQ	2717	9.04	26	2574.1621	2		0	[18] Carbamidomethyl C | [19] Oxidation M	no	5.19e+005	6.63e+004	3.52e+004	1.87e+005
AQGFTEDTIVFLPQTDKCMTEQ	882	9.04	26	2574.1686	3		0	[18] Carbamidomethyl C | [19] Oxidation M	no	1.42e+006	4.78e+005	2.44e+005	6.60e+005
FTAFCK	1057	9.17	29	772.3566	1		0	[5] Carbamidomethyl C	no	6.44e+005	3.17e+005	1.82e+005	3.94e+005
FTAFCK	548	9.17	29	772.3619	2		0	[5] Carbamidomethyl C	no	5.64e+005	5.81e+005	2.23e+005	3.20e+005
GPGEDFR	280	9.01	26	776.3640	2		1		no	5.94e+005	6.92e+005	2.79e+005	4.99e+005
MATLYSR	949	9.26	29	840.4121	2		0		no	2.92e+005	2.37e+005	1.27e+005	2.37e+005
MATLYSR	3339	9.26	26	840.4057	1		0		no	9.29e+004	4.33e+004	4.02e+004	1.21e+005
MATLYSR	2152	8.75	4	856.4152	2		0	[1] Oxidation M	no	1.15e+005	8.90e+004	4.81e+004	4.99e+004
MATLYSR	10858	7.96	6	856.4169	2		0	[1] Oxidation M	no	1.23e+004	4688.58	1426.55	7352.22
TMLLQPAGSLGSYSYR	14880	8.95	8	1742.8515	1		0		yes	1.23e+004	1411.25	2841.06	2.69e+004
TMLLQPAGSLGSYSYR	156	9.98	35	1742.8614	2		0		yes	3.14e+006	1.50e+006	5.87e+005	3.15e+006
TMLLQPAGSLGSYSYR	1709	9.19	4	1758.8468	2		0	[2] Oxidation M	no	8.75e+005	3.85e+005	3.46e+005	5.71e+005
TMLLQPAGSLGSYSYR	388	9.37	32	1758.8416	2		0	[2] Oxidation M	yes	2.23e+006	1.88e+006	4.64e+005	1.33e+006

Fragment ions for: AQGFTEDTIVFLPQTDK

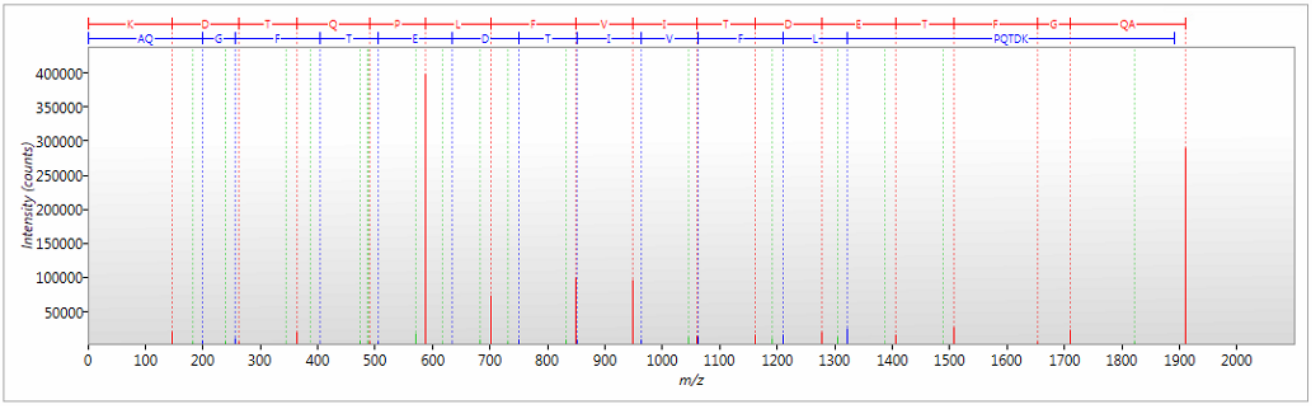

Fragment ions for: TMLLQPAGSLGSYSYR

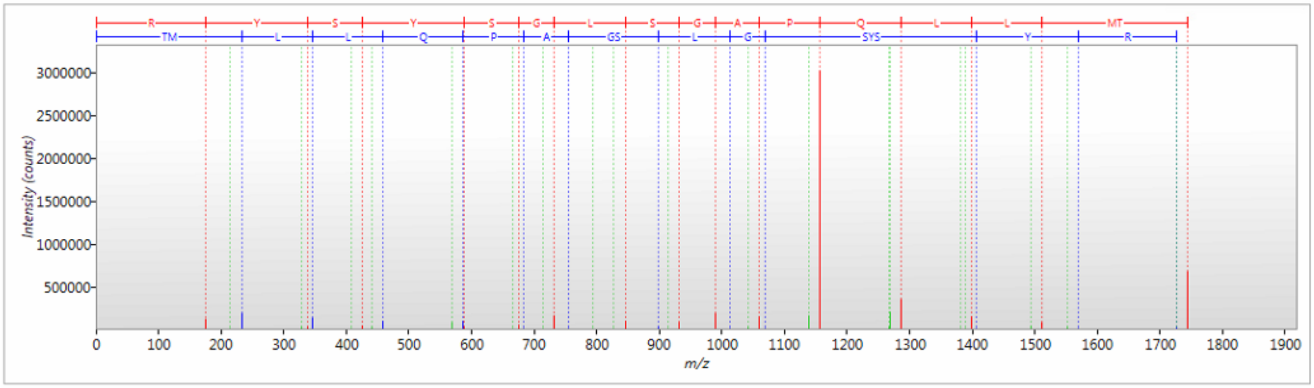

Fragment ions for: TMLLQPAGSLGSYSYR

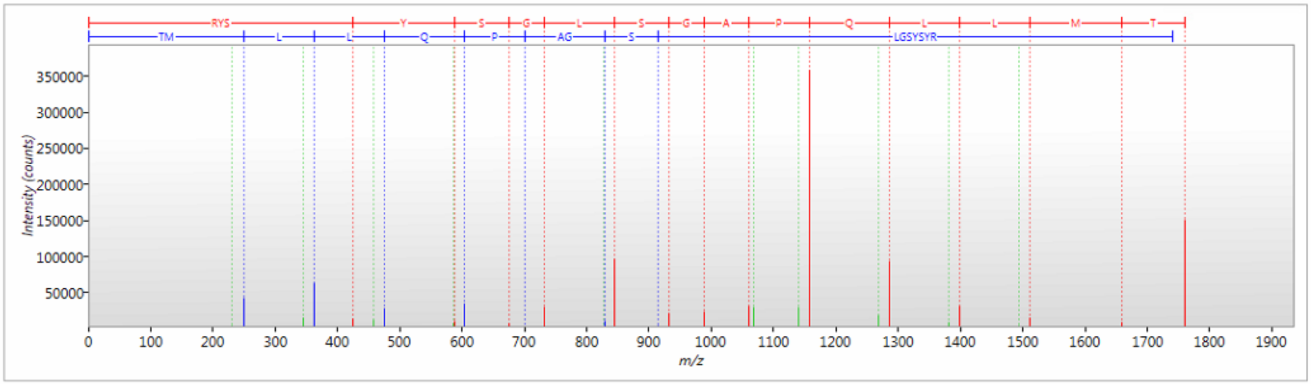

P06702

Protein S100-A9 OS=Homo sapiens GN=S100A9 PE=1 SV=1
3 peptides

Sequence	Peptide Ion	Score	Hits	Mass	Charge	Tags	Conflicts	Modifications	In quantitation	Average Normalised Abundances			
										PCa	BPH	Bc	Rc
DLQNFLK	2467	8.49	5	876.4655	2		0		yes	1.90e+004	1.13e+005	4.15e+004	3.72e+004
LGHPDTLNQGEFK	3621	9.24	4	1454.7006	2		0		yes	3.02e+004	2.08e+005	2.24e+004	7.36e+004
LGHPDTLNQGEFK	5004	6.41	2	1454.6958	2		0		yes	1.24e+004	7.77e+004	6.62e+004	2.11e+004
LTWASHEK	12038	8.88	2	970.4358	3		0		no	534.46	1360.21	4493.00	828.10

Fragment ions for: DLQNFLK

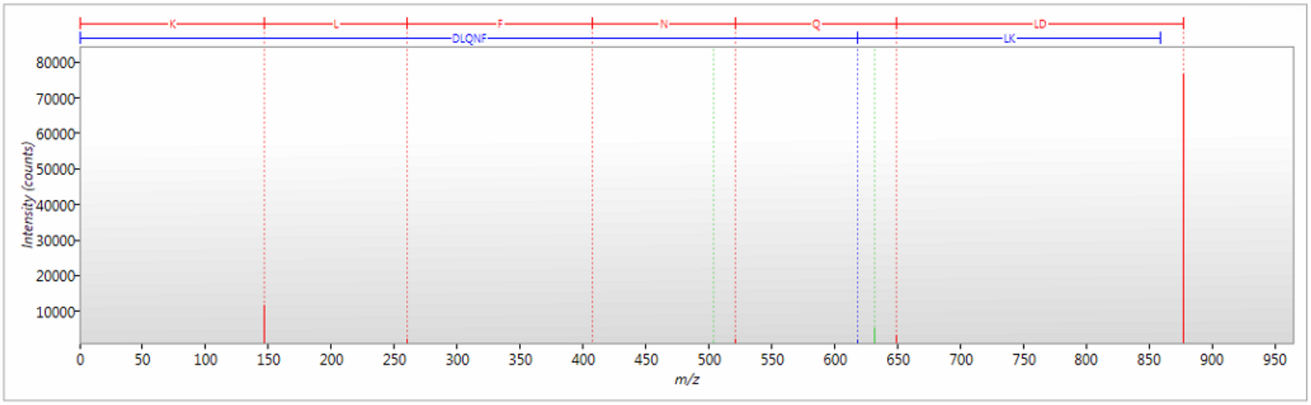

Fragment ions for: LGHPDTLNQGEFK

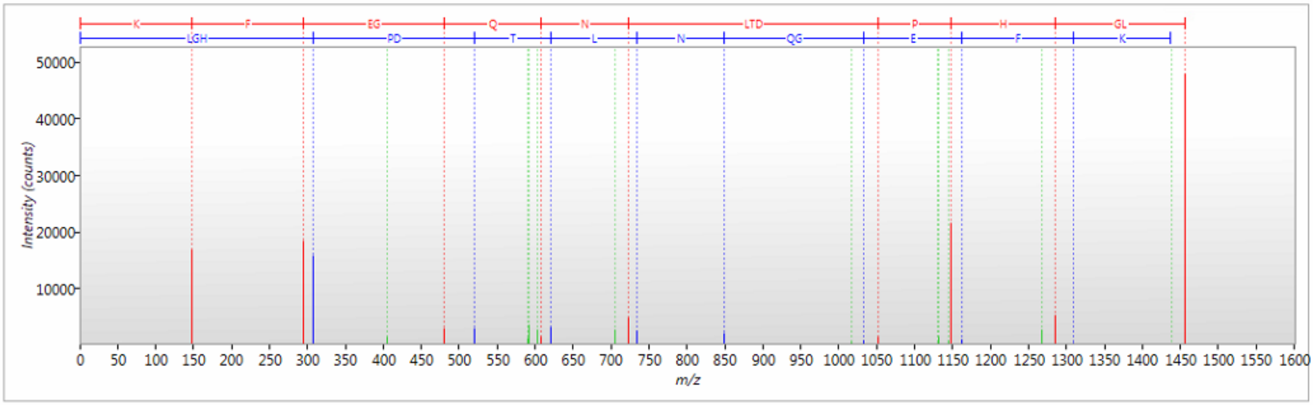

Fragment ions for: LGHPDTLNQGEFK

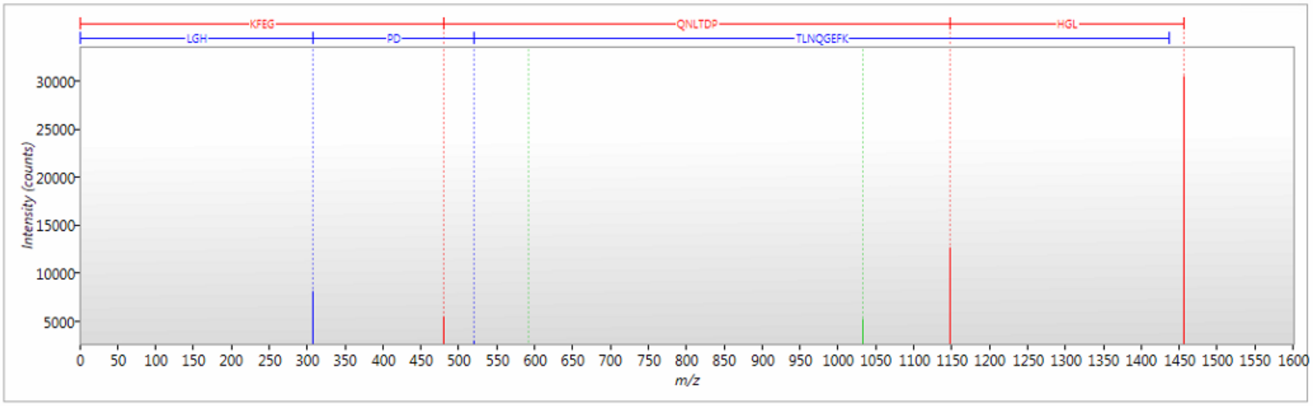

Q8WZ75

Roundabout homolog 4 OS=Homo sapiens GN=ROBO4 PE=1 SV=1
7 peptides

Sequence	Peptide Ion	Score	Hits	Mass	Charge	Tags	Conflicts	Modifications	In quantitation	Average Normalised Abundances			
										PCa	BPH	Bc	Rc
APSPPTTYGYISVPTASEFTDMGR	390	5.64	2	2544.1176	2		0		yes	1.71e+006	4.42e+006	3.11e+006	2.26e+006
EADCVFIDASSPPSPRDEIFLTPNLSLPLWEWRPDWLEDMEVSHTQR	21052	6.15	2	5580.5106	4		0	[4] Carbamidomethyl C	no	1868.24	4.35e+004	0.00	8204.29
EDFQIQPR	17680	7.54	11	1031.4570	1		0		no	2.81e+004	1.46e+004	1.99e+004	3.63e+004
GPDSNVLLLR	2983	7.49	12	1082.5865	2		0		yes	5.62e+004	7.05e+004	9.01e+004	9.19e+004
LGTAVSR	6941	6.90	7	702.3889	1		0		no	7990.78	6202.56	3313.03	1.63e+004
MDHSDSQWLADTWRSTSGSR	9600	5.58	2	2338.0510	3		0	[1] Oxidation M	no	5.36e+004	2.94e+004	2.95e+004	6.58e+004
VSIQEPQDYTEPVELLAVR	4592	8.20	16	2185.1077	2		0		yes	1.15e+005	2.51e+004	9606.89	1.68e+005
VSIQEPQDYTEPVELLAVR	4830	8.20	16	2185.0882	3		0		yes	1.58e+005	1.32e+005	1.44e+005	1.74e+005

Fragment ions for: APSPPTTYGYISVPTASEFTDMGR

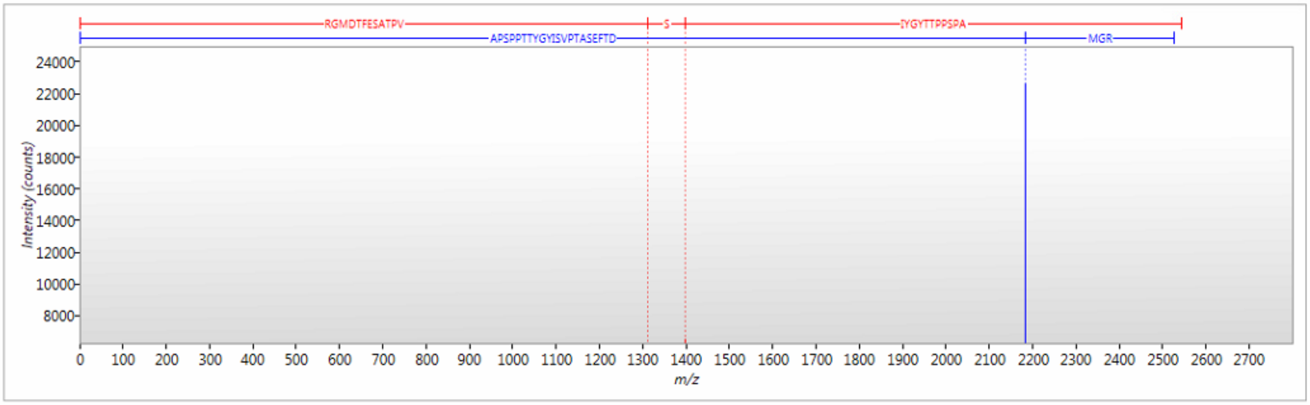

Fragment ions for: GPDSNVLLLR

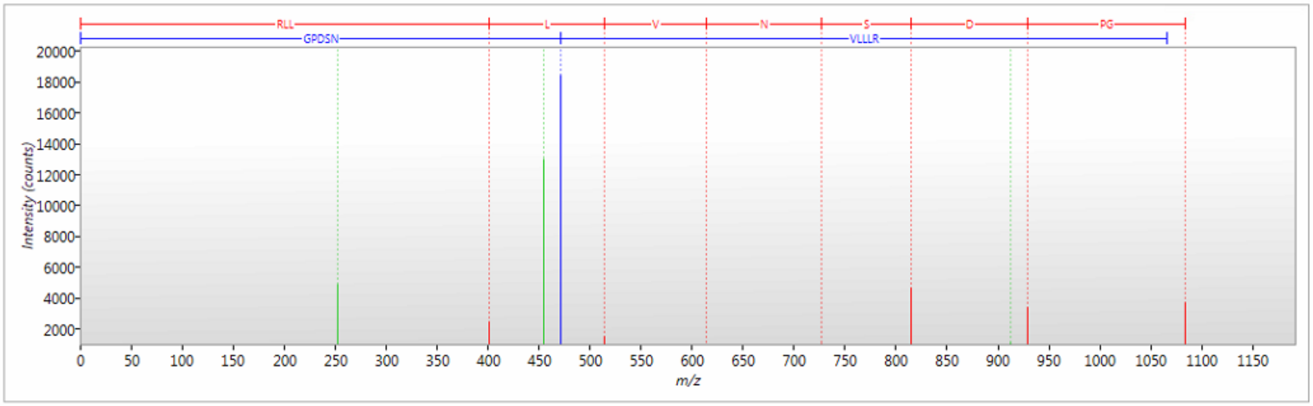

Fragment ions for: VSIQEPQDYTEPVELLAVR

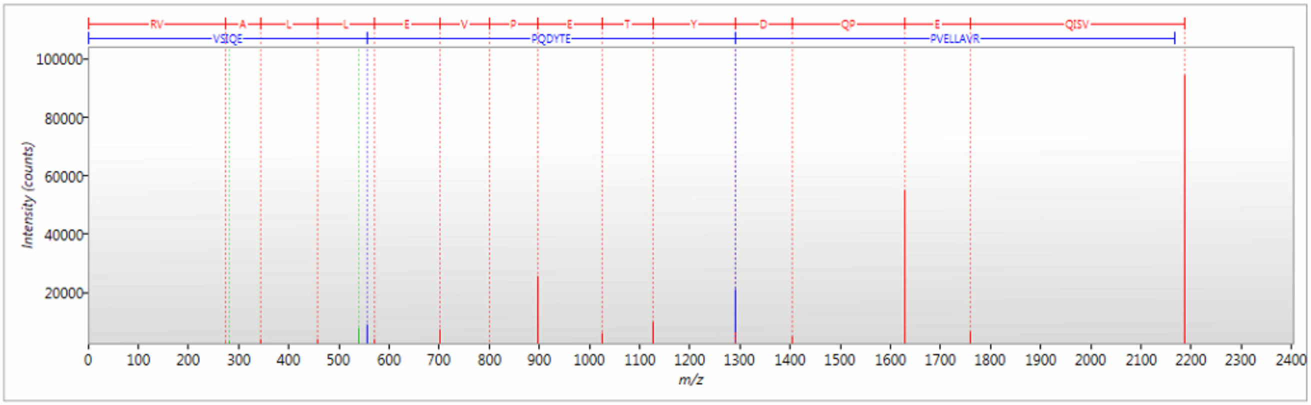

Q12907

Vesicular integral-membrane protein VIP36 OS=Homo sapiens GN=LMAN2 PE=1 SV=1
9 peptides

Sequence	Peptide Ion	Score	Hits	Mass	Charge	Tags	Conflicts	Modifications	In quantitation	Average Normalised Abundances			
										PCa	BPH	Bc	Rc
AAEGWIWR	3544	7.00	4	987.5068	2		0		no	1.34e+005	9.31e+004	7.22e+004	8.57e+004
DNFHGLAIFLDTYPNDETTER	4549	8.20	15	2467.1506	3		0		no	1.48e+005	1.58e+005	7.10e+004	1.68e+005
DNFHGLAIFLDTYPNDETTER	29751	8.20	16	2467.1240	2		0		no	4327.03	3706.71	1707.87	7300.71
DNFHGLAIFLDTYPNDETTER	37431	7.83	2	2467.1291	2		0		no	4126.89	1783.71	1965.75	406.51
LFQLMVEHTPDEESIDWTK	3962	6.38	2	2333.0968	2		0	[5] Oxidation M	yes	1.15e+006	2.16e+005	1.85e+005	1.35e+006
LPTGYYFGASAGTGDLSDNHDIISMK	4819	7.17	5	2729.2700	3		0		yes	2.45e+005	1.82e+005	8.35e+004	3.29e+005
LTPDER	3286	7.50	4	729.3533	1		1		no	5.56e+004	2.95e+004	2.29e+004	4.60e+004
LVPGPVFGSK	2711	8.52	24	999.5716	2		0		no	1.93e+005	2.16e+005	1.63e+005	2.04e+005
LVPGPVFGSK	9165	8.37	20	999.5691	1		0		no	1.44e+004	1.41e+004	8625.87	2.33e+004
NCIDITGVR	523	7.99	21	1046.5196	2		0	[2] Carbamidomethyl C	yes	1.24e+006	1.18e+006	7.86e+005	1.03e+006
NCIDITGVR	6622	7.99	21	1046.4685	1		0	[2] Carbamidomethyl C	yes	9.21e+004	3.85e+004	7.28e+004	7.59e+004
NRDHDTFLAVR	4224	8.35	3	1342.6747	3		0		no	4.65e+004	897.27	1.15e+004	1459.84
NRDHDTFLAVR	4641	8.46	2	1342.6746	3		0		no	5.60e+004	4795.46	354.47	1902.85
WTELAGCTADFR	19989	8.89	23	1425.6304	1		0	[7] Carbamidomethyl C	no	9051.30	1187.95	657.15	1.35e+004
WTELAGCTADFR	41394	7.84	3	1425.6358	1		0	[7] Carbamidomethyl C	no	5433.61	2859.72	1088.07	527.13

Fragment ions for: LFQLMVEHTPDEESIDWTK

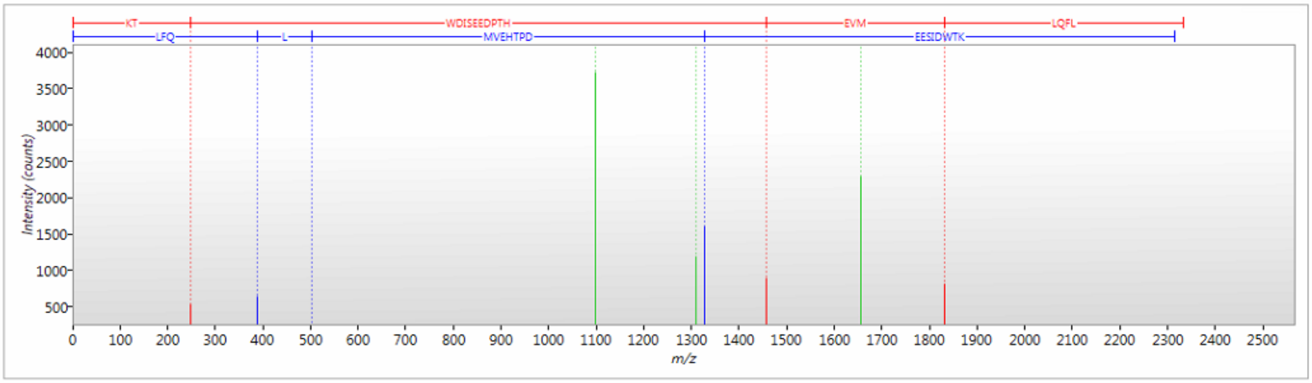

Fragment ions for: LPTGYYFGASAGTGDLSDNHDIISMK

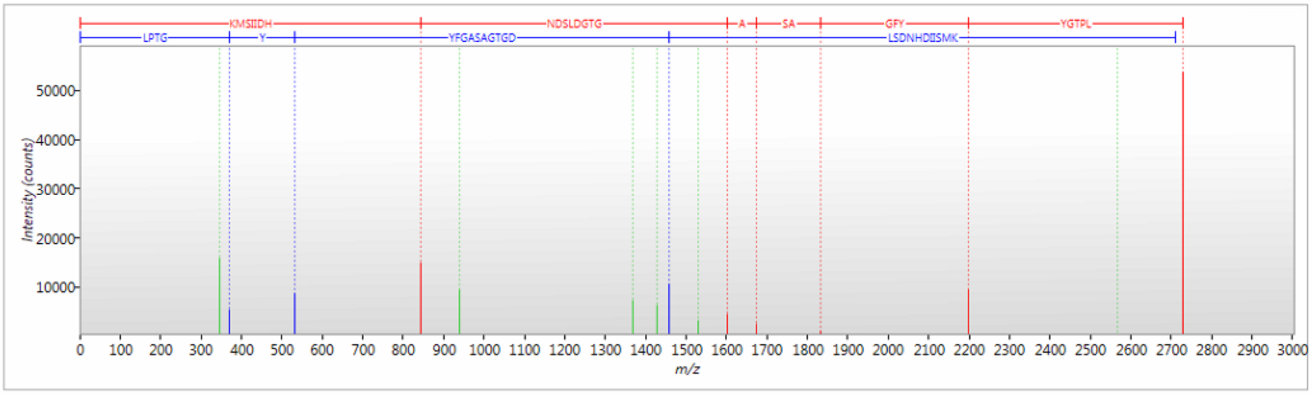

Fragment ions for: NCIDITGVR

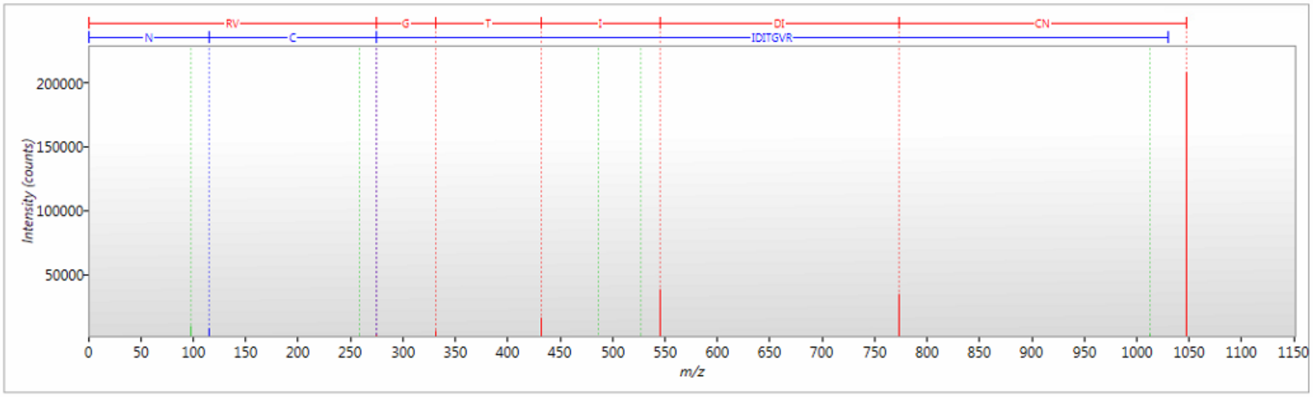

Q14624

Inter-alpha-trypsin inhibitor heavy chain H4 OS=Homo sapiens GN=ITIH4 PE=1 SV=4
9 peptides

Sequence	Peptide Ion	Score	Hits	Mass	Charge	Tags	Conflicts	Modifications	In quantitation	Average Normalised Abundances			
										PCa	BPH	Bc	Rc
FKPTLSQQQK	5105	6.52	6	1203.5503	2		0		no	7.52e+004	1.21e+005	1.05e+005	6.72e+004
GPDVLTATVSGK	5806	7.51	3	1143.5913	2		0		no	5.70e+004	5.00e+004	6.23e+004	4.76e+004
HLQMDIHIFEPQGISFLETESTFMTNQLVDALTTWQNK	43407	6.00	2	4478.1712	4		0	[24] Oxidation M	no	836.93	2862.93	3077.83	868.01
LDYQEGPPGVEISCWSVEL	2346	9.21	28	2177.0087	2		0	[14] Carbamidomethyl C	yes	6.07e+005	1.89e+005	9.82e+004	6.71e+005
NMEQFQVSVSVAPNAK	7467	6.21	2	1747.8319	3		0		no	2.09e+004	2.81e+004	3.70e+004	1.48e+004
NPLVWVHASPEHVVVTR	1250	7.67	14	1939.0153	3		0		yes	8.85e+005	1.00e+006	3.51e+005	1.99e+006
NPLVWVHASPEHVVVTR	1539	7.28	2	1939.0350	3		0		no	7.17e+004	8.20e+004	4.29e+004	2.36e+005
NPLVWVHASPEHVVVTR	2112	7.74	6	1939.0320	3		0		no	4.19e+005	2.16e+005	1.97e+005	5.89e+004
NPLVWVHASPEHVVVTR	7043	7.28	3	1939.0176	4		0		no	4574.78	918.17	6930.64	2.79e+004
QGPVNLLSDPEQGVEVTGQYER	685	9.15	31	2414.1764	3		0		yes	9.57e+005	6.54e+005	3.56e+005	1.32e+006
QGPVNLLSDPEQGVEVTGQYER	1009	9.15	33	2414.1718	2		0		yes	9.24e+005	2.55e+005	1.68e+005	1.43e+006
QGPVNLLSDPEQGVEVTGQYEREK	1572	8.79	13	2671.2884	3		0		no	3.77e+005	1.00e+005	5.91e+004	6.62e+005
QGPVNLLSDPEQGVEVTGQYEREK	2676	8.88	3	2671.2618	3		0		no	2.87e+005	3.38e+004	3.72e+004	4.95e+004
QGPVNLLSDPEQGVEVTGQYEREK	19102	8.79	10	2671.2978	2		0		no	1.98e+004	5078.58	2823.05	2.72e+004
SPEQQETVLDGNLIIR	6163	7.55	10	1810.9765	3		0		no	1.32e+005	3.86e+004	1.27e+005	2.58e+004

Fragment ions for: LDYQEGPPGVEISCWSVEL

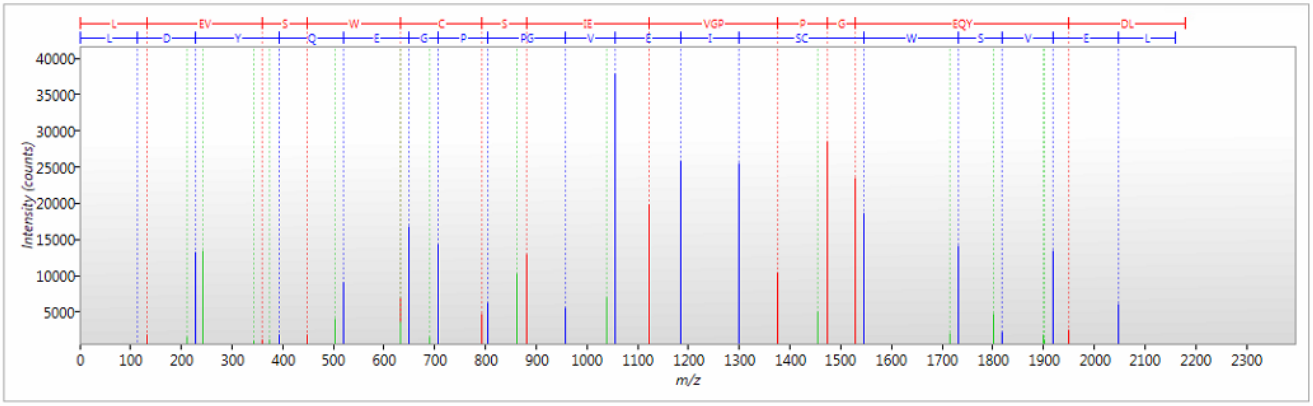

Fragment ions for: NPLVWVHASPEHVVVTR

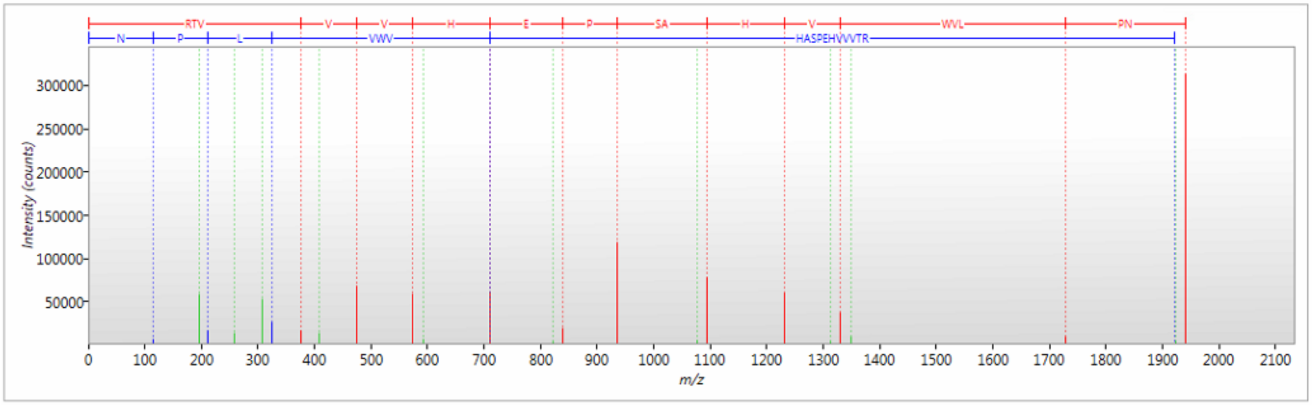

Fragment ions for: QGPVNLLSDPEQGVEVTGQYER

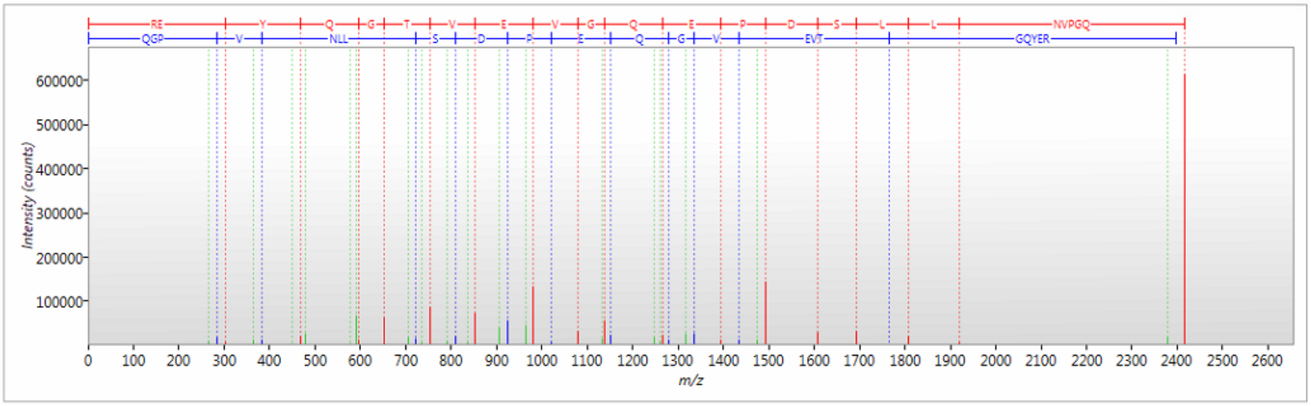

P63267

Actin_ gamma-enteric smooth muscle OS=Homo sapiens GN=ACTG2 PE=1 SV=1
3 peptides

Sequence	Peptide Ion	Score	Hits	Mass	Charge	Tags	Conflicts	Modifications	In quantitation	Average Normalised Abundances			
										PCa	BPH	Bc	Rc
DLYANNVLSGGTTMYPGIADRMQK	3540	5.42	2	2630.2275	3		0	[14] Oxidation M	yes	2.62e+006	2.35e+006	1.66e+006	1.40e+006
IIAPPER	4810	7.87	5	794.4334	2		0		yes	1.92e+004	4.75e+004	1.73e+004	1.76e+004
YSVWIGGSILASLSTFQQMWISKPEYDEAGPSIVHR	19385	6.34	2	4052.0663	4		0		yes	2.17e+005	1.84e+004	1.45e+004	1.78e+005

Fragment ions for: DLYANNVLSGGTTMYPGIADRMQK

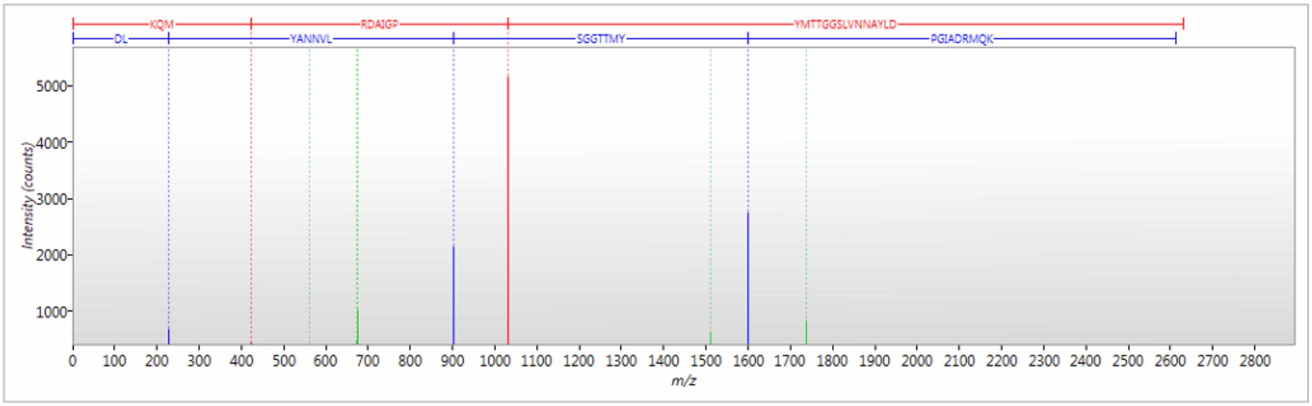

Fragment ions for: IIAPPER

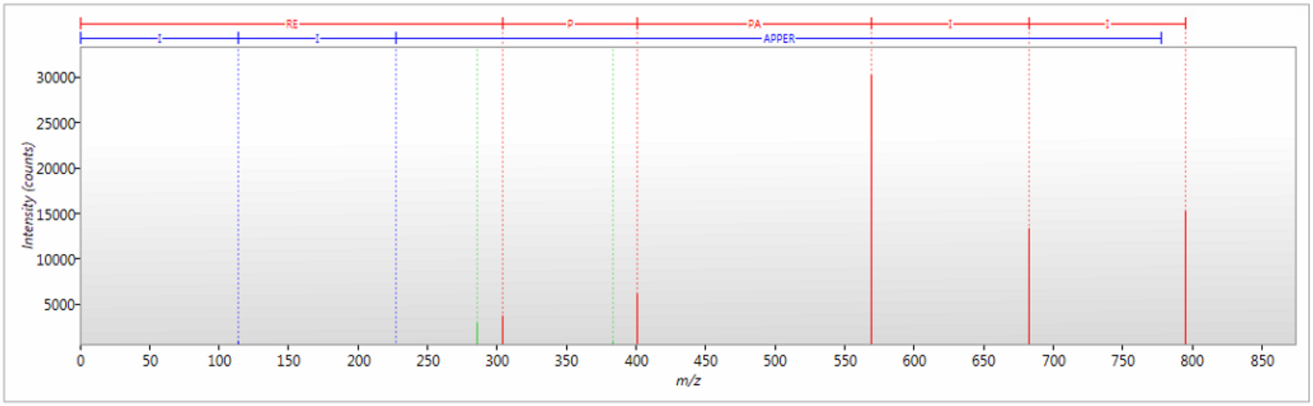

Fragment ions for: YSVWIGGSILASLSTFQQMWISKPEYDEAGPSIVHR

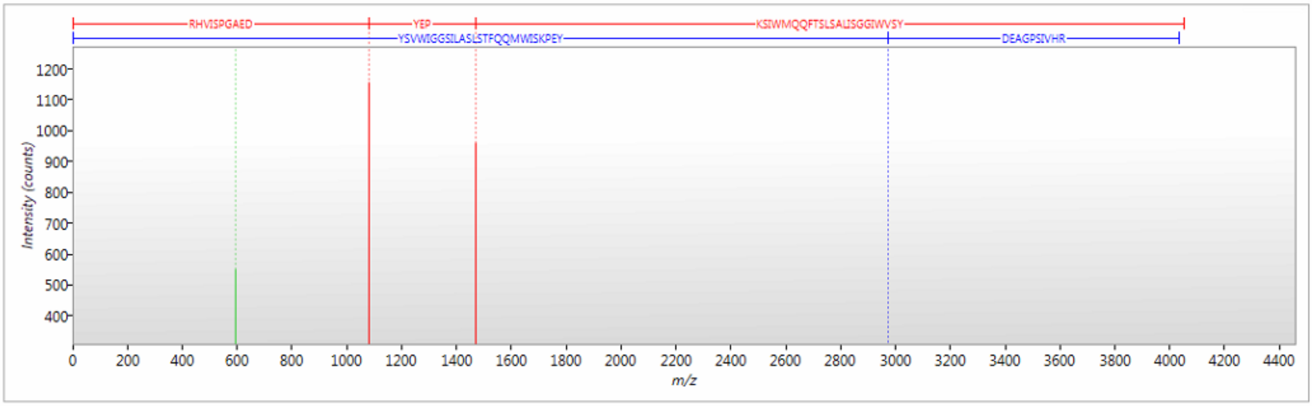

P07858

Cathepsin B OS=Homo sapiens GN=CTSB PE=1 SV=3
3 peptides

Sequence	Peptide Ion	Score	Hits	Mass	Charge	Tags	Conflicts	Modifications	In quantitation	Average Normalised Abundances			
										PCa	BPH	Bc	Rc
DQGSCGSCWAFGAVEAISDR	13303	5.24	2	2172.1343	2		0	[5] Carbamidomethyl C|[8] Carbamidomethyl C	yes	2.55e+004	5.64e+004	7.73e+004	6.56e+004
EQWPQCPTIK	28839	6.47	3	1285.6436	1		0	[6] Carbamidomethyl C	yes	501.68	835.09	4987.48	3536.74
ICEPGYSPTYK	62	7.34	4	1313.6574	2		1	[2] Carbamidomethyl C	no	1.61e+006	3.81e+006	1.63e+007	1.35e+006

Fragment ions for: DQGSCGSCWAFGAVEAISDR

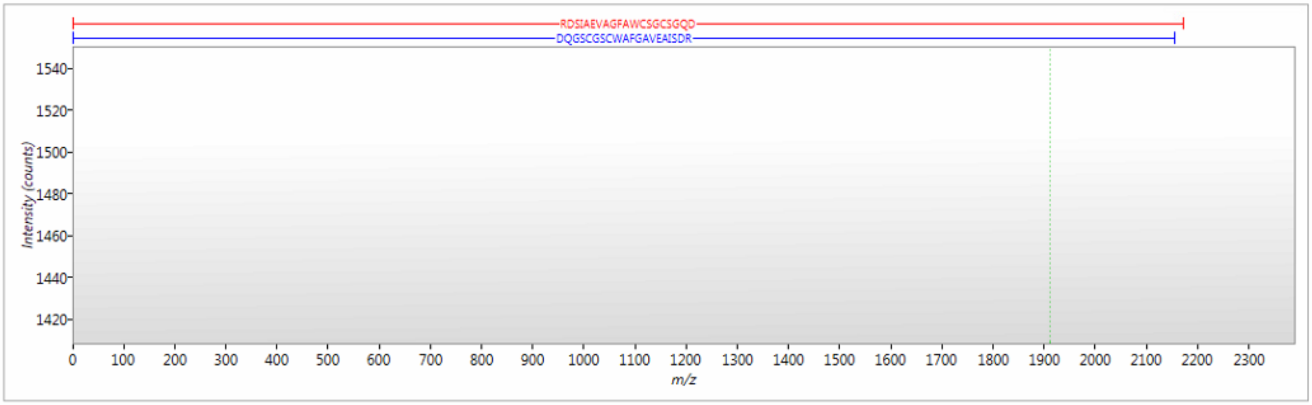

Fragment ions for: EQWPQCPTIK

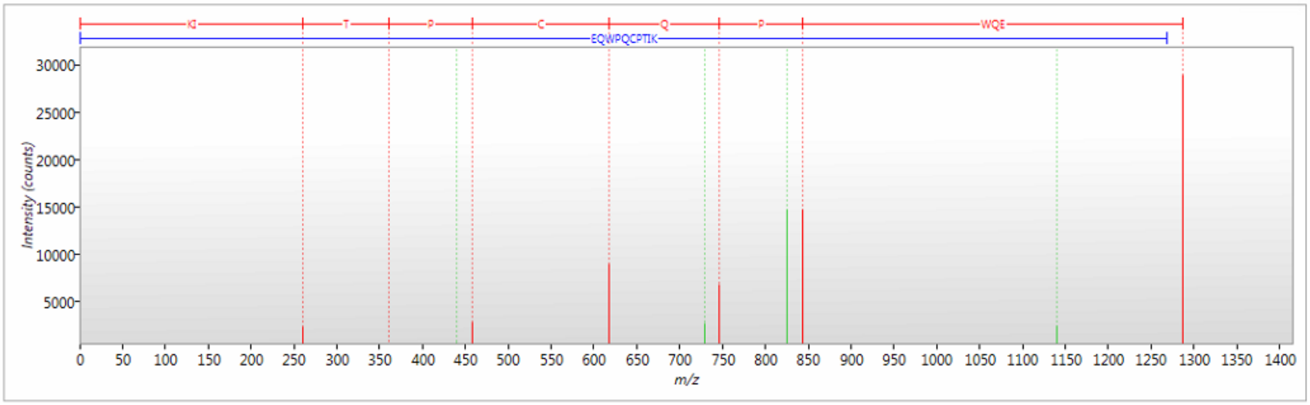

Supplement: Supplementary file 1 [file proteomes-06-00001-s001.zip › proteomes-247986 supplementary 12.27/Supplementary document 2.pdf]
