# Supplementary material for: Comparative Proteomics Analysis of Urine Reveals Down-Regulation of Acute Phase Response Signaling and LXR/RXR Activation Pathways in Prostate Cancer
Source: Proteomes. 2017 Dec 29;6(1):1. doi: 10.3390/proteomes6010001 (PMC5874760; doi:10.3390/proteomes6010001)
Supplement: Supplementary file 1 [file proteomes-06-00001-s001.zip › proteomes-247986 supplementary 12.27/Supplementary Table 4.pdf]

Supplementary Table 4. Functional characterization and association with prostate and other types of malignancy of 20 proteins with differential abundance in PCa identified in this study in more than one group comparison.

Protein name	Gene Name	Protein abundance in PCa in this study	Molecular/biological function(s)	Classification and involvment in disease	Association with PCa (proteomics studies)	Associations with cancer other than PCa (proteomics studies)	Association with cancer (genomics and functional studies)
Monocyte differentiation antigen CD14	CD14	↑	The protein encoded by this gene is a surface antigen that is preferentially expressed on monocytes/ macrophages. It cooperates with other proteins to mediate the innate immune response to bacterial lipopolysaccharide. Alternative splicing results in multiple transcript variants encoding the same protein.		Urinary CD14 bears high potential in differential diagnosis of BPH from the normal as well as from the prostate cancer subjects [1]; Candidate biomarker for PCa in EPS [2]	Serum soluble CD14 is a potential prognostic indicator of recurrence of human breast invasive ductal carcinoma [3]; CD14 is potential serological cancer marker for liver cancer [4]; CD14 were differentially up-regulated in head and neck and breast tumor cells [5]	
Alpha-2-HS-glycoprotein	AHSG	↑	This glycoprotein present in serum is synthesized by hepatocytes. It is involved in several functions, such as endocytosis, brain development and the formation of bone tissue. It has been postulated that it participates in the development of the tissues.		Increased in serum of PCa-non progresing [6]	Increased Complement C3 and AHSG in pancreatic ductal adenocarcinoma [7]; Increased in serum of papillary thyroid cancer [8]; Up-regulated in hypopharyngeal squamous cell carcinoma [9]; Marker for lung carcinoma [10]; Up regulated in plasma of esophageal squamous cell carcinoma patients [11]; Marker for breast and lung cancer [12];	
Alpha-enolase	ENO1	↓	Alpha-enolase is one of three enolase isoenzymes found in mammals. It functions as a glycolytic enzyme and as a structural lens protein (tau-crystallin) in the monomeric form. Alternative splicing of this gene results in a shorter isoform that has been shown to bind to the c-myc promoter and function as a tumor suppressor.	ENO1 overexpression and post-translational modifications could be of diagnostic and prognostic value in many cancer types [13].	Increased in PCa cell lines PC346C and VCap [14]; Stromal ENO1 levels are increased in PCa compared with those in normal tissue [15]; Down-regulated in urine from PCa patients [16];	Upregulated in lung carcinoma [17-19]; Slightly elevated (not significant) in serum and urine of renal cell carcinoma patients [20]; Down-regulation of ENO1 in nasopharyngeal carcinoma [21]; Upregulated in serum in cholangiocarcinoma [22];	ENO1 mRNA and protein levels were upregulated in glioma tissues [23]; ENO1 possesses tumor-suppressing effects in neurocytoma [24] ;
Annexin A1	ANXA1	↑	This gene encodes a membrane-localized protein that binds phospholipids. This protein inhibits phospholipase A2 and has anti-inflammatory activity.	It act as angiogenic factor and can increase the vascular development in the neighbourhood of the tumour [25]	Present in metastasis-derived prostasomes [25];;	Up-regulated in lung carcinoma [26,27]; Tissue marker for lung carcinoma [28].	

Clusterin	CLU	↓	The protein encoded by this gene is a secreted chaperone. It has been suggested to be involved in several basic biological events such as cell death, tumor progression, and neurodegenerative disorders.		Decresed in G7 vs G5, decreased with PCa progression [29]; Decreased with PCa progression [30]; Decreased in PCa [31]	Urine biomarker for lung adenocarcinoma [32]	
Collagen alpha-1(VI) chain	COL6A1	↓	The collagens are a superfamily of proteins that play a role in maintaining the integrity of various tissues. Collagen VI is a major structural component of microfibrils.		Decreased in PCa tissue [33]; Decreased in PCa [31]	Up-regulated in lung cancer [34]; Up regulated in bone metastasis [35];<br>Down regulated in colorectal cancer [36];	Down-regulated in ovarian cancer [37];
Complement C3	C3	↑	Complement component C3 plays a central role in the activation of complement system. Its activation is required for both classical and alternative complement activation pathways.	Mutations in this gene are associated with atypical hemolytic uremic syndrome and age-related macular degeneration in human patients.	Increased in serum of PCa [38]; Increased in G7 vs G5, increased with PCa progression [29];	Up-regulated in pancreatic ductal adenocarcinoma [7]; Increased in serum of lung cancer [39]; Up-regulated in plasma of patients with squamous cell carcinoma of the uterine cervix [40]; Marker for breast and lung cancer [12]; Up regulated in lung cancer [27]; Increased in serum of patients with lung cancer [41]; Increased in serum of patient with neuroblastoma [42,43]; Increased in serum of 200 different cancer patients (colon, pancreas, esophagus, lung, prostate,bladder, ovary, cervix, breast) [44];	Up-regulated in ovarian cancer cells [45];
Fibrinogen alpha chain	FGA	↓	The protein encoded by this gene is the alpha component of fibrinogen. Following vascular injury, fibrinogen is cleaved by thrombin to form fibrin which is the most abundant component of blood clots. In addition, various cleavage products of fibrinogen and fibrin regulate cell adhesion and spreading, display vasoconstrictor and chemotactic activities, and are mitogens for several cell types.	Mutations in this gene lead to several disorders, including dysfibrinogenemia, hypofibrinogenemia, afibrinogenemia and renal amyloidosis.	Up-regulated in urine of PCa patients [46]	Degradation products in urine - markers for bladder cancer [47]; Up-regulated in urine of bladder cancer patients [48]; Down regulated in liver cancer [49]; Up-regulated in the plasma of ovarian cancer patients [50]; Up-regulated in serum of ovarian cancer patients [51]; Up-regulated in plasma of patients with squamous cell carcinoma of the uterine cervix [40]	
Fibrinogen gamma chain	FGG	↓	The protein encoded by this gene is the gama component of fibrinogen.	Mutations in this gene lead to several disorders, including dysfibrinogenemia, hypofibrinogenemia and thrombophilia.	Differentially expressed in PCa tissue [52]; Down-regulated in urine of PCa patients [46]	Degradation products in urine - markers for bladder cancer [47]; Up-regulated in urine of bladder cancer patients [48]; down-regulated in hypopharyngeal squamous cell carcinoma [9]; up-regulated in the hepatocellular carcinoma serum samples [53]; Down regulated in plasma of esophageal squamous cell carcinoma patients [11]	

Hemopexin	HPX	↓	This gene encodes a plasma glycoprotein that binds heme with high affinity. The encoded protein is an acute phase protein that transports heme from the plasma to the liver and may be involved in protecting cells from oxidative stress.	Decreased in serum of PCa patients [38]; Increased in serum of PCa patients [30]	Up-regulated in non-small-cell lung cancer [54]; Down-regulated in plasma of breast cancer patients [55];	Up-regulated in testicular germ cell tumors [56]; Differentially expressed in breast cancer tissues [57];
Prostaglandin-H2 D-isomerase	PTGDS	↑	This protein catalyzes the conversion of prostaglandin H2 (PGH2) to postaglandin D2 (PGD2).	Up-regulated in urine of PCa patients [46,58];	Down-regulated in human cerebrospinal fluid from meningioma [59];	
Protein S100-A9	S100A9	↓	S100A9 is a calcium- and zinc-binding protein which plays a prominent role in the regulation of inflammatory processes and immune response. The extracellular functions involve proinfammatory, antimicrobial, oxidant-scavenging and apoptosis-inducing activities. Its proinflammatory activity includes recruitment of leukocytes, promotion of cytokine and chemokine production, and regulation of leukocyte adhesion and migration.	S100A9 serum levels were significantly elevated in PCa patients compared with BPH or healthy individuals [60]; Overexxpressed in urine from subset of PCa patients but absent in urine from BPH [16]	Up-regulated in human hypopharyngeal cancer cell [61]; Up-regulated in both serum and tissue samples of clear-cell renal cell carcinoma [62]; Up-regulated in hepatocellular carcinoma [63,64]; Up-regulated in serum of colorectal cancer [65]; Up-regulated in serum of bladder cancer [66]; Down-regulated in non-small lung cancer [67]	TSPAN13-to-S100A9 ratio showed a strong potential as a diagnostic marker for PCa [68]; S100A8/A9 mRNA, protein and urinary nucleic acid levels are lower in PCa than in BPH [69];
Vesicular integral-membrane protein VIP36	LMAN2	↑	The encoded protein shuttles between the endoplasmic reticulum, the Golgi apparatus and the plasma membrane. It binds high mannose type glycoproteins and may facilitate their sorting and trafficking.	Up-regulated in urine of PCa patients [46]	Up-regulated in gastric cancer cells [70];	
Inter-alpha-trypsin inhibitor heavy chain H4	ITIH4	↑	The protein encoded by this gene is secreted into the blood, where it is cleaved by plasma kallikrein into two smaller forms. Expression of this gene has been detected only in liver, and it seems to be up-regulated during surgical trauma.	Up-regulated in urine of PCa patients [71]; Down-regulated in serum of PCa patients [72]; Up-regulated in urine of PCa patients [46]; Up-regulated in serum of progresing PCa [6]	Up-regulated in serum peptide biomarkers for breast cancer [73]; up-regulated in serum of gastric cancer patients [74]; Up-regulated in serum of colonic adenomas [75]; Up-regulated in serum of breast cancer patients [76,77]; Up-regulated in liver cancer [49]	
Actin, aortic smooth muscle	ACTA2	↓	The protein encoded by this gene belongs to the actin family of proteins, which are highly conserved proteins that play a role in cell motility, structure and integrity. Alpha, beta and gamma actin isoforms have been identified, with alpha actins being a major constituent of the contractile apparatus, while beta and gamma actins are involved in the regulation of cell motility.	increased ACTA2 observed in extraprostatic extension components of locally advanced PCa [78,79]; decreased in cancer-associated stroma when compared to BPH stroma [80];	A proteomic study identified α-smooth muscle actin (ACTA2) as a PGAM1-associated protein. PGAM1 modulated actin filaments assembly, cell motility and cancer cell migration via directly interacting with ACTA2 [81]; Candidate biomarker for blader cancer [82]; ACTA2 regulates c-MET and FAK expression in lung adenocarcinoma cells, which	mRNA and protein expression levels down-regulated in bladder cancer [84]; aberrant ACTA2 expression accelerated the invasiveness and metastasis of breast cancer cells[85];

					positively and selectively influence metastatic potential [83];	
Granulins	GRN	↓	The 88 kDa precursor protein, progranulin, is also called proepithelin and PC cell-derived growth factor. Cleavage of the signal peptide produces mature granulin which can be further cleaved into a variety of active, 6 kDa peptides. These smaller cleavage products are named granulin A, granulin B, granulin C, etc. Both the peptides and intact granulin protein regulate cell growth. Granulin family members are important in normal development, wound healing, and tumorigenesis.		GRN-A can serve as a prostate cancer serum and tumor marker [86]	Up-regulated in urine of hepatocarcinoma patients [63]; Progranulin is overexpressed in ovarian cancer [87];	Proepithelin is stimulating the migration, invasion, proliferation, and anchorage-independent growth of prostate cancer cells [88];
Hemoglobin subunit beta	HBB	↓	The alpha (HBA) and beta (HBB) loci determine the structure of the 2 types of polypeptide chains in adult hemoglobin. The normal adult hemoglobin tetramer consists of two alpha chains and two beta chains.	Mutant beta globin causes sickle cell anemia. Absence of beta chain causes beta-zero-thalassemia. Reduced amounts of detectable beta globin causes beta-plus-thalassemia.	HBB is selectively deregulated in prostate, breast and lung cancer cells [89];	Down-regulated in colorectal carcinoma [90]; Down-regulated in lung cancer [91]; HBB suppresses lung neuroblastoma cells growth and metastasis [92];	Decreased expression of HBB in anaplastic thyroid cancer [93];
Phosphatidyl ethanolamin e-binding protein 1	PEBP1	↓	This gene encodes a member of the phosphatidylethanolamine-binding family of proteins and has been shown to modulate multiple signaling pathways, including the MAP kinase (MAPK), NF-kappa B, and glycogen synthase kinase-3 (GSK-3) signaling pathways. The encoded protein can be further processed to form a smaller cleavage product, hippocampal cholinergic neurostimulating peptide (HCNP), which may be involved in neural development.	This gene has been implicated in numerous human cancers and may act as a metastasis suppressor gene.	PEBP1 inhibits the migration and invasion of human prostate cancer PC-3M cells [94]; Down-regulated in prostate cancer tissues [95];	Down-regulated in glioblastoma [96]; Loss of PEBP1 expression is observed in many cancers as they progress [97]; Up-regulated in nasopharyngeal carcinoma [98]; PEBP1 is a metastasis suppressor gene of human epithelial ovarian cancer [99];	Up-regulated in nasopharyngeal carcinoma [100]; Up-regulated in breast cancer [101]; mRNA expression was found to be significantly down-regulated in non-small cell lung cancer [102]; reduced PEBP1 mRNA in transitional cell carcinoma of the urinary bladder [103];

Cathepsin B	CTSB	↓	This enzyme is a lysosomal cysteine protease with both endopeptidase and exopeptidase activity that may play a role in protein turnover. It is also known as amyloid precursor protein secretase and is involved in the proteolytic processing of amyloid precursor protein (APP). Multiple pseudogenes of this gene have been identified.	Incomplete proteolytic processing of APP has been suggested to be a causative factor in Alzheimer's disease. Overexpression of CTSB has been associated invasive and metastatic phenotypes in cancers [104].	Up-regulated in prostate cancer biopsy specimens [105];	Up-regulated in cholangiocarcinoma [106]; Up-regulated in Inflammatory breast cancer (IBC) as compared to non-IBC tissues [107]; Up-regulated in late stage colorectal cancer patients with lymph node metastases when compared to early stage patients [108];	Up-regulated in ovarian cancer cell lines [37];
Osteopontin	SPP1	↑	This protein is involved in the attachment of osteoclasts to the mineralized bone matrix. It is secreted and binds hydroxyapatite with high affinity. The osteoclast vitronectin receptor is found in the cell membrane and may be involved in the binding to this protein. This protein is also a cytokine that upregulates expression of interferon-gamma and interleukin-12.		Increased in human prostate cancer specimens and human prostate cancer cell lines LNCaP and C4-2 [109]; Increased gradient of osteopontin expression throughout the stages of murine prostate cancer, beginning from the preneoplastic lesions to distant metastases [110];	Increased in ovarial, endometrium, esophagus, stomach, pancreas, bile duct, liver, colon, kidney, bladder, prostate, head and neck, lung and brain cancers [111]; Increased in advance stage oesophageal squamous cell carcinoma [112]; The osteopontin levels in non-small cell lung cancer were significantly higher compared to those of the controls [113]; Elevated serum levels in patients with pancreatic cancer [114];	Identified as lead marker of colon cancer progression [115]; Significantly higher levels of osteopontin mRNA in PCa tissue [116]; Up regulated gene expression in metastatic PCa [117]; Down-regulation of SPP1 expression by RNAi led to a decline in the malignant phenotype in PCa cells [118,119];

REFERENCES

1. Cheng, H.L.; Huang, H.J.; Ou, B.Y.; Chow, N.H.; Chen, Y.W.; Tzai, T.S.; Wu, C.J.; Chen, S.H. Urinary cd14 as a potential biomarker for benign prostatic hyperplasia - discovery by combining maldi-tof-based biostatistics and esi-ms/ms-based stable-isotope labeling. *Proteomics Clin Appl* **2011**, *5*, 121-132.
2. Li, R.; Guo, Y.; Han, B.M.; Yan, X.; Utleg, A.G.; Li, W.; Tu, L.C.; Wang, J.; Hood, L.; Xia, S.*, et al.* Proteomics cataloging analysis of human expressed prostatic secretions reveals rich source of biomarker candidates. *Proteomics Clin Appl* **2008**, *2*, 543-555.
3. He, W.; Tong, Y.; Wang, Y.; Liu, J.; Luo, G.; Wu, J.; Zhang, J. Serum soluble cd14 is a potential prognostic indicator of recurrence of human breast invasive ductal carcinoma with her2-enriched subtype. *PLoS One* **2013**, *8*, e75366.
4. Wu, C.C.; Hsu, C.W.; Chen, C.D.; Yu, C.J.; Chang, K.P.; Tai, D.I.; Liu, H.P.; Su, W.H.; Chang, Y.S.; Yu, J.S. Candidate serological biomarkers for cancer identified from the secretomes of 23 cancer cell lines and the human protein atlas. *Mol Cell Proteomics* **2010**, *9*, 1100-1117.
5. Hoover, H.; Li, J.; Marchese, J.; Rothwell, C.; Borawoski, J.; Jeffery, D.A.; Gaither, L.A.; Finkel, N. Quantitative proteomic verification of membrane proteins as potential therapeutic targets located in the 11q13 amplicon in cancers. *J Proteome Res* **2015**, *14*, 3670-3679.
6. Rehman, I.; Evans, C.A.; Glen, A.; Cross, S.S.; Eaton, C.L.; Down, J.; Pesce, G.; Phillips, J.T.; Yen, O.S.; Thalmann, G.N.*, et al.* Itraq identification of candidate serum biomarkers associated with metastatic progression of human prostate cancer. *PLoS One* **2012**, *7*, e30885.

7. Chen, J.; Wu, W.; Chen, L.; Zhou, H.; Yang, R.; Hu, L.; Zhao, Y. Profiling the potential tumor markers of pancreatic ductal adenocarcinoma using 2d-dige and maldi-tof-ms: Up-regulation of complement c3 and alpha-2-hs-glycoprotein. *Pancreatology* **2013**, *13*, 290-297.
8. Abdullah, M.I.; Lee, C.C.; Mat Junit, S.; Ng, K.L.; Hashim, O.H. Tissue and serum samples of patients with papillary thyroid cancer with and without benign background demonstrate different altered expression of proteins. *PeerJ* **2016**, *4*, e2450.
9. Tian, W.D.; Li, J.Z.; Hu, S.W.; Peng, X.W.; Li, G.; Liu, X.; Chen, H.H.; Xu, X.; Li, X.P. Proteomic identification of alpha-2-hs-glycoprotein as a plasma biomarker of hypopharyngeal squamous cell carcinoma. *Int J Clin Exp Pathol* **2015**, *8*, 9021-9031.
10. Dowling, P.; O'Driscoll, L.; Meleady, P.; Henry, M.; Roy, S.; Ballot, J.; Moriarty, M.; Crown, J.; Clynes, M. 2-d difference gel electrophoresis of the lung squamous cell carcinoma versus normal sera demonstrates consistent alterations in the levels of ten specific proteins. *Electrophoresis* **2007**, *28*, 4302-4310.
11. Zhao, J.; Fan, Y.X.; Yang, Y.; Liu, D.L.; Wu, K.; Wen, F.B.; Zhang, C.Y.; Zhu, D.Y.; Zhao, S. Identification of potential plasma biomarkers for esophageal squamous cell carcinoma by a proteomic method. *Int J Clin Exp Pathol* **2015**, *8*, 1535-1544.
12. Dowling, P.; Clarke, C.; Hennessy, K.; Torralbo-Lopez, B.; Ballot, J.; Crown, J.; Kiernan, I.; O'Byrne, K.J.; Kennedy, M.J.; Lynch, V.*, et al.* Analysis of acute-phase proteins, ahsg, c3, cli, hp and saa, reveals distinctive expression patterns associated with breast, colorectal and lung cancer. *Int J Cancer* **2012**, *131*, 911-923.
13. Cappello, P.; Principe, M.; Bulfamante, S.; Novelli, F. Alpha-enolase (eno1), a potential target in novel immunotherapies. *Front Biosci (Landmark Ed)* **2017**, *22*, 944-959.
14. Duijvesz, D.; Burnum-Johnson, K.E.; Gritsenko, M.A.; Hoogland, A.M.; Vredenbregt-van den Berg, M.S.; Willemsen, R.; Luider, T.; Pasa-Tolic, L.; Jenster, G. Proteomic profiling of exosomes leads to the identification of novel biomarkers for prostate cancer. *PLoS One* **2013**, *8*, e82589.
15. Yu, L.; Shi, J.; Cheng, S.; Zhu, Y.; Zhao, X.; Yang, K.; Du, X.; Klocker, H.; Yang, X.; Zhang, J. Estrogen promotes prostate cancer cell migration via paracrine release of eno1 from stromal cells. *Mol Endocrinol* **2012**, *26*, 1521-1530.
16. Rehman, I.; Azzouzi, A.R.; Catto, J.W.; Allen, S.; Cross, S.S.; Feeley, K.; Meuth, M.; Hamdy, F.C. Proteomic analysis of voided urine after prostatic massage from patients with prostate cancer: A pilot study. *Urology* **2004**, *64*, 1238-1243.
17. Li, L.S.; Kim, H.; Rhee, H.; Kim, S.H.; Shin, D.H.; Chung, K.Y.; Park, K.S.; Paik, Y.K.; Chang, J. Proteomic analysis distinguishes basaloid carcinoma as a distinct subtype of nonsmall cell lung carcinoma. *Proteomics* **2004**, *4*, 3394-3400.
18. Yu, L.; Shen, J.; Mannoor, K.; Guarnera, M.; Jiang, F. Identification of eno1 as a potential sputum biomarker for early-stage lung cancer by shotgun proteomics. *Clin Lung Cancer* **2014**, *15*, 372-378 e371.
19. Chang, G.C.; Liu, K.J.; Hsieh, C.L.; Hu, T.S.; Charoenfuprasert, S.; Liu, H.K.; Luh, K.T.; Hsu, L.H.; Wu, C.W.; Ting, C.C.*, et al.* Identification of alpha-enolase as an autoantigen in lung cancer: Its overexpression is associated with clinical outcomes. *Clin Cancer Res* **2006**, *12*, 5746-5754.
20. White, N.M.; Masui, O.; Desouza, L.V.; Krakovska, O.; Metias, S.; Romaschin, A.D.; Honey, R.J.; Stewart, R.; Pace, K.; Lee, J.*, et al.* Quantitative proteomic analysis reveals potential diagnostic markers and pathways involved in pathogenesis of renal cell carcinoma. *Oncotarget* **2014**, *5*, 506-518.
21. Yang, J.; Zhou, M.; Zhao, R.; Peng, S.; Luo, Z.; Li, X.; Cao, L.; Tang, K.; Ma, J.; Xiong, W.*, et al.* Identification of candidate biomarkers for the early detection of nasopharyngeal carcinoma by quantitative proteomic analysis. *J Proteomics* **2014**, *109*, 162-175.
22. Rucksaken, R.; Pairojkul, C.; Pinlaor, P.; Khuntikeo, N.; Roytrakul, S.; Selmi, C.; Pinlaor, S. Plasma autoantibodies against heat shock protein 70, enolase 1 and ribonuclease/angiogenin inhibitor 1 as potential biomarkers for cholangiocarcinoma. *PLoS One* **2014**, *9*, e103259.
23. Song, Y.; Luo, Q.; Long, H.; Hu, Z.; Que, T.; Zhang, X.; Li, Z.; Wang, G.; Yi, L.; Liu, Z.*, et al.* Alpha-enolase as a potential cancer prognostic marker promotes cell growth, migration, and invasion in glioma. *Mol Cancer* **2014**, *13*, 65.
24. Sedoris, K.C.; Thomas, S.D.; Miller, D.M. C-myc promoter binding protein regulates the cellular response to an altered glucose concentration. *Biochemistry* **2007**, *46*, 8659-8668.
25. Ronquist, K.G.; Ronquist, G.; Larsson, A.; Carlsson, L. Proteomic analysis of prostate cancer metastasis-derived prostasomes. *Anticancer Res* **2010**, *30*, 285-290.
26. Liu, Y.F.; Chen, Y.H.; Li, M.Y.; Zhang, P.F.; Peng, F.; Li, G.Q.; Xiao, Z.Q.; Chen, Z.C. Quantitative proteomic analysis identifying three annexins as lymph node metastasis-related proteins in lung adenocarcinoma. *Med Oncol* **2012**, *29*, 174-184.
27. Pastor, M.D.; Nogal, A.; Molina-Pinelo, S.; Melendez, R.; Salinas, A.; Gonzalez De la Pena, M.; Martin-Juan, J.; Corral, J.; Garcia-Carbonero, R.; Carnero, A.*, et al.* Identification of proteomic signatures associated with lung cancer and copd. *J Proteomics* **2013**, *89*, 227-237.
28. Li, B.; Chang, J.; Chu, Y.; Kang, H.; Yang, J.; Jiang, J.; Ma, H. Membrane proteomic analysis comparing squamous cell lung cancer tissue and tumour-adjacent normal tissue. *Cancer Lett* **2012**, *319*, 118-124.
29. Byrne, J.C.; Downes, M.R.; O'Donoghue, N.; O'Keane, C.; O'Neill, A.; Fan, Y.; Fitzpatrick, J.M.; Dunn, M.; Watson, R.W. 2d-dige as a strategy to identify serum markers for the progression of prostate cancer. *Journal of proteome research* **2009**, *8*, 942-957.
30. Fan, Y.; Murphy, T.B.; Byrne, J.C.; Brennan, L.; Fitzpatrick, J.M.; Watson, R.W. Applying random forests to identify biomarker panels in serum 2d-dige data for the detection and staging of prostate cancer. *Journal of proteome research* **2011**, *10*, 1361-1373.

31. Sun, C.; Song, C.; Ma, Z.; Xu, K.; Zhang, Y.; Jin, H.; Tong, S.; Ding, W.; Xia, G.; Ding, Q. Periostin identified as a potential biomarker of prostate cancer by itraq-proteomics analysis of prostate biopsy. *Proteome Sci* **2011**, *9*, 22.
32. Zhang, Y.; Li, Y.; Qiu, F.; Qiu, Z. Comparative analysis of the human urinary proteome by 1d sds-page and chip-hplc-ms/ms identification of the aact putative urinary biomarker. *J Chromatogr B Analyt Technol Biomed Life Sci* **2010**, *878*, 3395-3401.
33. Garbis, S.D.; Tyritzis, S.I.; Roumeliotis, T.; Zerefos, P.; Giannopoulou, E.G.; Vlahou, A.; Kossida, S.; Diaz, J.; Vourekas, S.; Tamvakopoulos, C.*, et al.* Search for potential markers for prostate cancer diagnosis, prognosis and treatment in clinical tissue specimens using amine-specific isobaric tagging (itraq) with two-dimensional liquid chromatography and tandem mass spectrometry. *J Proteome Res* **2008**, *7*, 3146-3158.
34. Chiu, K.H.; Chang, Y.H.; Wu, Y.S.; Lee, S.H.; Liao, P.C. Quantitative secretome analysis reveals that col6a1 is a metastasis-associated protein using stacking gel-aided purification combined with itraq labeling. *J Proteome Res* **2011**, *10*, 1110-1125.
35. Blanco, M.A.; LeRoy, G.; Khan, Z.; Aleckovic, M.; Zee, B.M.; Garcia, B.A.; Kang, Y. Global secretome analysis identifies novel mediators of bone metastasis. *Cell Res* **2012**, *22*, 1339-1355.
36. Yang, H.Y.; Kwon, J.; Park, H.R.; Kwon, S.O.; Park, Y.K.; Kim, H.S.; Chung, Y.J.; Chang, Y.J.; Choi, H.I.; Chung, K.J.*, et al.* Comparative proteomic analysis for the insoluble fractions of colorectal cancer patients. *J Proteomics* **2012**, *75*, 3639-3653.
37. Parker, L.P.; Taylor, D.D.; Kesterson, S.; Gercel-Taylor, C. Gene expression profiling in response to estradiol and genistein in ovarian cancer cells. *Cancer Genomics Proteomics* **2009**, *6*, 189-194.
38. Bergamini, S.; Bellei, E.; Reggiani Bonetti, L.; Monari, E.; Cuoghi, A.; Borelli, F.; Sighinolfi, M.C.; Bianchi, G.; Ozben, T.; Tomasi, A. Inflammation: An important parameter in the search of prostate cancer biomarkers. *Proteome Sci* **2014**, *12*, 32.
39. Okano, T.; Kondo, T.; Kakisaka, T.; Fujii, K.; Yamada, M.; Kato, H.; Nishimura, T.; Gemma, A.; Kudoh, S.; Hirohashi, S. Plasma proteomics of lung cancer by a linkage of multi-dimensional liquid chromatography and two-dimensional difference gel electrophoresis. *Proteomics* **2006**, *6*, 3938-3948.
40. Jeong, D.H.; Kim, H.K.; Prince, A.E.; Lee, D.S.; Kim, Y.N.; Han, J.; Kim, K.T. Plasma proteomic analysis of patients with squamous cell carcinoma of the uterine cervix. *J Gynecol Oncol* **2008**, *19*, 173-180.
41. Gminski, J.; Mykala-Ciesla, J.; Machalski, M.; Drozdz, M.; Najda, J. Immunoglobulins and complement components levels in patients with lung cancer. *Rom J Intern Med* **1992**, *30*, 39-44.
42. Carli, M.; Bucolo, C.; Pannunzio, M.T.; Ongaro, G.; Businaro, R.; Revoltella, R. Fluctuation of serum complement levels in children with neuroblastoma. *Cancer* **1979**, *43*, 2399-2404.
43. Kim, P.Y.; Tan, O.; Diakiw, S.M.; Carter, D.; Sekerye, E.O.; Wasinger, V.C.; Liu, T.; Kavallaris, M.; Norris, M.D.; Haber, M.*, et al.* Identification of plasma complement c3 as a potential biomarker for neuroblastoma using a quantitative proteomic approach. *J Proteomics* **2014**, *96*, 1-12.
44. Verhaegen, H.; De Cock, W.; De Cree, J.; Verbruggen, F. Increase of serum complement levels in cancer patients with progressing tumors. *Cancer* **1976**, *38*, 1608-1613.
45. Rupaimoole, R.; Sood, A.; Afshar-Kharghan, V. Complement component 3 (c3) is transcriptionally regulated by twist1. *Blood* **2013**, *122*, 1046-1046.
46. Davalieva, K.; Kiprijanovska, S.; Komina, S.; Petrusevska, G.; Zografska, N.C.; Polenakovic, M. Proteomics analysis of urine reveals acute phase response proteins as candidate diagnostic biomarkers for prostate cancer. *Proteome Sci* **2015**, *13*, 014-0059.
47. Schmetter, B.S.; Habicht, K.K.; Lamm, D.L.; Morales, A.; Bander, N.H.; Grossman, H.B.; Hanna, M.G., Jr.; Silberman, S.R.; Butman, B.T. A multicenter trial evaluation of the fibrin/fibrinogen degradation products test for detection and monitoring of bladder cancer. *J Urol* **1997**, *158*, 801-805.
48. Li, H.; Li, C.; Wu, H.; Zhang, T.; Wang, J.; Wang, S.; Chang, J. Identification of apo-a1 as a biomarker for early diagnosis of bladder transitional cell carcinoma. *Proteome Sci* **2011**, *9*, 21.
49. Li, B.; Guo, T.; Sun, Z.; Li, X.; Wang, H.; Chen, W.; Chen, P.; Qiao, M.; Xia, L.; Mao, Y. Application value of mass spectrometry in the differentiation of benign and malignant liver tumors. *Med Sci Monit* **2017**, *23*, 1636-1644.
50. Ogata, Y.; Heppelmann, C.J.; Charlesworth, M.C.; Madden, B.J.; Miller, M.N.; Kalli, K.R.; Cilby, W.A.; Bergen, H.R., 3rd; Saggese, D.A.; Muddiman, D.C. Elevated levels of phosphorylated fibrinogen-alpha-isoforms and differential expression of other post-translationally modified proteins in the plasma of ovarian cancer patients. *J Proteome Res* **2006**, *5*, 3318-3325.
51. Lee, J.H.; Yoo, B.C.; Kim, Y.H.; Ahn, S.A.; Yeo, S.G.; Cho, J.Y.; Kim, K.H.; Kim, S.C. Low-mass-ion discriminant equation (lome) for ovarian cancer screening. *BioData Min* **2016**, *9*, 32.
52. Alaiya, A.A.; Al-Mohanna, M.; Aslam, M.; Shinwari, Z.; Al-Mansouri, L.; Al-Rodayan, M.; Al-Eid, M.; Ahmad, I.; Hanash, K.; Tulbah, A.*, et al.* Proteomics-based signature for human benign prostate hyperplasia and prostate adenocarcinoma. *Int J Oncol* **2011**, *38*, 1047-1057.

53. Gao, H.J.; Chen, Y.J.; Zuo, D.; Xiao, M.M.; Li, Y.; Guo, H.; Zhang, N.; Chen, R.B. Quantitative proteomic analysis for high-throughput screening of differential glycoproteins in hepatocellular carcinoma serum. *Cancer Biol Med* **2015**, *12*, 246-254.
54. Jin, Y.; Wang, J.; Ye, X.; Su, Y.; Yu, G.; Yang, Q.; Liu, W.; Yu, W.; Cai, J.; Chen, X.*, et al.* Identification of glcnacylated alpha-1-antichymotrypsin as an early biomarker in human non-small-cell lung cancer by quantitative proteomic analysis with two lectins. *Br J Cancer* **2016**, *114*, 532-544.
55. Delmonico, L.; Bravo, M.; Silvestre, R.T.; Ornellas, M.H.; De Azevedo, C.M.; Alves, G. Proteomic profile of saliva and plasma from women with impalpable breast lesions. *Oncol Lett* **2016**, *12*, 2145-2152.
56. Biermann, K.; Heukamp, L.C.; Steger, K.; Zhou, H.; Franke, F.E.; Sonnack, V.; Brehm, R.; Berg, J.; Bastian, P.J.; Muller, S.C.*, et al.* Genome-wide expression profiling reveals new insights into pathogenesis and progression of testicular germ cell tumors. *Cancer Genomics Proteomics* **2007**, *4*, 359-367.
57. Cine, N.; Baykal, A.T.; Sunnetci, D.; Canturk, Z.; Serhatli, M.; Savli, H. Identification of apoa1, hpx and potee genes by omic analysis in breast cancer. *Oncol Rep* **2014**, *32*, 1078-1086.
58. Haj-Ahmad, T.A.; Abdalla, M.A.; Haj-Ahmad, Y. Potential urinary protein biomarker candidates for the accurate detection of prostate cancer among benign prostatic hyperplasia patients. *J Cancer* **2014**, *5*, 103-114.
59. Kim, J.H.; Lee, S.K.; Yoo, Y.C.; Park, N.H.; Park, D.B.; Yoo, J.S.; An, H.J.; Park, Y.M.; Cho, K.G. Proteome analysis of human cerebrospinal fluid as a diagnostic biomarker in patients with meningioma. *Med Sci Monit* **2012**, *18*, BR450-460.
60. Hermani, A.; Hess, J.; De Servi, B.; Medunjanin, S.; Grobholz, R.; Trojan, L.; Angel, P.; Mayer, D. Calcium-binding proteins s100a8 and s100a9 as novel diagnostic markers in human prostate cancer. *Clin Cancer Res* **2005**, *11*, 5146-5152.
61. Wu, P.; Quan, H.; Kang, J.; He, J.; Luo, S.; Xie, C.; Xu, J.; Tang, Y.; Zhao, S. Downregulation of calcium binding protein s100a9 inhibits hypopharyngeal cancer cell proliferation and invasion ability through inactivation of nfkappab signaling. *Oncol Res* **2017**.
62. Zhang, L.; Jiang, H.; Xu, G.; Chu, N.; Xu, N.; Wen, H.; Gu, B.; Liu, J.; Mao, S.; Na, R.*, et al.* Itraq-based quantitative proteomic analysis reveals potential early diagnostic markers of clear-cell renal cell carcinoma. *Biosci Trends* **2016**, *10*, 210-219.
63. Huang, C.H.; Kuo, C.J.; Liang, S.S.; Chi, S.W.; Hsi, E.; Chen, C.C.; Lee, K.T.; Chiou, S.H. Onco-proteogenomics identifies urinary s100a9 and grn as potential combinatorial biomarkers for early diagnosis of hepatocellular carcinoma. *BBA Clin* **2015**, *3*, 205-213.
64. Sun, W.; Xing, B.; Guo, L.; Liu, Z.; Mu, J.; Sun, L.; Wei, H.; Zhao, X.; Qian, X.; Jiang, Y.*, et al.* Quantitative proteomics analysis of tissue interstitial fluid for identification of novel serum candidate diagnostic marker for hepatocellular carcinoma. *Sci Rep* **2016**, *6*, 26499.
65. Fan, N.J.; Chen, H.M.; Song, W.; Zhang, Z.Y.; Zhang, M.D.; Feng, L.Y.; Gao, C.F. Macrophage mannose receptor 1 and s100a9 were identified as serum diagnostic biomarkers for colorectal cancer through a label-free quantitative proteomic analysis. *Cancer Biomark* **2016**, *16*, 235-243.
66. Minami, S.; Sato, Y.; Matsumoto, T.; Kageyama, T.; Kawashima, Y.; Yoshio, K.; Ishii, J.; Matsumoto, K.; Nagashio, R.; Okayasu, I. Proteomic study of sera from patients with bladder cancer: Usefulness of s100a8 and s100a9 proteins. *Cancer Genomics Proteomics* **2010**, *7*, 181-189.
67. Rodriguez-Pineiro, A.M.; Blanco-Prieto, S.; Sanchez-Otero, N.; Rodriguez-Berrocal, F.J.; de la Cadena, M.P. On the identification of biomarkers for non-small cell lung cancer in serum and pleural effusion. *J Proteomics* **2010**, *73*, 1511-1522.
68. Yan, C.; Kim, Y.H.; Kang, H.W.; Seo, S.P.; Jeong, P.; Lee, I.S.; Kim, D.; Kim, J.M.; Choi, Y.H.; Moon, S.K.*, et al.* Urinary nucleic acid tspan13-to-s100a9 ratio as a diagnostic marker in prostate cancer. *J Korean Med Sci* **2015**, *30*, 1784-1792.
69. Yun, S.J.; Yan, C.; Jeong, P.; Kang, H.W.; Kim, Y.H.; Kim, E.A.; Lee, O.J.; Kim, W.T.; Moon, S.K.; Kim, I.Y.*, et al.* Comparison of mrna, protein, and urinary nucleic acid levels of s100a8 and s100a9 between prostate cancer and bph. *Ann Surg Oncol* **2015**, *22*, 2439-2445.
70. Marimuthu, A.; Subbannayya, Y.; Sahasrabuddhe, N.A.; Balakrishnan, L.; Syed, N.; Sekhar, N.R.; Katte, T.V.; Pinto, S.M.; Srikanth, S.M.; Kumar, P.*, et al.* Silac-based quantitative proteomic analysis of gastric cancer secretome. *Proteomics Clin Appl* **2013**, *7*, 355-366.
71. Jayapalan, J.J.; Ng, K.L.; Shuib, A.S.; Razack, A.H.; Hashim, O.H. Urine of patients with early prostate cancer contains lower levels of light chain fragments of inter-alpha-trypsin inhibitor and saposin b but increased expression of an inter-alpha-trypsin inhibitor heavy chain 4 fragment. *Electrophoresis* **2013**, *34*, 1663-1669.
72. Jayapalan, J.J.; Ng, K.L.; Razack, A.H.; Hashim, O.H. Identification of potential complementary serum biomarkers to differentiate prostate cancer from benign prostatic hyperplasia using gel- and lectin-based proteomics analyses. *Electrophoresis* **2012**, *33*, 1855-1862.
73. Yang, J.; Xiong, X.; Liu, S.; Zhu, J.; Luo, M.; Liu, L.; Zhao, L.; Qin, Y.; Song, T.; Huang, C. Identification of novel serum peptides biomarkers for female breast cancer patients in western china. *Proteomics* **2016**, *16*, 925-934.
74. Subbannayya, Y.; Mir, S.A.; Renuse, S.; Manda, S.S.; Pinto, S.M.; Puttamallesh, V.N.; Solanki, H.S.; Manju, H.C.; Syed, N.; Sharma, R.*, et al.* Identification of differentially expressed serum proteins in gastric adenocarcinoma. *J Proteomics* **2015**, *127*, 80-88.

75. Ivancic, M.M.; Irving, A.A.; Jonakin, K.G.; Dove, W.F.; Sussman, M.R. The concentrations of egfr, lrg1, itih4, and f5 in serum correlate with the number of colonic adenomas in apcpirc/+ rats. *Cancer Prev Res (Phila)* **2014**, *7*, 1160-1169.
76. Opstal-van Winden, A.W.; Krop, E.J.; Karedal, M.H.; Gast, M.C.; Lindh, C.H.; Jeppsson, M.C.; Jonsson, B.A.; Grobbee, D.E.; Peeters, P.H.; Beijnen, J.H.*, et al.* Searching for early breast cancer biomarkers by serum protein profiling of pre-diagnostic serum; a nested case-control study. *BMC Cancer* **2011**, *11*, 381.
77. van den Broek, I.; Sparidans, R.W.; van Winden, A.W.; Gast, M.C.; van Dulken, E.J.; Schellens, J.H.; Beijnen, J.H. The absolute quantification of eight inter-alpha-trypsin inhibitor heavy chain 4 (itih4)-derived peptides in serum from breast cancer patients. *Proteomics Clin Appl* **2010**, *4*, 931-939.
78. Chang, L.; Graham, P.H.; Hao, J.; Ni, J.; Bucci, J.; Cozzi, P.J.; Kearsley, J.H.; Li, Y. Acquisition of epithelial-mesenchymal transition and cancer stem cell phenotypes is associated with activation of the pi3k/akt/mtor pathway in prostate cancer radioresistance. *Cell Death Dis* **2013**, *4*, e875.
79. Verrill, C.; Cerundolo, L.; McKee, C.; White, M.; Kartsonaki, C.; Fryer, E.; Morris, E.; Brewster, S.; Ratnayaka, I.; Marsden, L.*, et al.* Altered expression of epithelial-to-mesenchymal transition proteins in extraprostatic prostate cancer. *Oncotarget* **2016**, *7*, 1107-1119.
80. Yu, Y.; Yang, O.; Fazli, L.; Rennie, P.S.; Gleave, M.E.; Dong, X. Progesterone receptor expression during prostate cancer progression suggests a role of this receptor in stromal cell differentiation. *Prostate* **2015**, *75*, 1043-1050.
81. Zhang, D.; Jin, N.; Sun, W.; Li, X.; Liu, B.; Xie, Z.; Qu, J.; Xu, J.; Yang, X.; Su, Y.*, et al.* Phosphoglycerate mutase 1 promotes cancer cell migration independent of its metabolic activity. *Oncogene* **2017**, *36*, 2900-2909.
82. Wu, Z.; Wang, S.; Jiang, F.; Li, Q.; Wang, C.; Wang, H.; Zhang, W.; Xue, P. Mass spectrometric detection combined with bioinformatic analysis identified possible protein markers and key pathways associated with bladder cancer. *Gene* **2017**.
83. Lee, H.W.; Park, Y.M.; Lee, S.J.; Cho, H.J.; Kim, D.H.; Lee, J.I.; Kang, M.S.; Seol, H.J.; Shim, Y.M.; Nam, D.H.*, et al.* Alpha-smooth muscle actin (acta2) is required for metastatic potential of human lung adenocarcinoma. *Clin Cancer Res* **2013**, *19*, 5879-5889.
84. Wang, Y.; Wang, G.; Zhang, X.; Zhou, X.; Liu, Z.; Huang, L.; Liu, R.; Lang, B.; Xu, X.; Liu, W.*, et al.* Gamma-secretase inhibitor inhibits bladder cancer cell drug resistance and invasion by reducing epithelial-mesenchymal transition. *Mol Med Rep* **2015**, *12*, 2821-2827.
85. Jeon, M.; You, D.; Bae, S.Y.; Kim, S.W.; Nam, S.J.; Kim, H.H.; Kim, S.; Lee, J.E. Dimerization of egfr and her2 induces breast cancer cell motility through stat1-dependent acta2 induction. *Oncotarget* **2016**.
86. Deftos, L.J.; Abrahamsson, P.A. Granins and prostate cancer. *Urology* **1998**, *51*, 141-145.
87. Cuevas-Antonio, R.; Cancino, C.; Arechavaleta-Velasco, F.; Andrade, A.; Barron, L.; Estrada, I.; Fernandez, R.L.; Olguin, V.; Ruiz, S.; Imani, F.*, et al.* Expression of progranulin (acrogranin/pcdgf/granulin-epithelin precursor) in benign and malignant ovarian tumors and activation of mapk signaling in ovarian cancer cell line. *Cancer Invest* **2010**, *28*, 452-458.
88. Monami, G.; Emiliozzi, V.; Bitto, A.; Lovat, F.; Xu, S.Q.; Goldoni, S.; Fassan, M.; Serrero, G.; Gomella, L.G.; Baffa, R.*, et al.* Proepithelin regulates prostate cancer cell biology by promoting cell growth, migration, and anchorage-independent growth. *Am J Pathol* **2009**, *174*, 1037-1047.
89. Zheng, Y.; Miyamoto, D.T.; Wittner, B.S.; Sullivan, J.P.; Aceto, N.; Jordan, N.V.; Yu, M.; Karabacak, N.M.; Comaills, V.; Morris, R.*, et al.* Expression of beta-globin by cancer cells promotes cell survival during blood-borne dissemination. *Nat Commun* **2017**, *8*, 14344.
90. Gan, Y.; Chen, D.; Li, X. Proteomic analysis reveals novel proteins associated with progression and differentiation of colorectal carcinoma. *J Cancer Res Ther* **2014**, *10*, 89-96.
91. Rho, J.H.; Roehrl, M.H.; Wang, J.Y. Glycoproteomic analysis of human lung adenocarcinomas using glycoarrays and tandem mass spectrometry: Differential expression and glycosylation patterns of vimentin and fetuin a isoforms. *Protein J* **2009**, *28*, 148-160.
92. Maman, S.; Sagi-Assif, O.; Yuan, W.; Ginat, R.; Meshel, T.; Zubrilov, I.; Keisari, Y.; Lu, W.; Witz, I.P. The beta subunit of hemoglobin (hbb2/hbb) suppresses neuroblastoma growth and metastasis. *Cancer Res* **2017**, *77*, 14-26.
93. Onda, M.; Akaishi, J.; Asaka, S.; Okamoto, J.; Miyamoto, S.; Mizutani, K.; Yoshida, A.; Ito, K.; Emi, M. Decreased expression of haemoglobin beta (hbb) gene in anaplastic thyroid cancer and recovery of its expression inhibits cell growth. *Br J Cancer* **2005**, *92*, 2216-2224.
94. Xinzhou, H.; Ning, Y.; Ou, W.; Xiaodan, L.; Fumin, Y.; Huitu, L.; Wei, Z. Rkip inhibits the migration and invasion of human prostate cancer pc-3m cells through regulation of extracellular matrix. *Mol Biol (Mosk)* **2011**, *45*, 1004-1011.
95. Lu, S.; Zhong, K.; Zhu, L.; He, L. [expression of raf kinase inhibitor protein and e-cadherin in prostate cancer tissues]. *Zhong Nan Da Xue Xue Bao Yi Xue Ban* **2009**, *34*, 892-897.
96. Gimenez, M.; Marie, S.K.; Oba-Shinjo, S.; Uno, M.; Izumi, C.; Oliveira, J.B.; Rosa, J.C. Quantitative proteomic analysis shows differentially expressed hspb1 in glioblastoma as a discriminating short from long survival factor and nova1 as a differentiation factor between low-grade astrocytoma and oligodendroglioma. *BMC Cancer* **2015**, *15*, 481.
97. Lamiman, K.; Keller, J.M.; Mizokami, A.; Zhang, J.; Keller, E.T. Survey of raf kinase inhibitor protein (rkip) in multiple cancer types. *Crit Rev Oncog* **2014**, *19*, 455-468.
98. Chen, Y.; Ouyang, G.L.; Yi, H.; Li, M.Y.; Zhang, P.F.; Li, C.; Li, J.L.; Liu, Y.F.; Chen, Z.C.; Xiao, Z.Q. Identification of rkip as an invasion suppressor protein in nasopharyngeal carcinoma by proteomic analysis. *J Proteome Res* **2008**, *7*, 5254-5262.

99. Li, H.Z.; Wang, Y.; Gao, Y.; Shao, J.; Zhao, X.L.; Deng, W.M.; Liu, Y.X.; Yang, J.; Yao, Z. Effects of raf kinase inhibitor protein expression on metastasis and progression of human epithelial ovarian cancer. *Mol Cancer Res* **2008**, *6*, 917-928.
100. He, Q.Y.; Yi, H.M.; Yi, H.; Xiao, T.; Qu, J.Q.; Yuan, L.; Zhu, J.F.; Li, J.Y.; Wang, Y.Y.; Li, L.N.*, et al.* Reduction of rkip expression promotes nasopharyngeal carcinoma invasion and metastasis by activating stat3 signaling. *Oncotarget* **2015**, *6*, 16422-16436.
101. Hagan, S.; Al-Mulla, F.; Mallon, E.; Oien, K.; Ferrier, R.; Gusterson, B.; Garcia, J.J.; Kolch, W. Reduction of raf-1 kinase inhibitor protein expression correlates with breast cancer metastasis. *Clin Cancer Res* **2005**, *11*, 7392-7397.
102. Wang, Q.; Wu, X.; Wu, T.; Li, G.M.; Shi, Y. Clinical significance of rkip mrna expression in non-small cell lung cancer. *Tumour Biol* **2014**, *35*, 4377-4380.
103. Zaravinos, A.; Chatziioannou, M.; Lambrou, G.I.; Boulalas, I.; Delakas, D.; Spandidos, D.A. Implication of raf and rkip genes in urinary bladder cancer. *Pathol Oncol Res* **2011**, *17*, 181-190.
104. Gondi, C.S.; Rao, J.S. Cathepsin b as a cancer target. *Expert Opin Ther Targets* **2013**, *17*, 281-291.
105. Qian, J.; Bostwick, D.G.; Iczkowski, K.A.; Betre, K.; Wilson, M.J.; Le, C.; Sinha, A.A. Characterization of prostate cancer in needle biopsy by cathepsin b, cell proliferation and DNA ploidy. *Anticancer Res* **2010**, *30*, 719-725.
106. Kawase, H.; Fujii, K.; Miyamoto, M.; Kubota, K.C.; Hirano, S.; Kondo, S.; Inagaki, F. Differential lc-ms-based proteomics of surgical human cholangiocarcinoma tissues. *J Proteome Res* **2009**, *8*, 4092-4103.
107. Nouh, M.A.; Mohamed, M.M.; El-Shinawi, M.; Shaalan, M.A.; Cavallo-Medved, D.; Khaled, H.M.; Sloane, B.F. Cathepsin b: A potential prognostic marker for inflammatory breast cancer. *J Transl Med* **2011**, *9*, 1.
108. Abdulla, M.H.; Valli-Mohammed, M.A.; Al-Khayal, K.; Al Shkieh, A.; Zubaidi, A.; Ahmad, R.; Al-Saleh, K.; Al-Obeed, O.; McKerrow, J. Cathepsin b expression in colorectal cancer in a middle east population: Potential value as a tumor biomarker for late disease stages. *Oncol Rep* **2017**, *37*, 3175-3180.
109. Thalmann, G.N.; Sikes, R.A.; Devoll, R.E.; Kiefer, J.A.; Markwalder, R.; Klima, I.; Farach-Carson, C.M.; Studer, U.E.; Chung, L.W. Osteopontin: Possible role in prostate cancer progression. *Clin Cancer Res* **1999**, *5*, 2271-2277.
110. Khodavirdi, A.C.; Song, Z.; Yang, S.; Zhong, C.; Wang, S.; Wu, H.; Pritchard, C.; Nelson, P.S.; Roy-Burman, P. Increased expression of osteopontin contributes to the progression of prostate cancer. *Cancer Res* **2006**, *66*, 883-888.
111. Coppola, D.; Szabo, M.; Boulware, D.; Muraca, P.; Alsarraj, M.; Chambers, A.F.; Yeatman, T.J. Correlation of osteopontin protein expression and pathological stage across a wide variety of tumor histologies. *Clin Cancer Res* **2004**, *10*, 184-190.
112. Kita, Y.; Natsugoe, S.; Okumura, H.; Matsumoto, M.; Uchikado, Y.; Setoyama, T.; Owaki, T.; Ishigami, S.; Aikou, T. Expression of osteopontin in oesophageal squamous cell carcinoma. *Br J Cancer* **2006**, *95*, 634-638.
113. Zhang, H.; Liu, H.B.; Yuan, D.M.; Wang, Z.F.; Wang, Y.F.; Song, Y. Prognostic value of secreted phosphoprotein-1 in pleural effusion associated with non-small cell lung cancer. *BMC Cancer* **2014**, *14*, 280.
114. Koopmann, J.; Fedarko, N.S.; Jain, A.; Maitra, A.; Iacobuzio-Donahue, C.; Rahman, A.; Hruban, R.H.; Yeo, C.J.; Goggins, M. Evaluation of osteopontin as biomarker for pancreatic adenocarcinoma. *Cancer Epidemiol Biomarkers Prev* **2004**, *13*, 487-491.
115. Agrawal, D.; Chen, T.; Irby, R.; Quackenbush, J.; Chambers, A.F.; Szabo, M.; Cantor, A.; Coppola, D.; Yeatman, T.J. Osteopontin identified as lead marker of colon cancer progression, using pooled sample expression profiling. *J Natl Cancer Inst* **2002**, *94*, 513-521.
116. Tilli, T.M.; Thuler, L.C.; Matos, A.R.; Coutinho-Camillo, C.M.; Soares, F.A.; da Silva, E.A.; Neves, A.F.; Goulart, L.R.; Gimba, E.R. Expression analysis of osteopontin mrna splice variants in prostate cancer and benign prostatic hyperplasia. *Exp Mol Pathol* **2012**, *92*, 13-19.
117. Chandran, U.R.; Ma, C.; Dhir, R.; Bisceglia, M.; Lyons-Weiler, M.; Liang, W.; Michalopoulos, G.; Becich, M.; Monzon, F.A. Gene expression profiles of prostate cancer reveal involvement of multiple molecular pathways in the metastatic process. *BMC Cancer* **2007**, *7*, 64.
118. Zhang, Y.; Forootan, S.S.; Kamalian, L.; Bao, Z.Z.; Malki, M.I.; Foster, C.S.; Ke, Y. Suppressing tumourigenicity of prostate cancer cells by inhibiting osteopontin expression. *Int J Oncol* **2011**, *38*, 1083-1091.
119. Zheng, J.; Hou, Z.B.; Jiao, N.L. Effects of osteopontin downregulation on the growth of prostate cancer pc-3 cells. *Mol Med Rep* **2011**, *4*, 1225-1231.
