# Supplementary material for: Comparative Proteomics Analysis of Urine Reveals Down-Regulation of Acute Phase Response Signaling and LXR/RXR Activation Pathways in Prostate Cancer
Source: Proteomes. 2017 Dec 29;6(1):1. doi: 10.3390/proteomes6010001 (PMC5874760; doi:10.3390/proteomes6010001)
Supplement: Supplementary file 1 [file proteomes-06-00001-s001.zip › proteomes-247986 supplementary 12.27/Supplementary Table 5.pdf]

Supplementary Table 5. Functional characterization and association with malignancy of 9 proteins with differential abundance in PCa identified in this study in more than one group comparison.

Protein name	Gene Name	Protein abundance in PCa in this study	Molecular/biological function(s)	Classification and involvement in disease	Association with PCa (proteomics studies)	Associations with cancer other than PCa (proteomics studies)	Association with cancer (genomics and functional studies)
Azurocidin	AZU1	↓	This gene encodes a preproprotein that is proteolytically processed to generate a mature azurophil granule antibiotic protein, with monocyte chemotactic and antimicrobial activity. It is also an important multifunctional inflammatory mediator.			Up-regulated in gastric cancer [1];	Potential biomarker for myelofibrosis [2];
Ig gamma-1 chain C region	IGHG1	↓	/		/	Up regulated in pancreatic cancer tissue [3]; Differentially expressed in serum samples from glioma patients [4]; Up regulated in human pancreatic carcinomas [5]; Down-regulated in infiltrating ductal carcinomas of the breast [6];	IgG silencing induces apoptosis and suppresses proliferation, migration and invasion in LNCaP prostate cancer cells [7];
Non-secretory ribonuclease	RNASE2	↑	The protein encoded by this gene is a non-secretory ribonuclease that belongs to the pancreatic ribonuclease family, a subset of the ribonuclease A superfamily. The protein antimicrobial activity against viruses.				Up-regulated in colorectal cancer [8]; Up-regulated in childhood acute lymphoblastic leukemia [9];
Pregnancy zone protein	PZP	↓	/			Down-regulated in malignant breast cancer cell lines [10,11];	Up-regulated in esophageal cancer [12];
Lithostathine-1-alpha	REG1A	↑	This gene is a type I subclass member of the Reg gene family. The Reg gene family is a multigene family grouped into four subclasses, types I, II, III and IV, based on the primary structures of the encoded proteins. This gene encodes a protein that is secreted by the exocrine pancreas. It is associated with islet cell regeneration and diabetogenesis			Up regulated in bladder cancer tissues compared with adjacent normal samples [13]; Up regulated in serum of pancreatic ductal adenocarcinoma patients compared to healthy controls [14]; Up regulated in cancer pancreatic ductal fluid compared to normal [15]; Biomarker for early pancreatic lesions [16]; Up regulated in gastric cancer tissues [17]; Up regulated in breast cancer tissues [18];	Overexpressed in non-small cell lung cancer [19]; Overexpressed in colorectal cancer [20,21];

			and may be involved in pancreatic lithogenesis.			
Alpha-amylase 1	AMY1A	↑	This gene encodes an amylase isoenzyme produced by the salivary gland. It catalyzes the first step in digestion of starch and glycogen.		Up regulated in lung cancer [22];	Up regulated in lung cancer [23];
Pancreatic alpha-amylase	AMY2A	↑	This gene encodes a member of the alpha-amylase family of proteins produced by the pancreas.		Up regulated in lung cancer [22];	Candidate tumor-suppressor gene loss in gastric carcinoma [24]; Marker for lung cancer [25]; Up regulated in lung cancer [23];
Actin_ gamma-enteric smooth muscle	ACTG2	↑	The beta and gamma actins co-exist in most cell types as components of the cytoskeleton and as mediators of internal cell motility. This gene encodes actin gamma 2; a smooth muscle actin found in enteric tissues. Alternative splicing results in multiple transcript variants encoding distinct isoforms.		Up regulated in hepatocellular carcinoma and strongly correlated with the epithelial-mesenchymal transition process [26]; Biomarker for leiomyosarcomas [27];	Biomarker for breast cancer [28]; Biomarker for colon cancer [29]; Biomarker for estimating chemotherapy sensitivity of colorectal cancer [30]; Differentially expressed in osteosarcoma [31]; Diagnostic marker for malignant pleural mesothelioma [32]; Marker for cisplatin resistance in breast cancer [33];
Collagen alpha-1(XVIII) chain	COL18A 1	↑	This gene encodes the alpha chain of type XVIII collagen. This collagen is one of the multiplexins, extracellular matrix proteins that contain multiple triple-helix domains (collagenous domains) interrupted by non-collagenous domains. A long isoform of the protein has an N-terminal domain that is homologous to the extracellular part of frizzled receptors. Proteolytic processing at several endogenous cleavage sites in the C-terminal domain results in production of endostatin, a potent antiangiogenic protein that is able to inhibit angiogenesis and tumor growth. Alternative splicing results in multiple transcript variants.	Mutations in this gene are associated with Knobloch syndrome. The main features of this syndrome involve retinal abnormalities, so type XVIII collagen may play an important role in retinal structure and in neural tube closure.	Up regulated in cutaneous squamous cell carcinoma [34];	Differentially expressed between the chromophobe renal cell carcinoma and normal tissues [35]; mRNA levels of collagen XVIII are decreased in liver cancer [36]; Hepatocellular carcinoma is associated with a significant decrease in the expression of the collagen XVIII [37]; Down regulated in metastasis melanomas [38];

REFERENCES

1. Ran, X.; Xu, X.; Yang, Y.; She, S.; Yang, M.; Li, S.; Peng, H.; Ding, X.; Hu, H.; Hu, P., *et al.* A quantitative proteomics study on olfactomedin 4 in the development of gastric cancer. *Int J Oncol* **2015**, *47*, 1932-1944.
2. Hasselbalch, H.C.; Skov, V.; Stauffer Larsen, T.; Thomassen, M.; Hasselbalch Riley, C.; Jensen, M.K.; Bjerrum, O.W.; Kruse, T.A. Transcriptional profiling of whole blood identifies a unique 5-gene signature for myelofibrosis and imminent myelofibrosis transformation. *PLoS One* **2014**, *9*, e85567.
3. Chen, J.H.; Ni, R.Z.; Xiao, M.B.; Guo, J.G.; Zhou, J.W. Comparative proteomic analysis of differentially expressed proteins in human pancreatic cancer tissue. *Hepatobiliary Pancreat Dis Int* **2009**, *8*, 193-200.
4. Syed, P.; Gupta, S.; Choudhary, S.; Pandala, N.G.; Atak, A.; Richharia, A.; K, P.M.; Zhu, H.; Epari, S.; Noronha, S.B., *et al.* Autoantibody profiling of glioma serum samples to identify biomarkers using human proteome arrays. *Sci Rep* **2015**, *5*, 13895.
5. Li, X.; Ni, R.; Chen, J.; Liu, Z.; Xiao, M.; Jiang, F.; Lu, C. The presence of ighg1 in human pancreatic carcinomas is associated with immune evasion mechanisms. *Pancreas* **2011**, *40*, 753-761.
6. Kabbage, M.; Chahed, K.; Hamrita, B.; Guillier, C.L.; Trimeche, M.; Remadi, S.; Hoebeke, J.; Chouchane, L. Protein alterations in infiltrating ductal carcinomas of the breast as detected by nonequilibrium ph gradient electrophoresis and mass spectrometry. *J Biomed Biotechnol* **2008**, *2008*, 564127.
7. Xu, Y.; Chen, B.; Zheng, S.; Wen, Y.; Xu, A.; Xu, K.; Li, B.; Liu, C. Igg silencing induces apoptosis and suppresses proliferation, migration and invasion in lncap prostate cancer cells. *Cell Mol Biol Lett* **2016**, *21*, 27.
8. Kim, S.T.; Sohn, I.; Do, I.G.; Jang, J.; Kim, S.H.; Jung, I.H.; Park, J.O.; Park, Y.S.; Talasaz, A.; Lee, J., *et al.* Transcriptome analysis of cd133-positive stem cells and prognostic value of survivin in colorectal cancer. *Cancer Genomics Proteomics* **2014**, *11*, 259-266.
9. Niini, T.; Vettenranta, K.; Hollmen, J.; Larramendy, M.L.; Aalto, Y.; Wikman, H.; Nagy, B.; Seppanen, J.K.; Ferrer Salvador, A.; Mannila, H., *et al.* Expression of myeloid-specific genes in childhood acute lymphoblastic leukemia - a cdna array study. *Leukemia* **2002**, *16*, 2213-2221.
10. Liang, X.; Huuskonen, J.; Hajivandi, M.; Manzanedo, R.; Predki, P.; Amshey, J.R.; Pope, R.M. Identification and quantification of proteins differentially secreted by a pair of normal and malignant breast-cancer cell lines. *Proteomics* **2009**, *9*, 182-193.
11. Liang, X.; Zhao, J.; Hajivandi, M.; Wu, R.; Tao, J.; Amshey, J.W.; Pope, R.M. Quantification of membrane and membrane-bound proteins in normal and malignant breast cancer cells isolated from the same patient with primary breast carcinoma. *J Proteome Res* **2006**, *5*, 2632-2641.
12. Singh, V.; Singh, L.C.; Vasudevan, M.; Chattopadhyay, I.; Borthakar, B.B.; Rai, A.K.; Phukan, R.K.; Sharma, J.; Mahanta, J.; Kataki, A.C., *et al.* Esophageal cancer epigenomics and integrome analysis of genome-wide methylation and expression in high risk northeast indian population. *OMICS* **2015**, *19*, 688-699.
13. Geng, J.; Fan, J.; Wang, Q.; Zhang, X.P.; Kang, L.; Li, Q.Y.; Xu, Y.F.; Peng, B.; Zheng, J.H.; Yao, X.D. Decreased reg1alpha expression suppresses growth, invasion and angiogenesis of bladder cancer. *Eur J Surg Oncol* **2017**, *43*, 837-846.
14. Li, Q.; Wang, H.; Zogopoulos, G.; Shao, Q.; Dong, K.; Lv, F.; Nwilati, K.; Gui, X.Y.; Cuggia, A.; Liu, J.L., *et al.* Reg proteins promote acinar-to-ductal metaplasia and act as novel diagnostic and prognostic markers in pancreatic ductal adenocarcinoma. *Oncotarget* **2016**, *7*, 77838-77853.
15. Porterfield, M.; Zhao, P.; Han, H.; Cunningham, J.; Aoki, K.; Von Hoff, D.D.; Demeure, M.J.; Pierce, J.M.; Tiemeyer, M.; Wells, L. Discrimination between adenocarcinoma and normal pancreatic ductal fluid by proteomic and glycomic analysis. *J Proteome Res* **2014**, *13*, 395-407.
16. Park, J.; Yun, H.S.; Lee, K.H.; Lee, K.T.; Lee, J.K.; Lee, S.Y. Discovery and validation of biomarkers that distinguish mucinous and nonmucinous pancreatic cysts. *Cancer Res* **2015**, *75*, 3227-3235.
17. Hara, K.; Fukui, H.; Sun, C.; Kitayama, Y.; Eda, H.; Yamasaki, T.; Kondo, T.; Tomita, T.; Oshima, T.; Watari, J., *et al.* Effect of reg ialpha protein on angiogenesis in gastric cancer tissues. *Oncol Rep* **2015**, *33*, 2183-2189.
18. Sasaki, Y.; Minamiya, Y.; Takahashi, N.; Nakagawa, T.; Katayose, Y.; Ito, A.; Saito, H.; Motoyama, S.; Ogawa, J. Reg1a expression is an independent factor predictive of poor prognosis in patients with breast cancer. *Ann Surg Oncol* **2008**, *15*, 3244-3251.

19. Minamiya, Y.; Kawai, H.; Saito, H.; Ito, M.; Hosono, Y.; Motoyama, S.; Katayose, Y.; Takahashi, N.; Ogawa, J. Reg1a expression is an independent factor predictive of poor prognosis in patients with non-small cell lung cancer. *Lung Cancer* **2008**, *60*, 98-104.
20. Bishnupuri, K.S.; Luo, Q.; Korzenik, J.R.; Henderson, J.O.; Houchen, C.W.; Anant, S.; Dieckgraefe, B.K. Dysregulation of reg gene expression occurs early in gastrointestinal tumorigenesis and regulates anti-apoptotic genes. *Cancer Biol Ther* **2006**, *5*, 1714-1720.
21. Astrosini, C.; Roeefzaad, C.; Dai, Y.Y.; Dieckgraefe, B.K.; Jons, T.; Kemmner, W. Reg1a expression is a prognostic marker in colorectal cancer and associated with peritoneal carcinomatosis. *Int J Cancer* **2008**, *123*, 409-413.
22. Pastor, M.D.; Nogal, A.; Molina-Pinelo, S.; Melendez, R.; Salinas, A.; Gonzalez De la Pena, M.; Martin-Juan, J.; Corral, J.; Garcia-Carbonero, R.; Carnero, A., *et al.* Identification of proteomic signatures associated with lung cancer and copd. *J Proteomics* **2013**, *89*, 227-237.
23. Seyama, K.; Nukiwa, T.; Takahashi, K.; Takahashi, H.; Kira, S. Amylase mrna transcripts in normal tissues and neoplasms: The implication of different expressions of amylase isogenes. *J Cancer Res Clin Oncol* **1994**, *120*, 213-220.
24. Kang, J.U.; Koo, S.H.; Kwon, K.C.; Park, J.W. Amy2a: A possible tumor-suppressor gene of 1p21.1 loss in gastric carcinoma. *Int J Oncol* **2010**, *36*, 1429-1435.
25. Watanabe, T.; Miura, T.; Degawa, Y.; Fujita, Y.; Inoue, M.; Kawaguchi, M.; Furihata, C. Comparison of lung cancer cell lines representing four histopathological subtypes with gene expression profiling using quantitative real-time pcr. *Cancer Cell Int* **2010**, *10*, 2.
26. Benzoubir, N.; Mussini, C.; Lejamtel, C.; Dos Santos, A.; Guillaume, C.; Desterke, C.; Samuel, D.; Brechot, C.; Bourgeade, M.F.; Guettier, C. Gamma-smooth muscle actin expression is associated with epithelial-mesenchymal transition and stem-like properties in hepatocellular carcinoma. *PLoS One* **2015**, *10*, e0130559.
27. Demicco, E.G.; Boland, G.M.; Brewer Savannah, K.J.; Lusby, K.; Young, E.D.; Ingram, D.; Watson, K.L.; Bailey, M.; Guo, X.; Hornick, J.L., *et al.* Progressive loss of myogenic differentiation in leiomyosarcoma has prognostic value. *Histopathology* **2015**, *66*, 627-638.
28. Zhang, C.; Zhao, H.; Li, J.; Liu, H.; Wang, F.; Wei, Y.; Su, J.; Zhang, D.; Liu, T.; Zhang, Y. The identification of specific methylation patterns across different cancers. *PLoS One* **2015**, *10*, e0120361.
29. Drew, J.E.; Farquharson, A.J.; Mayer, C.D.; Vase, H.F.; Coates, P.J.; Steele, R.J.; Carey, F.A. Predictive gene signatures: Molecular markers distinguishing colon adenomatous polyp and carcinoma. *PLoS One* **2014**, *9*, e113071.
30. Lu, X.; Pan, J.; Li, S.; Shen, S.; Chi, P.; Lin, H.; Huang, Y.; Xu, Z.; Huang, S. Establishment of a predictive genetic model for estimating chemotherapy sensitivity of colorectal cancer with synchronous liver metastasis. *Cancer Biother Radiopharm* **2013**, *28*, 552-558.
31. Lauvrak, S.U.; Munthe, E.; Kresse, S.H.; Stratford, E.W.; Namlos, H.M.; Meza-Zepeda, L.A.; Myklebost, O. Functional characterisation of osteosarcoma cell lines and identification of mrnas and mirnas associated with aggressive cancer phenotypes. *Br J Cancer* **2013**, *109*, 2228-2236.
32. Dong, L.; Jensen, R.V.; De Rienzo, A.; Gordon, G.J.; Xu, Y.; Sugarbaker, D.J.; Bueno, R. Differentially expressed alternatively spliced genes in malignant pleural mesothelioma identified using massively parallel transcriptome sequencing. *BMC Med Genet* **2009**, *10*, 149.
33. Watson, M.B.; Lind, M.J.; Smith, L.; Drew, P.J.; Cawkwell, L. Expression microarray analysis reveals genes associated with in vitro resistance to cisplatin in a cell line model. *Acta Oncol* **2007**, *46*, 651-658.
34. Karppinen, S.M.; Honkanen, H.K.; Heljasvaara, R.; Riihila, P.; Autio-Harmainen, H.; Sormunen, R.; Harjunen, V.; Vaisanen, M.R.; Vaisanen, T.; Hurskainen, T., *et al.* Collagens xv and xviii show different expression and localisation in cutaneous squamous cell carcinoma: Type xv appears in tumor stroma, while xviii becomes upregulated in tumor cells and lost from microvessels. *Exp Dermatol* **2016**, *25*, 348-354.
35. He, H.T.; Xu, M.; Kuang, Y.; Han, X.Y.; Wang, M.Q.; Yang, Q. Biomarker and competing endogenous rna potential of tumor-specific long noncoding rna in chromophobe renal cell carcinoma. *Onco Targets Ther* **2016**, *9*, 6399-6406.
36. Sun, H.C.; Tang, Z.Y. Angiogenesis in hepatocellular carcinoma: The retrospectives and perspectives. *J Cancer Res Clin Oncol* **2004**, *130*, 307-319.
37. Musso, O.; Rehn, M.; Theret, N.; Turlin, B.; Bioulac-Sage, P.; Lotrian, D.; Campion, J.P.; Pihlajaniemi, T.; Clement, B. Tumor progression is associated with a significant decrease in the expression of the endostatin precursor collagen xviii in human hepatocellular carcinomas. *Cancer Res* **2001**, *61*, 45-49.
38. Qiu, T.; Wang, H.; Wang, Y.; Zhang, Y.; Hui, Q.; Tao, K. Identification of genes associated with melanoma metastasis. *Kaohsiung J Med Sci* **2015**, *31*, 553-561.
